# Organophotoredox-Driven Three-Component Synthesis of β‑Trifluoromethyl β‑Amino Ketones

**DOI:** 10.1021/acs.joc.4c03142

**Published:** 2025-01-30

**Authors:** Pau Sarró, Roser Pleixats, Carolina Gimbert-Suriñach, Adelina Vallribera, Albert Granados

**Affiliations:** Department of Chemistry and Centro de Innovación en Química Avanzada (ORFEO−CINQA), 16719 Universitat Autònoma de Barcelona, Cerdanyola del Vallès, 08193 Barcelona, Spain

## Abstract

In this work, we present a photoredox three-component reaction that enables the synthesis of medicinally relevant β-trifluoromethyl β-amino ketones from a *N*-trifluoroethylhydroxylamine derivative, styrenes and DMSO. Remarkably, fluoromethyl, difluoromethyl and pentafluoroethyl analogues are also accessed using the same reaction conditions. The mechanistic investigations, including radical trapping experiments, cyclic voltammetry, Stern–Volmer quenching studies and isotope labelling experiments support the photoinduced radical/polar crossover and Kornblum-type oxidation mechanisms. Finally, the applicability of the accessed organic skeletons is showcased by notable derivatization reactions.

## Graphical abstract

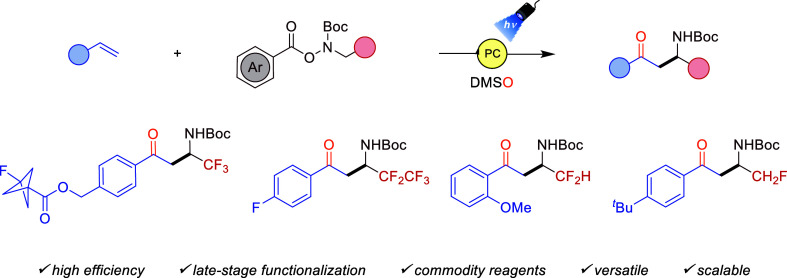

## Introduction

The trifluoromethyl substituent has widespread within organic compounds, representing one of the most popular fluorinated groups.^1^ Particularly, this moiety has proven to be key in pharmaceuticals, agrochemicals and materials chemistry.^2^ The extraordinary properties provided such as lipophilicity or metabolic stability are recognized factors that directly impact the physicochemical behavior and biological activity.^3^ Notably, the α-trifluoromethylamine unit is present in many biologically relevant molecules,^4^ which is not surprising given the unique properties provided when replacing H by fluorine atoms at the α-Me of an amine moiety. The amine basicity modulation and the inherent geometry of this structure constitutes itself as an extraordinarily attractive scaffold in medical chemist’s toolbox. These properties enable α-CF_3_ amines to behave as a bioisostere for amide functional group^5^ with an interesting higher resistance toward proteolytic cleavage (Scheme 1). β-Trifluoromethyl β-amino ketones represent an important class of trifluoromethylated building blocks in biochemistry and pharmacology settings.^6^ Their chemical synthesis typically utilized enolates derived from organic ketone/aldehyde carbonyls via Mannich-type reactions.^7^ However, these protocols based on two-electron processes require metal catalysts,^7a^ operationally demanding conditions or elaborated starting materials. Great efforts have been made to provide diastereo- ^7b–7d^ or enantio-enriched β-trifluoromethyl β-amino ketones, for example asymmetric derivatives can be accessed via *N*-heterocyclic carbene organocatalysis.^8^ Recently, hydroxylamine-derived compounds have proven to be effective nitrogen-centered radical precursors. For example, in 2022 Huang and Xu described iridium-based photoredox methods for the *N*-trifluoromethylamination of organic skeletons.^9^ Particularly, *N*-trifluoroethyl hydroxylamine reagents (Scheme 2A) have shown to serve as suitable N-based radicals under suitable reductive conditions (radical synthon **A** in Scheme 2A).^10^ Remarkably, the generated radical can undergo intramolecular 1,2-hydrogen atom transfer (1,2-HAT) under the appropriate reaction conditions to yield a valuable C-centered radical (**B** in Scheme 2A). Last year, a ruthenium-based reductive quenching photoredox approach using Hantzsch Ester as a sacrificial electron-donor, enabled the *gem*-difluoroallylation of α-trifluoromethylamines by employing captodative α-trifluoromethylstyrenes as radical acceptors.^11^

**Scheme 1:**
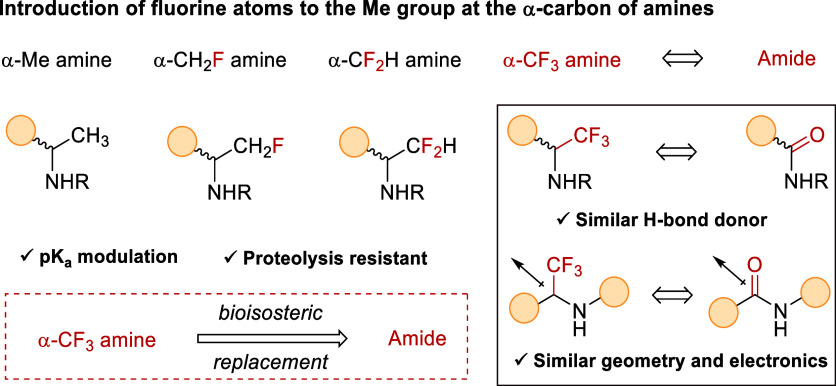
Properties of α-Trifluoromethyl Amines as Bioisosteres of the Amide Functional Group

**Scheme 2:**
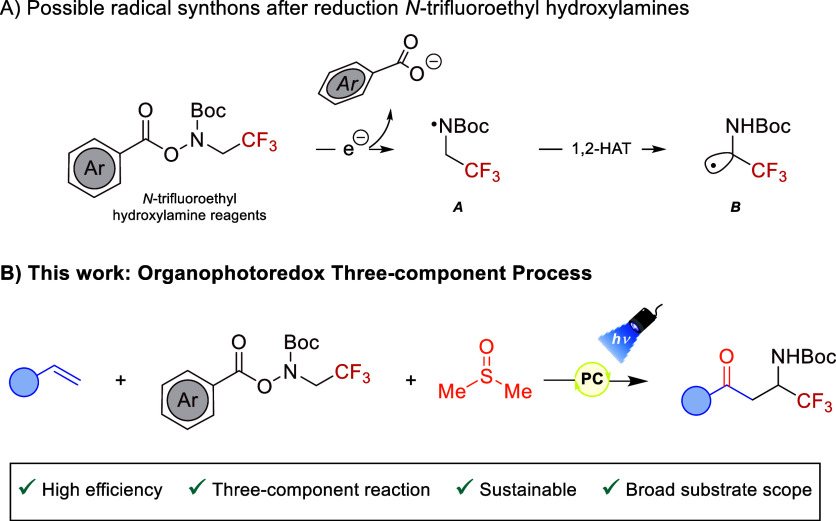
(A) Synthetic Opportunities after Reduction of *N*-Trifluoroethyl Hydroxylamine Reagents; (B) Reaction Design for the Photoinduced Three-Component Synthesis of β-Trifluoromethyl β-Amino Ketones via 1,2-HAT

To date, three-component synthetic methods for assembling β-trifluoromethyl β-amino ketones are unknown yet challenging. We have developed a difunctionalization transformation employing a *N*-trifluoroethyl hydroxylamine derivative as a redox-active species, styrenes, and DMSO (Scheme 2B) through an oxidative quenching photoredox cycle, an underexplored activation mode in this context. This photoredox difunctionalization process represents the first visible light mediated synthesis of β-CF_3_ β-amino carbonyls. Notably, this strategy expedites high molecular complexity in a single step and from simple and readily accessible starting materials. Such chemical scaffolds serve as valuable intermediates with the potential to give rise to more complex and interesting molecules, such as azirines^12^ or pyrazoles.^13^

## Results and Discussion

To evaluate the tenability of this approach, styrene **1a** was selected as model substrate and we selected two different photocatalysts, the organometallic Ir­(ppy)_3_ (where ppy is 2-phenylpyridine) and the organic 4DPAIPN (1,3-dicyano- 2,4,5,6-tetrakis­(diphenylamino)-benzene). Pleasingly, both photocatalysts facilitated the desired transformation without the need for additives in excellent yields (Table 1, entries 1–2). We selected the more accessible and sustainable organic dye as a suitable photocatalyst. Of note, this transformation is completed after only 4 h of illumination (Table 1, entries 2–4). Finally, control studies verified that each component of the reaction was necessary (Table 1, entries 5–7) and the involvement of open-shell species since the addition of TEMPO impaired the reactivity (Table 1, entry 8).

**Table 1:**
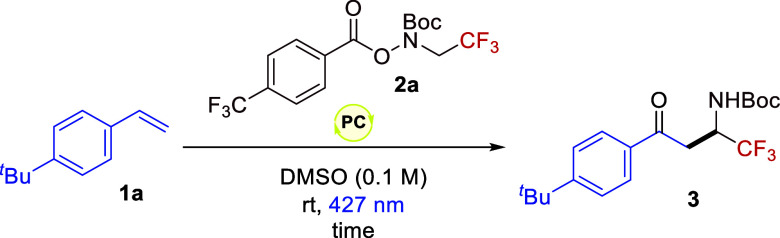
Keto-Alkylation of Styrenes: Optimization of Reaction Conditions^f^

entry^a^	PC	time (h)	yield (%)^b^
1	Ir(ppy)_3_	16	97
2	4DPAIPN	16	98
3	4DPAIPN	4	97
4	4DPAIPN	1	46
5	none	4	0
6^c^	4DPAIPN	4	0
7^d^	4DPAIPN	4	0
8^e^	4DPAIPN	4	0

a Reaction conditions: **1a** (0.1 mmol, 1 equiv), **2a** (0.2 mmol, 2 equiv), PC (2 mol %) in 1 mL of degassed DMSO (*c* = 0.1 M) under 427 nm Kessil lamp irradiation at rt.

b Yields determined by^1^H NMR analysis.

c No light experiment.

d MeCN as solvent and ambient atmosphere.

e 5 equiv of TEMPO added.

f PC = photocatalyst.

With suitable conditions established, the scope of this difunctionalization process was assessed. First, we focused on examining styrene diversity (Table 2). An array of olefinic substrates were competent in the presented difunctionalization process. Monosubstitutions at *para* position afforded the desired compounds from good to excellent yields (**3**–**9**). Both electron-donating and moderate electron-withdrawing groups could be well tolerated.

**Table 2:**
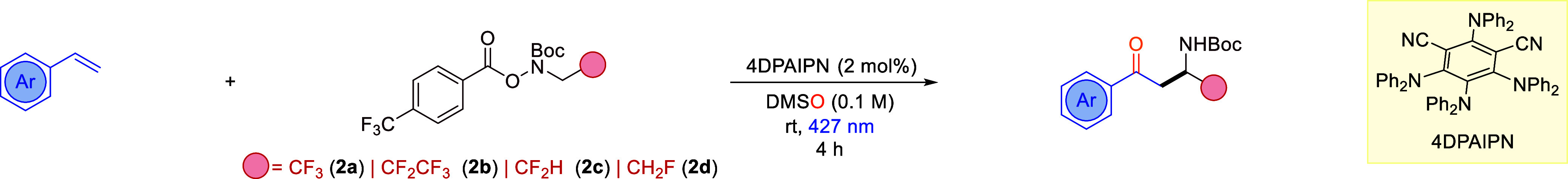

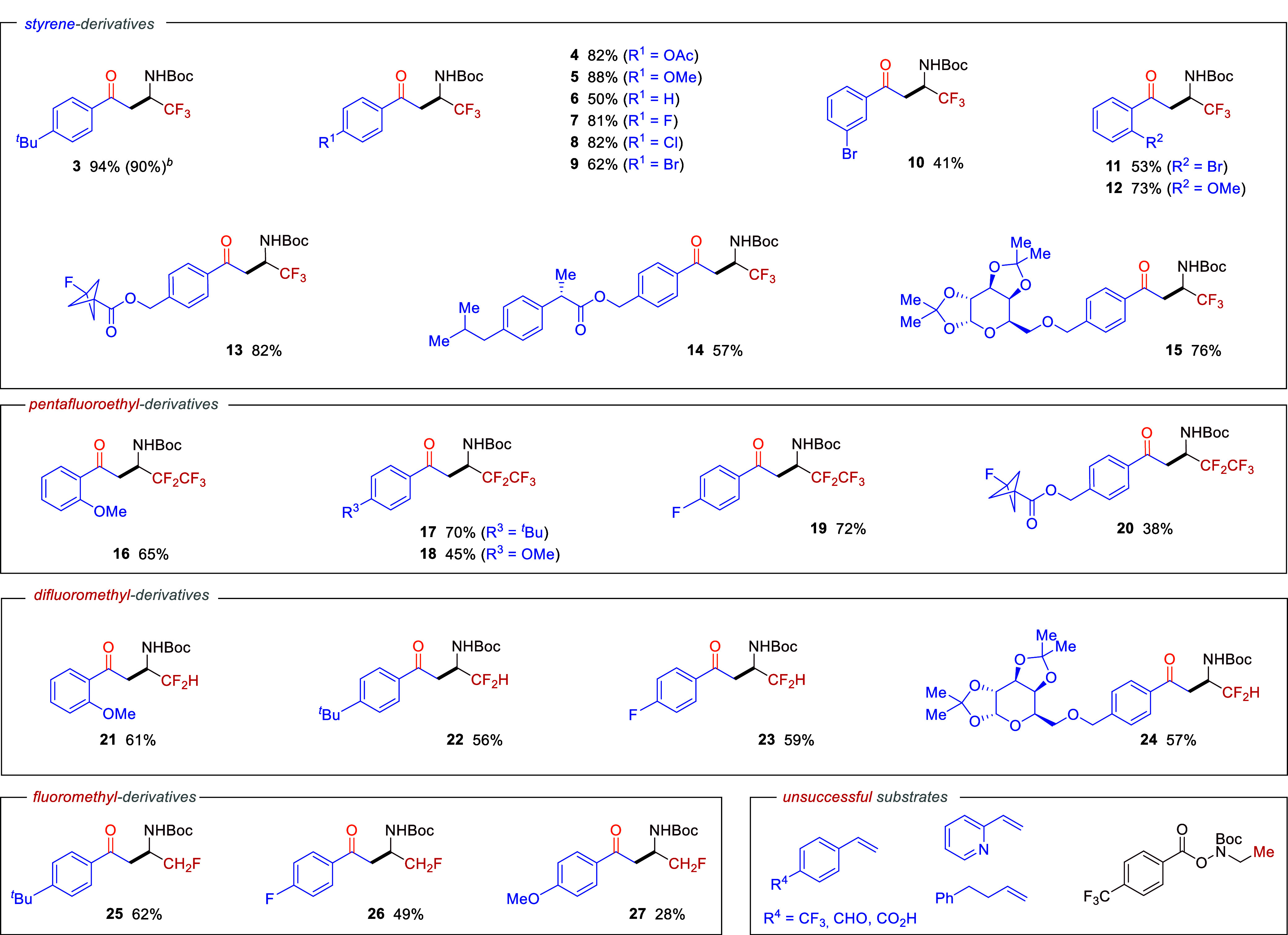
Substrate Scope Evaluation for the Photoinduced Three-Component Keto-Alkylation Process^a^

a General reaction conditions: **1** (0.25 mmol, 1 equiv), **2a**–**d** (0.50 mmol, 2 equiv), 4DPAIPN (2 mol %) in DMSO (2.5 mL, 0.1 M), under Kessil lamp irradiation (λ_max_ = 427 nm) at rt for 4hours.

b From 2.0 mmol of 4-*tert*-butylstyrene.

Unfortunately, nonaromatic alkenes and strong electron-withdrawing groups tethered to the phenyl ring of the styrene, such as formyl, trifluoromethyl or carboxyl, and pyridine-derived alkenes did not produce the product, thus falling beyond the substrate scope of this transformation (see Table 2 and Supporting Information). This can be attributed to the electronic nature of the benzylic radical intermediate species that has to be oxidized in the last step of the mechanism, necessary to close the photocatalytic cycle (vide infra). Remarkably, this method is not limited to 4-substituted styrenes, but also *ortho*- and *meta*-substituted derivatives also produced the desired carbonyls in yields ranging from 41% to 73%. The *para*- (**9**), *meta*- (**10**), and *ortho*- (**11**) brominated regioisomers were isolated in 62%, 41%, and 53% yields, respectively. These results demonstrate the general applicability of this protocol, despite the lower yields observed in certain cases due to steric and electronic factors. Finally, complex styrene-derived small molecules such as the medicinally relevant bicyclopentane unit (**13**), anti-inflammatory ibuprofen (**14**) and d-galactopyranose (**15**) can be converted to the corresponding β-trifluoromethyl β-amino ketones in high yields, proving its potential application for late-stage functionalization environments. Given the efficiency of the developed protocol, we scaled up 8-fold in batch using regular glassware and the same light source. Using 4-*tert*-butylstyrene as model alkene we observed same efficacy (90% yield, **3**).

Next, given that high efficiency of the reaction we sought to explore further the reactivity by replacing the CF_3_ group with other relevant fluorinated scaffolds (Table 2), such as pentafluoroethyl (CF_2_CF_3_), difluoromethyl (CF_2_H), and fluoromethyl (CH_2_F). These substitutions were chosen to investigate the impact of varying degrees of fluorination on the reaction outcomes. The introduction of the CF_2_CF_3_ group is expected to influence the electronic and steric properties more significantly compared to the CF_3_ group.^14^ Substitution with the less fluorinated moiety CF_2_H, normally provides insight into the role of the hydrogen atom in modulating reactivity and selectivity.^15^ Lastly, the single fluorine atom in the CH_2_F group was chosen to explore the impact of minimal fluorination, offering unique electronic and steric effects and serving as a CH_2_OH bioisostere.^16^ Overall, these substitutions will allow for a comprehensive understanding of how varying degrees and patterns of fluorination can influence the reactivity, selectivity, and overall efficiency. Initially, novel redox-active species **2b** (Table 2) was synthesized in good yield (see Supporting Information for details). Then, when applying the optimal conditions, we successfully synthesized five different products by varying the substitution patterns on the aromatic phenyl ring (**16**–**20**). The yields of these products ranged from 38% to 72%, demonstrating the influence of substituent positioning and electronic effects on the reaction efficiency. First, *para*-styrenes yielded the desired products from moderate to good yields, observing best results with moderate electron-donating groups (^*t*^Bu, **17**) and moderate electron-withdrawing (F, **19**). The strong activating OMe group facilitated the formation of the desired product, albeit in slightly less efficacy. Then, an *ortho*-substituted styrene was efficiently keto-alkylated in a good 65% yield (**16**). Finally, a more complex styrene derivative also gave access to the desired pentafluoroalkylated compound (**20**) in moderate yield. Subsequently, we evaluated the tenability of this difunctionalization process utilizing the difluoromethylated reagent **2c**. In general, this transformation allowed the formation of the targeted molecules, although in less efficacy. In contrast to similar organic skeletons with the CF_2_H unit present at a β-carbonyl position,^17^ the synthesized compounds could be purified by flash column chromatography without compromising its structure. Importantly, this acetophenone-derivative synthesis is not only accessed from simple styrene, but also more complex organic cores can be prepared, such as d-galactopyranose (**24**). Finally, the substrate scope was extended to the preparation of monofluorinated analogues. In this case, we unlocked the preparation of the monofluorinated redox-active hydroxylamine **2d** (see Supporting Information), and then we demonstrated that our conditions could be also successfully applied. Significantly, styrenes with different electronic properties were amenable and we could prepare **25**, **26** and **27** in good chemical yields (28–62%). Nonfluorinated hydroxylamines were ineffective (see Supporting Information).

We propose that a photoinduced net-neutral radical/polar crossover^18^ process enables this difunctionalization reaction via oxidative quenching. To prove our hypothesis, we conducted mechanistic experiments including Stern–Volmer luminescence quenching studies, cyclic voltammetry, isotopic labeling and radical trapping experiments. First, to determine which species is quenching the photocatalyst’s excited state, binary mixtures of photocatalyst and model reagents were prepared. Fluorimetry experiments determined that the photocatalyst/**2a** combination showed more effective 4DPAIPN* quenching than the mixture with the alkene. This resulted in an observed *K*_SV_ of 96.8 M^–1^ (see Scheme 3A and Supporting Information). Next, to investigate the feasibility of a single-electron transfer (SET) event between 4DPAIPN* and hydroxylamine derived compounds **2a**–**d** we conducted cyclic voltammetry experiments. The redox potentials of the fluorinated redox active reagents (**2a**–**d**) were measured (see Supporting Information for details). The set of the four species showed irreversible reduction waves with *E*_red_ values that ranged from −1.90 to −1.98 V vs Fc^+^/Fc (Scheme 3B). Importantly, these results support that 4DPAIPN* can thermodynamically (*E*_red_ PC^*/^PC^^•^+^ = −1.98 V vs Fc^+^/Fc) undergo SET with reagents **2a**–**d**. Finally, the radical and photochemical nature of this transformation was confirmed when the reaction was inhibited in the presence of TEMPO or in the absence of photocatalyst or light illumination (see Table 1 entries 5–7).

**Scheme 3:**
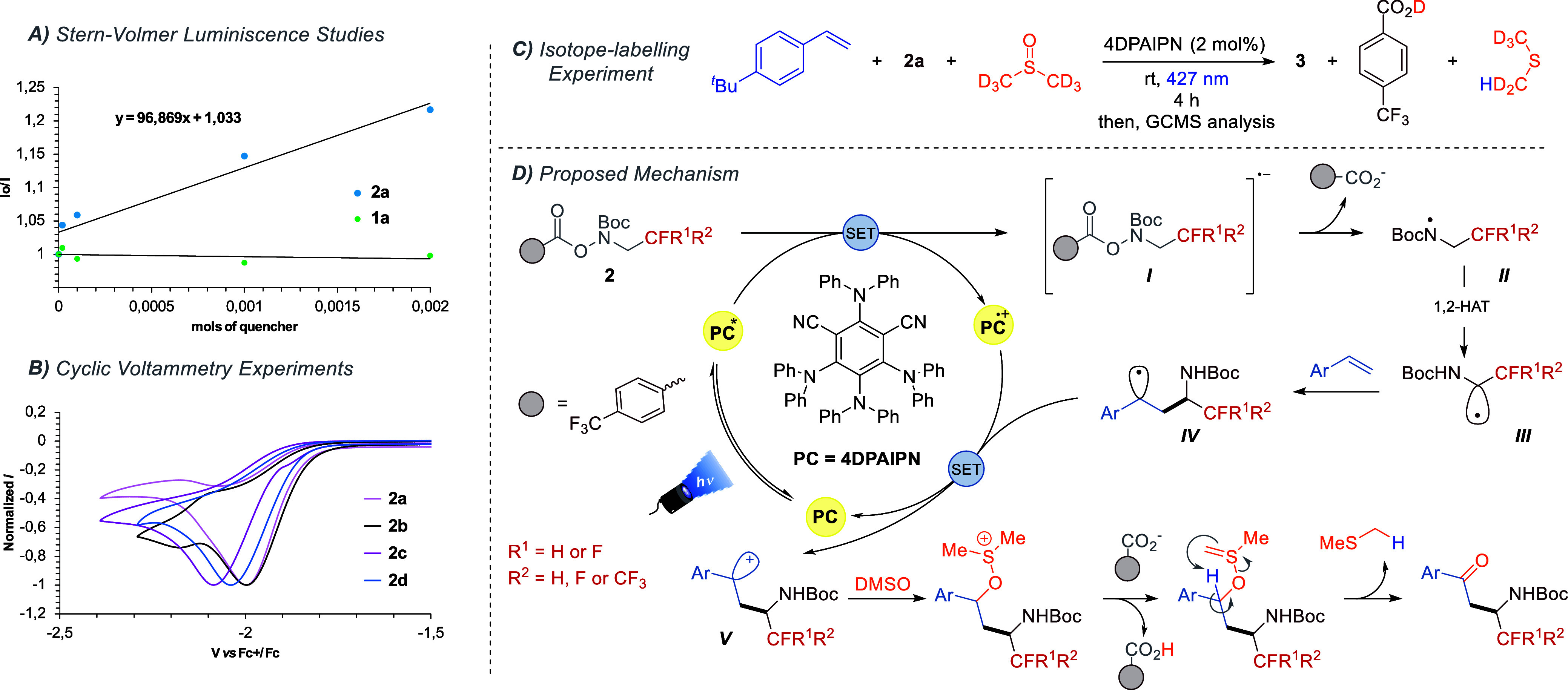
(A) Stern–Volmer Plots for Luminescence Quenching of 4DPAIPN by *p*-*tert*-Butylstyrene **1a** (green) and Redox-Active Species **2a** (blue) (See the Supporting Information for Experimental Details); (B) Cyclic Voltammetry of Redox Active Species **2a**–**d**, See the Supporting Information for Further Details; (C) Isotope Labelling Experiment; (D) Proposed Mechanism for the Three-Component Synthesis of β-Alkyl β-Amino Ketones

To gain deeper understanding into the oxidation process mediated by DMSO, we performed isotope-labeling experiments using DMSO-*d*_6_ as the solvent (Scheme 3C). Notably, alongside the desired compound **3**, we observed the formation of pentadeuterated dimethyl sulfide as the main side product, as well as deuterated *p*-trifluoromethylbenzoic acid by GCMS (see Supporting Information). These findings suggest that the benzoate derivative abstracts a deuterium atom from the in situ-formed sulfonium intermediate (Scheme 3D).

Overall, the presented mechanistic evidence depicted above and related literature^10a,17,18^ favor to detail the operative mechanism for this transformation in Scheme 3D. Upon photoirradiation under Kessil lamp, organic 4DPAIPN species achieves its excited state (PC*) that accomplishes single electron reduction of **2**, generating the radical anion intermediate ***I***. Next, species ***I*** undergoes O–N bond scission producing the N-centered radical ***II*** and the benzoate-derivative. Subsequently, ***II*** produces a synthetically useful carbon-centered radical ***III*** via efficient 1,2-hydrogen atom transfer (1,2-HAT) process, which engages in a Giese addition with an alkene (***IV***). The Giese adduct ***IV*** is oxidized further by PC^^•^+^ through SET event, producing the carbocation **V** and returning the photocatalyst back to its neutral ground state. Finally, the resulting cationic species ***V*** is trapped by DMSO, leading to the formation of a sulfonium cation. Subsequent deprotonation by the benzoate^19^ species generates a sulfonium ylide, which undergoes an intramolecular attack on the benzylic proton, ultimately yielding the final ketone.

To showcase the versatility of the presented photoredox method, a series of different derivatization reactions were conducted. These reactions highlighted the product’s broad applicability and efficiency in modifying substrate **3**, prepared in a 2 mmol scale, through either the carbonyl or the nitrogen moiety under mild reaction conditions (Scheme 4). First, NHBoc deprotection (**28**) and carbonyl reduction (**29**) afforded the corresponding products in excellent yields. Next, we evaluated the preparation of interesting heterocyclic compounds. Pyrazole **30** was accessed^13^ in 51% yield via diazonium salt intermediate, while strained trifluoromethylated azidirine **31** was isolated after a two-step process^12^ from **3** in 84% yield.

**Scheme 4:**
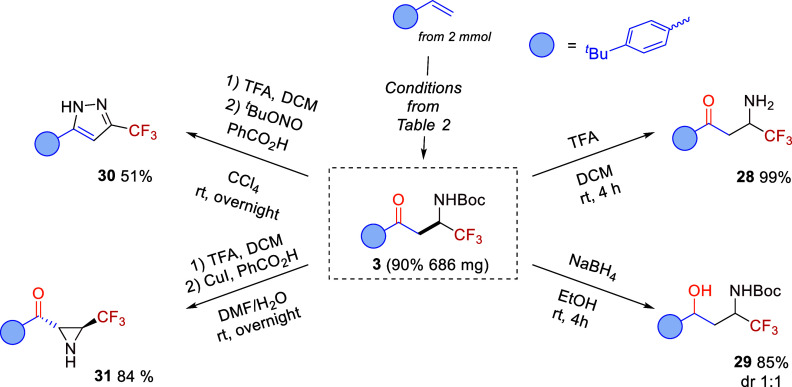
Downstream Transformations from **3** (See Supporting Information for Details)

## Conclusions

In summary, we have unraveled the synthesis of β-trifluoromethyl β-amino ketones from readily available styrenes in a single step via organophotoredox conditions. This three-component protocol increases molecular complexity not only in β-CF_3_ β-amino ketones but also in CF_2_H, CF_2_CF_3_, and CH_2_F settings. Also, we demonstrate the first application of an oxidative quenching photoredox cycle in a three-component reaction with fluorinated hydroxylamine reagents **2a**–**d**, offering a mechanistic alternative to reductive quenching systems. The efficiency and robustness of the reaction are remarkable, achieving high yields in both small- and large-scale synthesis, and it is applicable to late-stage functionalization. Mechanistic investigations support the operation of the reaction via photoinduced radical/polar crossover, and the applicability of this method is showcased by downstream derivatization reactions.

## Experimental Section

### General Information

All chemical transformations requiring inert atmosphere were done using Schlenk line techniques. For violet light irradiation, a Kessil PR160-violet LED lamp (30 W High Luminous DEX 2100 LED, λ_max_ = 427 nm) was placed 4 cm away from the reaction vials. Photoinduced reactions were performed using 4 or 8 mL Chemglass vials (15–425 Green Open Top Cap, TFE Septa). Reactions were monitored by TLC or NMR. TLC analysis was performed using hexanes/EtOAc mixtures as the eluent unless specified and visualized using UV light and/or Vanillin solution. The cyclic voltammetry (CV) experiments were performed with a BioLogic SP-50 Single Channel Potentiostat in a one-compartment three-electrode setup using a glassy carbon disk as the working electrode (ø = 3 mm), platinum wire as the auxiliary electrode, and SCE or AgNO_3_/Ag (0.01 M AgNO_3_, 0.1 M [^*n*^Bu_4_N]­PF_6_ (TBAPF_6_), MeCN) as reference electrodes. CV were performed at room temperature using the appropriate solvent, degassing with argon for 60 s and using TBAPF_6_ as supporting electrolyte (0.1 M). All the experiments were referred to ferrocene as an internal standard. Polishing of the working electrode has been done using an alumina polishing pad with a solution of 0.05 μm alumina in water (purchased from BAS INC.). NMR experiments (^1^H, ^13^C, ^19^F) were performed in the Servei de Ressonància Magnètica Nuclear, UAB, using NEO 300, NEO 400, NEO 500, or NEO 600 spectrometers. Chemical shifts are referenced to residual, nondeuterated CHCl_3_ (δ 7.26 in ^1^H NMR and 77.16 in ^13^C NMR). The HRMS (ESI+) and elemental analyses were done by the Servei d’Anàlisi Química of UAB and Parque Científico Tecnológico of UBU. HRMS is determined by a Bruker microTOF-QII mass spectrometer (fly time analyzer) through positive electrospray ionization. IR spectra were recorded on an FT-IR PerkinElmer using either neat oil or solid products. Fluorescence measurements were obtained using septa-capped UV-Quartz cuvettes (10 mm path length) from Hellma Analytics and were recorded in a PerkinElmer LS 55 Fluorescence Spectrometer attached to a PTP 1 Peltier Temperature Programmer maintaining the temperature at 25 °C. Melting points (°C) are uncorrected. Deuterated NMR solvents were purchased from Eurisotop. Dry solvents were obtained from Aldrich or Fisher and used as received. Bulk DCM, EtOAc and hexane were purchased from VWR. Chemicals were purchased from Fluorochem and Merck and used as received unless specified.

### General Procedure for the Photoinduced Synthesis of Fluorinated β-Amino Ketones (**3**–**27**)

To an 8 mL Chemglass vial equipped with a magnetic stirring bar, the corresponding styrene **1**(0.25 mmol, 1.0 equiv), 4DPAIPN (0.02 equiv) and the corresponding hydroxylamine **2** (0.50 mmol, 2.0 equiv) were added. Then, 2.5 mL of dry DMSO were added under inert atmosphere and the reaction was degassed with Argon for 20 s. The reaction mixture was irradiated for 4 h with a 427 nm Kessil PR160-purple LED as described in the “Workflow” section (Figure S1 in the Supporting Information). The temperature of the reaction was maintained at approximately 25 °C via a fan. Upon completion, the reaction mixture was diluted with AcOEt (10 mL) and washed with brine (3 × 10 mL). The organic layer was dried over anhydrous Na_2_SO_4_, filtered and concentrated under reduced pressure. The crude mixture was subjected to flash column chromatography purification using hexanes/EtOAc mixtures to yield the desired compound. Caution! Proper protective eyewear, specifically goggles designed to shield against the emitted wavelengths, was used to prevent potential eye damage from prolonged exposure to the light source. Researchers are advised to take similar precautions when reproducing this work.

#### *Tert*-Butyl (4-(4-(*Tert*-Butyl)­phenyl)-1,1,1-trifluoro-4-oxobutan-2-yl)­carbamate **(3)**

Prepared according to the General Procedure from the corresponding styrene **1a** (40 mg, 0.25 mmol, 1.0 equiv) and **2a** (194 mg, 0.5 mmol, 2.0 equiv). After purification by flash column chromatography (hexane/EtOAc 10:1), the title compound **3** was obtained as a white solid (88 mg, 0.24 mmol, 94%). *R*_*f*_ = 0.49 (silica gel, *n*-hexane/EtOAc, 10:1 (v/v)); mp 112–114 °C. ^1^H NMR (500 MHz, CDCl_3_): δ (ppm) = 7.89 (d, *J* = 8.5 Hz, 2H), 7.51 (d, *J* = 8.5 Hz, 2H), 5.36 (d, *J* = 7.5 Hz, 1H), 4.99–4.84 (m, 1H), 3.42–3.25 (m, 2H), 1.45 (s, 9H), 1.36 (s, 9H). ^13^C­{^1^H} NMR (126 MHz, CDCl_3_): δ (ppm) = 194.9, 157.7, 154.7, 133.6, 128.1, 125.8, 125.2 (q, *J* = 281.8 Hz), 80.6, 49.1 (q, *J* = 31.6 Hz), 36.6, 35.2, 31.0, 28.2.^19^F­{^1^H} NMR (376 MHz, CDCl_3_): δ (ppm) = −75.7. FT-IR (cm^–1^, neat, ATR), ν̃ = 3323, 2966, 2922, 2872, 1688, 1532, 1367, 1349, 1305, 1249, 1155, 1108, 1054, 1027, 991. HRMS (ESI+) calcd for C_19_H_27_F_3_NO_3_ [M + H]^+^: 374.1938; found, 374.1943.

#### 4-(3-((*Tert*-Butoxycarbonyl)­amino)-4,4,4-trifluorobutanoyl)­phenyl Acetate **(4)**

Prepared according to the General Procedure from the corresponding styrene **1b** (41 mg, 0.25 mmol, 1.0 equiv) and **2a** (194 mg, 0.5 mmol, 2.0 equiv). After purification by flash column chromatography (hexane/EtOAc 10:1), the title compound **4** was obtained as a pale yellow solid (76 mg, 0.20 mmol, 82%). *R*_*f*_ = 0.10 (silica gel, *n*-hexane/EtOAc, 7:1 (v/v)); mp 131–133 °C. ^1^H NMR (500 MHz, CDCl_3_): δ (ppm) = 8.00 (d, *J* = 8.8 Hz, 2H), 7.25 (d, *J* = 8.8 Hz, 2H), 5.22 (m, 1H), 4.98–4.89 (m, 1H), 3.36–3.29 (m, 2H), 2.36 (s, 3H), 1.47 (s, 9H). ^13^C­{^1^H} NMR (126 MHz, CDCl_3_): δ (ppm) = 193.9, 168.7, 154.9, 154.9, 133.7, 129.8, 125.2 (q, *J* = 281.7 Hz), 122.1, 80.8, 49.4 (q, *J* = 28.9 Hz), 36.8, 28.2, 21.2.^19^F­{^1^H} NMR (376 MHz, CDCl_3_): δ (ppm) = −75.7. FT-IR (cm^–1^, neat, ATR), ν̃ = 3342, 2980, 2931, 1761, 1697, 1600, 1528, 1368, 1299, 1248, 1195, 1163, 1125, 1016. HRMS (ESI+) calcd for C_17_H_20_F_3_NO_5_Na [M + Na]+: 398.1186; found, 398.1195.

#### *Tert*-Butyl (1,1,1-Trifluoro-4-(4-methoxyphenyl)-4-oxobutan-2-yl)­carbamate **(5)**

Prepared according to the General Procedure from the corresponding styrene **1c** (34 mg, 0.25 mmol, 1.0 equiv) and **2a** (194 mg, 0.5 mmol, 2.0 equiv). After purification by flash column chromatography (hexane/EtOAc 7:3), the title compound **5** was obtained as a pale yellow solid (76 mg, 0.22 mmol, 88%). *R*_*f*_ = 0.13 (silica gel, *n*-hexane/EtOAc, 10:1 (v/v)); mp 149–151 °C. ^1^H NMR (500 MHz, CDCl_3_): δ (ppm) = 7.94 (d, *J* = 9.0 Hz, 2H), 6.97 (d, *J* = 9.1 Hz, 2H), 5.32 (s, 1H), 4.95–4.86 (m, 1H), 3.90 (s, 3H), 3.36–3.25 (m, 2H), 1.46 (s, 9H). ^13^C­{^1^H} NMR (126 MHz, CDCl_3_): δ (ppm) = 193.7, 164.1, 154.7, 130.5, 129.3, 125.2 (q, *J* = 281.7 Hz), 114.0, 80.6, 55.6, 49.2 (q, *J* = 29.8 Hz), 36.3, 28.2.^19^F­{^1^H} NMR (376 MHz, CDCl_3_): δ (ppm) = −75.7. FT-IR (cm^–1^, neat, ATR), ν̃ = 3318, 2974, 2983, 2844, 1680, 1602, 1577, 1511, 1421, 1362, 1291, 1248, 1234, 1156, 1119, 1026, 989. HRMS (ESI+) calcd for C_16_H_21_F_3_NO_4_ [M + H]^+^: 348.1417; found, 348.1426.

#### *Tert*-Butyl (1,1,1-Trifluoro-4-oxo-4-phenylbutan-2-yl)­carbamate **(6)**

Prepared according to the General Procedure from the corresponding styrene **1d** (26 mg, 0.25 mmol, 1.0 equiv) and **2a** (194 mg, 0.5 mmol, 2.0 equiv). After purification by flash column chromatography (hexane/EtOAc 10:1), the title compound **6** was obtained as a white solid (40 mg, 0.13 mmol, 50%). *R*_*f*_ = 0.31 (silica gel, *n*-hexane/EtOAc, 10:1 (v/v)); mp 129–131 °C. ^1^H NMR (500 MHz, CDCl_3_): δ (ppm) = 7.96 (d, *J* = 8.3 Hz, 2H), 7.63 (t, *J* = 8.7 Hz, 1H), 7.51 (t, *J* = 8.3 Hz, 2H), 5.29 (d, *J* = 9.8 Hz, 1H), 4.98–4.89 (m, 1H), 3.41–3.32 (m, 2H), 1.46 (s, 9H). ^13^C­{^1^H} NMR (126 MHz, CDCl_3_): δ (ppm) = 195.2, 154.7, 136.2, 133.8, 128.9, 128.1, 125.2 (q, *J* = 282.2 Hz), 80.7, 49.1 (q, *J* = 31.7 Hz), 36.8, 28.2.^19^F­{^1^H} NMR (376 MHz, CDCl_3_): δ (ppm) = −75.8. FT-IR (cm^–1^, neat, ATR), ν̃ = 3391, 2982, 2926, 1713, 1685, 1513, 1354, 1317, 1301, 1286, 1241, 1179, 1149, 1048, 1022, 999. HRMS (ESI+) calcd for C_15_H_19_F_3_NO_3_ [M + H]^+^: 318.1312; found, 318.1309.

#### *Tert*-Butyl (1,1,1-Trifluoro-4-(4-fluorophenyl)-4-oxobutan-2-yl)­carbamate **(7)**

Prepared according to the General Procedure from the corresponding styrene **1e** (31 mg, 0.25 mmol, 1.0 equiv) and **2a** (194 mg, 0.5 mmol, 2.0 equiv). After purification by flash column chromatography (hexane/EtOAc 10:1), the title compound **7** was obtained as a white solid (67 mg, 0.20 mmol, 81%). *R*_*f*_ = 0.36 (silica gel, *n*-hexane/EtOAc, 10:1 (v/v)); mp 143–144 °C. ^1^H NMR (500 MHz, CDCl_3_): δ (ppm) = 7.99 (dd, *J* = 8.9, 5.3 Hz, 2H), 7.18 (t, *J* = 6.7 Hz, 2H), 5.25 (d, *J* = 10.5 Hz, 1H), 4.94–4.91 (m, 1H), 3.39–3.29 (m, 2H), 1.46 (s, 9H). ^13^C­{^1^H} NMR (126 MHz, CDCl_3_): δ (ppm) = 193.5, 166.1 (d, *J* = 256.0 Hz), 154.7, 132.6 (d, *J* = 3.2 Hz), 130.8 (d, *J* = 9.2 Hz), 125.1 (q, *J* = 281.8 Hz), 116.0 (d, *J* = 22.1 Hz), 80.8, 49.1 (q, *J* = 30.8 Hz), 36.8, 28.2.^19^F­{^1^H} NMR (376 MHz, CDCl_3_): δ (ppm) = −103.8, −75.8. FT-IR (cm^–1^, neat, ATR), ν̃ = 3354, 2987, 2940, 2920, 1696, 1686, 1594, 1522, 1346, 1300, 1285, 1250, 1231, 1158, 115, 1060, 993. HRMS (ESI+) calcd for C_15_H_17_F_4_NO_3_Na [M + Na]^+^: 358.1037; found, 358.1050.

#### *Tert*-Butyl (4-(4-Chlorophenyl)-1,1,1-trifluoro-4-oxobutan-2-yl)­carbamate **(8)**

Prepared according to the General Procedure from the corresponding styrene **1f** (35 mg, 0.25 mmol, 1.0 equiv) and **2a** (194 mg, 0.5 mmol, 2.0 equiv). After purification by flash column chromatography (hexane/EtOAc 10:1), the title compound **8** was obtained as a white solid (72 mg, 0.21 mmol, 82%). *R*_*f*_ = 0.39 (silica gel, *n*-hexane/EtOAc, 10:1 (v/v)); mp 64–66 °C. ^1^H NMR (500 MHz, CDCl_3_): δ (ppm) = 7.90 (d, *J* = 8.7 Hz, 2H), 7.49 (d, *J* = 8.7 Hz, 2H), 5.22 (m, 1H), 4.95–4.89 (m, 1H), 3.35–3.29 (m, 2H), 1.46 (s, 9H). ^13^C­{^1^H} NMR (126 MHz, CDCl_3_): δ (ppm) = 193.9, 154.6, 140.4, 134.5, 129.5, 129.2, 125.1 (q, *J* = 281.7 Hz), 80.8, 49.1 (q, *J* = 30.2 Hz), 36.9, 28.2.^19^F­{^1^H} NMR (376 MHz, CDCl_3_): δ (ppm) = −75.8. FT-IR (cm^–1^, neat, ATR), ν̃ = 3360, 2967, 2924, 2854, 1698, 1524, 1492, 1368, 1324, 1249, 1145, 1080, 1049, 1013, 972. HRMS (ESI+) calcd for C_15_H_17_ClF_3_NO_3_Na [M + Na]^+^: 374.0741; found, 374.0750.

#### *Tert*-Butyl (4-(4-Bromophenyl)-1,1,1-trifluoro-4-oxobutan-2-yl)­carbamate **(9)**

Prepared according to the General Procedure from the corresponding styrene **1g** (46 mg, 0.25 mmol, 1.0 equiv) and **2a** (194 mg, 0.5 mmol, 2.0 equiv). After purification by flash column chromatography (hexane/EtOAc 10:1), the title compound **9** was obtained as a pale yellow solid (62 mg, 0.16 mmol, 62%). *R*_*f*_ = 0.42 (silica gel, *n*-hexane/EtOAc, 10:1 (v/v)); mp 136–138 °C. ^1^H NMR (500 MHz, CDCl_3_): δ (ppm) = 7.82 (d, *J* = 8.7 Hz, 2H), 7.65 (d, *J* = 8.6 Hz, 2H), 5.23 (d, *J* = 10.4 Hz, 1H), 4.95–4.87 (m, 1H), 3.38–3.28 (m, 2H), 1.46 (s, 9H). ^13^C­{^1^H} NMR (126 MHz, CDCl_3_): δ (ppm) = 194.1, 154.6, 134.9, 132.2, 129.6, 129.1, 125.1 (q, *J* = 281.7 Hz), 80.8, 49.1 (q, *J* = 31.3 Hz), 36.9, 28.2.^19^F­{^1^H} NMR (376 MHz, CDCl_3_): δ (ppm) = −75.8. FT-IR (cm^–1^, neat, ATR), ν̃ = 3348, 2957, 2923, 2854, 1704, 1687, 1493, 1460, 1364, 1302, 1275, 1259, 1180, 1079, 1022, 1011, 965. HRMS (ESI+) calcd for C_15_H_17_BrF_3_NO_3_Na [M + Na]^+^: 418.0236; found, 418.0244.

#### *Tert*-Butyl (4-(3-Bromophenyl)-1,1,1-trifluoro-4-oxobutan-2-yl)­carbamate **(10)**

Prepared according to the General Procedure from the corresponding styrene **1h** (46 mg, 0.25 mmol, 1.0 equiv) and **2a** (194 mg, 0.5 mmol, 2.0 equiv). After purification by flash column chromatography (hexane/EtOAc 10:1), the title compound **10** was obtained as a white solid (40 mg, 0.10 mmol, 41%). *R*_*f*_ = 0.31 (silica gel, *n*-hexane/EtOAc, 10:1 (v/v)); mp 129–131 °C. ^1^H NMR (500 MHz, CDCl_3_): δ (ppm) = 8.08 (s, 1H), 7.88 (d, *J* = 7.9 Hz, 1H), 7.76 (d, *J* = 8.0 Hz, 1H), 7.40 (t, *J* = 7.9 Hz, 1H), 5.19 (d, *J* = 9.5 Hz, 1H), 5.06–4.85 (m, 1H), 3.32 (s, 2H), 1.47 (s, 9H). ^13^C­{^1^H} NMR (126 MHz, CDCl_3_): δ (ppm) = 193.8, 154.6, 137.8, 136.7, 131.2, 130.4, 126.6, 125.1 (q, *J* = 282.1 Hz), 123.3, 80.8, 49.0 (q, *J* = 32.2 Hz), 37.1, 28.2.^19^F­{^1^H} NMR (376 MHz, CDCl_3_): δ (ppm) = −75.8. FT-IR (cm^–1^, neat, ATR), ν̃ = 3349, 2979, 1701, 1689, 1522, 1307, 1282, 1225, 1150, 1118, 1049, 1023, 991. HRMS (ESI+) calcd for C_15_H_17_BrF_3_NO_3_Na [M + Na]^+^: 418.0236; found, 418.0244.

#### *Tert*-Butyl (4-(2-Bromophenyl)-1,1,1-trifluoro-4-oxobutan-2-yl)­carbamate **(11)**

Prepared according to the General Procedure from the corresponding styrene **1i** (46 mg, 0.25 mmol, 1.0 equiv) and **2a** (194 mg, 0.5 mmol, 2.0 equiv). After purification by flash column chromatography (hexane/EtOAc 10:1), the title compound **11** was obtained as a white solid (53 mg, 0.13 mmol, 53%). *R*_*f*_ = 0.31 (silica gel, *n*-hexane/EtOAc, 10:1 (v/v)); mp 115–117 °C. ^1^H NMR (500 MHz, CDCl_3_): δ (ppm) = 7.65 (d, *J* = 7.9 Hz, 1H), 7.42 (d, *J* = 3.2 Hz, 2H), 7.39–7.35 (m, 1H), 5.17 (d, *J* = 8.7 Hz, 1H), 4.90–4.81 (m, 1H), 3.41–3.29 (m, 2H), 1.48 (s, 9H). ^13^C­{^1^H} NMR (126 MHz, CDCl_3_): δ (ppm) = 199.1, 154.5, 140.4, 133.9, 132.4, 129.0, 127.7, 124.9 (q, *J* = 281.7 Hz), 118.8, 80.8, 49.3 (q, *J* = 30.8 Hz), 40.9, 28.2.^19^F­{^1^H} NMR (376 MHz, CDCl_3_): δ (ppm) = −76.0. FT-IR (cm^–1^, neat, ATR), ν̃ = 3316, 2987, 2932, 1709, 1692, 1531, 1370, 1355, 1305, 1250, 1182, 1156, 1120, 1023, 995. HRMS (ESI+) calcd for C_15_H_17_BrF_3_NO_3_Na [M + Na]^+^: 418.0236; found, 418.0244.

#### *Tert*-Butyl (1,1,1-Trifluoro-4-(4-methoxyphenyl)-4-oxobutan-2-yl)­carbamate **(12)**

Prepared according to the General Procedure from the corresponding styrene **1j** (34 mg, 0.25 mmol, 1.0 equiv) and **2a** (194 mg, 0.5 mmol, 2.0 equiv). After purification by flash column chromatography (hexane/EtOAc 5:1), the title compound **12** was obtained as a pale yellow solid (63 mg, 0.18 mmol, 73%). *R*_*f*_ = 0.30 (silica gel, *n*-hexane/EtOAc, 6:1 (v/v)); mp 114–116 °C. ^1^H NMR (500 MHz, CDCl_3_): δ (ppm) = 7.75 (dd, *J* = 7.7, 1.9 Hz, 1H), 7.53 (td, *J* = 8.5, 1.9 Hz, 1H), 7.06–7.00 (m, 2H), 5.20 (d, *J* = 8.7 Hz, 1H), 4.93–4.78 (m, 1H), 3.95 (s, 3H), 3.46–3.41 (m, 1H), 3.29–3.21 (m, 1H), 1.43 (s, 9H). ^13^C­{^1^H} NMR (126 MHz, CDCl_3_): δ (ppm) = 197.0, 158.7, 154.7, 134.5, 130.9, 126.9, 125.3 (q, *J* = 281.7 Hz), 121.0, 111.6, 80.4, 55.5, 49.3 (q, *J* = 30.8 Hz), 42.2, 28.2.^19^F­{^1^H} NMR (376 MHz, CDCl_3_): δ (ppm) = −76.2. FT-IR (cm^–1^, neat, ATR), ν̃ = 3309, 2977, 2945, 1691, 1674, 1597, 1532, 1484, 1468, 1358, 1291, 1244, 1173, 1157, 1107, 1055, 996. HRMS (ESI+) calcd for C_16_H_20_F_3_NO_4_Na [M + Na]^+^: 370.1237; found, 370.1243.

#### 4-(3-((*Tert*-Butoxycarbonyl)­amino)-4,4,4-trifluorobutanoyl)­benzyl 3-Fluorobicyclo­[1.1.1] pentane-1-carboxylate **(13)**

Prepared according to the General Procedure from the corresponding styrene **1k** (62 mg, 0.25 mmol, 1.0 equiv) and **2a** (194 mg, 0.5 mmol, 2.0 equiv). After purification by flash column chromatography (hexane/EtOAc 6:1), the title compound **13** was obtained as a pale yellow semisolid (94 mg, 0.21 mmol, 82%). *R*_*f*_ = 0.18 (silica gel, *n*-hexane/EtOAc, 6:1 (v/v)); mp 35–37 °C. ^1^H NMR (500 MHz, CDCl_3_): δ (ppm) = 7.96 (d, *J* = 8.3 Hz, 2H), 7.46 (d, *J* = 8.2 Hz, 2H), 5.29–5.27 (m, 1H), 5.22 (s, 2H), 4.96–4.88 (m, 1H), 3.36–3.28 (m, 2H), 2.42 (d, *J* = 3.3 Hz, 6H), 1.46 (s, 9H). ^13^C­{^1^H} NMR (126 MHz, CDCl_3_): δ (ppm) = 194.6, 168.6 (d, *J* = 37.2 Hz), 154.6, 141.5, 136.0, 128.5, 128.0, 125.2 (q, *J* = 281.8 Hz), 80.7, 76.1, 73.5, 65.8, 55.6 (d, *J* = 21.6 Hz), 49.1 (q, *J* = 31.7 Hz), 36.9, 28.2.^19^F­{^1^H} NMR (376 MHz, CDCl_3_): δ (ppm) = −149.8, −75.8. FT-IR (cm^–1^, neat, ATR), ν̃ = 3351, 2981, 2931, 1697, 1605, 1520, 1413, 1369, 1323, 1249, 1150, 1051, 1016, 914. HRMS (ESI+) calcd for C_22_H_26_F_4_NO_5_ [M + H]^+^: 460.1742; found, 460.1735.

#### 4-(3-((*Tert*-Butoxycarbonyl)­amino)-4,4,4-trifluorobutanoyl)­benzyl (2*S*)-2-(4-Isobutylphenyl)­propanoate **(14)**

Prepared according to the General Procedure from the corresponding styrene **1m** (81 mg, 0.25 mmol, 1.0 equiv) and **2a** (194 mg, 0.5 mmol, 2.0 equiv). After purification by flash column chromatography (hexane/EtOAc 8:1), the title compound **15** was obtained as a pale yellow semisolid (76 mg, 0.14 mmol, 57%). *R*_*f*_ = 0.33 (silica gel, *n*-hexane/EtOAc, 6:1 (v/v); mp 35–37 °C. ^1^H NMR (500 MHz, CDCl_3_): δ (ppm) = 7.85 (d, *J* = 8.5 Hz, 2H), 7.28 (d, *J* = 8.7 Hz, 2H), 7.20 (d, *J* = 8.2 Hz, 2H), 7.10 (d, *J* = 8.3 Hz, 2H), 5.21–5.10 (m, 3H), 4.97–4.84 (m, 1H), 3.78 (q, *J* = 7.0 Hz, 1H), 3.32–3.27 (m, 2H), 2.46 (d, *J* = 7.1 Hz, 2H), 1.85 (sept, *J* = 6.8 Hz, 1H), 1.52 (d, *J* = 7.2, 3H), 1.44 (s, 9H), 0.90 (d, *J* = 6.6 Hz, 6H). ^13^C­{^1^H} NMR (126 MHz, CDCl_3_): δ (ppm) = 194.6, 174.3, 154.6, 142.3, 140.8, 137.4, 135.6, 129.4, 128.3, 127.6, 127.3, 125.2 (q, *J* = 282.2 Hz), 80.7, 65.3, 49.1 (q, *J* = 34.5 Hz), 45.1, 45.0, 36.8, 30.2, 28.2, 22.4, 18.3.^19^F­{^1^H} NMR (376 MHz, CDCl_3_): δ (ppm) = −75.7. FT-IR (cm^–1^, neat, ATR), ν̃ = 3366, 2957, 2928, 2870, 1735, 1704, 1687, 1524, 1456, 1417, 1367, 1302, 1247, 1152, 1051, 1020, 991. HRMS (ESI+) calcd for C_29_H_36_F_3_NO_5_Na [M + Na]^+^: 558.2438; found, 558.2448.

#### *Tert*-Butyl (1,1,1-Trifluoro-4-oxo-4-(4-((((3a*R*,5*R*,5a*S*,8a*S*,8b*R*)-2,2,7,7-tetramethyl tetrahydro-5*H*-bis­([1,3]­dioxolo)­[4,5-*b*:4′,5′-*d*]­pyran-5-yl)­methoxy)­methyl)­phenyl)­butan-2-yl)­carbamate **(15)**

Prepared according to the General Procedure from the corresponding styrene **1l** (94 mg, 0.25 mmol, 1.0 equiv) and **2a** (194 mg, 0.5 mmol, 2.0 equiv). After purification by flash column chromatography (hexane/EtOAc 10:1), the title compound **14** was obtained as a pale yellow solid (111 mg, 0.19 mmol, 76%). *R*_*f*_ = 0.10 (silica gel, *n*-hexane/EtOAc, 6:1 (v/v)); mp > 230 °C. ^1^H NMR (500 MHz, CDCl_3_): δ (ppm) = 7.92 (d, *J* = 8.4 Hz, 2H), 7.48 (d, *J* = 8.2 Hz, 2H), 5.57 (d, *J* = 5.0 Hz, 1H), 5.28 (d, *J* = 13.2 Hz, 1H), 4.97–4.88 (m, 1H), 4.72 (d, *J* = 9.0 Hz, 1H), 4.64 (d, *J* = 13.5 Hz, 2H), 4.35 (dd, *J* = 5.0, 2.4 Hz, 1H), 4.29 (d, *J* = 10.0 Hz, 1H), 4.06 (t, *J* = 5.2 Hz, 1H), 3.76–3.66 (m, 2H), 3.40–3.29 (m, 2H), 1.57 (s, 3H), 1.46 (bs, 12H), 1.36 (bs, 6H). ^13^C­{^1^H} NMR (126 MHz, CDCl_3_): δ (ppm) = 194.8, 154.7, 146.9, 135.3, 128.6, 127.5, 125.2 (q, *J* = 281.8 Hz), 109.3, 108.6, 96.3, 80.7, 72.5, 71.2, 70.7, 70.6, 69.5, 67.0, 49.1 (q, *J* = 31.3 Hz), 36.8, 34.1, 29.7, 28.2, 26.1, 26.0, 24.9, 24.5, 22.3, 14.1.^19^F­{^1^H} NMR (376 MHz, CDCl_3_): δ (ppm) = −75.7. FT-IR (cm^–1^, neat, ATR), ν̃ = 3330, 2986, 2932, 1697, 1369, 1284, 1255, 1211, 1098, 1017. HRMS (ESI+) calcd for C_28_H_38_F_3_NO_9_Na [M + Na]^+^: 612.2391; found, 612.2390.

#### *Tert*-Butyl (1,1,1,2,2-Pentafluoro-5-(2-methoxyphenyl)-5-oxopentan-3-yl)­carbamate **(16)**

Prepared according to the General Procedure from the corresponding styrene **1j** (33 mg, 0.25 mmol, 1.0 equiv) and **2b** (218 mg, 0.5 mmol, 2.0 equiv). After purification by flash column chromatography (hexane/EtOAc 10:1), the title compound **16** was obtained as a white solid (64 mg, 0.16 mmol, 65%). *R*_*f*_ = 0.18 (silica gel, *n*-hexane/EtOAc, 10:1 (v/v)); mp 93–95 °C. ^1^H NMR (600 MHz, CDCl_3_): δ (ppm) = 7.73 (dd, *J* = 7.7, 1.8 Hz, 1H), 7.53–7.47 (m, 1H), 7.02–6.98 (m, 2H), 5.12–4.84 (m, 2H), 3.93 (s, 3H), 3.59–3.35 (m, 1H), 3.22 (dd, *J* = 16.3, 8.8 Hz, 1H), 1.38 (s, 9H). ^13^C­{^1^H} NMR (151 MHz, CDCl_3_): δ (ppm) = 197.2, 158.8, 154.5, 134.7, 134.6, 131.1, 131.0, 126.9, 121.1, 120.3–117.8 (m), 80.6, 55.6 (q, *J* = 34.7 Hz), 42.1, 28.3 (q, *J* = 20.4 Hz, 3C). ^19^F­{^1^H} NMR (282 MHz, CDCl_3_): δ (ppm) = −82.3 (3F), −120.0 (d, *J* = 273.3 Hz, 1F), −126.1 (d, *J* = 273.2 Hz, 1F). FT-IR (cm^–1^, neat, ATR), ν̃ = 3315, 2979, 2928, 1695, 1670, 1540, 1485, 1368, 1287, 1216, 1158, 1083, 1054, 1011. HRMS (ESI+) calcd for C_17_H_20_F_5_NO_4_Na [M + Na]^+^: 420.1210; found, 420.1215.

#### *Tert*-Butyl (5-(4-(*Tert*-butyl)­phenyl)-1,1,1,2,2-pentafluoro-5-oxopentan-3-yl)­carbamate **(17)**

Prepared according to the General Procedure from the corresponding styrene **1a** (40 mg, 0.25 mmol, 1.0 equiv) and **2b** (218 mg, 0.5 mmol, 2.0 equiv). After purification by flash column chromatography (hexane/EtOAc 10:1), the title compound **17** was obtained as a white solid (74 mg, 0.18 mmol, 70%). *R*_*f*_ = 0.25 (silica gel, *n*-hexane/EtOAc, 10:1 (v/v)); mp 110–112 °C. ^1^H NMR (600 MHz, CDCl_3_): δ (ppm) = 7.88 (d, *J* = 8.5 Hz, 2H), 7.50 (d, *J* = 8.6 Hz, 2H), 5.25 (d, *J* = 10.1 Hz, 1H), 5.10–5.04 (m, 1H), 3.40–3.29 (m, 2H), 1.44 (s, 9H), 1.34 (s, 9H). ^13^C­{^1^H} NMR (151 MHz, CDCl_3_): δ (ppm) = 194.9, 157.7, 154.3, 133.6, 128.1, 125.8, 120.1–117.7 (m), 80.7, 47.7–46.6 (m), 36.3, 35.2, 31.0, 28.1.^19^F­{^1^H} NMR (376 MHz, CDCl_3_): δ (ppm) = −82.3 (3F), −118.8 (d, *J* = 273.3 Hz, 1F), −125.7 (d, *J* = 273.3 Hz, 1F). FT-IR (cm^–1^, neat, ATR), ν̃ = 3327, 2964, 2928, 2872, 1692, 1538, 1368, 1286, 1256, 1214, 1169, 1127, 1108, 1082, 1023, 1010, 973. HRMS (ESI+) calcd for C_20_H_26_F_5_NO_3_Na [M + Na]^+^: 446.1725; found, 446.1734.

#### *Tert*-Butyl (1,1,1,2,2-Pentafluoro-5-(4-methoxyphenyl)-5-oxopentan-3-yl)­carbamate **(18)**

Prepared according to the General Procedure from the corresponding styrene **1c** (33 mg, 0.25 mmol, 1.0 equiv) and **2b** (218 mg, 0.5 mmol, 2.0 equiv). After purification by flash column chromatography (hexane/EtOAc 10:1), the title compound **18** was obtained as a white solid (45 mg, 0.11 mmol, 45%). *R*_*f*_ = 0.18 (silica gel, *n*-hexane/EtOAc, 10:1 (v/v)); mp 115–117 °C. ^1^H NMR (400 MHz, CDCl_3_): δ (ppm) = 7.92 (d, *J* = 8.8 Hz, 2H), 6.95 (d, *J* = 8.8 Hz, 2H), 5.40–4.88 (m, 2H), 3.88 (s, 3H), 3.32 (d, *J* = 5.7 Hz, 2H), 1.42 (s, 9H). ^13^C­{^1^H} NMR (126 MHz, CDCl_3_): δ (ppm) = 193.9, 164.2, 154.5, 130.6, 129.4, 121.1–116.2 (m), 114.2, 80.8, 55.7, 50.0–44.4 (m), 36.2, 28.3.^19^F­{^1^H} NMR (376 MHz, CDCl_3_): δ (ppm) = −82.3 (3F), −118.8 (d, *J* = 273.2 Hz, 1F), −125.7 (d, *J* = 273.0 Hz, 1F). FT-IR (cm^–1^, neat, ATR),ν̃ = 3325, 3006, 2972, 2930, 2847, 1694, 1678, 1603, 1535, 1285, 1259, 1245, 1208, 1191, 1164, 1129, 1053, 1018, 1004. HRMS (ESI+) calcd for C_17_H_20_F_5_NO_4_Na [M + Na]^+^: 420.1210; found, 420.1215.

#### *Tert*-Butyl (1,1,1,2,2-Pentafluoro-5-(4-fluorophenyl)-5-oxopentan-3-yl)­carbamate **(19)**

Prepared according to the General Procedure from the corresponding styrene **1e** (30 mg, 0.25 mmol, 1.0 equiv) and **2b** (218 mg, 0.5 mmol, 2.0 equiv). After purification by flash column chromatography (hexane/EtOAc 9:1), the title compound **19** was obtained as a white solid (69 mg, 0.18 mmol, 72%). *R*_*f*_ = 0.20 (silica gel, *n*-hexane/EtOAc, 9:1 (v/v)); mp 132–134 °C. ^1^H NMR (300 MHz, CDCl_3_): δ (ppm) = 8.17–7.74 (m, 2H), 7.16 (t, *J* = 8.6 Hz, 2H), 5.24–4.94 (m, 2H), 3.35 (d, *J* = 5.6 Hz, 2H), 1.42 (s, 9H). ^13^C­{^1^H} NMR (126 MHz, CDCl_3_): δ (ppm) = 193.7, 166.3 (d, *J* = 256.2 Hz), 154.4, 132.8 (d, *J* = 3.0 Hz), 128.6 (d, *J* = 9.5 Hz), 121.2–117.2 (m), 116.2 (d, *J* = 22.0 Hz), 81.0, 48.1–46.6 (m), 36.6, 28.3.^19^F­{^1^H} NMR (282 MHz, CDCl_3_): δ (ppm) = −82.2 (3F), −103.8, −118.7 (d, *J* = 273.5 Hz, 1F), −125.9 (d, *J* = 273.5 Hz, 1F). FT-IR (cm^–1^, neat, ATR), ν̃ = 3313, 2975, 2923, 1687, 1598, 1540, 1369, 1283, 1239, 1214, 1188, 1171, 1154, 1053, 1027, 1011. HRMS (ESI+) calcd for C_16_H_17_F_6_NO_3_Na [M + Na]^+^: 408.1005; found, 408.1015.

#### 4-(3-((*Tert*-Butoxycarbonyl)­amino)-4,4,5,5,5-pentafluoropentanoyl)­benzyl 3-fluorobicyclo [1.1.1]­pentane-1-carboxylate **(20)**

Prepared according to the General Procedure from the corresponding styrene **1k** (61 mg, 0.25 mmol, 1.0 equiv) and **2b** (218 mg, 0.5 mmol, 2.0 equiv). After purification by flash column chromatography (hexane/EtOAc 7:3), the title compound **20** was obtained as a white solid (51 mg, 0.10 mmol, 38%). *R*_*f*_ = 0.67 (silica gel, *n*-hexane/EtOAc, 7:3 (v/v)); mp 86–88 °C. ^1^H NMR (400 MHz, CDCl_3_): δ (ppm) = 7.94 (d, *J* = 8.0 Hz, 2H), 7.43 (d, *J* = 8.0 Hz, 2H), 5.65–4.82 (m, 4H), 3.37 (d, *J* = 5.7 Hz, 2H), 2.40 (d, *J* = 2.4 Hz, 6H), 1.42 (s, 9H). ^13^C­{^1^H} NMR (126 MHz, CDCl_3_): δ (ppm) = 194.8, 168.7 (d, *J* = 37.2 Hz), 154.6, 141.6, 136.1, 128.6, 128.2, 122.7–111.7 (m), 80.9, 74.9 (d, *J* = 316.3 Hz), 65.9, 55.7 (d, *J* = 22.0 Hz), 47.9–46.3 (m), 36.8, 28.3.^19^F­{^1^H} NMR (376 MHz, CDCl_3_): δ (ppm) = −82.2 (3F), −118.7 (d, *J* = 273.3 Hz, 1F), −125.9 (d, *J* = 273.7 Hz, 1F), −149.7 (1F). FT-IR (cm^–1^, neat, ATR), ν̃ = 3315, 2981, 2929, 1735, 1694, 1537, 1523, 1370, 1334, 1286, 1248, 1213, 1168, 1044, 1012. HRMS (ESI+) calcd for C_23_H_25_F_6_NO_5_Na [M + Na]^+^: 532.1535; found, 532.1536.

#### *Tert*-Butyl (1,1-Difluoro-4-(2-methoxyphenyl)-4-oxobutan-2-yl)­carbamate **(21)**

Prepared according to the General Procedure from the corresponding styrene **1j** (33.5 mg, 0.25 mmol, 1.0 equiv) and **2c** (184 mg, 0.5 mmol, 2.0 equiv). After purification by flash column chromatography (hexane/EtOAc 4:1), the title compound **21** was obtained as a colorless oil (50.2 mg, 0.15 mmol, 61% yield). The product was obtained as a mixture of rotamers. *R*_*f*_ = 0.43 (silica gel, *n*-hexane/EtOAc 4:1 (v/v)). ^1^H NMR (500 MHz, CDCl_3_): δ (ppm): 7.88 (dd, *J* = 7.8, 1.9 Hz, 0.47 × 1, 1H), 7.81 (dd, *J* = 7.7, 1.9 Hz, 0.53 × 1, 1H), 7.54–7.46 (m, 1H), 7.07–7.01 (m, 1H), 6.98 (dd, *J* = 11.2, 8.5 Hz, 1H), 5.98 (tt, *J* = 56.2, 4.3 Hz, 0.60 × 1, 1H), 5.92 (tt, *J* = 56.2, 4.2 Hz, 0.40 × 1, 1H), 4.70 (s, 0.90 × 1, 2H), 4.61 (s, 1.10 × 1, 2H), 3.93 (s, 1.60 × 1, 3H), 3.92 (s, 1.40 × 1, 3H), 3.69–3.53 (m, 2H), 1.49 (s, 4.27 × 1, 9H), 1.37 (s, 4.64 × 1, 9H). ^13^C­{^1^H} NMR (126 MHz, CDCl_3_): δ (ppm): 196.1, 195.8, 159.6, 159.2, 156.0, 155.4, 134.7, 134.5, 131.1, 130.8, 125.7, 125.4, 121.1, 121.0, 115.1 (t, *J* = 242.5 Hz), 115.0 (t, *J* = 241.9 Hz), 111.7, 111.6, 81.1, 80.9, 59.8, 59.3, 55.7, 55.6, 51.2 (t, *J* = 27.3 Hz), 51.1 (t, *J* = 27.2 Hz), 28.4, 28.2.^19^F­{^1^H} NMR (377 MHz, CDCl_3_): δ (ppm): −121.07 (0.90 × 2, 2F), −121.17 (1.10 × 2, 2F). FT-IR (cm^–1^, neat, ATR), ν̃ = 3343, 2976, 2929, 1697, 1599, 1455, 1286, 1203, 1051, 757. HRMS (ESI+) *m*/*z*: [M + Na]^+^ Calcd. for C_16_H_21_F_2_NO_4_Na 352.1331; found, 352.1331.

#### *Tert*-Butyl (4-(4-(*Tert*-Butyl)­phenyl)-1,1-difluoro-4-oxobutan-2-yl)­carbamate **(22)**

Prepared according to the General Procedure from the corresponding styrene **1a** (40.1 mg, 0.25 mmol) and **2c** (184 mg, 0.5 mmol, 2.0 equiv). After purification by flash column chromatography (hexane/EtOAc 9:1), the title compound **22** was obtained as a colorless oil (49.8 mg, 0.14 mmol, 56% yield). The product was obtained as a mixture of rotamers. *R*_*f*_ = 0.5, (silica gel, *n*-hexane/EtOAc 4:1 (v/v)). ^1^H NMR (500 MHz, CDCl_3_): δ (ppm): 7.86 (dd, *J* = 10.1, 8.2 Hz, 2H), 7.49 (dd, *J* = 12.5, 8.3 Hz, 2H), 5.98 (tt, *J* = 56.1, 4.3 Hz, 0.50 × 1, 1H), 5.92 (tt, *J* = 56.2, 4.2 Hz, 0.50 × 1, 1H), 4.76 (s, 1H), 4.67 (s, 1H), 3.64 (qd, *J* = 14.4, 4.4 Hz, 2H), 1.50 (s, 4.50 × 1, 9H), 1.37 (s, 4.50 × 1, 9H), 1.35 (s, 4.50 × 1, 9H), 1.33 (s, 4.50 × 1, 9H). ^13^C­{^1^H} NMR (126 MHz, CDCl_3_): δ (ppm): 194.2, 193.9, 157.7, 155.8, 155.3, 132.6 128.0, 127.8, 126.0, 125.8, 115.0 (t, *J* = 242.0 Hz), 115.0 (t, *J* = 242.7 Hz), 81.5, 81.2, 55.2, 54.4, 51.0 (t, *J* = 27.1 Hz), 50.9 (t, *J* = 27.1 Hz), 35.3, 35.3, 31.2, 28.4, 28.2.^19^F­{^1^H} NMR (377 MHz, CDCl_3_): δ (ppm): −121.07 (s, 1.00 × 2, 2H), −121.13 (s, 1.00 × 2, 2H). FT-IR (cm^–1^, neat, ATR), ν̃ = 2966, 1695, 1605, 1454, 1394, 1367, 1234, 1165, 1119, 1054, 991, 894, 855, 775. HRMS (ESI+) *m*/*z*: [M + Na]^+^ Calcd. for C_19_H_27_F_2_NO_3_Na 378.1851; found, 378.1855.

#### *Tert*-Butyl (4-(4-Fluorophenyl)-1,1-difluoro-4-oxobutan-2-yl)­carbamate **(23)**

Prepared according to the General Procedure from the corresponding styrene **1e** (30.5 mg, 0.25 mmol) and **2c** (184 mg, 0.5 mmol, 2.0 equiv). After purification by column chromatography (hexane/EtOAc, 9:1), the title compound **23** was obtained as a colorless oil (46.8 mg, 0.15 mmol, 59% yield). The product was obtained as a mixture of rotamers. *R*_*f*_ = 0.65, (silica gel, *n*-hexane/EtOAc 4:1 (v/v)). ^1^H NMR (500 MHz, CDCl_3_): δ (ppm): 7.96 (td, *J* = 9.1, 5.5 Hz, 2H), 7.16 (dt, *J* = 13.5, 8.6 Hz, 2H), 5.97 (tt, *J* = 56.0, 4.3 Hz, 0.50 × 1, 1H), 5.92 (tt, *J* = 56.0, 4.2 Hz, 0.50 × 1, 1H), 4.74 (s, 1H), 4.65 (s, 1H), 3.72–3.56 (m, 2H), 1.50 (s, 4.60 × 1, 9H), 1.36 (s, 4.40 × 1, 9H). ^13^C­{^1^H} NMR (126 MHz, CDCl_3_): δ (ppm): 193.0, 192.8, 166.2 (d, *J* = 255.7 Hz), 155.6, 155.3, 131.6, 131.5, 130.6 (dd, *J* = 28.3, 9.4 Hz), 116.2 (dd, *J* = 21.9, 15.8 Hz), 114.9 (t, *J* = 242.3 Hz), 81.7, 81.4, 55.1, 54.4, 51.0 (t, *J* = 27.1 Hz), 50.8 (t, *J* = 26.9 Hz), 28.4, 28.2.^19^F­{^1^H} NMR (377 MHz, CDCl_3_): δ (ppm): −103.78 (s, 0.50 × 1, 1H), −103.83 (s, 0.50 × 1, 1H), −121.11 (s, 1.00 × 2, 2H), −121–16 (s, 1.00 × 2, 2H). FT-IR (cm^–1^, neat, ATR), ν̃ = 2979, 2934, 1694, 1599, 1509, 1454, 1395, 1368, 1226, 1157, 1119, 1053, 991,894, 836. HRMS (ESI+) *m*/*z*: [M + Na]^+^ Calcd. for C_15_H_18_F_3_NO_3_Na 340.1131; found, 340.1140.

#### *Tert*-Butyl (1,1-Difluoro-4-oxo-4-(4-((((3a*R*,5*R*,5a*S*,8a*S*,8b*R*)-2,2,7,7-tetramethyltetrahydro-5*H*-bis­([1,3]­dioxolo)­[4,5-*b*:4′,5′-*d*]­pyran-5-yl)­methoxy)­methyl)­phenyl)­butan-2-yl)­carbamate **(24)**

Compound **24** was prepared according to the general procedure from 2-vinylnaphthalene **1l** (94.1 mg, 0.25 mmol) and **2c** (184 mg, 0.5 mmol, 2.0 equiv). After purification by column chromatography (hexane/EtOAc, 9:1), the title compound **24** was obtained as a colorless oil (81.2 mg, 0.14 mmol, 57% yield). *R*_*f*_ = 0.5, (silica gel, *n*-hexane/EtOAc 4:1­(v/v)). The product was obtained as a mixture of rotamers. ^1^H NMR (600 MHz, CDCl_3_): δ (ppm): 7.89 (dd, *J* = 12.0, 8.0 Hz, 2H), 7.46 (dd, *J* = 15.4, 7.9 Hz, 2H), 5.98 (tt, *J* = 56.0, 4.3 Hz, 0.60 × 1, 1H), 5.92 (tt, *J* = 56.0, 4.1 Hz, 0.40 × 1, 1H), 5.55 (d, *J* = 5.1 Hz, 1H), 4.76 (s, 1H), 4.71–4.59 (m, 4H), 4.33 (dt, *J* = 5.4, 2.8 Hz, 1H), 4.27 (ddd, *J* = 8.1, 3.3, 1.9 Hz, 1H), 4.07–4.00 (m, 1H), 3.75–3.60 (m, 4H), 1.55 (s, 3.60 × 1, 6H), 1.50 (s, 4.80 × 1, 12H), 1.44 (s, 3.60 × 1, 6H), 1.36 (s, 3.60 × 1, 9H), 1.34 (s, 5.40 × 1, 9H). ^13^C­{^1^H} NMR (126 MHz, CDCl_3_): δ (ppm): 194.2, 194.0, 168.7, 155.7, 155.3, 144.8, 144.8, 134.3, 134.2, 130.7, 128.1, 127.9, 127.7, 127.6, 125.7 (q, *J* = 3.4 Hz), 116.9, 115.0 (t, *J* = 242.3 Hz), 109.5, 108.8, 96.5, 81.6, 81.3, 72.7, 71.4, 70.8, 70.7, 69.6, 69.6, 67.2, 55.3, 54.5, 51.0 (t, *J* = 27.2 Hz), 50.9 (t, *J* = 26.8 Hz), 28.4, 28.2, 26.2, 26.1, 25.1, 24.6.^19^F­{^1^H} NMR (377 MHz, CDCl_3_): δ (ppm): −121.09 (1.00 × 2, 2F), −121.16 (1.20 × 2, 2F). FT-IR (cm^–1^, neat, ATR), ν̃ = 3350, 2982, 2933, 1693, 1514, 1370, 1253, 1165, 1062, 1002, 860, 701. HRMS (ESI+) *m*/*z*: [M + Na]^+^ Calcd. for C_28_H_39_F_2_NO_9_Na 594.2485; found, 594.2496.

#### *Tert*-Butyl (4-(4-(*Tert*-butyl)­phenyl)-1-fluoro-4-oxobutan-2-yl)­carbamate **(25)**

Prepared according to the General Procedure from the corresponding styrene **1a** (40 mg, 0.25 mmol, 1.0 equiv) and **2d** (175 mg, 0.5 mmol, 2.0 equiv). After purification by flash column chromatography (hexane/EtOAc 9:1), the title compound **25** was obtained as a pale yellow oil (52 mg, 0.16 mmol, 62%). *R*_*f*_ = 0.30 (silica gel, *n*-hexane/EtOAc, 9:1 (v/v)). The product was obtained as a mixture of rotamers. ^1^H NMR (300 MHz, CDCl_3_): δ (ppm) = 7.94–7.82 (m, 2H), 7.54–7.44 (m, 2H), 4.77 (s, 1H), 4.72–4.60 (m, 2H), 4.50 (dt, *J* = 13.6, 4.8 Hz, 1H), 3.71–3.53 (m, 2H), 1.49 (s, 4.50 × 1, 9H), 1.37 (s, 4.50 × 1, 9H), 1.36–1.32 (m, 9H). ^13^C­{^1^H} NMR (151 MHz, CDCl_3_): δ (ppm) = 194.7, 194.4, 157.4, 155.7, 132.8, 132.7, 128.2, 128.0, 127.8, 125.9, 125.8, 125.7, 84.0 (*J* = 166.4 Hz), 83.3 (*J* = 167.9 Hz), 80.7, 80.6, 55.3, 54.5, 49.0 (d, *J* = 19.4 Hz), 48.9 (d, *J* = 20.7 Hz), 35.3, 31.2, 28.5, 28.3.^19^F­{^1^H} NMR (282 MHz, CDCl_3_): δ (ppm) = −221.5, −222.1. FT-IR (cm^–1^, neat, ATR), ν̃ = 2961, 2917, 2849, 1701, 1605, 1460, 1393, 1365, 1232, 1167, 1108, 1025, 989. HRMS (ESI+) calcd for C_19_H_28_FNO_3_Na [M + Na]^+^: 360.1945; found, 360.1955.

#### *Tert*-Butyl (1-Fluoro-4-(4-fluorophenyl)-4-oxobutan-2-yl)­carbamate **(26)**

Prepared according to the General Procedure from the corresponding styrene **1e** (31 mg, 0.25 mmol, 1.0 equiv) and **2d** (175 mg, 0.5 mmol, 2.0 equiv). After purification by flash column chromatography (hexane/EtOAc 4:1), the title compound **26** was obtained as a pale yellow oil (36.7 mg, 0.13 mmol, 49%). *R*_*f*_ = 0.42 (silica gel, *n*-hexane/EtOAc, 4:1 (v/v)). The product was obtained as a mixture of rotamers. ^1^H NMR (500 MHz, CDCl_3_): δ (ppm) = 8.01–7.93 (m, 2H), 7.19–7.13 (m, 2H), 4.76 (s, 1H), 4.70–4.49 (m, 3H), 3.63 (tt, *J* = 26.6, 4.8 Hz, 2H), 1.49 (s, 4.50 × 1, 9H), 1.37 (s, 4.50 × 1, 9H). ^13^C­{^1^H} NMR (126 MHz, CDCl_3_): δ (ppm) = 193.5, 193.3, 166.1 (d, *J* = 255.7 Hz), 166.1 (d, *J* = 255.0 Hz), 155.6, 155.5, 130.7 (d, *J* = 9.4 Hz), 130.5 (d, *J* = 9.4 Hz), 116.2 (d, *J* = 22.2 Hz), 116.1 (d, *J* = 21.7 Hz), 84.1 (*J* = 166.6 Hz), 83.4 (*J* = 167.6 Hz), 80.9, 80.7, 55.3, 55.2, 54.5, 54.5, 49.1, 48.9, 28.5, 28.3.^19^F­{^1^H} NMR (377 MHz, CDCl_3_): δ (ppm) = −104.2 (0.50 × 1, 1F), −104.2 (0.50 × 1, 1F), −221.5 (0.50 × 1, 1F), −222.1 (0.50 × 1, 1F). FT-IR (cm^–1^, neat, ATR), ν̃ = 3376, 2984, 2932, 1700, 1603, 1512, 1466, 1300, 1231, 1156, 1042, 899, 745. HRMS (ESI+) calcd for C_15_H_19_F_2_NO_3_Na [M + Na]^+^: 322.1225; found, 322.1228.

#### *Tert*-Butyl (1-Fluoro-4-(4-methoxyphenyl)-4-oxobutan-2-yl)­carbamate **(27)**

Prepared according to the General Procedure from the corresponding styrene **1c** (34 mg, 0.25 mmol, 1.0 equiv) and **2d** (175 mg, 0.5 mmol, 2.0 equiv). After purification by flash column chromatography (hexane/EtOAc 4:1), the title compound **27** was obtained as a pale yellow oil (21.8 mg, 0.07 mmol, 28%). *R*_*f*_ = 0.28 (silica gel, *n*-hexane/EtOAc, 4:1 (v/v)). The product was obtained as a mixture of rotamers. ^1^H NMR (400 MHz, CDCl_3_): δ (ppm) = 7.98–7.84 (m, 2H), 6.94 (t, *J* = 9.3 Hz, 2H), 4.75 (s, 1H), 4.69–4.47 (m, 3H), 3.91–3.84 (m, 3H), 3.70–3.54 (m, 2H), 1.49 (s, 4.50 × 1, 9H), 1.36 (s, 4.50 × 1, 9H). ^13^C­{^1^H} NMR (151 MHz, CDCl_3_): δ (ppm) = 193.5, 193.3, 163.9, 155.7, 130.6, 130.3, 130.3, 130.1, 128.4, 114.2, 114.1, 114.0, 84.0 (*J* = 166.9 Hz), 83.3 (*J* = 167.7 Hz), 80.7, 80.5, 55.7, 55.6, 55.0, 54.3, 49.0 (d, *J* = 19.9 Hz), 49.0 (*J* = 20.3 Hz), 28.5, 28.3.^19^F­{^1^H} NMR (377 MHz, CDCl_3_): δ (ppm) = −221.6 (0.50 × 1, 1F), −222.2 (0.50 × 1, 1F). FT-IR (cm^–1^, neat, ATR), ν̃ = 3366, 2974, 2929, 2849, 1686, 1600, 1512, 1456, 1366, 1231, 1166, 1066, 814. HRMS (ESI+) calcd for C_16_H_22_FNO_4_Na [M + Na]^+^: 334.1425; found, 334.1425.

## Supplementary Material



## Data Availability

The data underlying this study are available in the published article and its Supporting Information.

## Notes

Preparation of starting materials, general procedures, derivatization reactions, mechanistic studies and NMR spectra (PDF)

## †

Dedicated to Prof. Iluminada Gallardo (UAB) on the occasion of her retirement.
