# Supplementary material for: Organophotoredox-Driven Three-Component Synthesis of β‑Trifluoromethyl β‑Amino Ketones
Source: J Org Chem. 2025 Jan 30;90(6):2500–9. doi: 10.1021/acs.joc.4c03142 (PMC12136134; doi:10.1021/acs.joc.4c03142)
Supplement: Supplementary file 1 [file jo4c03142_si_001.pdf]

Supporting Information

Organophotoredox-driven Three-Component Synthesis of β-Trifluoromethyl β-Amino Ketones

Marta Gil-Ordóñez, Albert Gallego-Gamo,‡ Pau Sarró,‡ Roser Pleixats, Carolina Gimbert-Suriñach,* Adelina Vallribera* and Albert Granados*

Departament de Química and Centro de Innovación en Química Avanzada (ORFEO-CINQA), Universitat Autònoma de Barcelona, Cerdanyola del Vallès, 08193 Barcelona, Spain

‡These authors contributed equally.

*To whom correspondence should be addressed.

carolina.gimbert@uab.es adelina.vallribera@uab.es albert.granados@uab.es

TABLE OF CONTENT

1. General Considerations S2

2. List of Used Styrenes and Redox Active Species S2

3. Synthesis of Starting Materials S3

4. Synthesis of β-Trifluoromethyl β-amino Ketones. Reaction Workflow and Compound Characterization for Derivatization Reactions S8

5. Large-Scale Synthesis of Compound **3** S11

6. Unproductive Substrates S12

7. Mechanistic Investigation S12

8. NMR Data S19

1. General Considerations

1.1 Chemicals

Deuterated NMR solvents were purchased from Eurisotop. Dry solvents were obtained from Aldrich or Fisher and used as received. Bulk DCM, EtOAc and hexane were purchased from VWR. Chemicals were purchased from Fluorochem and Merck and used as received unless specified. Photoredox-catalyzed reactions were performed using 8 mL Chemglass vials (2-dram, 17 x 60 mm, 15-425 Green Open Top Cap, TFE Septa).

2. List of Used Styrenes and Redox Active Species

Styrenes **1k-1m** were prepared according to modified reported methods,[1] and the rest were purchased from Aldrich, Fisher or Fluorochem.

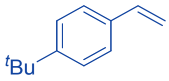

1a

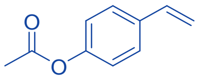

1b

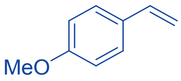

1c

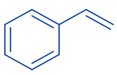

1d

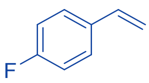

1e

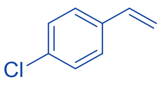

1f

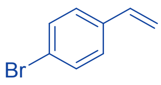

1g

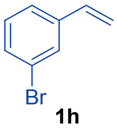

1h

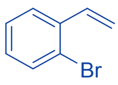

1i

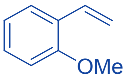

1j

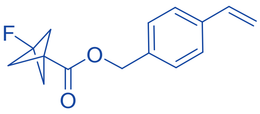

1k

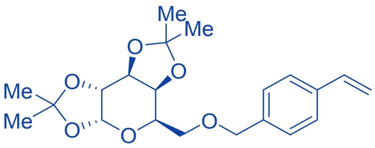

1l

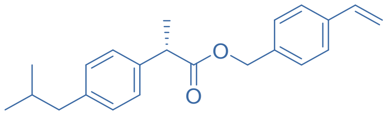

1m

Redox active species **2a-2e** were prepared according to modified reported procedures.[2]

---

[1] a) Granados, A., Dhungana, R. K., Sharique, M. and Molander, G. *Org. Lett.* **2022**, *24*, 4750–4755. b) Marcum, J. S., Cervarich, T. N., Manan, R. S., Roberts, C. C. and Meek, S. J. *ACS Catal.* **2019**, *9*, 5881–5889.

[2] a) Liu, Y., Zhou, T., Xuan, L., Lin, Y., Li, F., Wang, H., Lyu, J., Yan, Q., Zhou, H., Wamg, W. and Chen. F-E. *Org. Lett.* **2023**, *25*, 8693-8699. b) Wang, J., Liu, Shuai., Huang, Y., Xu, X-H. and Qing, F-L. **2022**, *58*, 1346-1349.

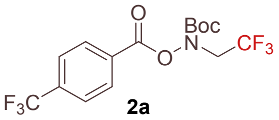

2a

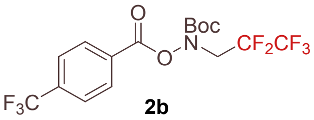

2b

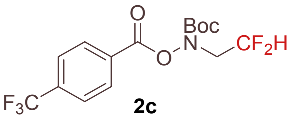

2c

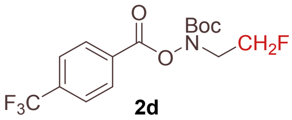

2d

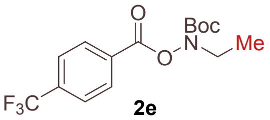

2e

3. Synthesis of Starting Materials

Synthesis of 4-Vinylbenzyl 3-Fluorobicyclo[1.1.1]pentane-1-carboxylate (1k)

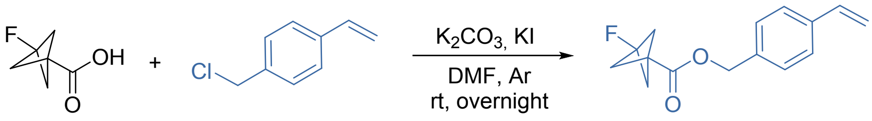

1k

In a Schlenk flask, 3-fluorobicyclo[1.1.1]pentane-1-carboxylic acid (195.2 mg, 1.5 mmol, 1 equiv) was dissolved in dry DMF (7.5 mL). Then, K_2CO_3 (317.9 mg, 2.3 mmol, 1.5 equiv) and KI (381.8 mg, 2.3 mmol, 1.5 equiv) were added and stirred. To the stirring suspension, 4-vinylbenzyl chloride (256.4 mg, 1.7 mmol, 1.1 equiv) was added, and the reaction was stirred overnight at room temperature. Upon completion, the mixture was diluted with 10 mL of AcOEt and 10 mL of water. The reaction mixture was then extracted and washed with brine (3 x 10 mL), dried over Na_2SO_4, filtered and concentrated under reduced pressure. After purification by flash column chromatography (DCM), the title compound **1k** was obtained as a colorless oil (332 mg, 1.35 mmol, 90%).

$\mathbf{R_f}$ = 0.48 (silica gel, *n*-hexane / EtOAc, 6:1 (v/v)). **^{1}H NMR** (500 MHz, $CDCl_3$), δ (ppm) = 7.44 (d, *J* = 8.3 Hz, 2H), 7.33 (d, *J* = 8.3 Hz, 2H), 6.76 (dd, *J* = 17.7, 10.9 Hz, 1H), 5.81 (d, *J* = 17.6 Hz, 1H), 5.31 (d, *J* = 10.8 Hz, 1H), 5.16 (s, 2H), 2.41 (s, 6H). **^{13}C{^{1}H} NMR** (126 MHz, $CDCl_3$), δ (ppm) = 168.8 (d, *J* = 36.8 Hz), 160.8, 137.8, 136.3, 135.1, 128.5, 126.5, 114.5, 76.2, 73.6, 66.6, 55.5 (*J* = 22.1 Hz). **^{19}F{^{1}H} NMR** (376 MHz, $CDCl_3$), δ (ppm) = -149.7. **FT-IR** (cm^{-1}, neat, ATR), ṽ = 3012, 2928, 1724, 1699, 1520, 1333, 1248, 1171, 1040, 809, 613. **HRMS (ESI+)** calcd for $C_{15}H_{15}FO_2Na$ $[M+Na]^+$: 269.0948, found 269.0954.

Synthesis of Tert-butyl (2,2,2-Trifluoroethyl)((4-(trifluoromethyl)benzoyl)oxy)carbamate (2a)

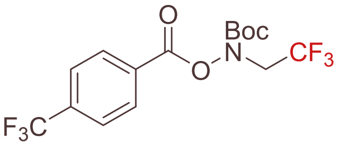

In a 250 mL round bottomed flask *tert*-butyl ((4-(trifluoromethyl)benzoyl)oxy)carbamate (3.05 g, 10 mmol, 1.0 equiv) is dissolved in 80 mL of dry DMF. Then, NaH (0.60 g, 60 % in mineral oil, 15 mmol, 1.6 equiv) was slowly added portion wise at 0ºC and the reaction was further stirred at 0ºC for 30 min. Subsequently, 2,2,2-trifluoroethyl trifluoromethanesulfonate (2.16 mL, 15 mmol, 1.5 equiv) was added dropwise at 0ºC. The reaction was allowed to proceed at room temperature for 2 h. After completion of the reaction, the mixture was quenched with H_2O and extracted with AcOEt (3 x 40 mL). The combined organic layers were further washed with brine and dried over anhydrous Na_2SO_4. The organic phase was concentrated under reduced pressure and purified by flash column chromatography through silica gel using hexane/EtOAc (10:1) to yield compound **2a** as white solid (3.25 g, 8.4 mmol, 84% yield). The NMR data agrees with those reported.[2]

^{1}H NMR (600 MHz, $CDCl_3$), δ (ppm) = 8.21 (d, *J* = 8.1 Hz, 2H), 7.78 (d, *J* = 8.2 Hz, 2H), 4.37 (s, 2H), 1.49 (s, 9H).

Synthesis of Tert-butyl (2,2,3,3,3-Pentafluoropropyl)((4-(trifluoromethyl)benzoyl) oxy)carbamate (2b)

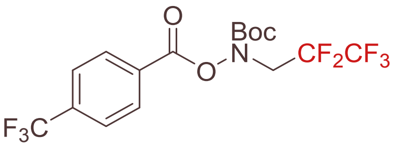

In a 250 mL round bottomed flask *tert*-butyl ((4-(trifluoromethyl)benzoyl)oxy)carbamate (3.05 g, 10 mmol, 1.0 equiv) is dissolved in 80 mL of dry DMF. Then, NaH (0.60 g, 60 % in mineral oil, 15 mmol, 1.6 equiv) was slowly added portion wise at 0ºC and the reaction was further stirred at 0ºC for 30 min. Subsequently, 2,2,3,3,3-pentafluoroethyl trifluoromethanesulfonate (2.49 mL, 15 mmol, 1.5 equiv) was added dropwise at 0ºC. The reaction was allowed to proceed at room temperature for 4 h. After completion of the reaction, the mixture was quenched with H_2O and extracted with AcOEt (3 x 40 mL). The combined organic layers were further washed with brine and dried over anhydrous Na_2SO_4. The organic phase was concentrated under reduced pressure and purified by flash column chromatography through silica gel using hexane/EtOAc (10:1) to yield compound **2b** as colorless oil (3.37 g, 7.7 mmol, 77% yield). The NMR data agrees with those reported.[2]

^{1}H NMR (600 MHz, $CDCl_3$), δ (ppm) = 8.21 (d, *J* = 8.1 Hz, 2H), 7.78 (d, *J* = 8.2 Hz, 2H), 4.37 (bs, 2H), 1.49 (s, 9H).

Synthesis of Tert-butyl (2,2-Difluoroethyl)((4-(trifluoromethyl)benzoyl)oxy)carbamate (2c)

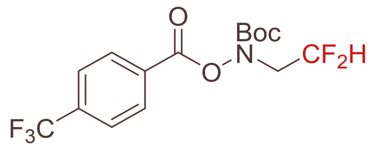

In a 100 mL round bottomed flask *tert*-butyl ((4-(trifluoromethyl)benzoyl)oxy)carbamate (0.90 g, 3.0 mmol, 1.0 equiv) is dissolved in 24 mL of dry DMF. Then, NaH (0.24 g, 60 % in mineral oil, 6.0 mmol, 2.0 equiv) was slowly added portion wise at 0ºC and the reaction was further stirred at 0ºC for 30 min. Subsequently, 2,2-difluoroethyl trifluoromethanesulfonate (0.48 mL, 3.6 mmol, 1.2 equiv) was added dropwise at 0ºC. The reaction was allowed to proceed at room temperature for 16 h. After completion of the reaction, the mixture was quenched with H_2O and extracted with AcOEt (3 x 15 mL). The combined organic layers were further washed with brine and dried over anhydrous Na_2SO_4. The organic phase was concentrated under reduced pressure and purified by flash column chromatography through silica gel using hexane / EtOAc (3:1) to yield compound **2c** as colorless oil (0.81 g, 2.2 mmol, 73% yield). **^{1}H NMR** (500 MHz, $CDCl_3$), δ (ppm): 8.18 (d, *J* = 8.1 Hz, 2H), 7.76 (d, *J* = 8.1 Hz, 2H), 6.03 (tt, *J* = 55.4, 4.3 Hz, 1H), 4.06 (td, *J* = 13.4, 4.3 Hz, 2H), 1.47 (s, 9H). **^{13}C NMR** (126 MHz, $CDCl_3$), δ (ppm): 163.6, 153.9, 135.7 (q, *J* = 32.9 Hz), 130.5, 130.5, 125.9 (q, *J* = 3.6 Hz), 123.5 (q, *J* = 273.0 Hz), 113.6 (t, *J* = 242.9 Hz), 84.1, 52.7 (t, *J* = 28.7 Hz), 28.1. **^{19}F{^{1}H} NMR** (377 MHz, $CDCl_3$), δ (ppm): -63.4 (3F), -121.6 (2F). **FT-IR** (cm^{-1}, neat, ATR), ṽ = 2983, 2937, 1773, 1726, 1412, 1371, 1323, 1260, 1235, 1166, 1123, 1065, 1003, 853, 768, 700. **HRMS** (ESI+): $[M+Na]^+$ Calcd. for $C_{15}H_{16}F_5NO_4Na$ 392.0892; found 392.0889.

Synthesis of tert-butyl (2-fluoroethyl)((4-(trifluoromethyl)benzoyl)oxy)carbamate (2d)

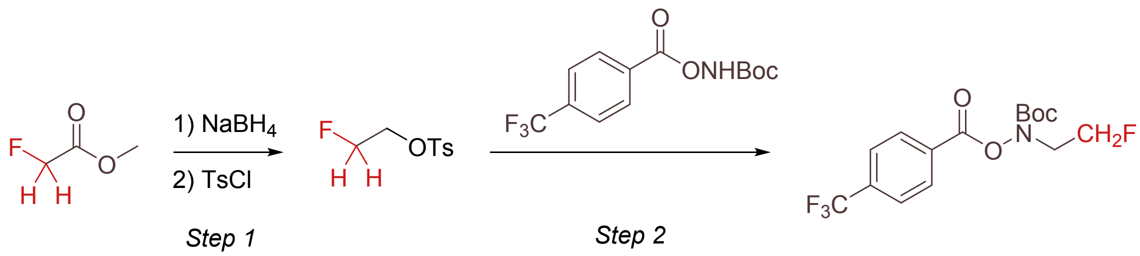

Step 1: Synthesis of 2-Fluoroethyl 4-Methylbenzenesulfonate

Modified from a combination of reported procedures.[3,4] Into a 250 mL Schlenk flask, methyl 2-fluoroacetate (4.97 g, 54 mmol, 1.0 equiv) was dissolved in 100 mL of dry THF. Subsequently, $NaBH_4$ (4.19 g, 111 mmol, 8.0 equiv) was suspended in dry THF and added dropwise to the reaction mixture at 0ºC. Once added, the reaction was allowed to proceed at 0ºC for 3 h. After completion of the reaction, the crude mixture was filtered and 20 drops of concentrated aqueous HCl solution was added to the combined organic layer. Then, anhydrous Na_2SO_4 was added, and the mixture was stirred for 30 minutes. Subsequently, the crude mixture was further filtered and transferred to a 250 mL Schlenk flask. Finally, NEt_3 (7.08 g, 70 mmol, 1.3 equiv) was added to the mixture and left to react for 10 minutes. Then, 4-methylbenzenesulfonyl chloride (13.34 g, 70 mmol, 1.3 equiv) was added portionwise at 0ºC and the reaction was allowed to proceed overnight. Once completed, the mixture was washed with H_2O and brine, dried over anhydrous Na_2SO_4, filtered and concentrated under reduced pressure. After purification by flash column chromatography using hexane / EtOAc (4:1) 2-fluoroethyl 4-methylbenzenesulfonate was obtained as an oil (472 mg, 2.2 mmol, 4%).[4]

^{1}H NMR (600 MHz, $CDCl_3$), δ (ppm) = 7.81 (d, J = 8.3 Hz, 2H), 7.36 (d, J = 8.0 Hz, 2H), 4.63 – 4.59 (m, 1H), 4.57 – 4.51 (m, 1H), 4.31 – 4.26 (m, 1H), 4.26 – 4.22 (m, 1H), 2.45 (s, 3H).

Step 2: Synthesis of Tert-butyl (2-Fluoroethyl)((4-(trifluoromethyl)benzoyl)oxy)carbamate

In a 100 mL round bottomed flask *tert*-butyl ((4-(trifluoromethyl)benzoyl)oxy)carbamate (0.51 g, 1.66 mmol, 1.0 equiv) was dissolved in 8 mL of dry DMF and the solution was cooled down using an ice/water bath. Then, NaH (0.10 g, 60 % in mineral oil, 2.5 mmol, 1.5 equiv) was slowly added portion wise and the reaction was further stirred at same temperature for 30 min. Subsequently, freshly prepared 2-fluoroethyl 4-methylbenzenesulfonate (0.44 g, 2.0 mmol, 1.2 equiv) from *Step 1* was dissolved in 5 mL of dry DMF and added dropwise. After the addition, the ice/water bath was removed, and the reaction was allowed to proceed at room temperature for 16 h. After completion of the reaction, the mixture was quenched with H_2O and extracted with AcOEt (3 x 10 mL). The combined organic layers were further washed with brine and dried over anhydrous Na_2SO_4. The organic phase was concentrated under reduced pressure and purified by silica gel flash column chromatography using hexane/EtOAc (10:1) mixtures to yield compound **2d** as a colorless oil (0.38 g, 1.08 mmol, 65% yield).

---

[3] Li, L-C., Jiang, J-X., Ren, J., Ren, Y., Pittman Jr., C. U., and Zhu, H-J. *Eur. J. Org. Chem.* **2006**, *8*, 1981-1990.
[4] Lin, Y., Yu, Z., Yu, W., Liao, S-L., Zhang, E., Guo, X., Huang, Z., Chen, Y., Qin, J., Cui, Y. and Bao, Z. *J. Mater. Chem. A.*, **2024**, *12*, 2986-2993.

R_f = 0.44 (silica gel, *n*-hexane / EtOAc, 10:1 (v/v)). **^{1}H NMR** (500 MHz, $CDCl_3$), δ (ppm) = 8.19 (d, J = 8.2 Hz, 2H), 7.75 (d, J = 8.2 Hz, 2H), 4.65 (dt, J = 47.1, 4.9 Hz, 2H), 4.03 (dt, J = 24.9, 5.0 Hz, 2H), 1.47 (s, 9H). **^{13}C{^{1}H} NMR** (151 MHz, $CDCl_3$), δ (ppm) = 163.8, 154.5, 135.5 (q, J = 32.8 Hz), 130.9, 130.5, 125.9 (q, J = 3.7 Hz, 2C), 123.6 (q, J = 272.9 Hz), 83.4, 80.9 (d, J = 171.2 Hz), 51.0 (d, J = 21.1 Hz), 28.2. **^{19}F{^{1}H} NMR** (377 MHz, $CDCl_3$), δ (ppm) = -63.31 (3F), -223.32 (1F). **FT-IR** (cm^{-1}, neat, ATR), ṽ = 3271, 2982, 2936, 1771, 1722, 1323, 1233, 1163, 1127, 1065, 1009, 857, 768, 700. **HRMS** (ESI+) calcd for $C_{15}H_{17}F_4NO_4Na$ $[M+Na]^+$: 374.0986, found 374.0985.

Tert-butyl ethyl((4-(trifluoromethyl)benzoyl)oxy)carbamate (2e)

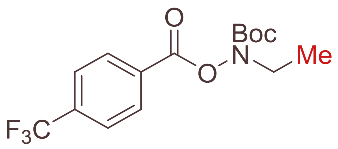

In a 100 mL round bottomed flask *tert*-butyl ((4-(trifluoromethyl)benzoyl)oxy)carbamate (500 mg, 1.6 mmol, 1.0 equiv) is dissolved in 13 mL of dry DMF. Then, NaH (104 mg, 60 % in mineral oil, 6.0 mmol, 1.6 equiv) was slowly added portion wise at 0ºC and the reaction was further stirred at 0ºC for 30 min. Subsequently, ethyl iodide (0.2 mL, 2.4 mmol, 1.5 equiv) was added dropwise at 0ºC. The reaction was allowed to proceed at room temperature for 16 h. After completion of the reaction, the mixture was quenched with H_2O and extracted with AcOEt (3 x 15 mL). The combined organic layers were further washed with brine and dried over anhydrous Na_2SO_4. The organic phase was concentrated under reduced pressure and purified by flash column chromatography through silica gel using DCM to yield compound **2e** as colorless oil (258 mg, 0.77 mmol, 48% yield). **^{1}H NMR** (500 MHz, $CDCl_3$), δ (ppm): 8.19 (d, J = 8.1 Hz 2H), 7.74 (d, J = 7.0 Hz, 2H), 3.75 (q, J = 7.1 Hz, 2H), 1.46 (s, 9H), 1.24 (td, J = 7.1, 1.7 Hz, 3H). **^{13}C NMR** (126 MHz, $CDCl_3$), δ (ppm): 163.8, 155.0, 135.3 (q, J = 32.6 Hz), 131.2, 130.4, 125.8 (q, J = 3.5 Hz), 123.6 (q, J = 272.8 Hz), 82.9 – 82.7 (m), 46.0, 28.2, 12.4. **^{19}F{^{1}H} NMR** (282 MHz, $CDCl_3$), δ (ppm): -63.3. **FT-IR** (cm^{-1}, neat, ATR), ṽ = 2950, 1754, 1733, 1400, 1326, 1260, 1233, 1160, 1130, 1005, 855, 768. **HRMS** (ESI+) m/z: $[M+Na]^+$ Calcd. for $C_{15}H_{18}F_3NO_4Na$ 356.1080; found 356.1080.

4. Synthesis of β-Trifluoromethyl β-amino Ketones. Reaction Workflow and Compound Characterization for Derivatization Reactions

4.1. Reaction Workflow

All photoinduced reactions were done using a Kessil PR160-violet LED lamp (30 W High Luminous DEX 2100 LED, λ_{max} = 427 nm). The LED was placed 4 cm away from the reaction vial within a ventilated fume hood and using a fan to maintain the temperature approximately at 25ºC.

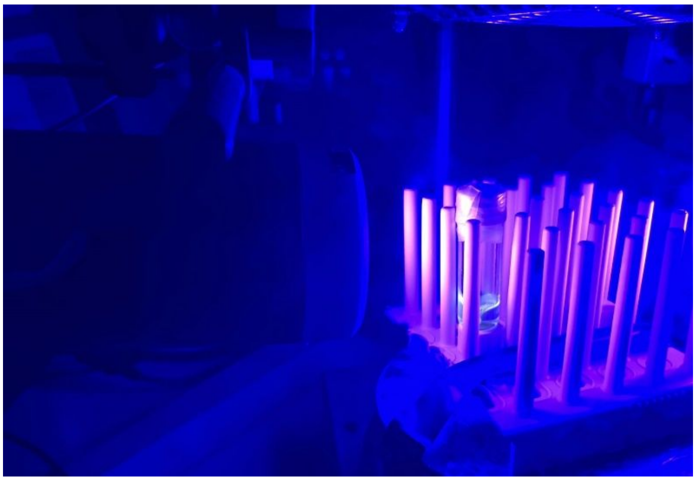

Figure S1*. Reaction setup for the photoinduced synthesis of fluorinated β-amino ketones*

*4.2. Compound characterization data (**28-31**)*

3-Amino-1-(4-(*tert*-butyl)phenyl)-4,4,4-trifluorobutan-1-one (28)

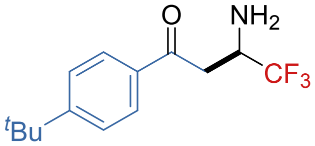

In a 50 mL round bottomed flask compound **3** (0.30 g, 0.80 mmol, 1.0 equiv) is dissolved in 7.5 mL of DCM. Then, TFA (1.5 mL) was slowly added at 0ºC. The reaction was allowed to proceed at room temperature for 4 h. After completion of the reaction, the mixture was quenched with $NaHCO_3$ and extracted with DCM (3 x 10 mL). The combined organic layers were dried over anhydrous Na_2SO_4, concentrated under reduced pressure and purified by flash column chromatography using hexane/EtOAc (2:1) to yield compound **28** as a yellow oil (218 mg, 0.8 mmol, 99% yield). $\mathbf{R_f}$ = 0.25, (silica gel, *n*-hexane / EtOAc 4:1). **^{1}H NMR** (400 MHz, $CDCl_3$), δ (ppm): 7.94-7.84 (m, 2H), 7.54-7.46 (m, 2H), 4.07-3.95 (m, 1H), 3.30 (dd, *J* = 17.3, 2.8 Hz, 1H),

3.15 (dd, J = 17.4, 9.7 Hz, 1H), 1.62 (s, 2H), 1.34 (s, 9H). **$^{13}C\{^1H\}$ NMR** (101 MHz, $CDCl_3$), δ (ppm): 196.0, 157.8, 133.9, 128.2, 126.6 (q, J = 280.2 Hz), 125.9, 50.4 (q, J = 29.7 Hz), 39.3, 35.3, 31.2. **$^{19}F\{^1H\}$ NMR** (377 MHz, $CDCl_3$), δ (ppm): -78.25. **FT-IR** (cm^{-1}, neat, ATR), ṽ = 3342, 2964, 1690, 1600, 1453, 1384, 1350, 1234, 1163, 1119, 1054, 992, 893, 860. **HRMS** (ESI+) m/z: $[M+H]^+$ Calcd. for $C_{14}H_{18}F_3NO$ 274.1419; found 274.1414.

***Tert*-butyl (4-(4-(*Tert*-butyl)phenyl)-1,1,1-trifluoro-4-hydroxybutan-2-yl)carbamate (29)**

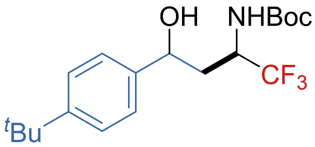

Into a 5 mL round bottomed flask compound **3** (186 mg, 0.5 mmol, 1.0 equiv) was dissolved in 0.6 mL of EtOH. Simultaneously, $NaBH_4$ (20.0 mg, 0.5 mmol, 12.8 equiv) was suspended in drops of H_2O in an Erlenmeyer. Then, the suspension was slowly added to the initial mixture. The reaction is monitored by thin layer chromatography. Upon completion of the reaction, 5 mL of aqueous solution of NaOH 1 M was added to the mixture and then diluted with 5 mL of Et_2O. The organic layer was separated, and the aqueous layer was further extracted with Et_2O (3 x 5 mL). The combined organic layers were dried over Na_2SO_4, concentrated under reduced pressure and purified by flash column chromatography to yield compound **29** as two separable diastereomers (1:1 ratio, 85% yield). After purification by flash column chromatography (5:1, hexane:AcOEt), the first diastereomer (**29-*d1***) was isolated as a yellow oil (80.7 mg, 0.22 mmol, 43%) and the second one (**29-*d2***) was obtained as a yellow oil (80.6 mg, 0.22 mmol, 42%).

Data for **29-*d1*.** **^1H NMR** (500 MHz, $CDCl_3$), δ (ppm) = 7.38 (d, J = 7.9 Hz, 2H), 7.30 (d, J = 8.0 Hz, 2H), 4.95 (d, J = 10.1 Hz, 1H), 4.76 (d, J = 10.8 Hz, 1H), 4.62 (q, J = 9.0 Hz, 1H), 3.01 (s, 1H), 2.14 (ddd, J = 14.0, 10.8, 2.7 Hz, 1H), 1.79 (td, J = 12.7, 6.1 Hz, 1H), 1.49 (s, 9H), 1.32 (s, 9H). **$^{13}C\{^1H\}$ NMR** (126 MHz, $CDCl_3$), δ (ppm) = 156.1, 151.0, 140.4, 125.7, 125.5 (q, J = 281.2 Hz), 125.4, 81.3, 69.4, 50.4 (q, J = 30.5 Hz), 38.6, 34.7, 31.5, 28.4. **$^{19}F\{^1H\}$ NMR** (376 MHz, $CDCl_3$), δ (ppm) = -75.7. **FT-IR** (cm^{-1}, neat, ATR), ṽ = 3335, 2965, 2930, 1703, 1511, 1392, 1367, 1270, 1248, 1175, 1130, 1051, 1030. **HRMS** (ESI+) calcd for $C_{19}H_{29}F_3NO_3$ $[M+H]^+$: 376.2094, found 376.2094.

Data for **29-*d2*.** **^1H NMR** (500 MHz, $CDCl_3$), δ (ppm) = 7.40 (d, J = 8.0 Hz, 2H), 7.29 (d, J = 8.1 Hz, 2H), 4.82 (t, J = 6.8 Hz, 1H), 4.78-4.72 (m, 1H), 4.55-4.19 (bs, 1H), 2.15-1.95 (m, 3H), 1.46 (s, 9H), 1.32 (s, 9H). **$^{13}C\{^1H\}$ NMR** (126 MHz, $CDCl_3$), δ (ppm) = 155.2, 151.4, 140.3, 125.9, 125.4 (q, J = 281.1 Hz), 125.7, 80.8, 71.8, 50.7 (q, J = 30.5 Hz), 37.7, 34.7, 31.5 , 28.4. **$^{19}F\{^1H\}$ NMR** (376 MHz, $CDCl_3$), δ (ppm) = -76.9. **FT-IR** (cm^{-1}, neat, ATR), ṽ = 3335, 2965, 2930,

1703, 1511, 1392, 1367, 1270, 1248, 1175, 1130, 1051, 1030. **HRMS** (ESI+) calcd for $C_{19}H_{29}F_3NO_3$ $[M+H]^+$: 376.2094, found 376.2094.

5-(4-(*Tert*-butyl)phenyl)-3-(trifluoromethyl)-1*H*-pyrazole (30)

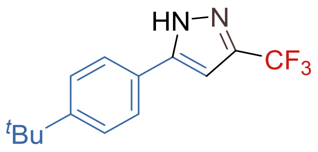

Compound **30** was prepared according to a protocol from literature[5] using compound **28** (41.0 mg, 0.15 mmol). After purification by column chromatography (hexane:EtOAc 9:1), the title compound **30** was obtained as a white solid (20.6 mg, 0.08 mmol, 51% yield). $\mathbf{R_f}$ = 0.53, (silica gel, hexane / EtOAc 4:1). **^{1}H NMR** (400 MHz, $CDCl_3$), δ (ppm): 11.07 (s, 1H), 7.54-7.47 (m, 4H), 6.75 (s, 1H), 1.36 (s, 9H). **^{13}C{^{1}H} NMR** (126 MHz, $CDCl_3$), δ (ppm): 153.0, 145.1, 144.2 (q, *J* = 37.7 Hz), 126.4, 125.6, 125.3, 121.3 (q, *J* = 268.7 Hz), 101.1 (m), 35.0, 31.3. **^{19}F{^{1}H} NMR** (377 MHz, $CDCl_3$), δ (ppm): -62.3. **FT-IR** (cm^{-1}, neat, ATR), ṽ = 3251, 2969, 2873, 1491, 1367, 1303, 1270, 1155, 1122, 1101, 981, 837, 806, 749. **HRMS** (ESI+) m/z: $[M+H]^+$ Calcd. for $C_{14}H_{16}F_3N_2$ 269.1260; found 269.1264.

(4-(*Tert*-butyl)phenyl)(3-(trifluoromethyl)aziridine-2-yl)methanone (31)

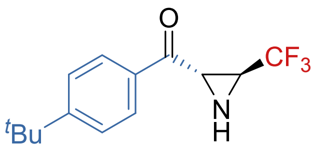

Compound **31** was prepared according to a protocol from literature[6] using compound **28** (27.3 mg, 0.10 mmol). After purification by column chromatography (hexane:EtOAc, 5:1), the title compound **31** was obtained as a colorless oil (22.6 mg, 0.08 mmol, 84% yield). $\mathbf{R_f}$ = 0.33, (silica gel, *n*-hexane / EtOAc 4:1). **^{1}H NMR** (400 MHz, $CDCl_3$), δ (ppm): 7.98 (d, *J* = 8.3 Hz, 2H), 7.57 (d, *J* = 8.2 Hz, 2H), 3.72 (dd, *J* = 8.1, 2.4 Hz, 1H), 2.78-2.72 (m, 1H), 2.36 (t, *J* = 9.0 Hz, 1H), 1.37 (s, 9H). **^{19}F{^{1}H} NMR** (377 MHz, $CDCl_3$), δ (ppm): -71.49. **HRMS** (ESI+) m/z: $[M+H]^+$ Calcd. for $C_{14}H_{17}F_3NO$ 272.1257; found 272.1259.

[5] Mei, H., Wang, N., Li, Z. and Han, J. *Org. Lett.* **2022**, *24*, 2258-2263.
[6] Wang, N., Du, Y., Mei, H., Escorihuela, J. and Han, J. *Org. Biomol. Chem.* **2023**, *23*, 4988-4992.

5. Large-Scale Synthesis of Compound 3

In a 50 mL Schlenk flask equipped with a magnetic stirring bar, the corresponding styrene **1a** (320 mg, 2.0 mmol, 1.0 equiv), 4DPAIPN (32 mg, 0.04 mmol, 0.02 equiv) and hydroxylamine **2a** (1.55 g, 4.0 mmol, 2.0 equiv) were added. Then, 20 mL of dry DMSO were added under inert atmosphere and the reaction was degassed with Argon for 20 seconds. The reaction mixture was irradiated for 4 hours with a 427 nm Kessil PR160-purple LED as seen in Figure S2. The temperature of the reaction was maintained at approximately 25 °C via a fan. Upon completion, the reaction mixture was diluted with AcOEt (40 mL) and washed with brine (3 x 40 mL). The organic layer was dried over anhydrous Na_2SO_4, filtered and concentrated under reduce pressure. The crude mixture was subjected to flash column chromatography purification using hexanes/EtOAc 10:1 to yield the desired compound **3** (686 mg, 1.84 mmol, 90% yield). ***Caution!*** *Proper protective eyewear, specifically goggles designed to shield against the emitted wavelengths, was used to prevent potential eye damage from prolonged exposure to the light source. Researchers are advised to take similar precautions when reproducing this work.*

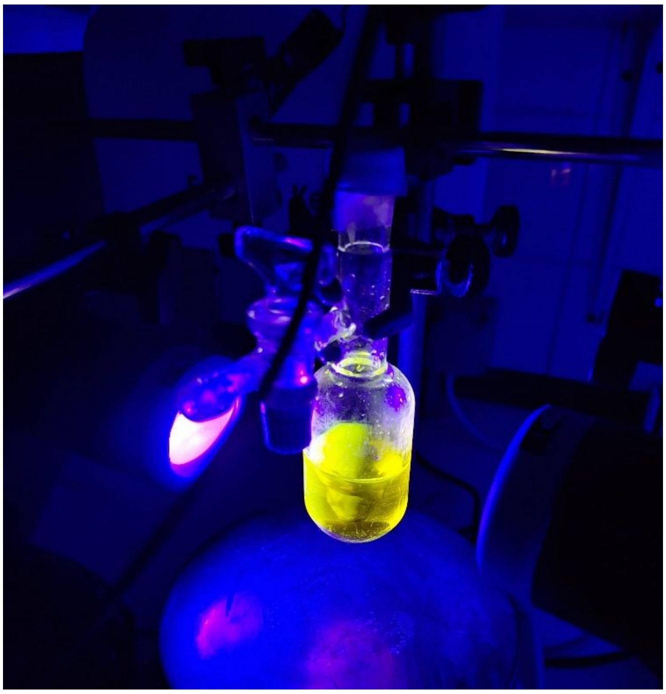

Figure S2**. Reaction setup for the gram-scale photoinduced synthesis of compound **3

6. Unproductive Substrates

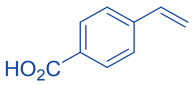

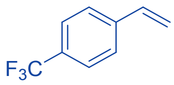

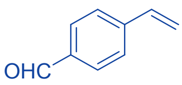

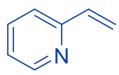

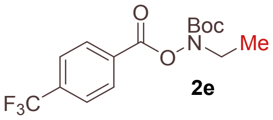

2e

7. Mechanistic Investigation

6.1. *Cyclic Voltammetry Experiment and Redox Data*

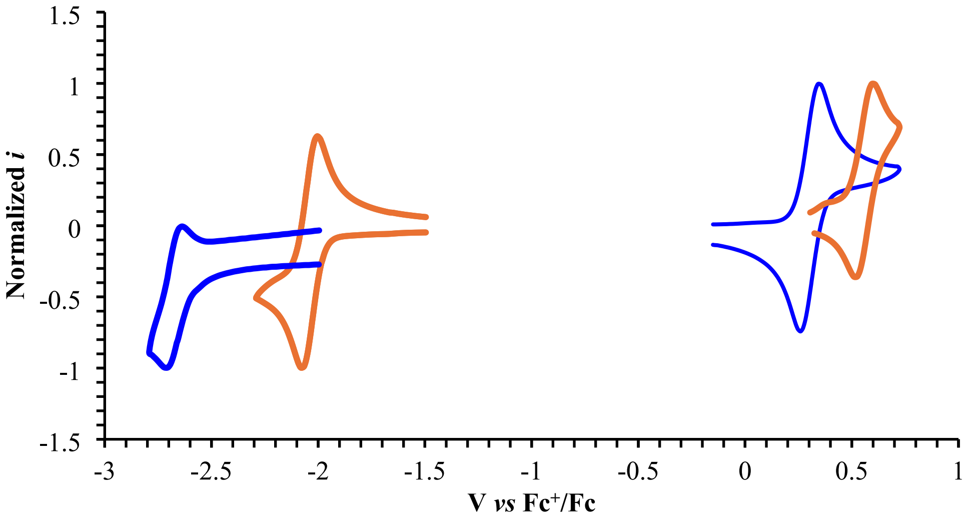

Figure S3: *Cyclic voltammetry of compounds Ir(ppy)$_3$ (**blue**), 4DPAIPN (**orange**). Conditions: Ir(ppy)$_3$ 2.3 mM (100 mV/s), 4DPAIPN 2.6 mM (100 mV/s), 4CzIPN 2.9 mM (100 mV/s) in MeCN, TBAPF$_6$ 0.1 M, rt. All measurements start at 0.0 V vs reference electrode. Scan direction to negative potentials for reductive scans and positive potentials for oxidative scans. Glassy carbon disk as working electrode, platinum wire as auxiliary electrode and SCE or AgNO$_3$/Ag as reference electrode. IUPAC plotting.*

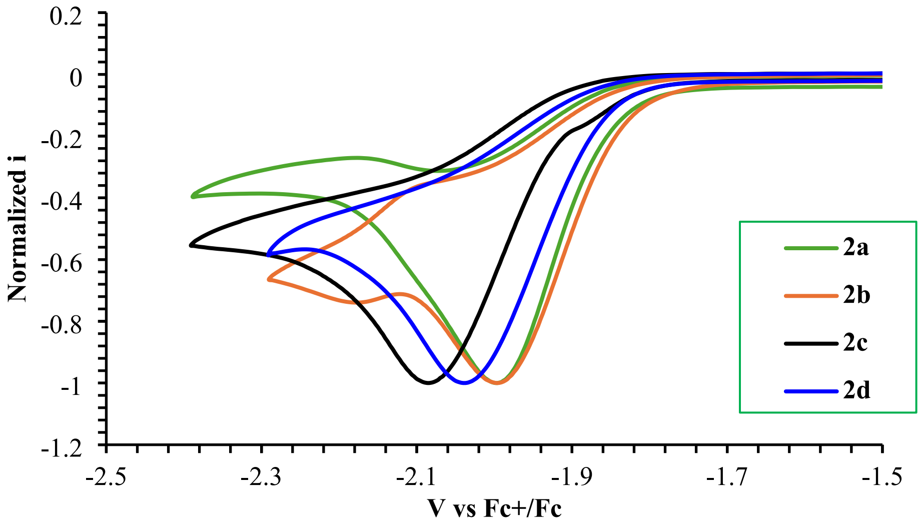

Figure S4: *Cyclic voltammetry of redox active species* ***2a*** *(green),* ***2b*** *(orange),* ***2c*** *(black) and* ***2d*** *(blue). Conditions:* ***2a*** *2.5 mM (100 mV/s),* ***2b*** *2.6 mM (100 mV/s),* ***2c*** *2.7 mM (100 mV/s) and* ***2d*** *2.6 mM (100 mV/s) in MeCN, $TBAPF_6$ 0.1 M, rt. Start at 0.0 V, scan direction to negative potentials. Glassy carbon disk as working electrode, platinum wire as auxiliary electrode and SCE as reference electrode. IUPAC plotting.*

Table S1: *Collected redox data of the presented work relevant compounds.*

Compound	$E_{1/2}$ *(P⁺/P)*	$E_{1/2}$ *(P/P⁻)*	E_a	E_c	*E (P⁺/*P)*	*E (*P/P⁻)*
Ir(ppy)₃	0.32	-2.68	-	-	-2.45	0.09
4DPAIPN	0.56	-2.04	-	-	-2.06	0.58
2a	-	-	-	-1.91	-	-
2b	-	-	-	-1.90	-	-
2c	-	-	-	-1.99	-	-
2d	-	-	-	-1.95	-	-

All values are given in V *vs* Fc^+/Fc. For those that presented irreversible waves, E_a (oxidation) or E_c (reduction) are extracted as an approximation of the thermodynamic $E_{1/2}$.

Calculus for the Excited State potentials were performed with the following equation:[7]

1) $E_{1/2}(P^+/{*P}) = E_{1/2}(P^+/P) - E_{0\text{-}0}$
2) $E_{1/2}({*P}/P^-) = E_{1/2}(P/P^-) + E_{0\text{-}0}$
3) E (eV) = 1.2398/λ (in µm), then $E_{0\text{-}0}$ is when $\lambda = (\lambda_{max,abs} + \lambda_{max,em}) / 2$ (*Rehem-Weller equation)*

[7] Tucker, J. W. and Stephenson. C. R. *J. Org. Chem.* **2012**, *77*, 1617-1622.

6.2. TEMPO Experiment

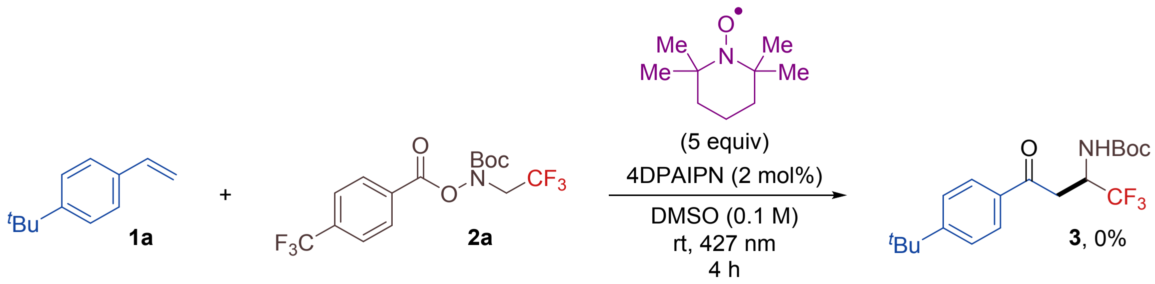

To a 4 mL vial equipped with a magnetic stirring bar, styrene **1a** (16.0 mg, 0.1 mmol, 1.0 equiv), 4DPAIPN (1.6 mg, 0.002 mmol, 2 mol%), hydroxylamine **2a** (38.7 mg, 0.2 mmol, 2.0 equiv.) and TEMPO (78.1 mg, 0.5 mmol, 5 equiv) were added. Then, 1.0 mL of dry DMSO was added under inert atmosphere and the reaction was degassed with Argon for 20 seconds. The reaction mixture was irradiated for 4 hours with a 427 nm Kessil PR160-purple LED as described in the "*Workflow*" section. The temperature of the reaction was maintained at approximately 25 °C via a fan. Upon completion, the reaction mixture was diluted with AcOEt (10 mL) and washed with brine (3 x 10 mL). The organic layer was dried over anhydrous Na_2SO_4, filtered and concentrated under reduce pressure. The crude mixture was analyzed by ^{1}H-NMR using 1,3,5-trimethoxybenzene as internal standard resulting in 0% yield of compound **3**.

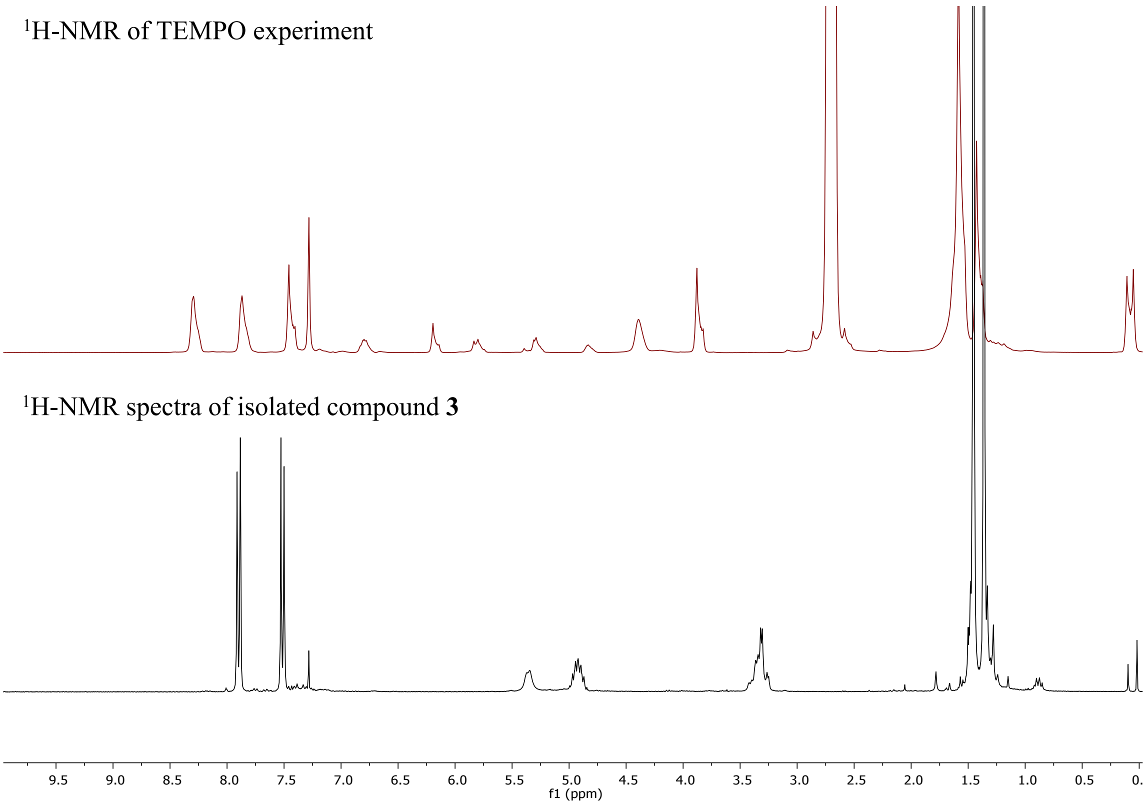

Figure S5: *Comparison between ^{1}H NMR (300 MHz, $CDCl_3$) of the TEMPO experiment reaction using 1,3,5-trimethoxybenzene as internal standard and the pure spectra of **3**.*

6.3. *Stern-Volmer Quenching Studies*

Fluorescence measurements were obtained using septa-capped UV-Quartz cuvettes (10 mm pathlength) obtained from Hellma Analytics. Excitation was performed at 400 nm; fluorescence spectra were obtained from 300-700 nm. The stock solutions were prepared as follows:

1. 4DPAIPN photocatalyst solution (0.0002 mM): To a 25 mL volumetric flask 3.90 mg of 4DPAIPN were dissolved in DMSO. Subsequently, 10 μL of the previous solution were diluted in a 10 mL volumetric flask with more DMSO obtaining a 1.96×10^{-6} M stock solution.
2. *Redox active species* **2a** (hydroxylamine) solution (4 mM): To a 10 mL volumetric flask 15.7 mg of **2a** were dissolved in DMSO obtaining a 4.00×10^{-3} M stock solution.
3. Styrene **1a** solution (4 mM): To a 10 mL volumetric flask 6.6 mg of **1a** and was dissolved in DMSO obtaining a 4.13×10^{-3} M stock solution.

Once the stock solutions were prepared, they were kept in the dark using aluminum foil. Then, the solutions were allocated to the cuvettes and fluorescence quenching was determined with individual quenchers (hydroxylamine and styrene). 1 mL of the photocatalyst solution was added along with 1 mL of DMSO for the initial measurement. For the experiments with quencher, 1 mL of photocatalyst solution was added together with increasing amounts of quencher (0, 10, 50, 500 and 1000 μL) and adjusting concentration with DMSO to reach 2 mL. Degassing of each individual solution for 30 seconds was performed prior to recording the data. Linear regression of I_0/I against concentration was carried out to yield the Stern-Volmer quenching rate constant (K_{SV}). The following Stern-Volmer plot for luminescence quenching of 4DPAIPN by two different quenchers was obtained. As depicted above, the excited catalyst (4DPAIPN*) is effectively quenched by the *redox active specie* **2a** with a Stern-Volmer quenching rate constant of 95.8 M^{-1} rather by the styrene substrate.

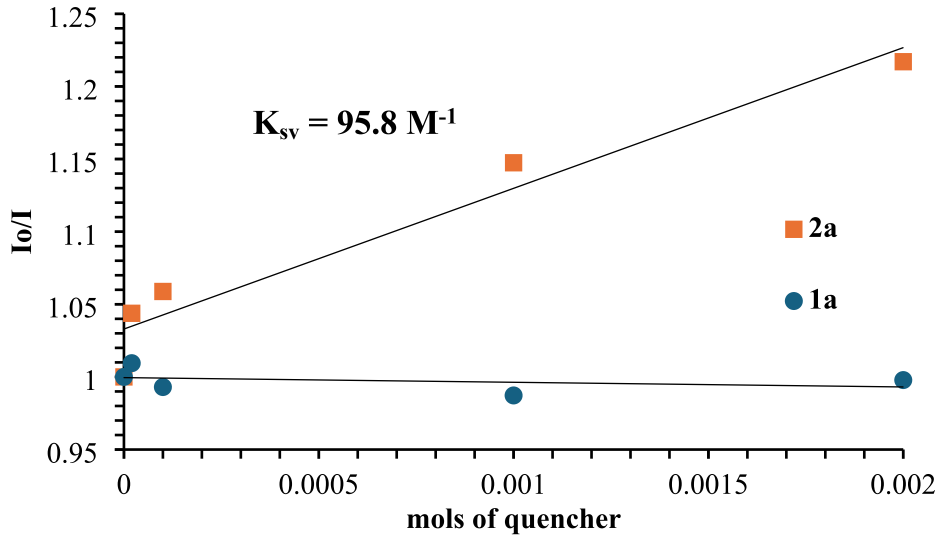

Figure S6*. Stern-Volmer plots for luminescence quenching of 4DPAIPN (2.0 x 10^{-6} M in degassed DMSO) by redox active specie* ***2a*** *(orange) and styrene* ***1a*** *(blue), $\lambda_{exc.}$ = 400 nm, $\lambda_{em.}$ = 530 nm, K_{sv} = Stern-Volmer constant.*

6.4. Isotope Labelling Experiment

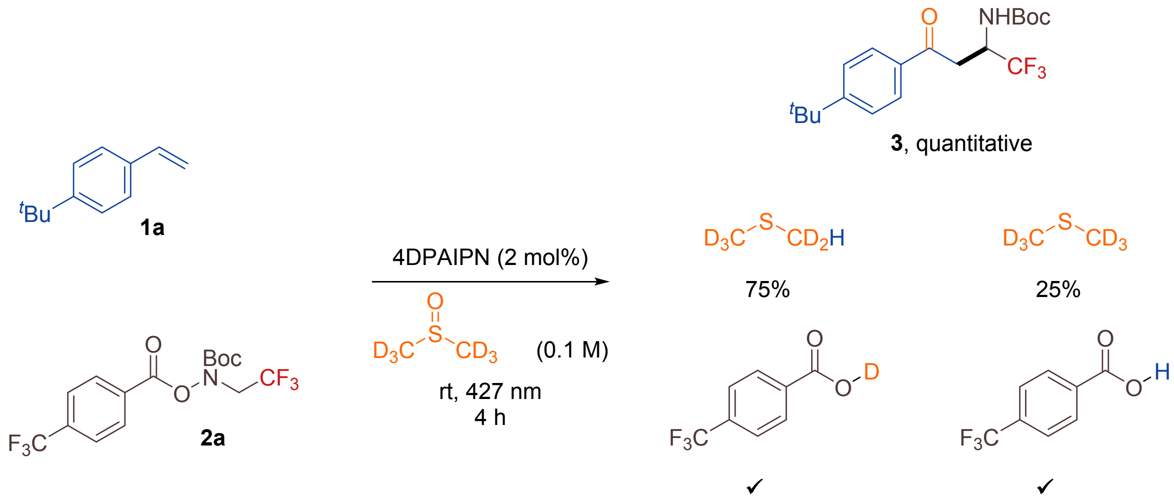

To a 4 mL vial equipped with a magnetic stirring bar, styrene **1a** (16.0 mg, 0.1 mmol, 1.0 equiv), 4DPAIPN (1.6 mg, 0.02 mmol, 0.02 equiv), hydroxylamine **2a** (38.7 mg, 0.2 mmol, 2.0 equiv.) and TEMPO (78.1 mg, 0.5 mmol, 5 equiv) were added. Then, 1.0 mL of dry d6-DMSO was added under inert atmosphere and the reaction was degassed with Argon for 20 seconds. The reaction mixture was irradiated for 4 hours with a 427 nm Kessil PR160-purple LED as described in the "*Workflow*" section. The temperature of the reaction was maintained at approximately 25 °C via a fan. Upon completion, the reaction mixture was analyzed using GC-MS. The starting materials, **1a** and **2a**, were completely consumed, and as expected, the desired compound **3** was detected as

the major product. Notably, we observed the formation of perdeuterated dimethyl sulfide as well as a pentadeuterated derivative, with a product ratio of 75:25 favoring the pentadeuterated compound. Additionally, the formation of deuterated *p*-trifluoromethylbenzoic acid was detected. These results confirm that, following the trapping of the carbocation by d_6-DMSO, the benzoate derivative abstracts an acidic deuterium atom bound to the carbon adjacent to the sulfonium cation, resulting in the formation of a sulfonium ylide intermediate. Subsequently, this intermediate undergoes an intramolecular reaction by attacking the benzylic proton, leading to the 25ormation of the carbonyl compound and the release of D_3CSCH_2D. The 75:30 ratio observed for the sulfide products suggests an alternative, albeit less predominant (Figure S7), mechanistic pathway. In this scenario, the benzoate derivative abstracts the benzylic proton, forming the carbonyl compound and generating perdeuterated dimethyl sulfide (D_3CSCD_3).

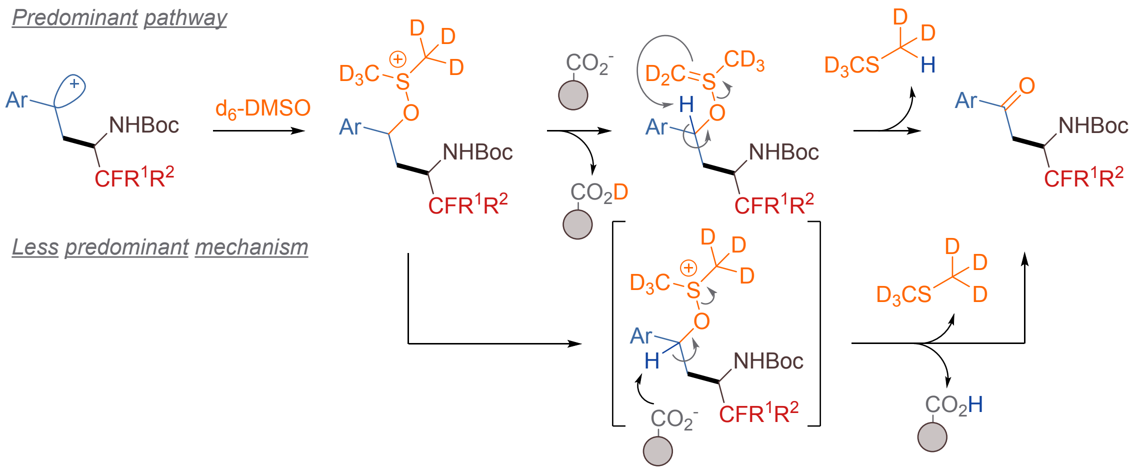

***Figure S7**. Mechanistic pathways*

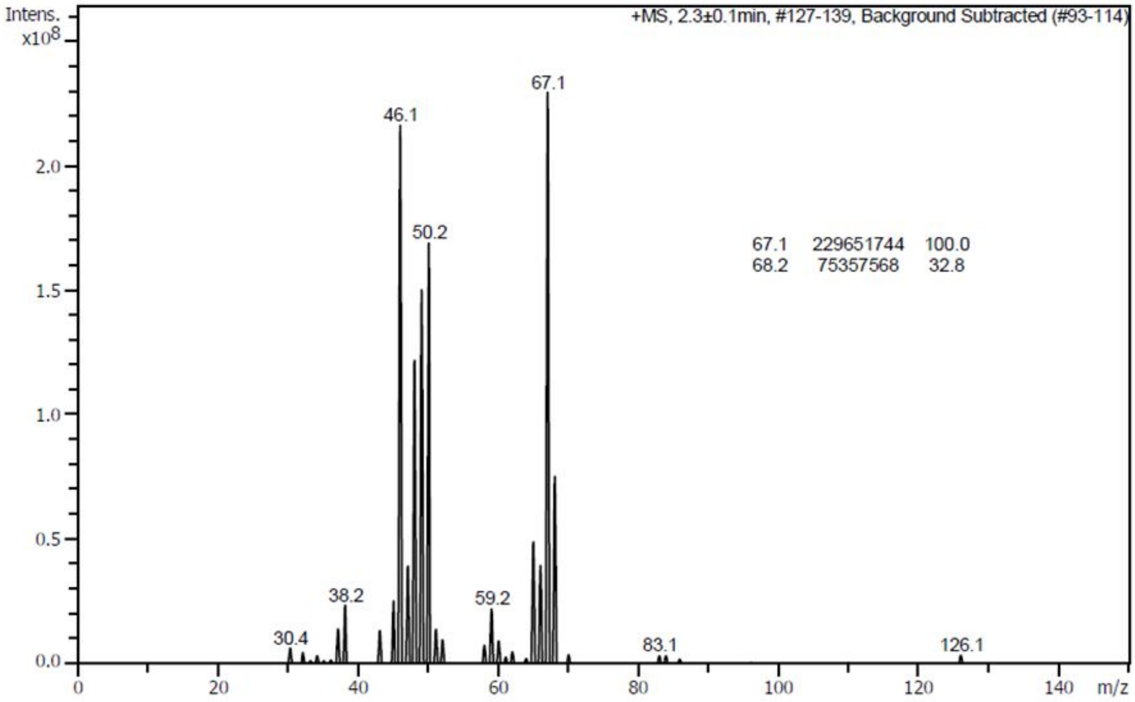
Intens.
x10^8
+MS, 2.3±0.1min, #127-139, Background Subtracted (#93-114)
67.1
46.1
50.2
38.2
59.2
30.4
83.1
126.1
67.1 229651744 100.0
68.2 75357568 32.8
m/z

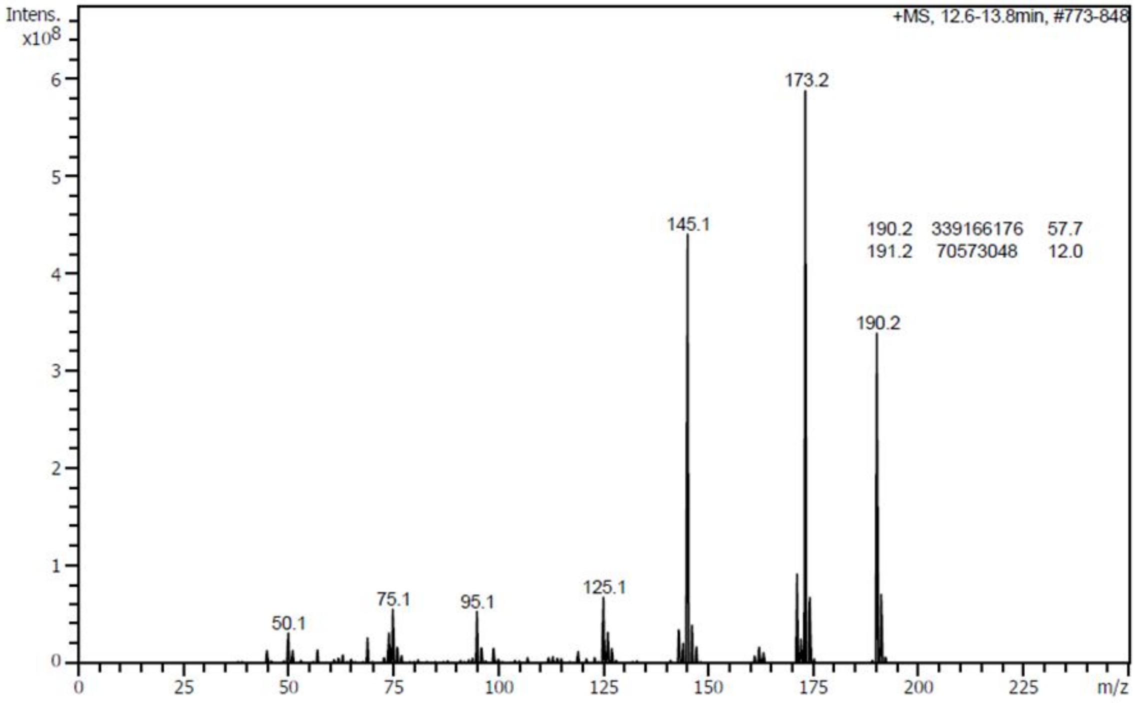
Intens.
x10^8
+MS, 12.6-13.8min, #773-848
173.2
145.1
190.2
125.1
75.1
95.1
50.1
190.2 339166176 57.7
191.2 70573048 12.0
m/z

8. NMR Data

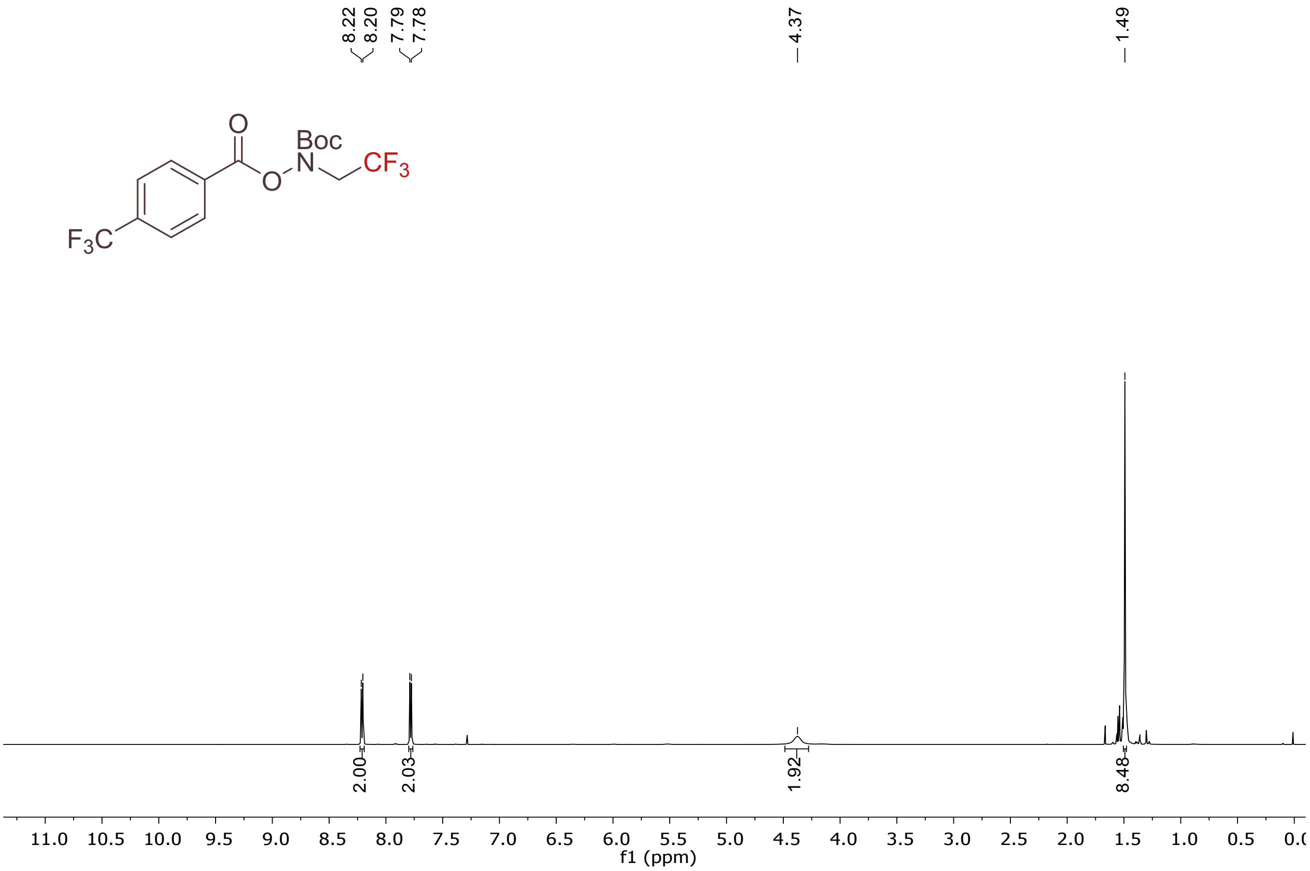

^{1}H NMR (600 MHz, $CDCl_3$) of compound **2a**.

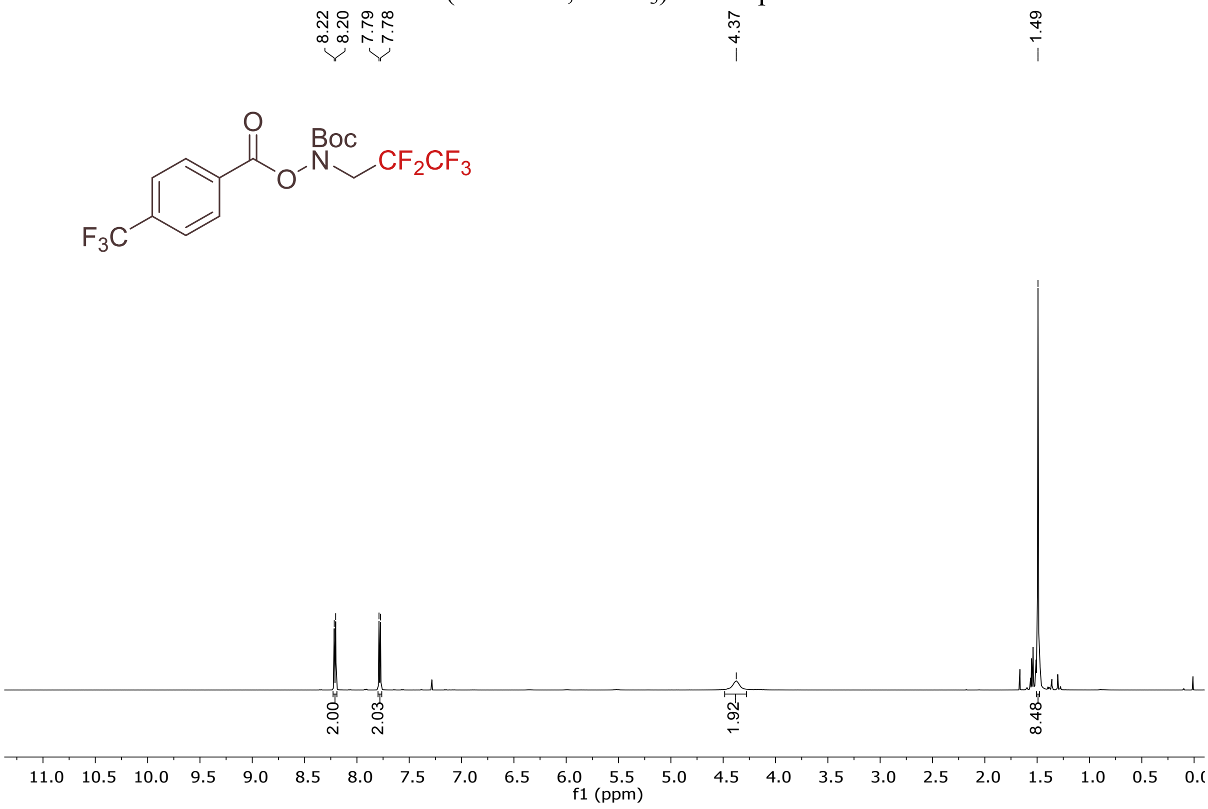

^{1}H NMR (600 MHz, $CDCl_3$) of compound **2b**.

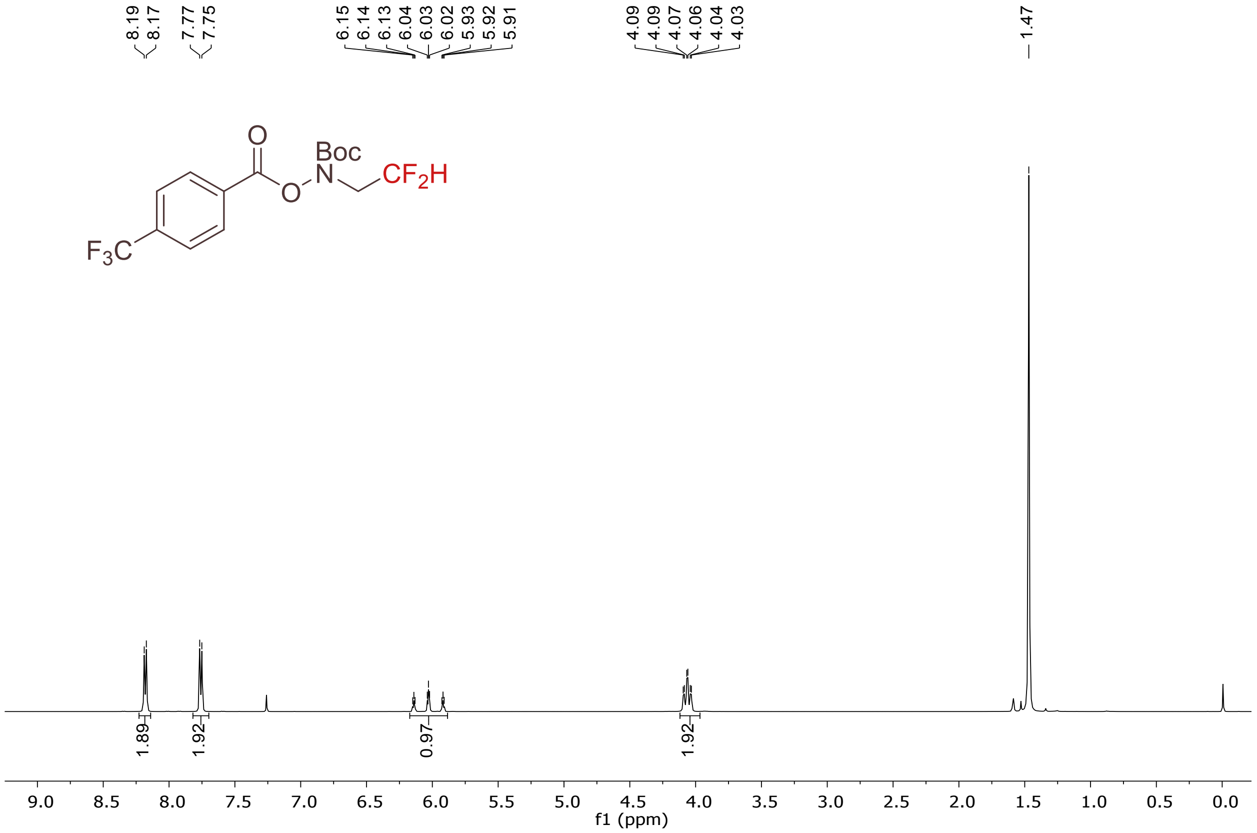

^{1}H NMR (500 MHz, $CDCl_3$) of compound **2c**.

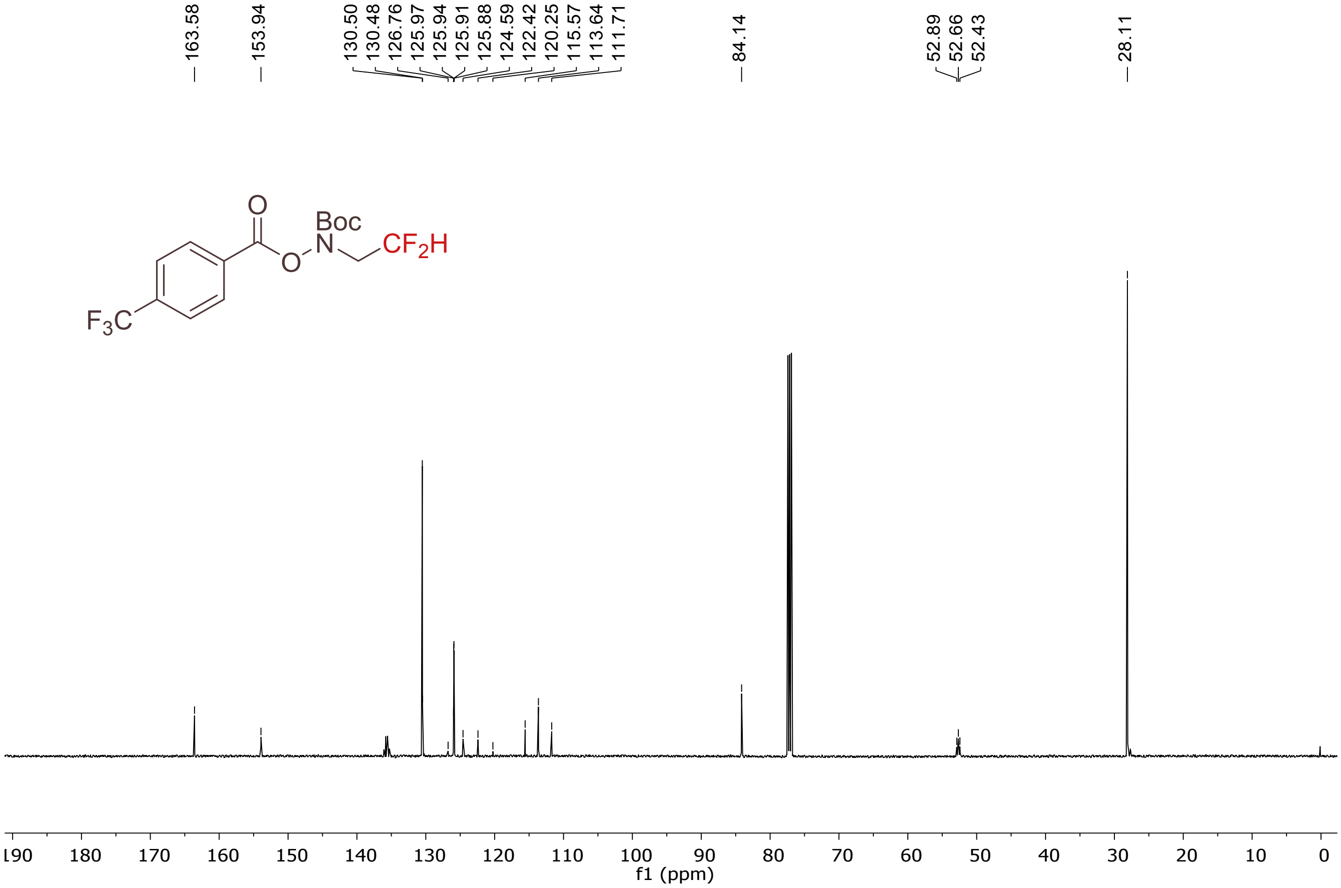

^{13}C{^{1}H} NMR (126 MHz, $CDCl_3$) of compound **2c**.

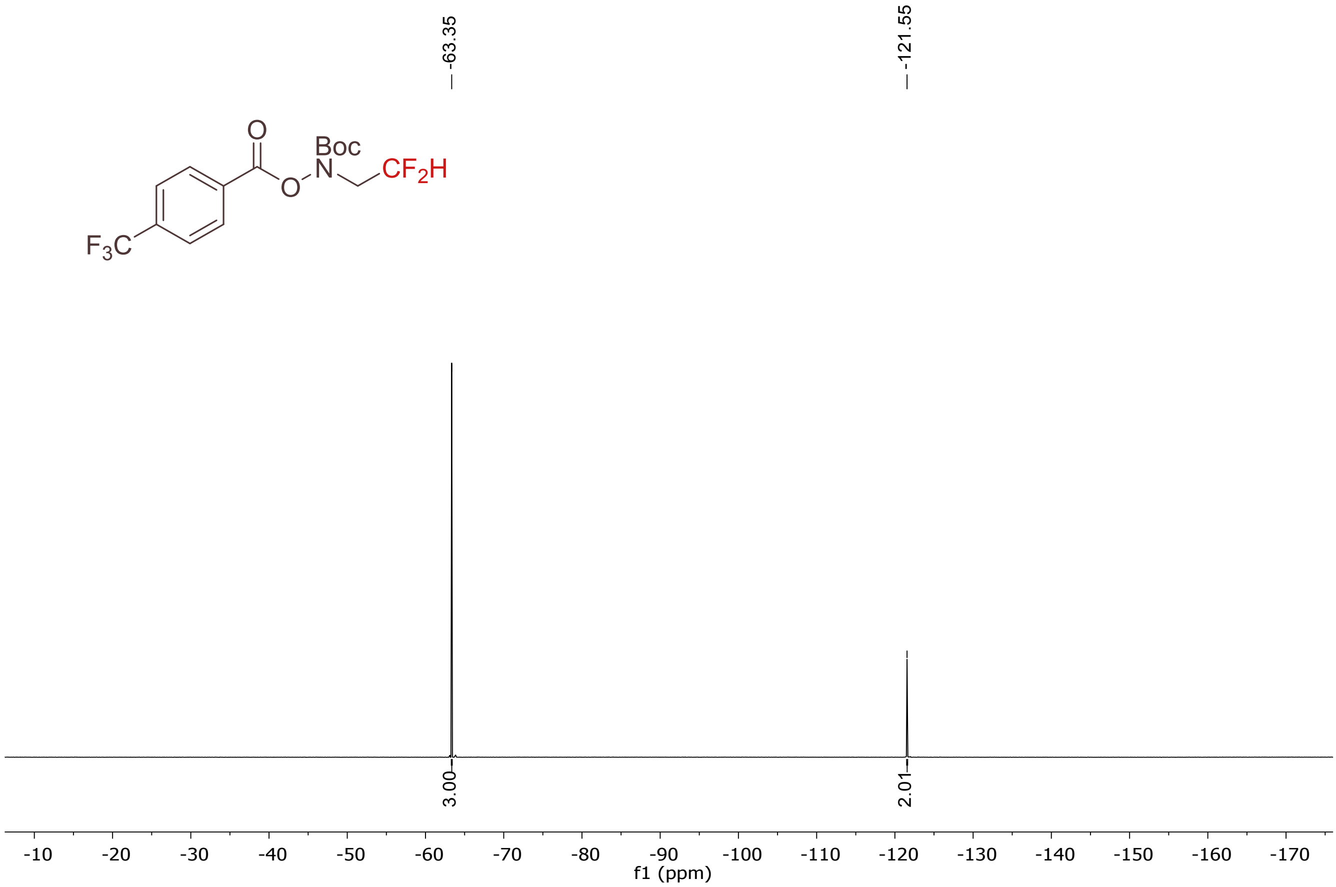

$^{19}F\{^{1}H\}$ NMR (377 MHz, $CDCl_3$) of compound **2c**.

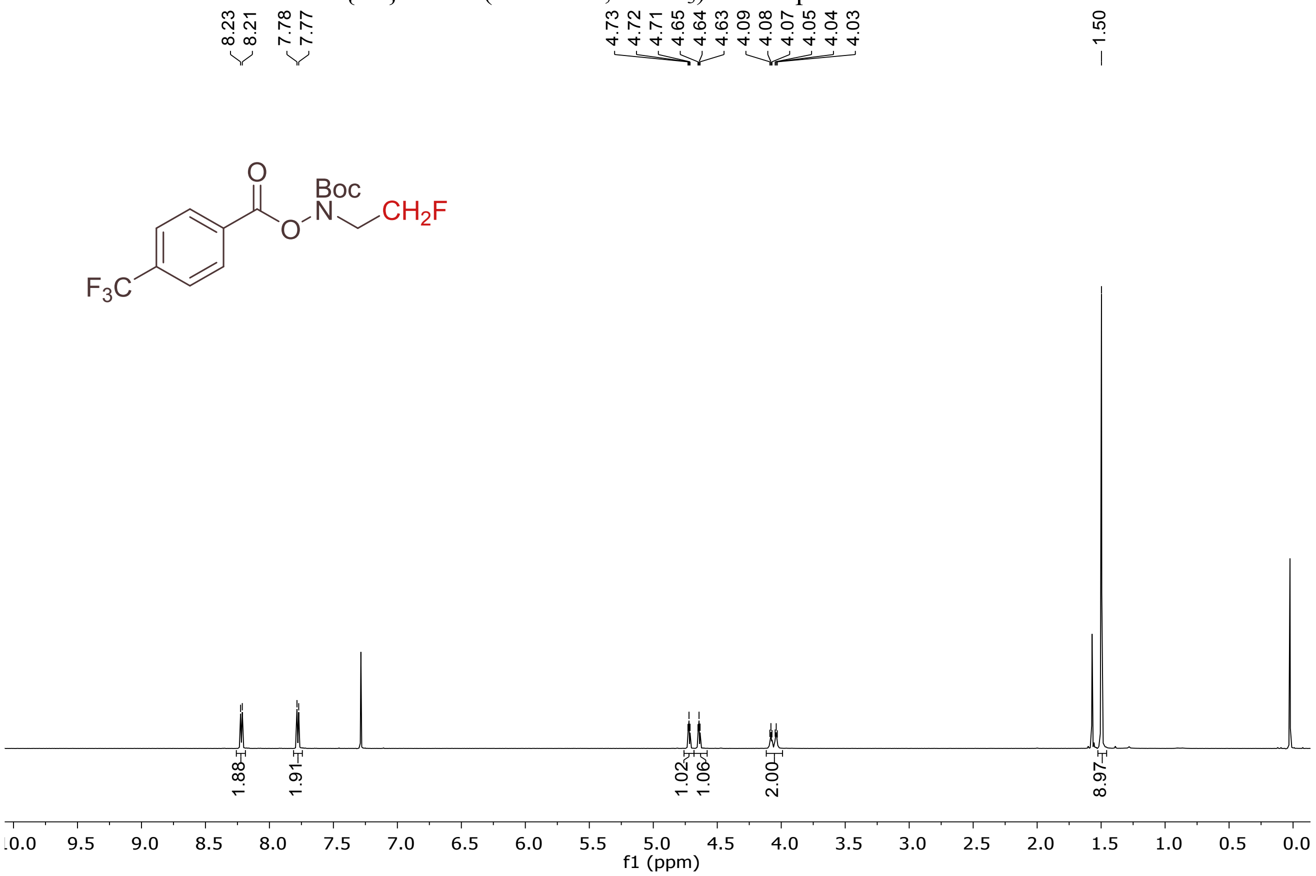

^{1}H NMR (600 MHz, $CDCl_3$) of compound **2d**.

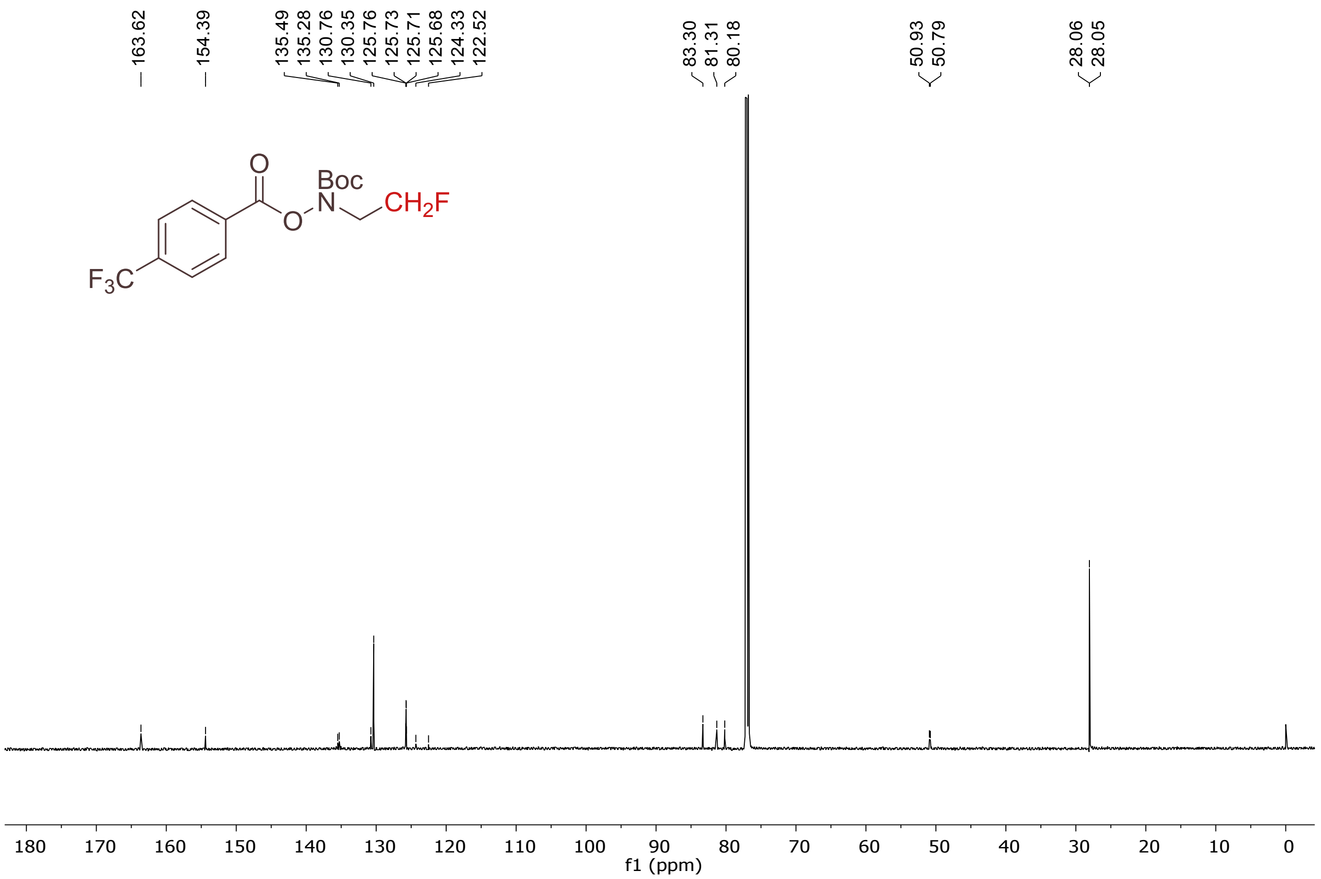

$^{13}C\{^1H\}$ NMR (151 MHz, $CDCl_3$) of compound **2d**.

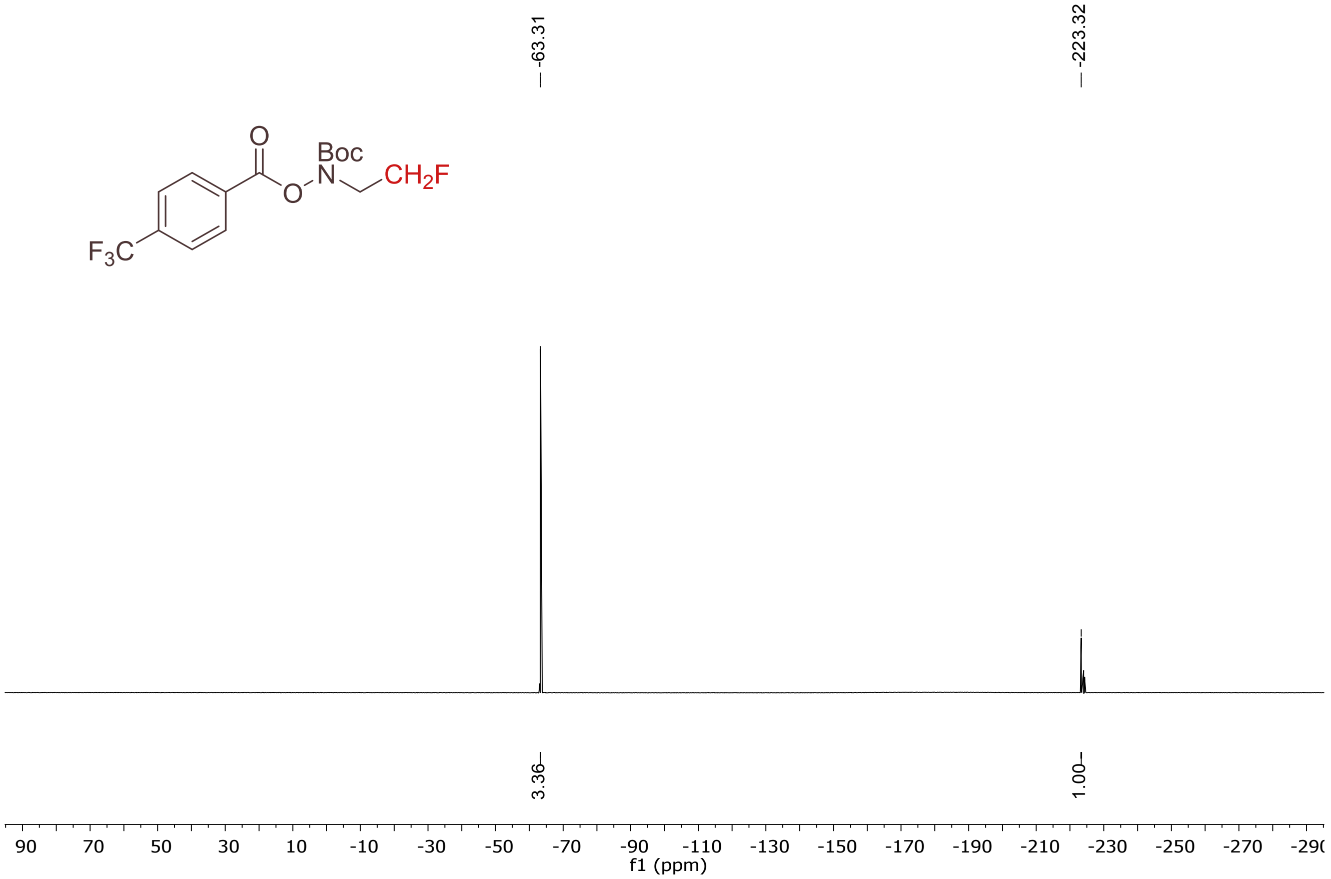

$^{19}F\{^1H\}$ NMR (377 MHz, $CDCl_3$) of compound **2d**.

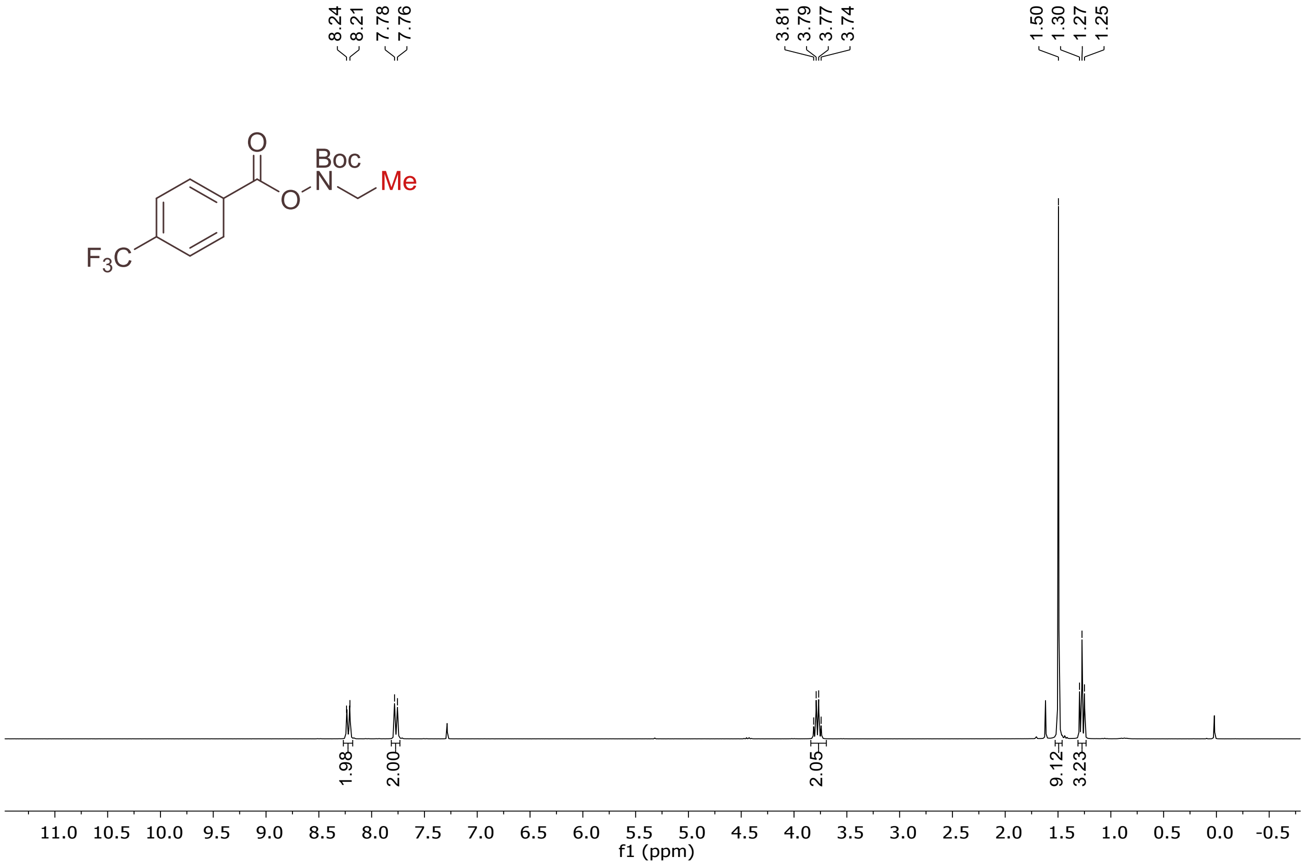

^{1}H NMR (300 MHz, $CDCl_3$) of compound **2e**.

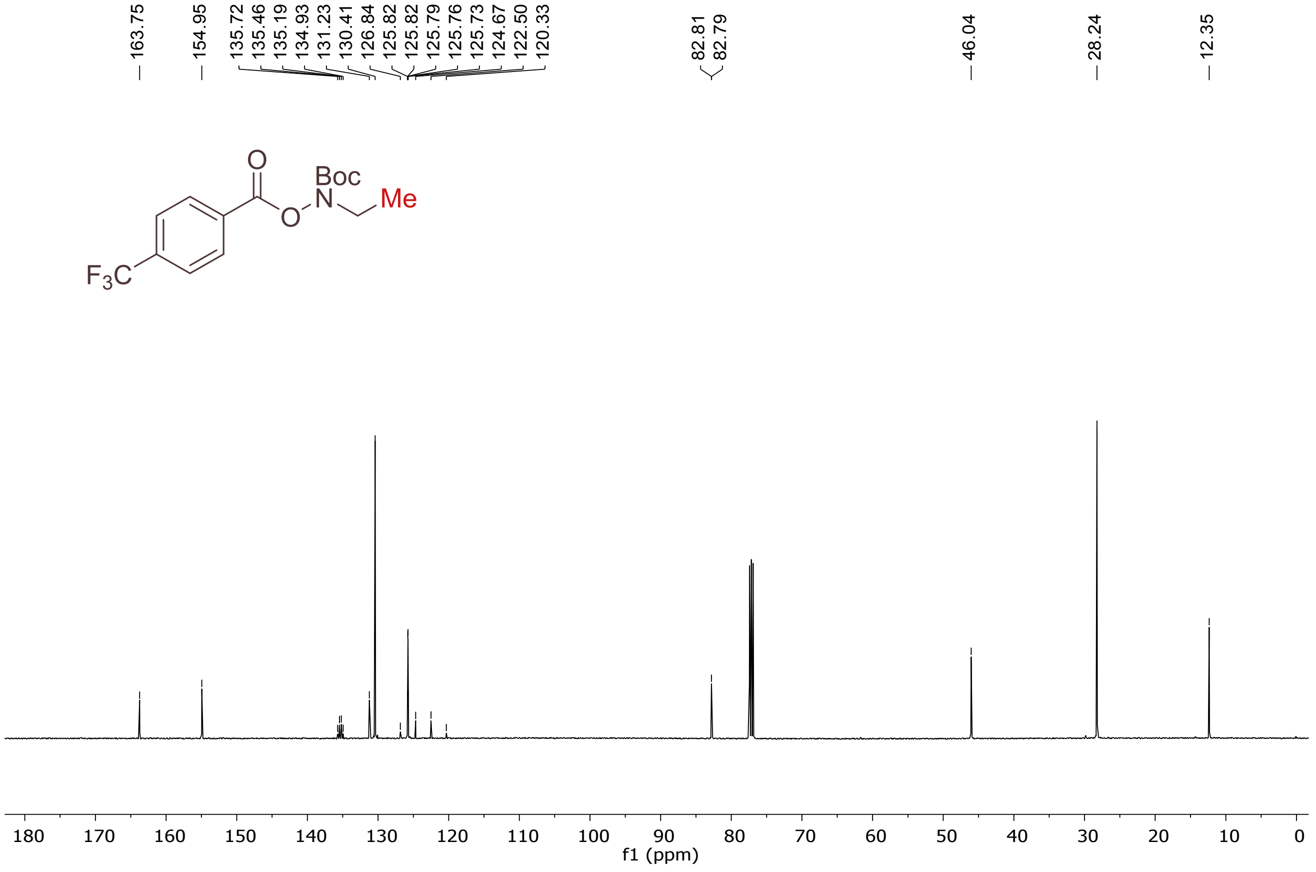

$^{13}C\{^{1}H\}$ NMR (126 MHz, $CDCl_3$) of compound **2e**.

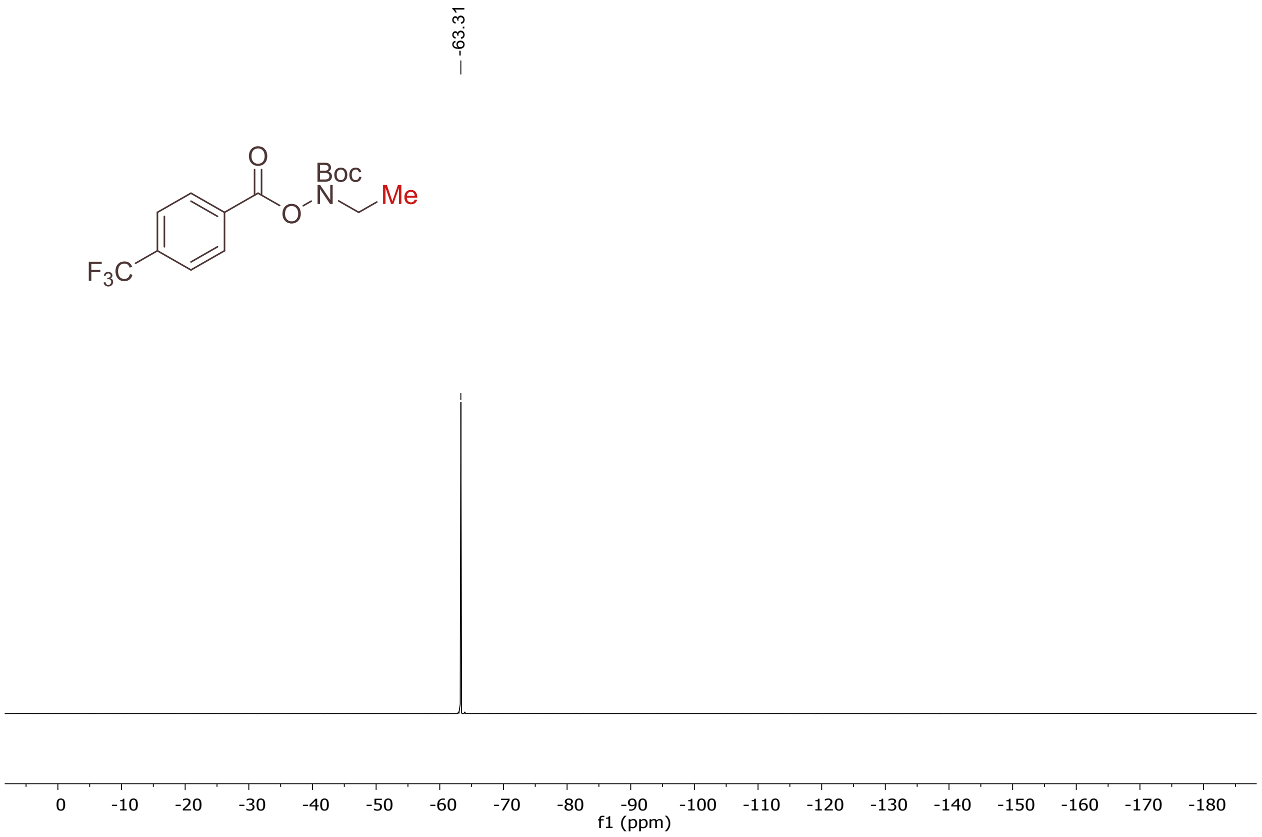

$^{19}F\{^{1}H\}$ NMR (282 MHz, $CDCl_3$) of compound **2e**.

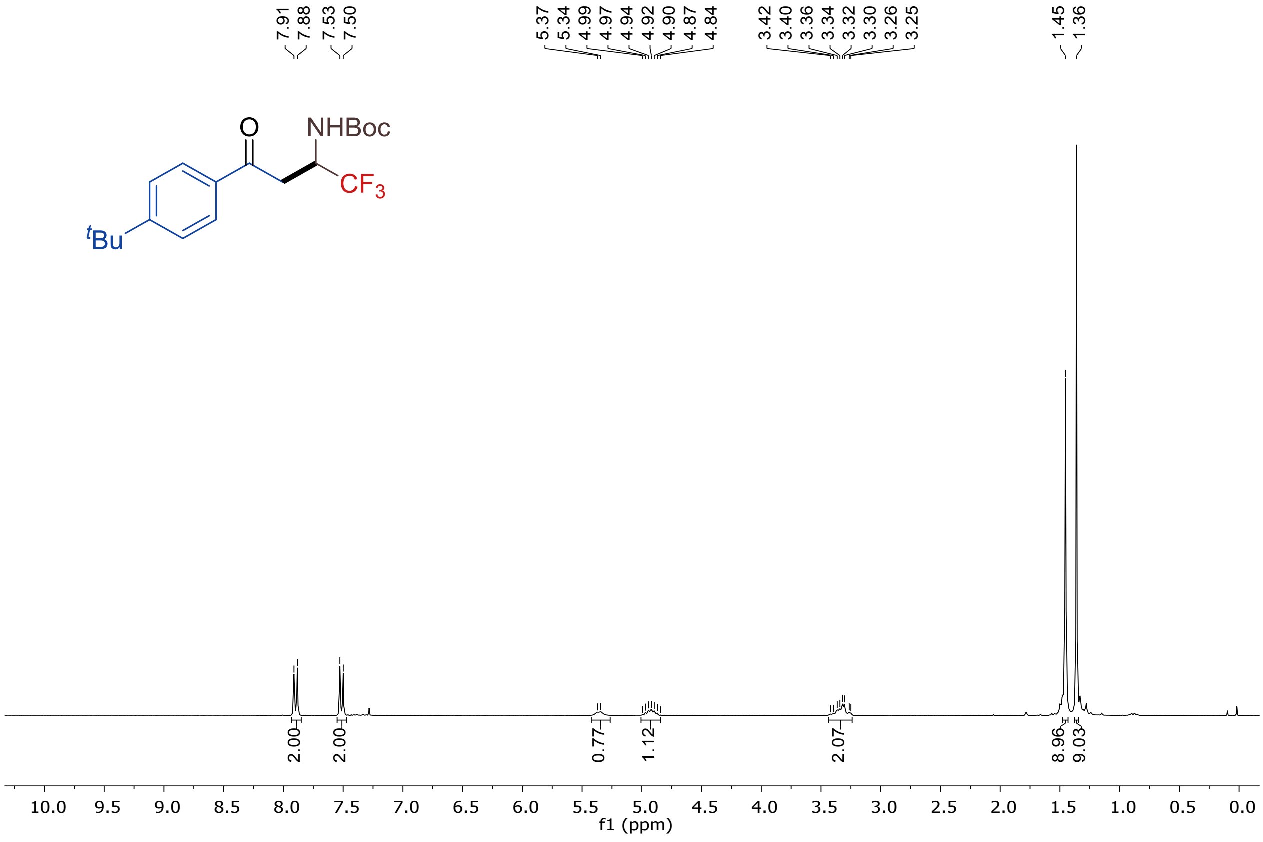

^{1}H NMR (300 MHz, $CDCl_3$) of compound **3**.

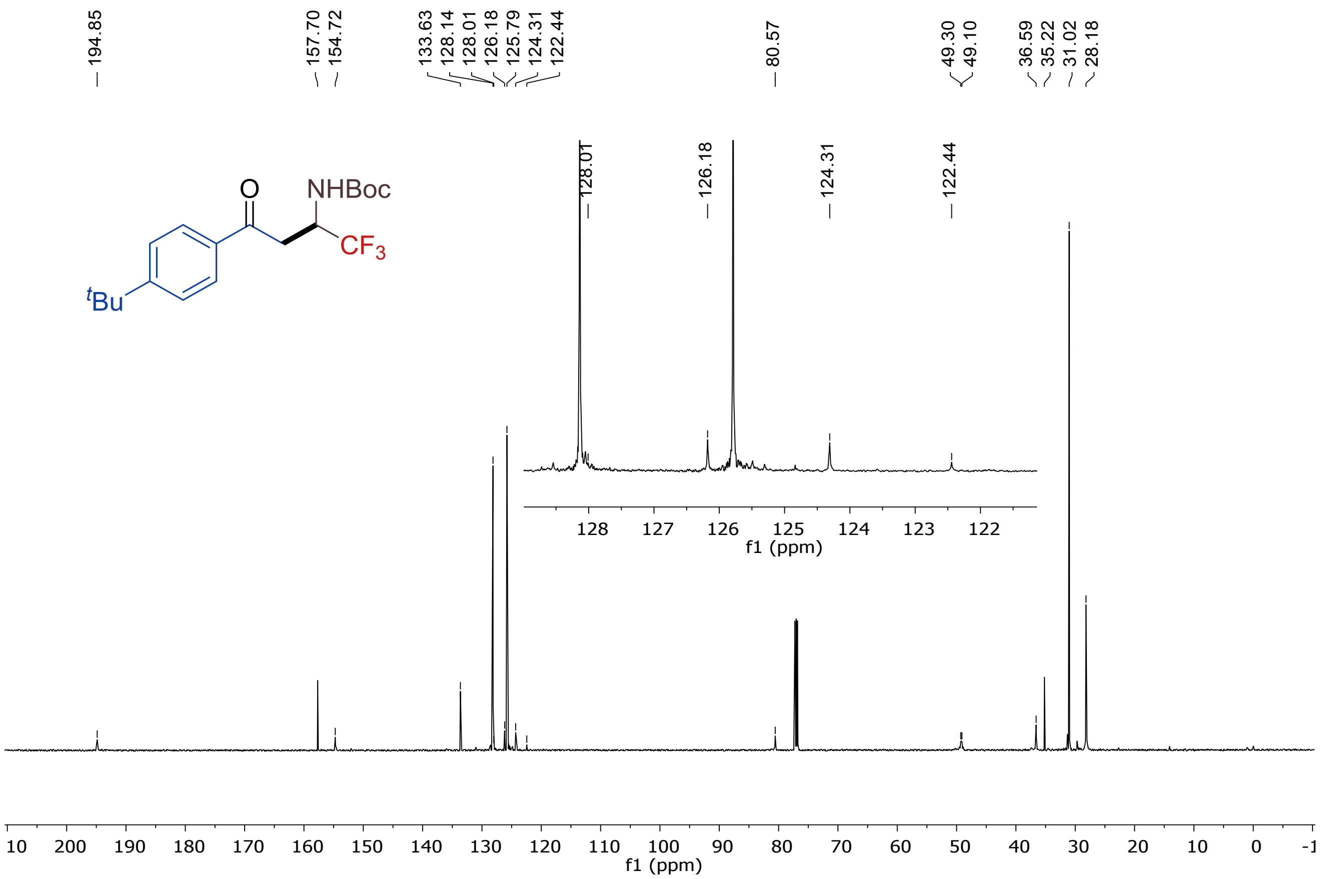

$^{13}C\{^{1}H\}$ NMR (151 MHz, $CDCl_3$) of compound **3**.

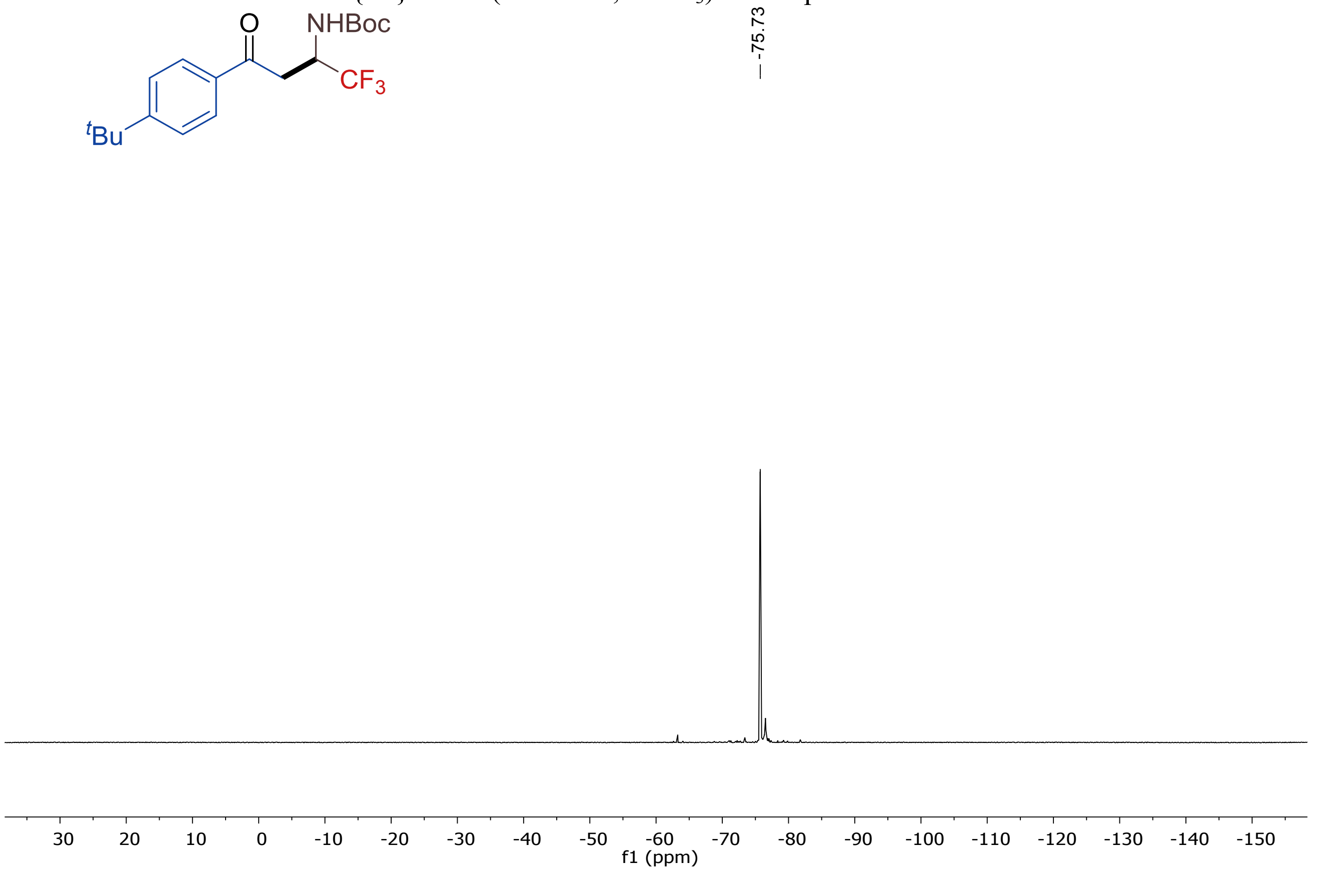

$^{19}F\{^{1}H\}$ NMR (282 MHz, $CDCl_3$) of compound **3**.

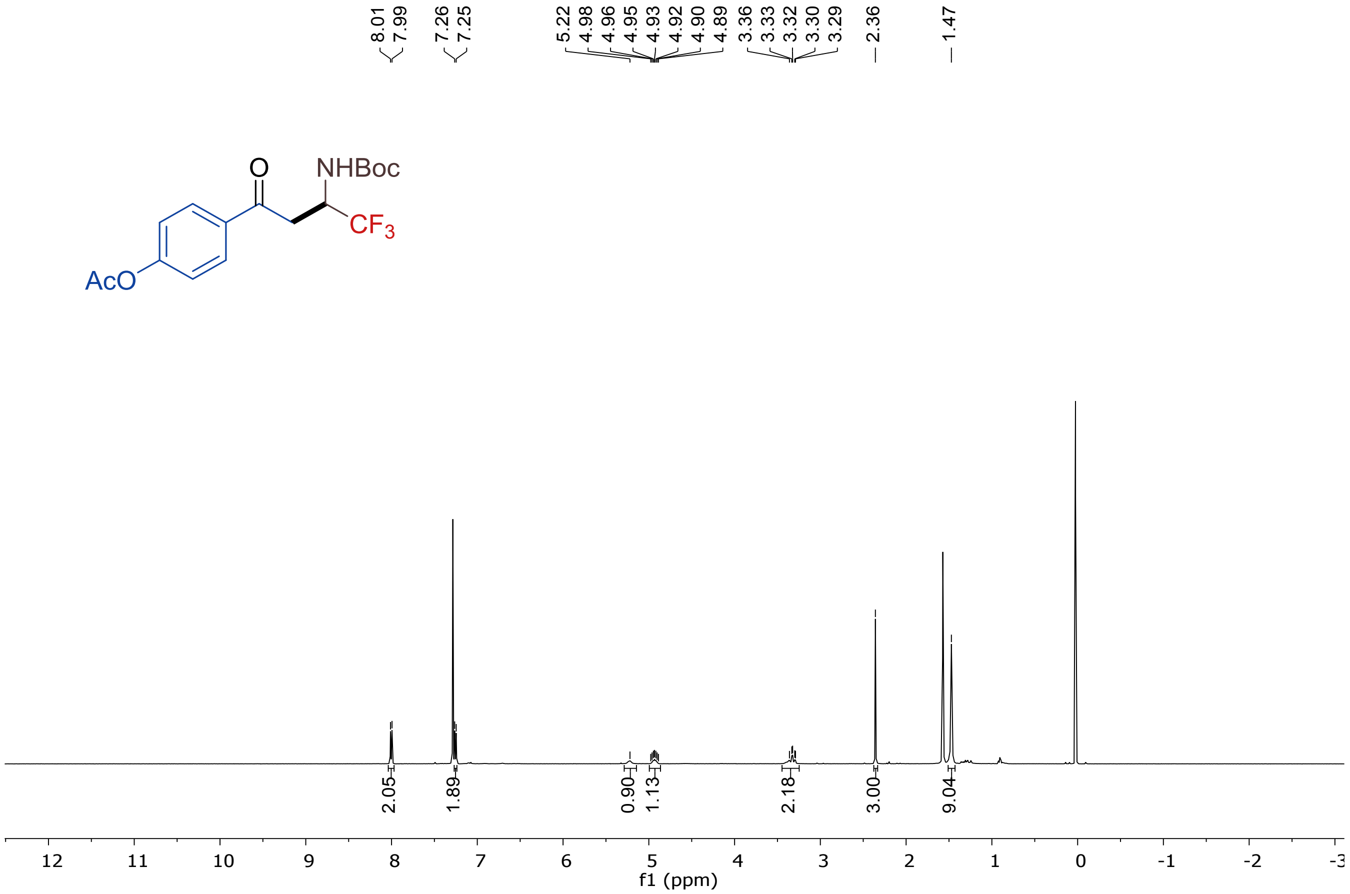

^{1}H NMR (500 MHz, $CDCl_3$) of compound **4**.

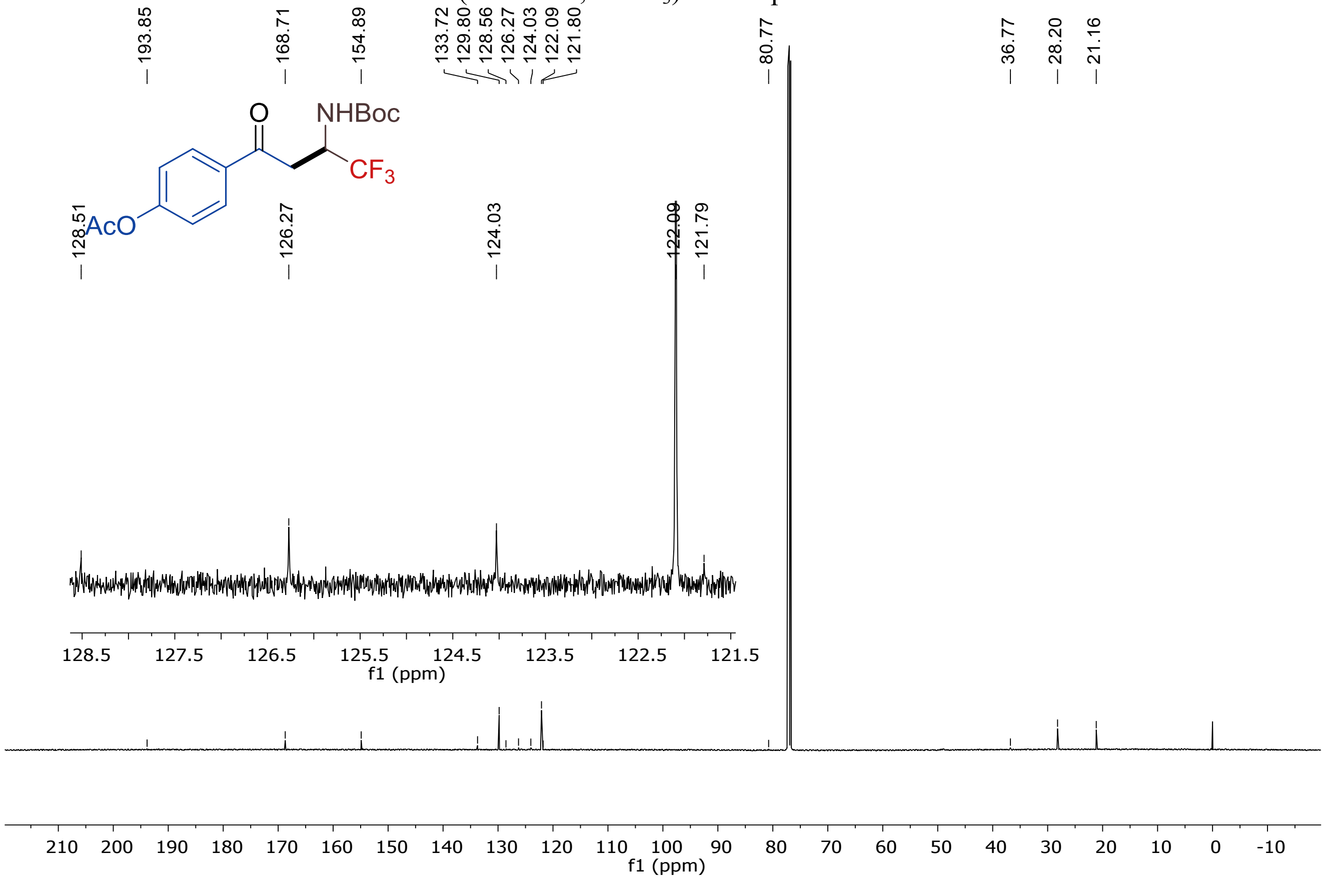

$^{13}C\{^1H\}$ NMR (126 MHz, $CDCl_3$) of compound **4**.

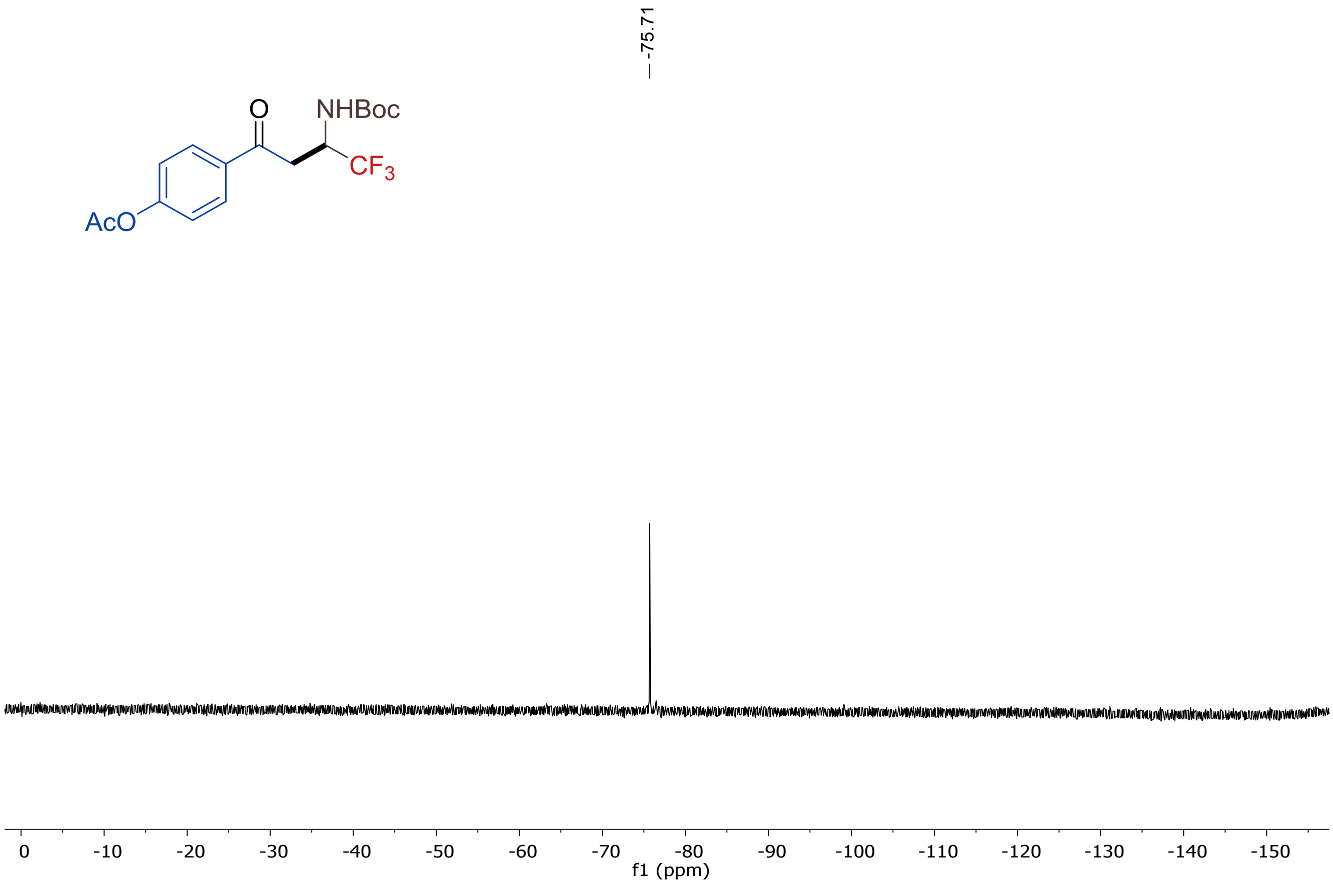

$^{19}F\{^{1}H\}$ NMR (282 MHz, $CDCl_3$) of compound **4**.

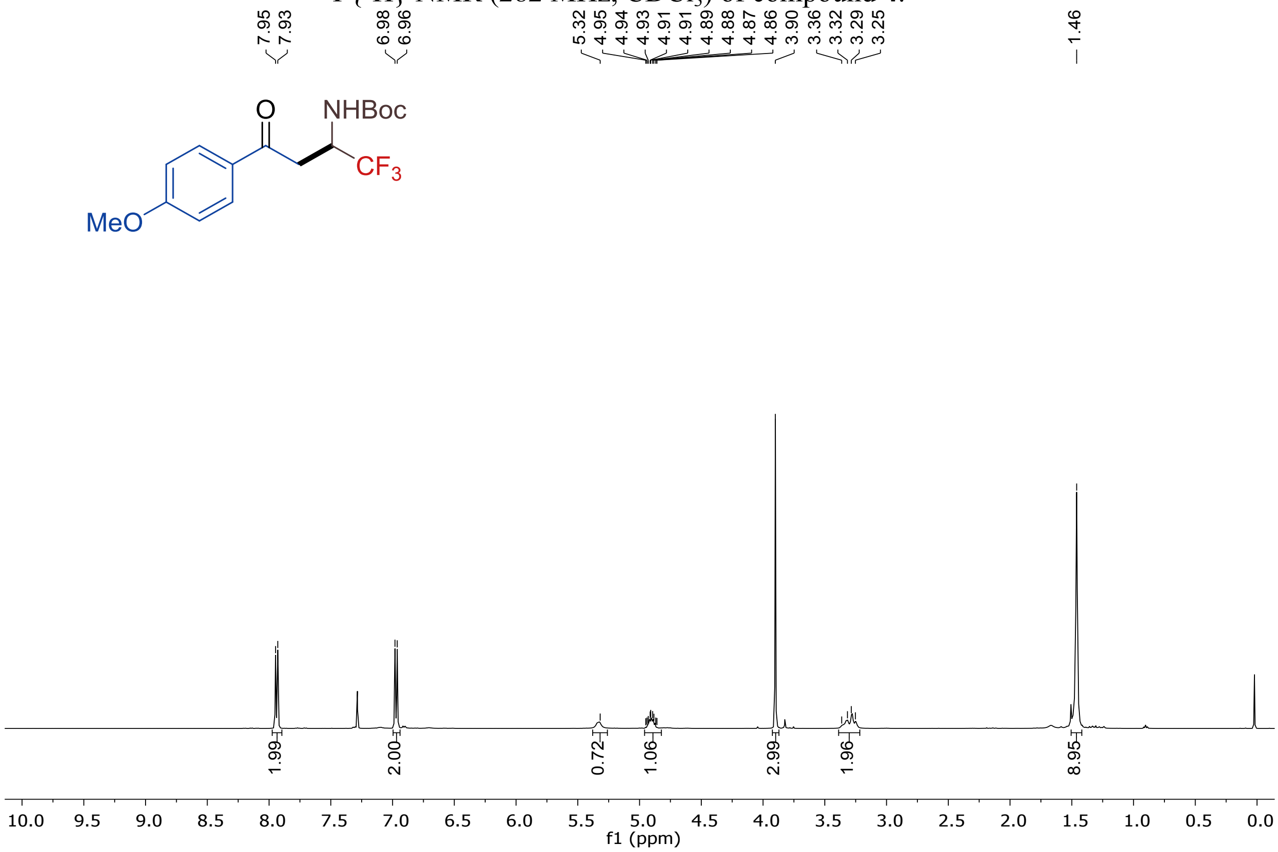

^{1}H NMR (500 MHz, $CDCl_3$) of compound **5**.

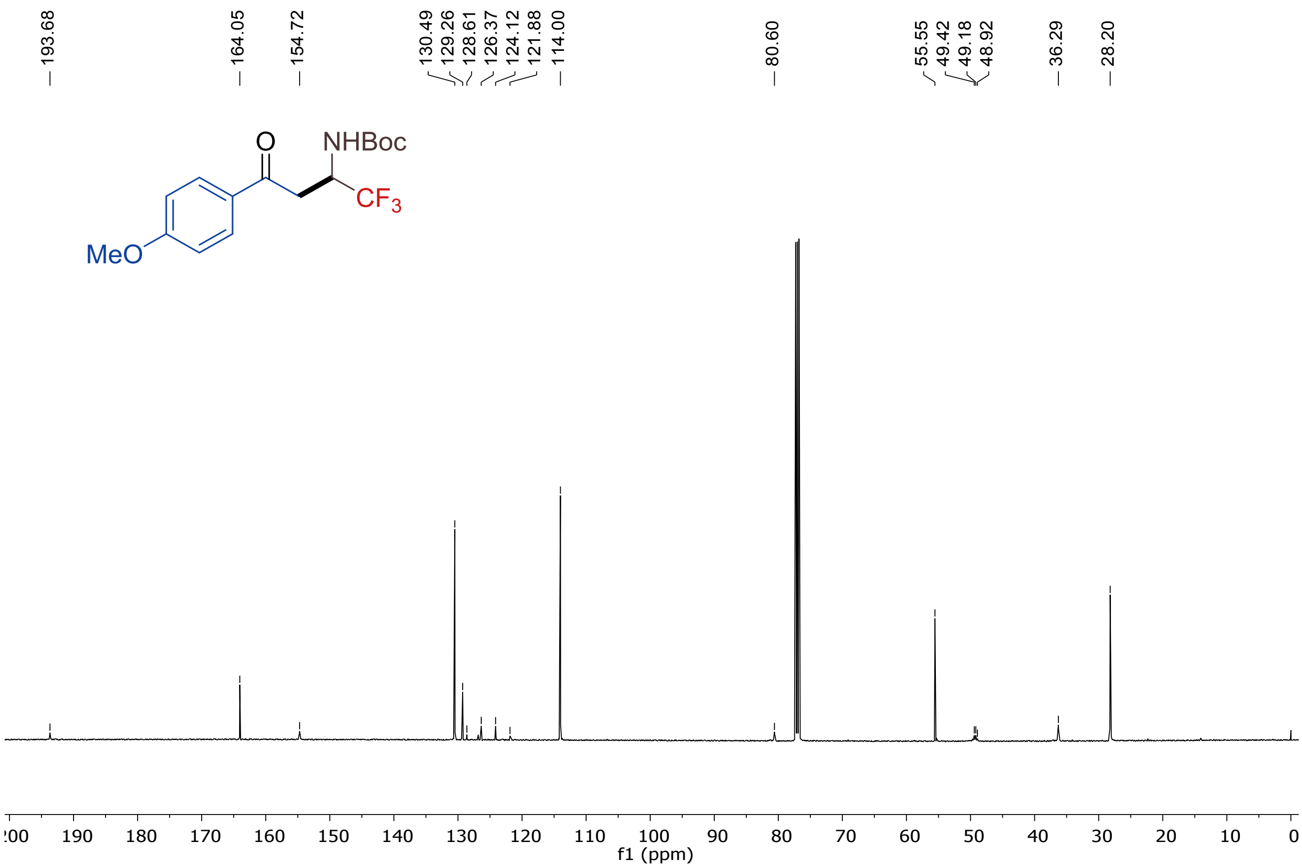

$^{13}C\{^1H\}$ NMR (126 MHz, $CDCl_3$) of compound **5**.

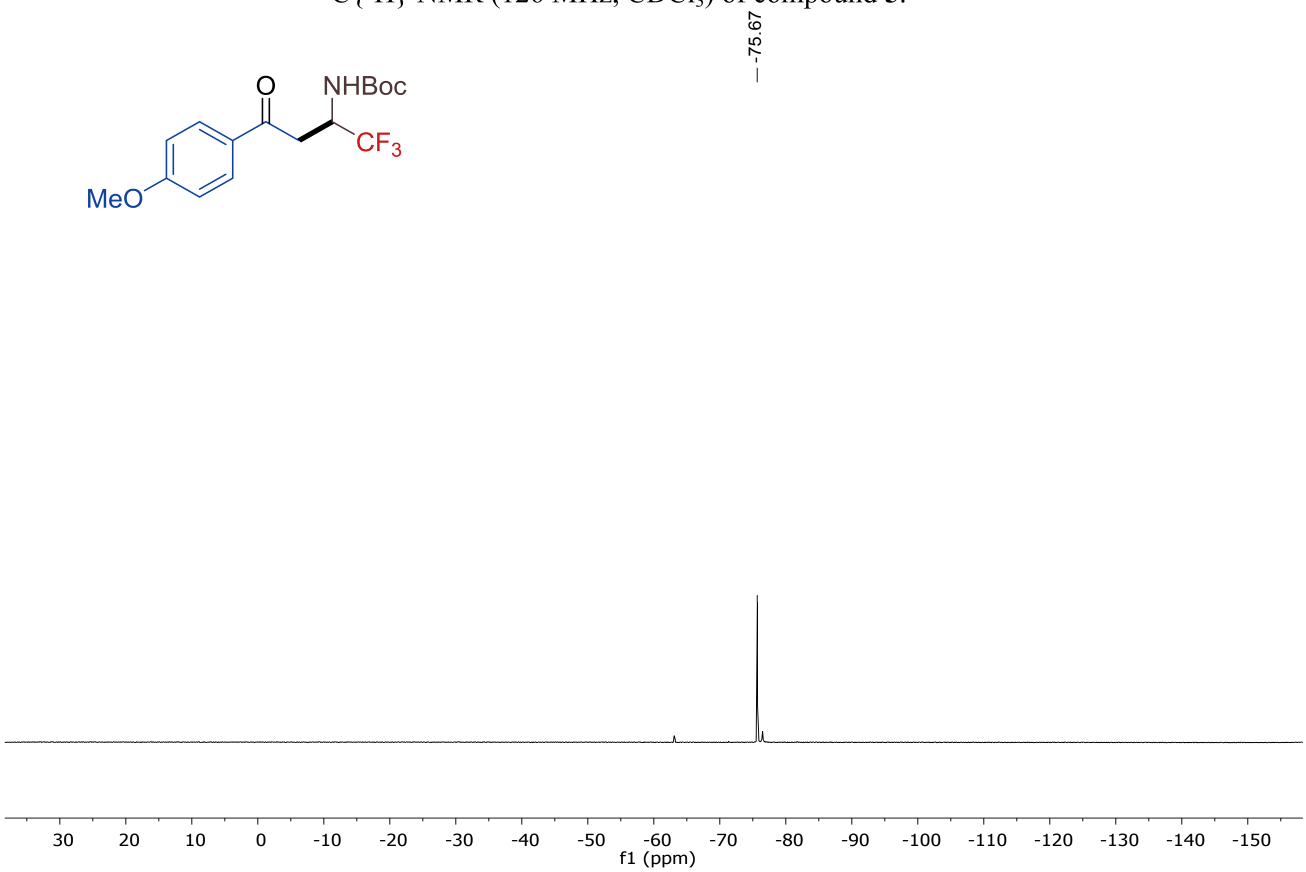

$^{19}F\{^1H\}$ NMR (282 MHz, $CDCl_3$) of compound **5**.

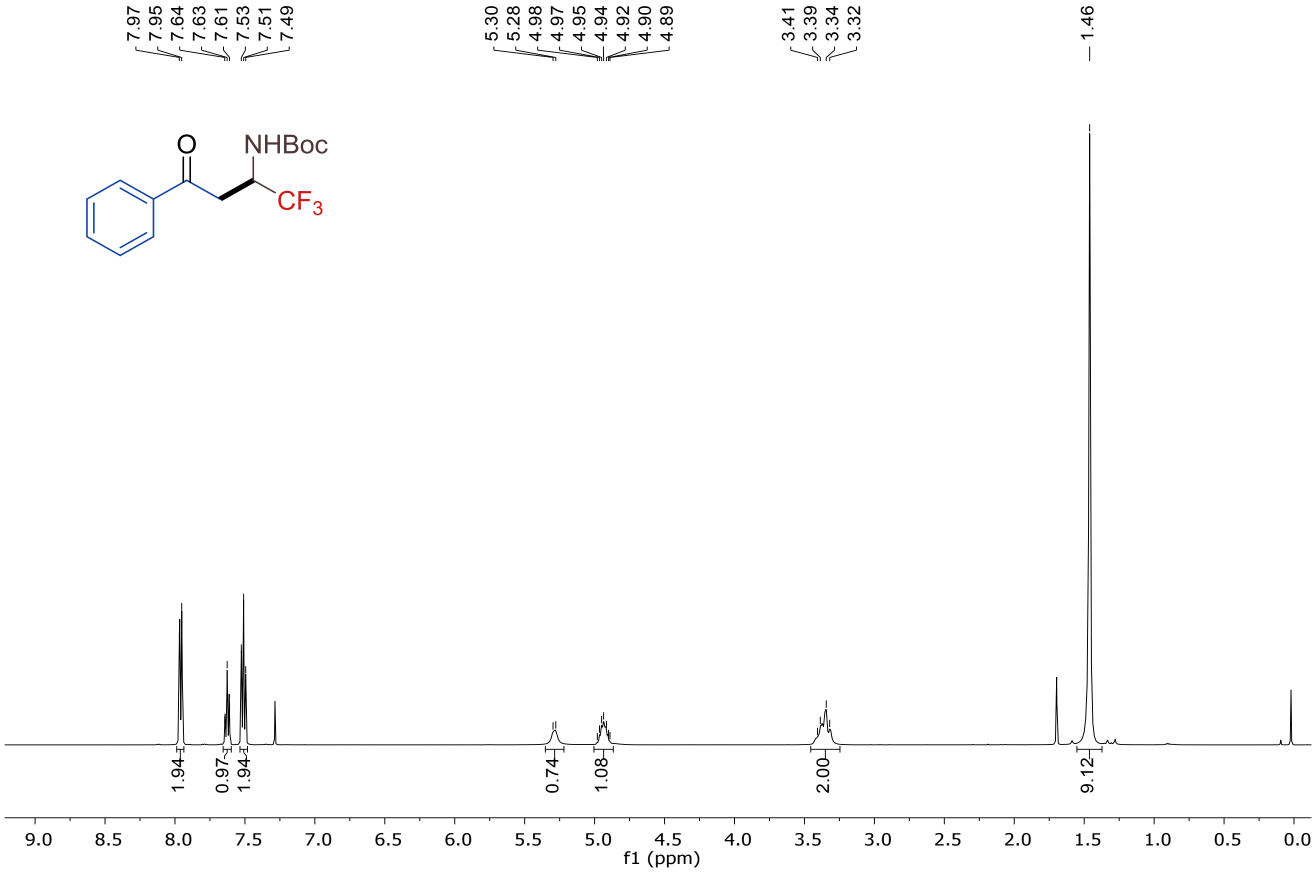

^{1}H NMR (500 MHz, $CDCl_3$) of compound **6**.

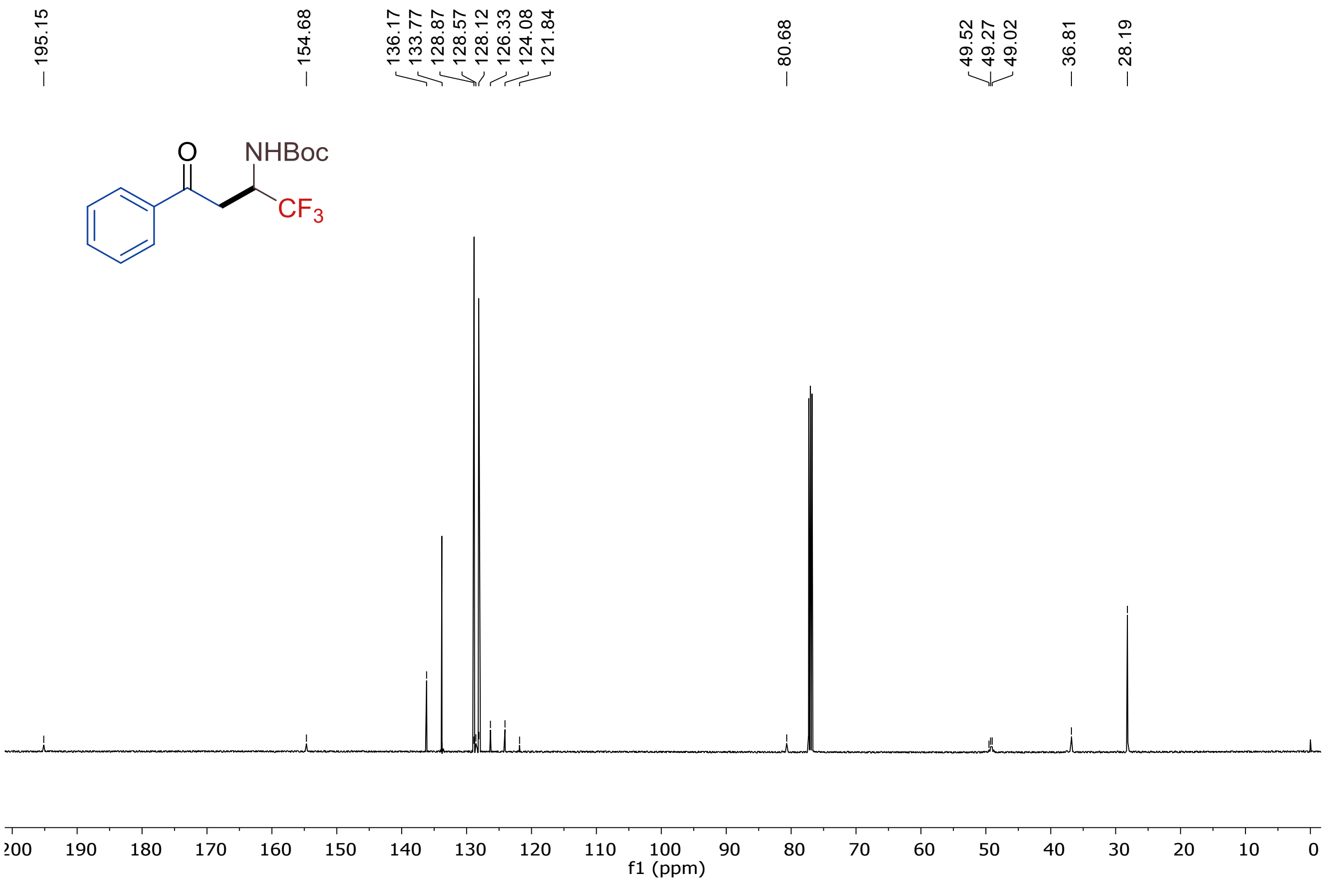

^{13}C{^{1}H} NMR (151 MHz, $CDCl_3$) of compound **6**.

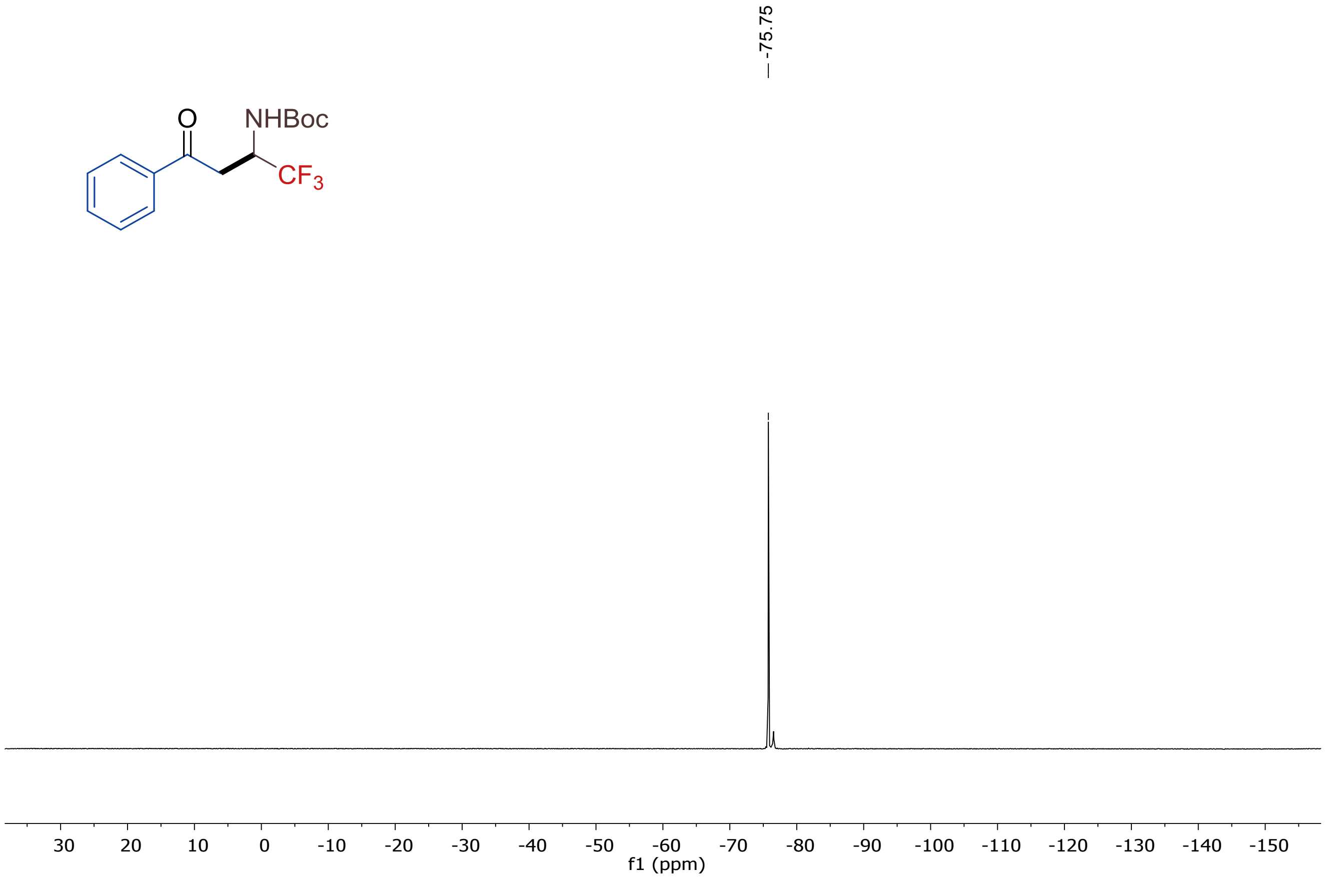

$^{19}F\{^1H\}$ NMR (282 MHz, $CDCl_3$) of compound **6**.

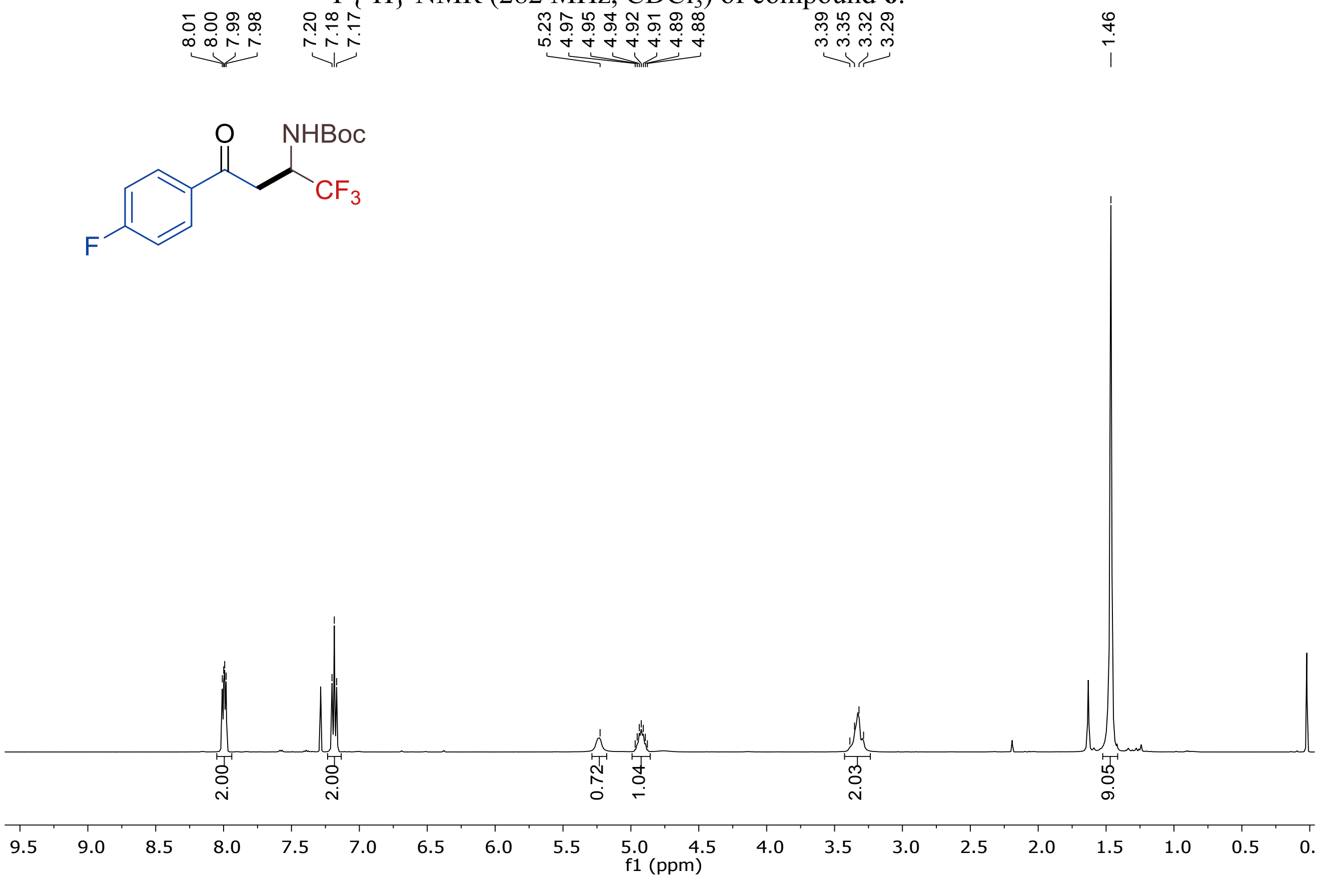

^1H NMR (500 MHz, $CDCl_3$) of compound **7**.

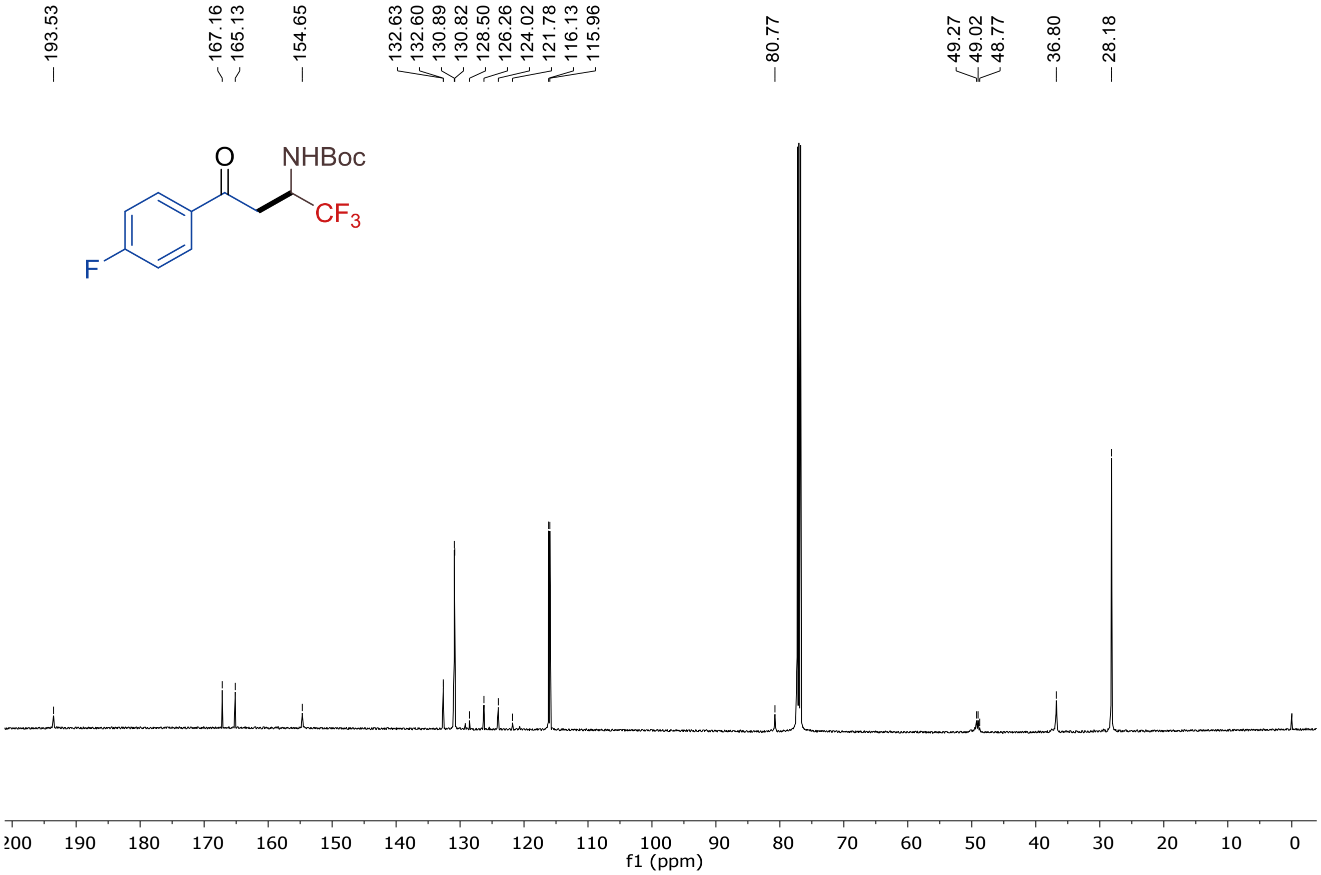

$^{13}C\{^{1}H\}$ NMR (126 MHz, $CDCl_3$) of compound **7**.

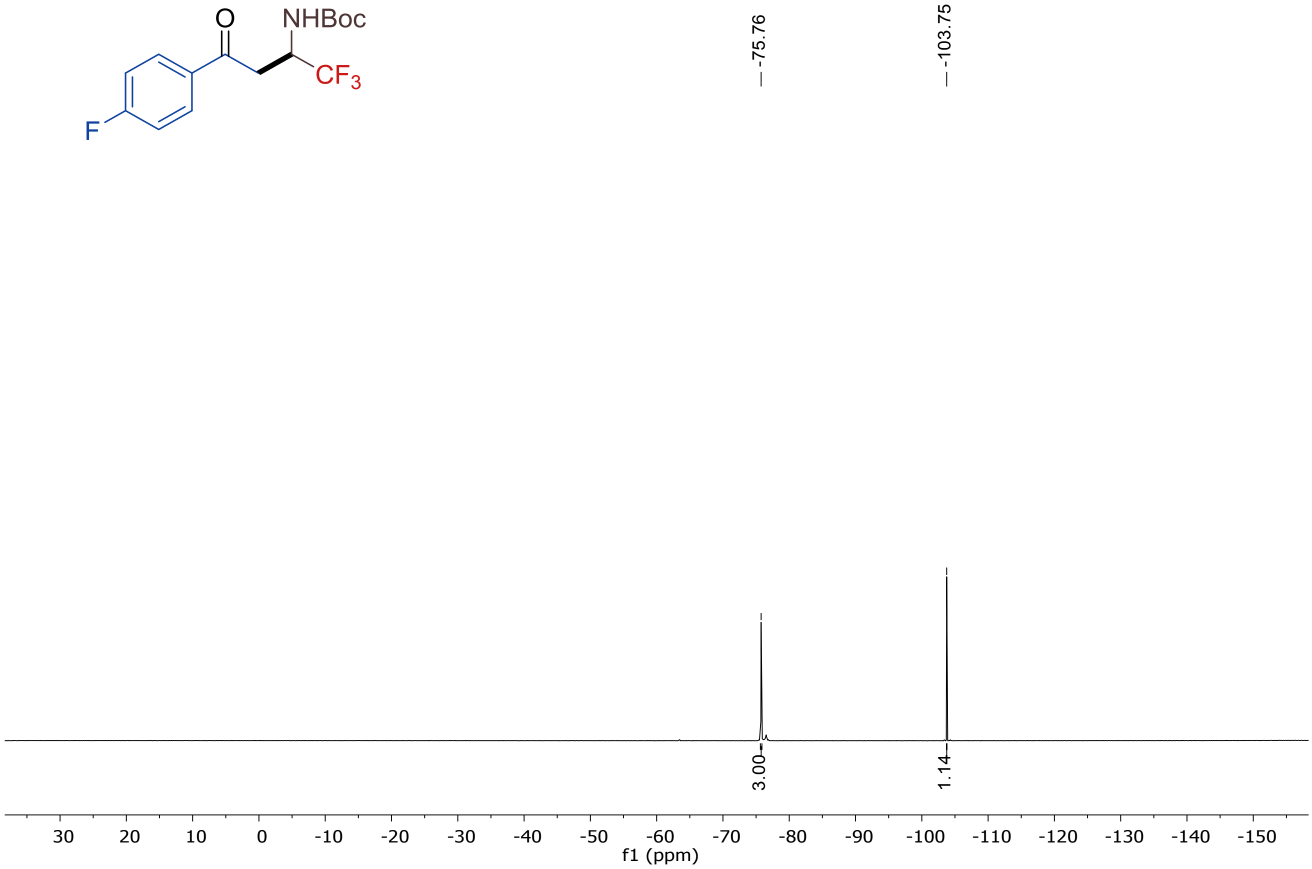

$^{19}F\{^{1}H\}$ NMR (282 MHz, $CDCl_3$) of compound **7**.

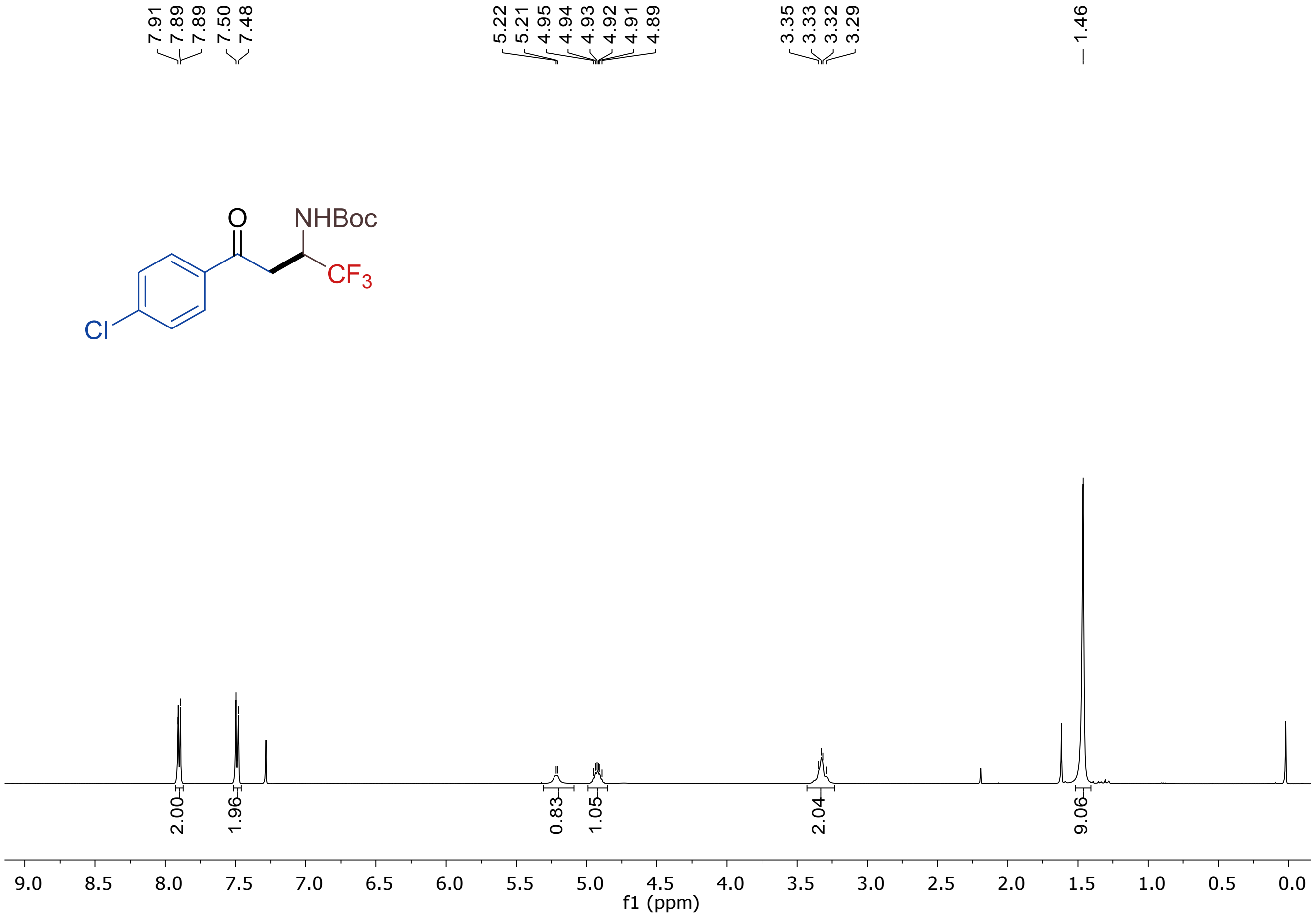

^{1}H NMR (500 MHz, $CDCl_3$) of compound **8**.

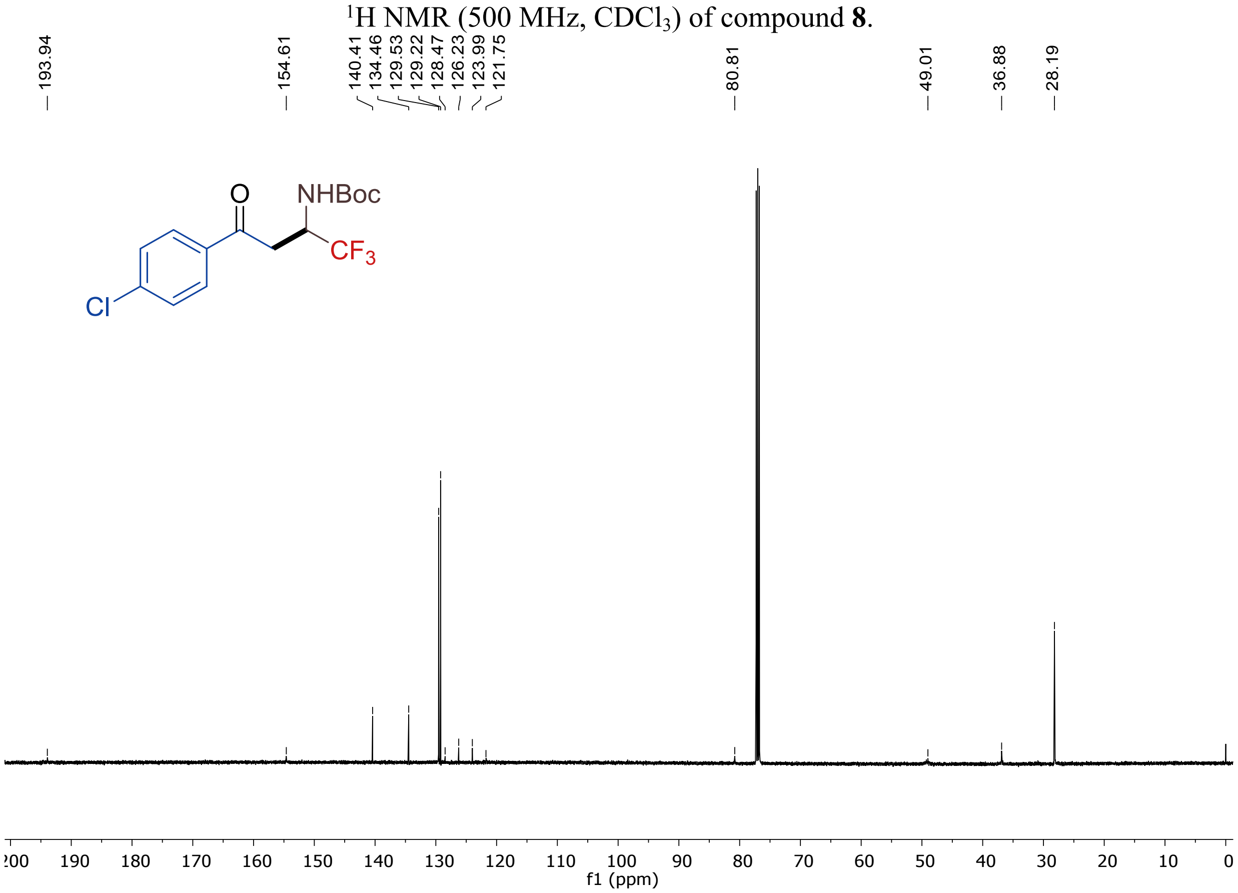

^{13}C{^{1}H} NMR (126 MHz, $CDCl_3$) of compound **8**.

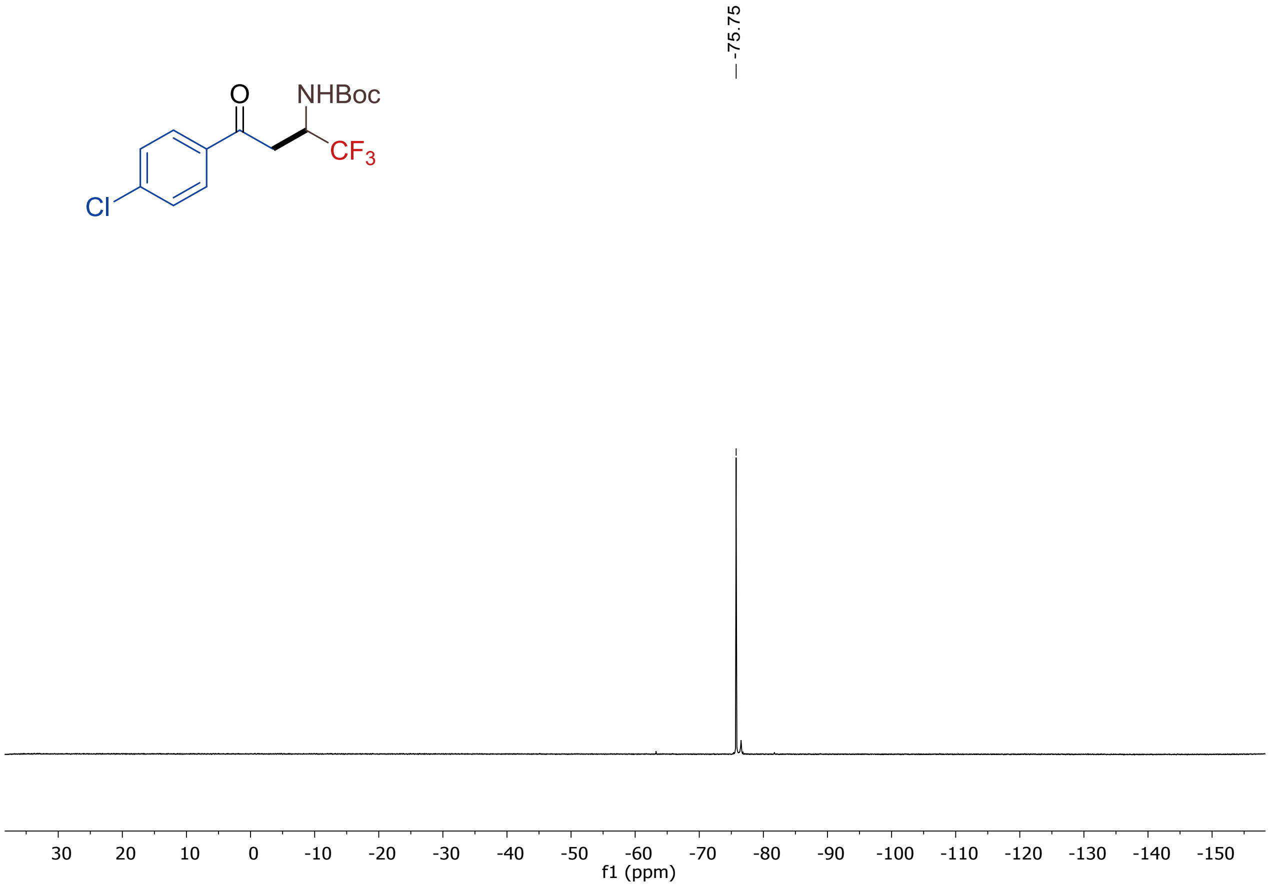

$^{19}F\{^{1}H\}$ NMR (282 MHz, $CDCl_3$) of compound **8**.

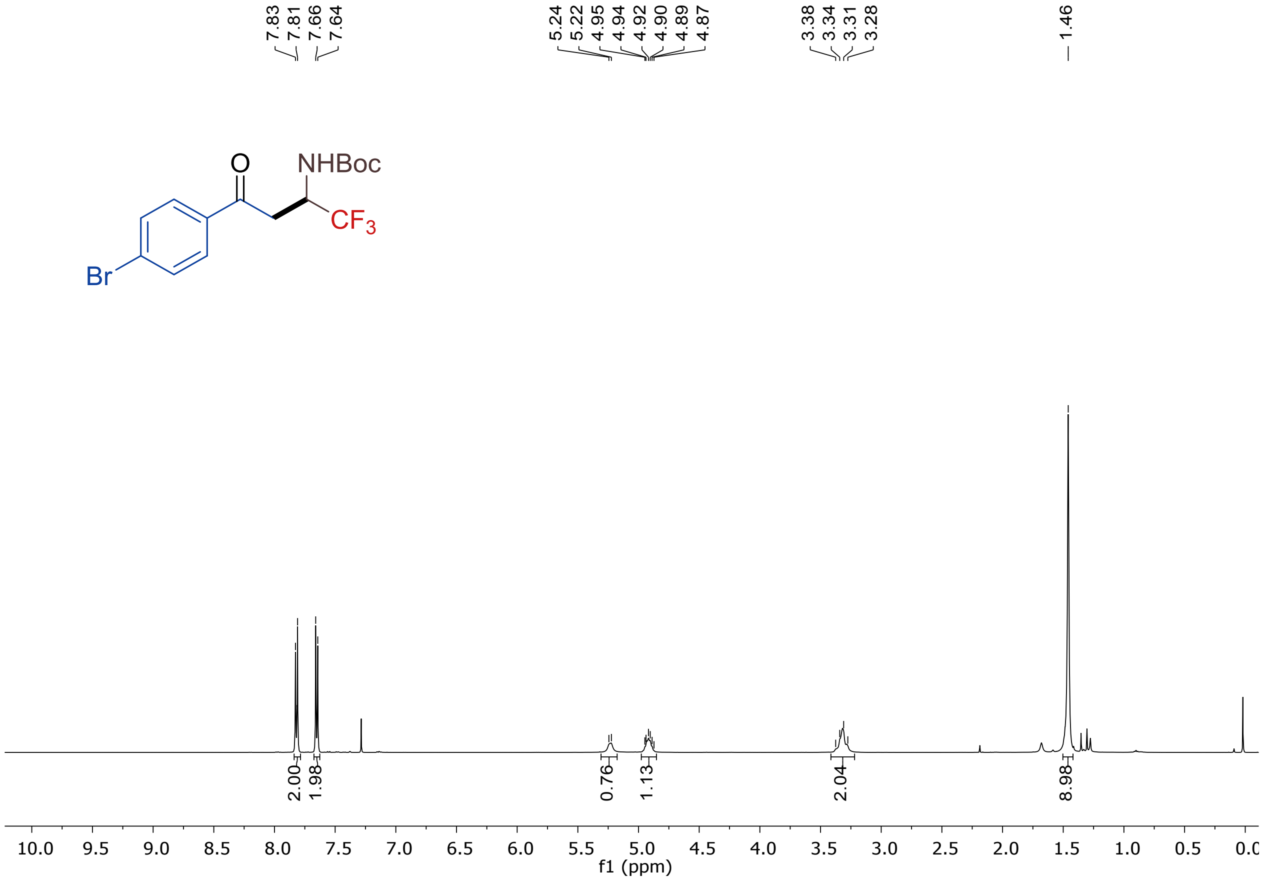

^{1}H NMR (500 MHz, $CDCl_3$) of compound **9**.

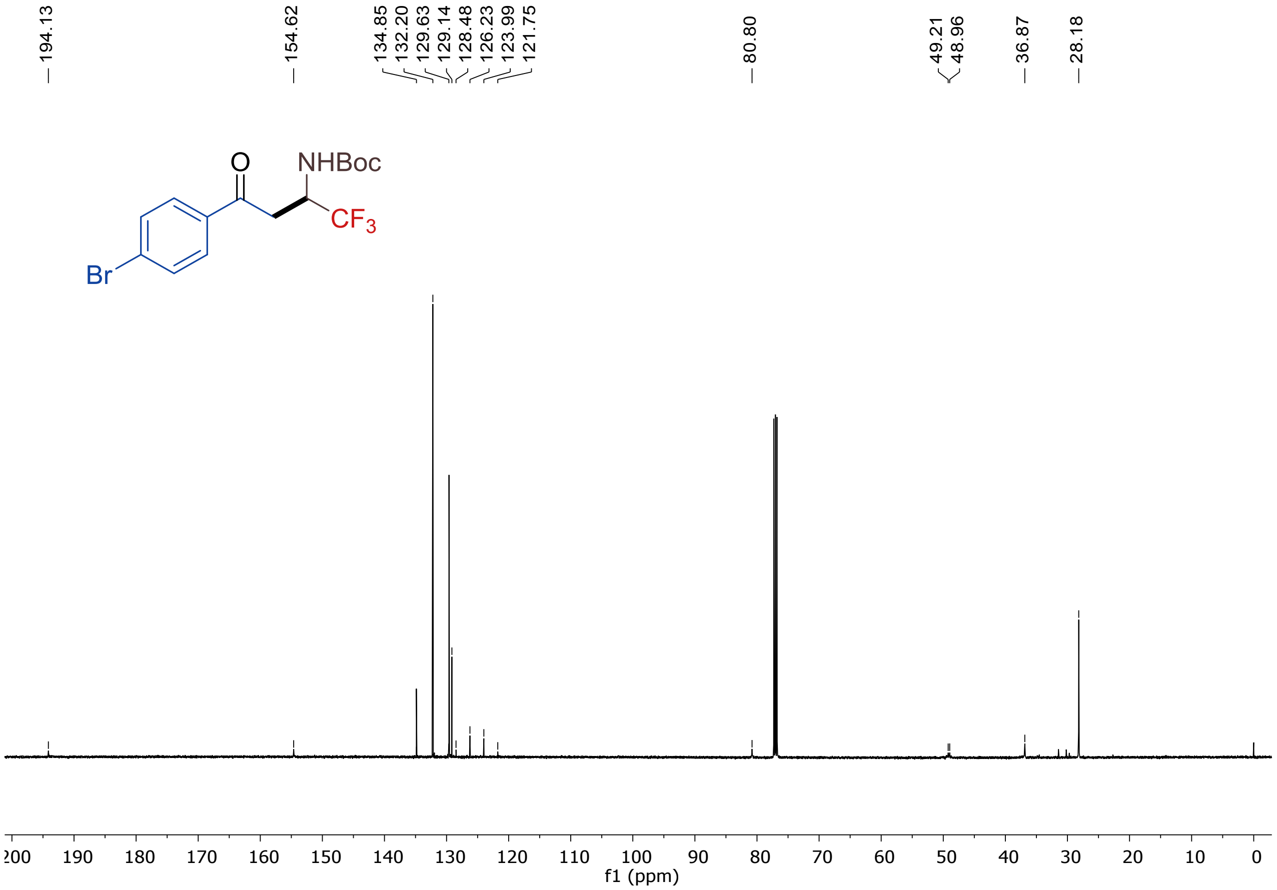

$^{13}C\{^{1}H\}$ NMR (126 MHz, $CDCl_3$) of compound **9**.

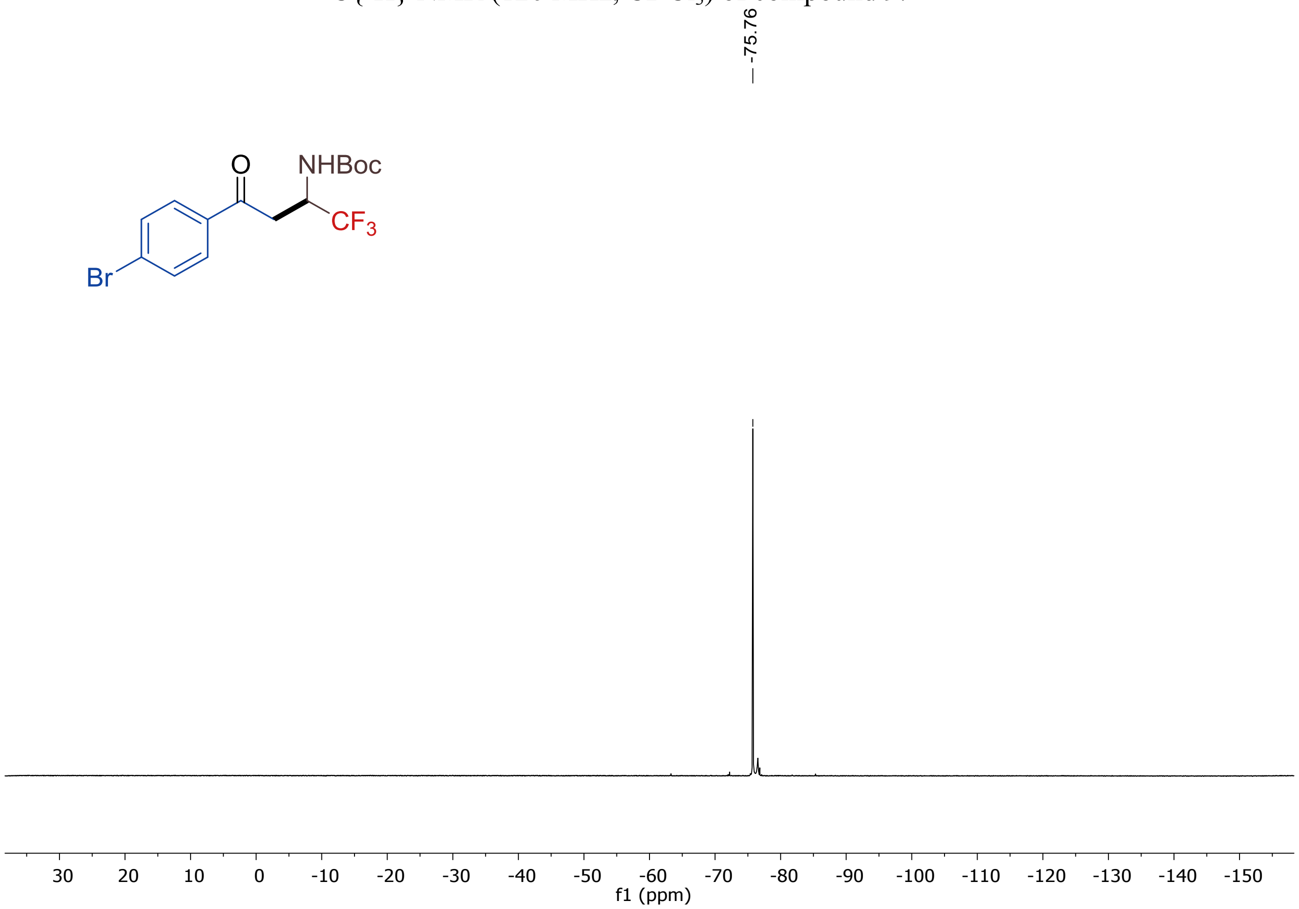

$^{19}F\{^{1}H\}$ NMR (282 MHz, $CDCl_3$) of compound **9**.

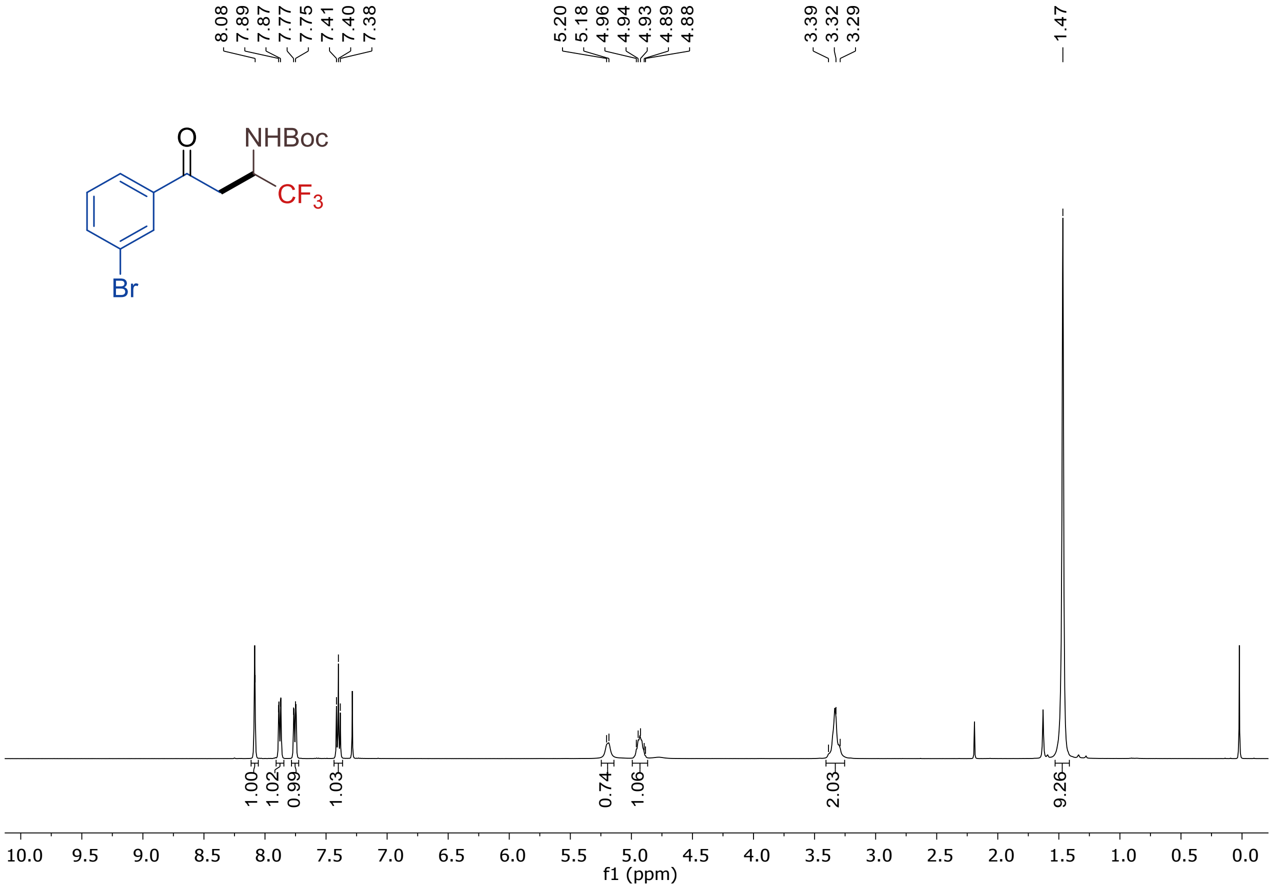

^{1}H NMR (500 MHz, $CDCl_3$) of compound **10**.

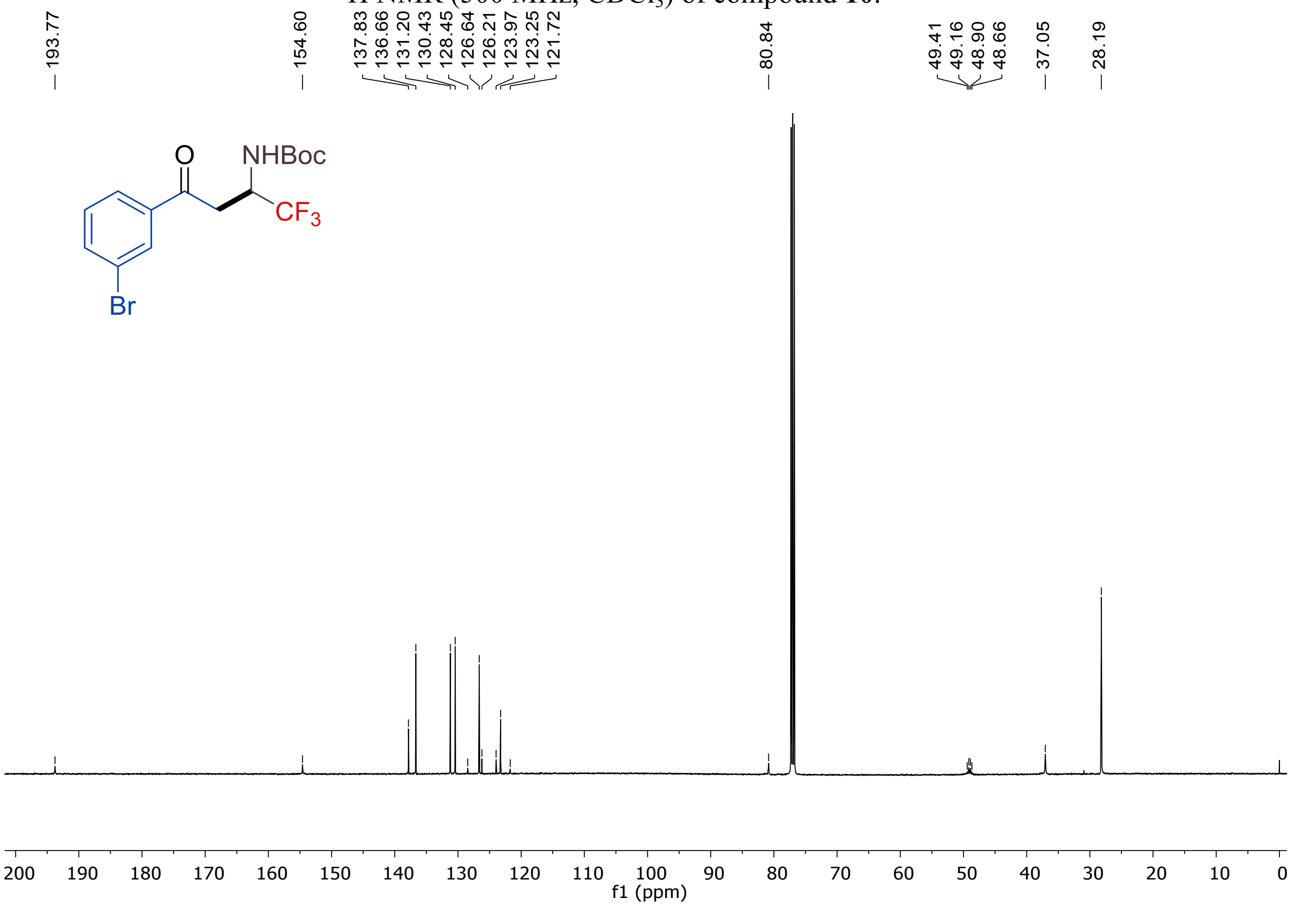

^{13}C{^{1}H} NMR (126 MHz, $CDCl_3$) of compound **10**.

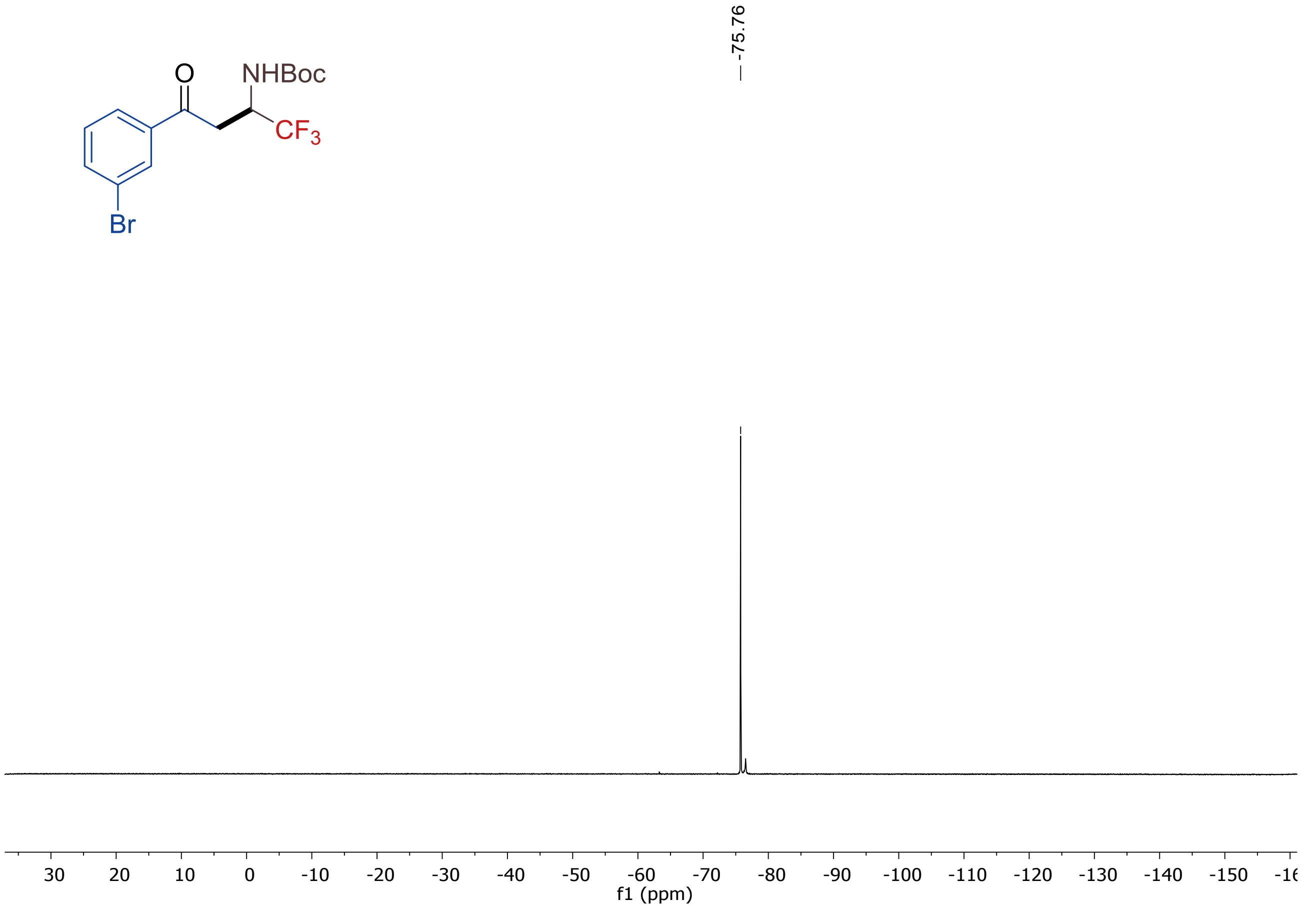

$^{19}F\{^{1}H\}$ NMR (377 MHz, $CDCl_3$) of compound **10**.

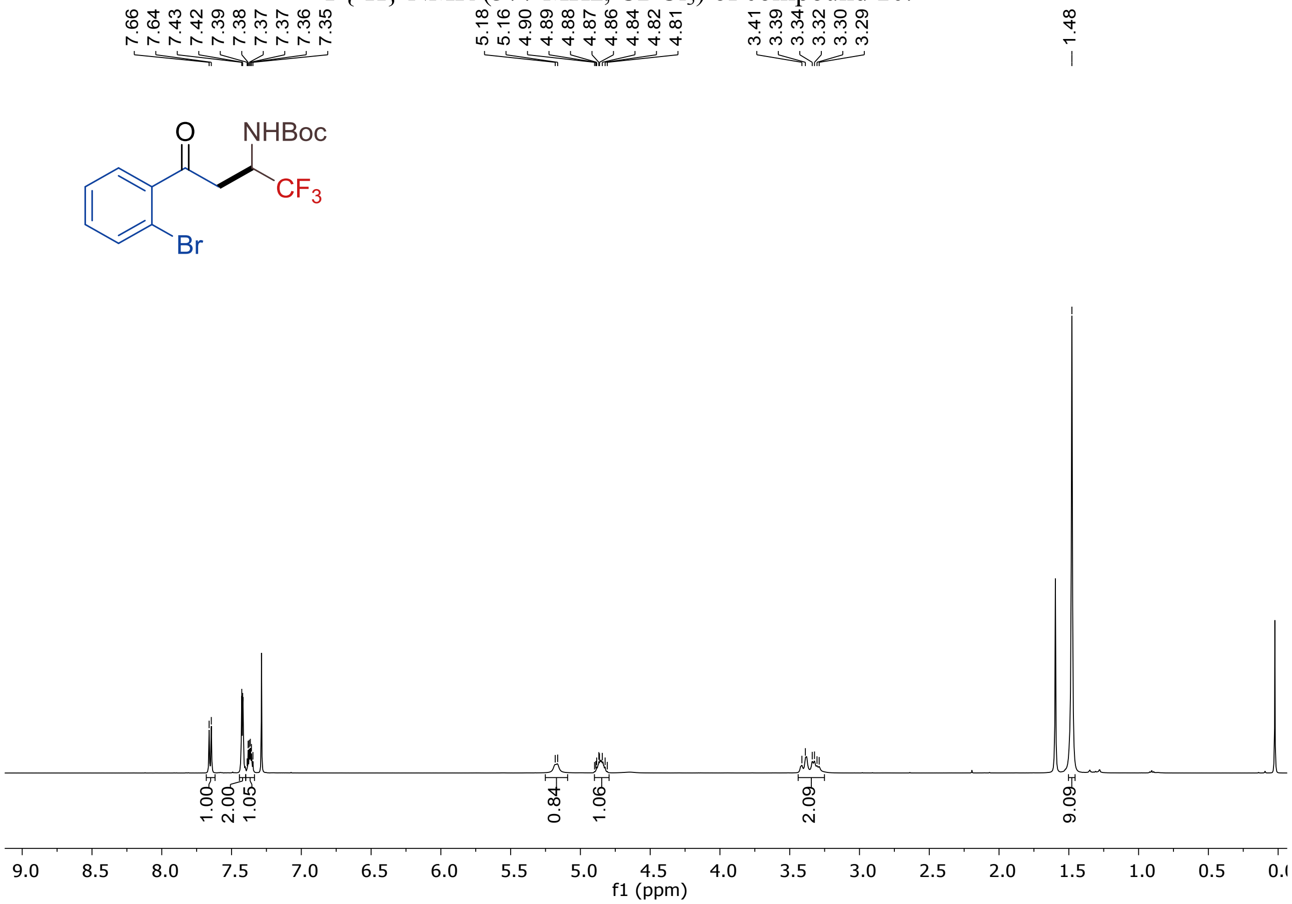

^{1}H NMR (500 MHz, $CDCl_3$) of compound **11**.

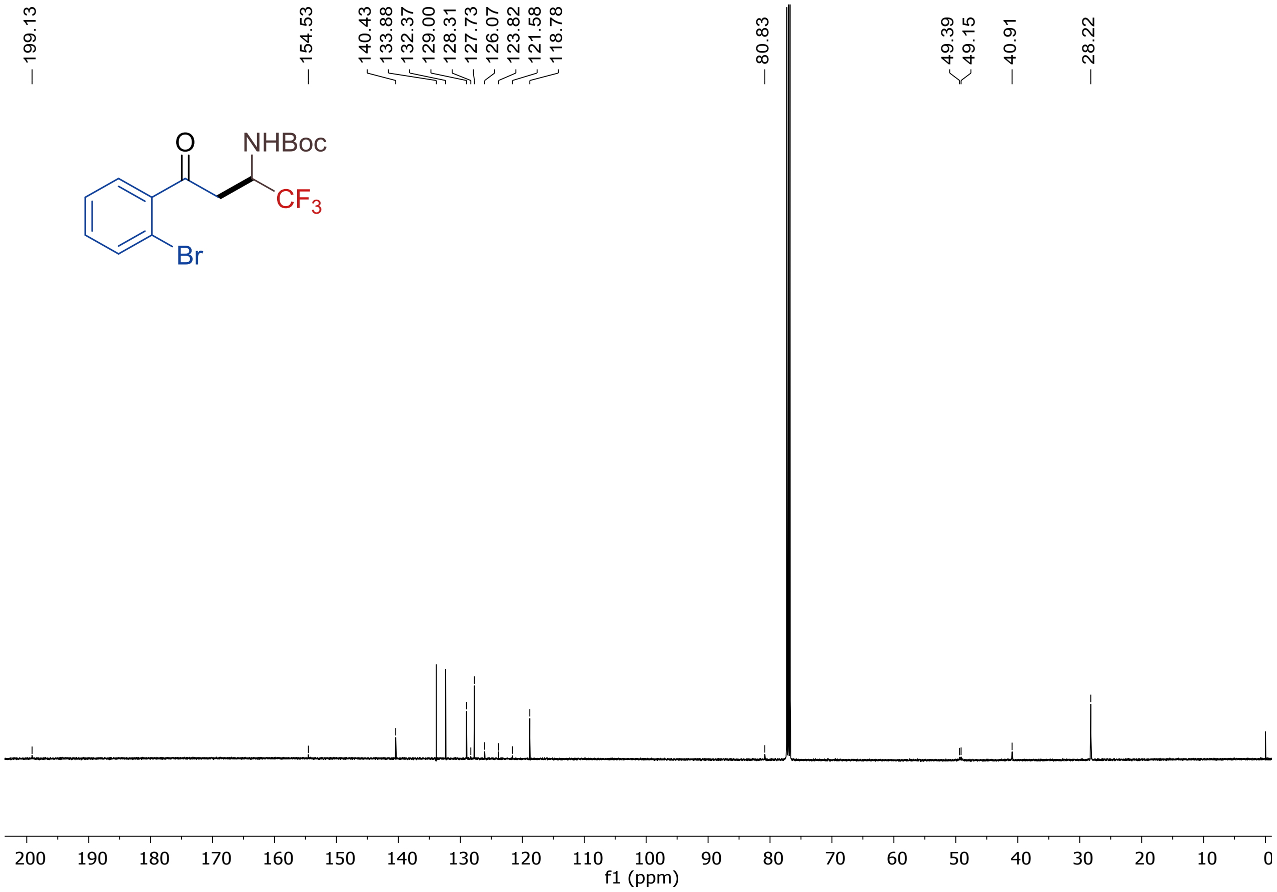

$^{13}C\{^1H\}$ NMR (126 MHz, $CDCl_3$) of compound **11**.

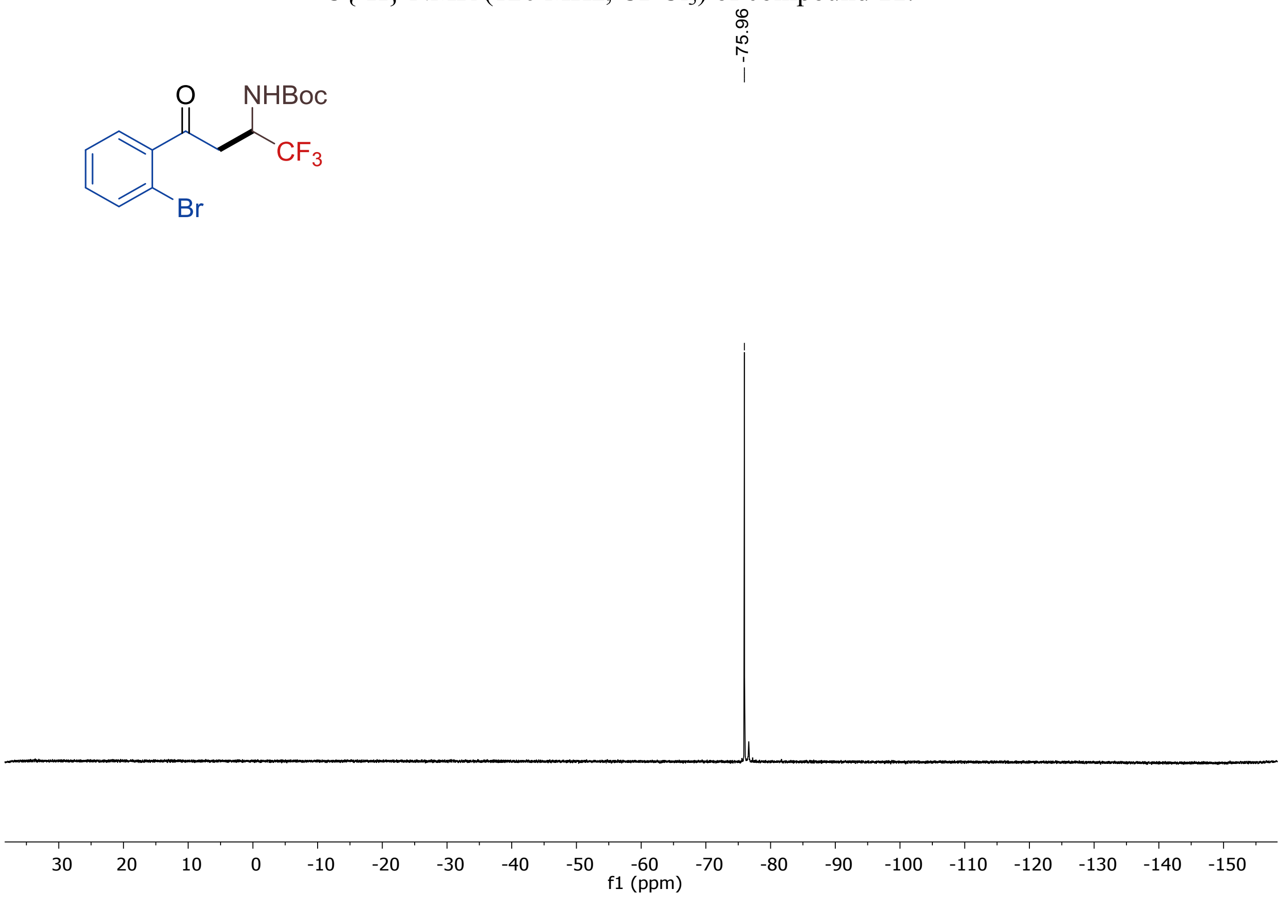

$^{19}F\{^1H\}$ NMR (282 MHz, $CDCl_3$) of compound **11**.

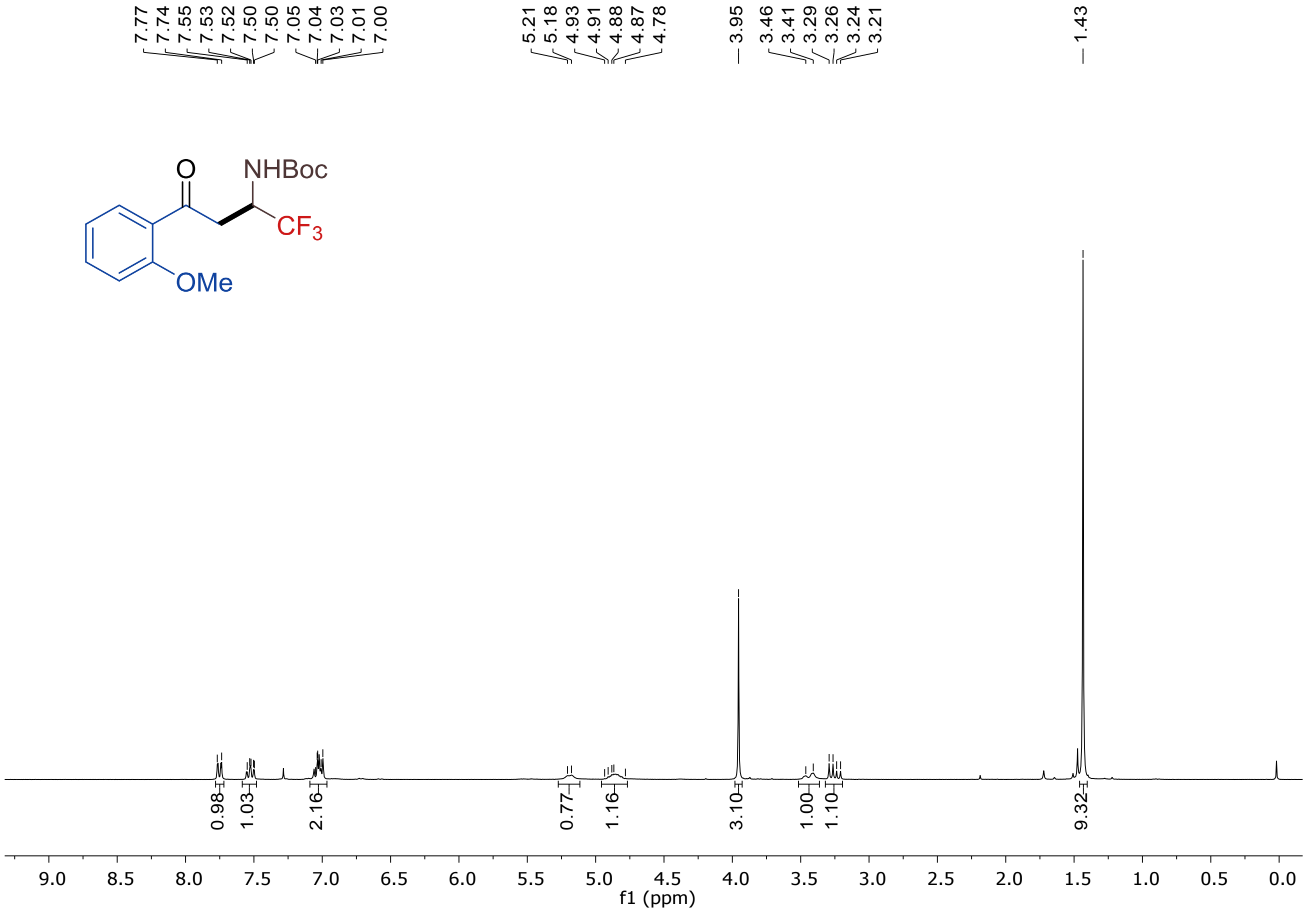

^{1}H NMR (300 MHz, $CDCl_3$) of compound **12**.

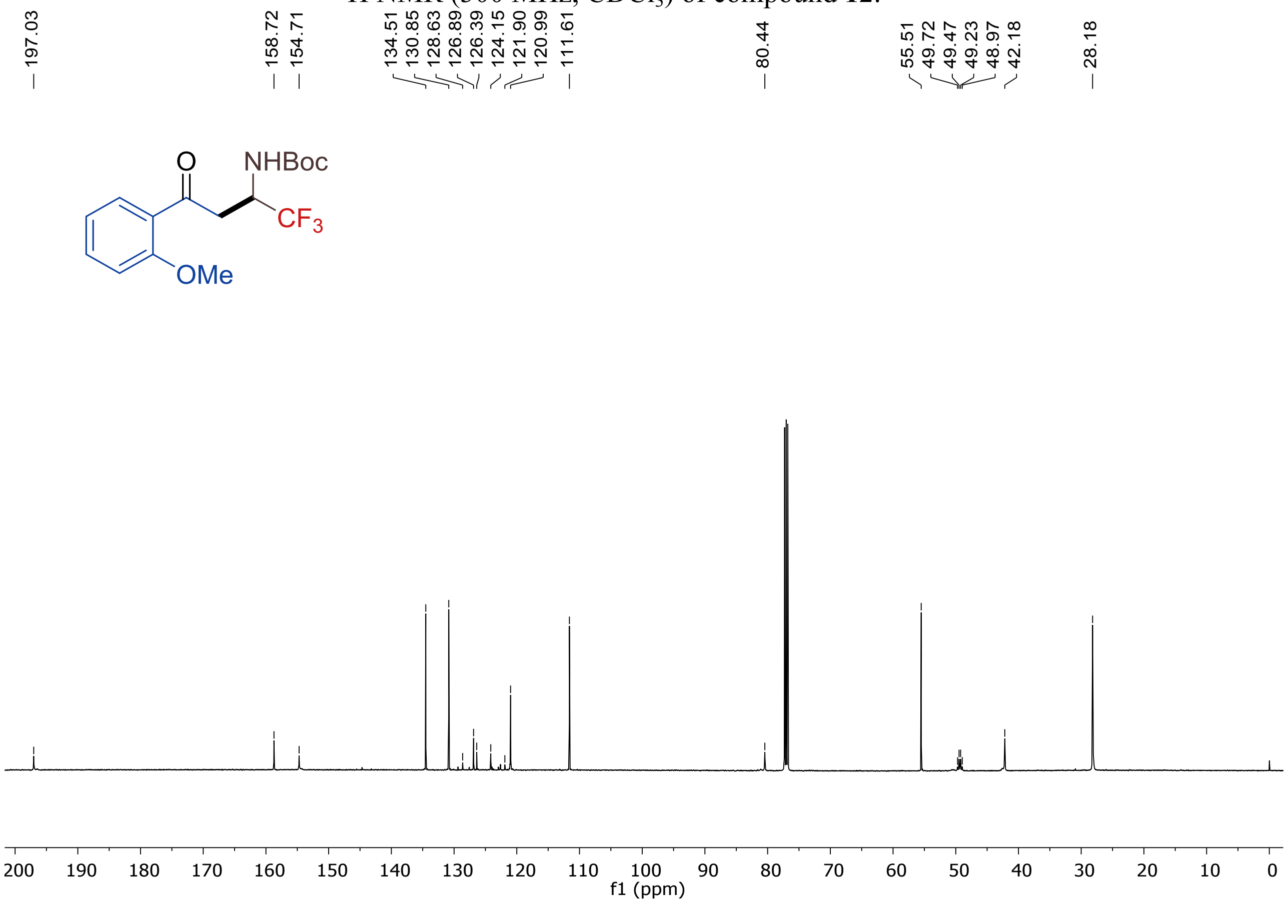

$^{13}C\{^{1}H\}$ NMR (126 MHz, $CDCl_3$) of compound **12**.

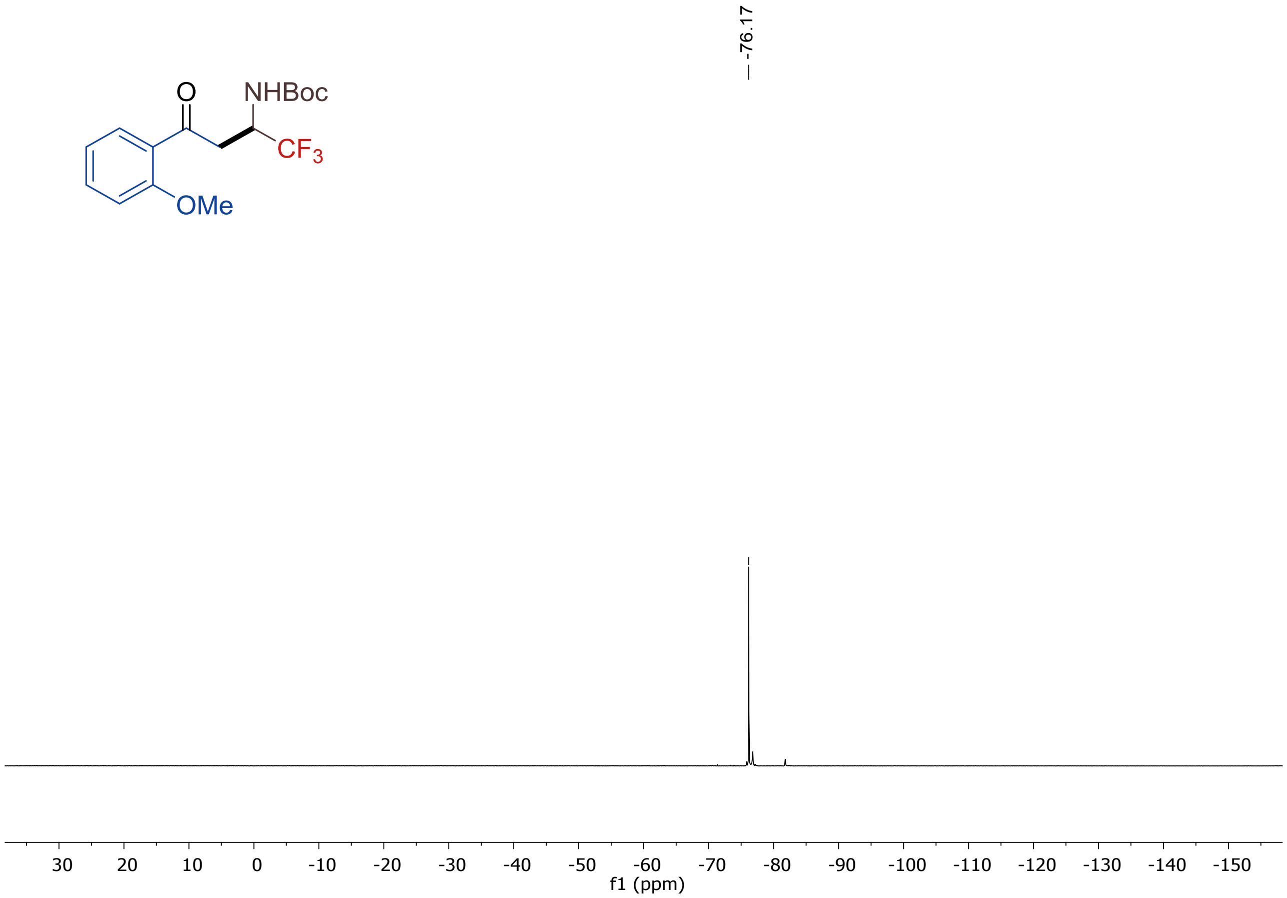

$^{19}F\{^{1}H\}$ NMR (282 MHz, $CDCl_3$) of compound **12**.

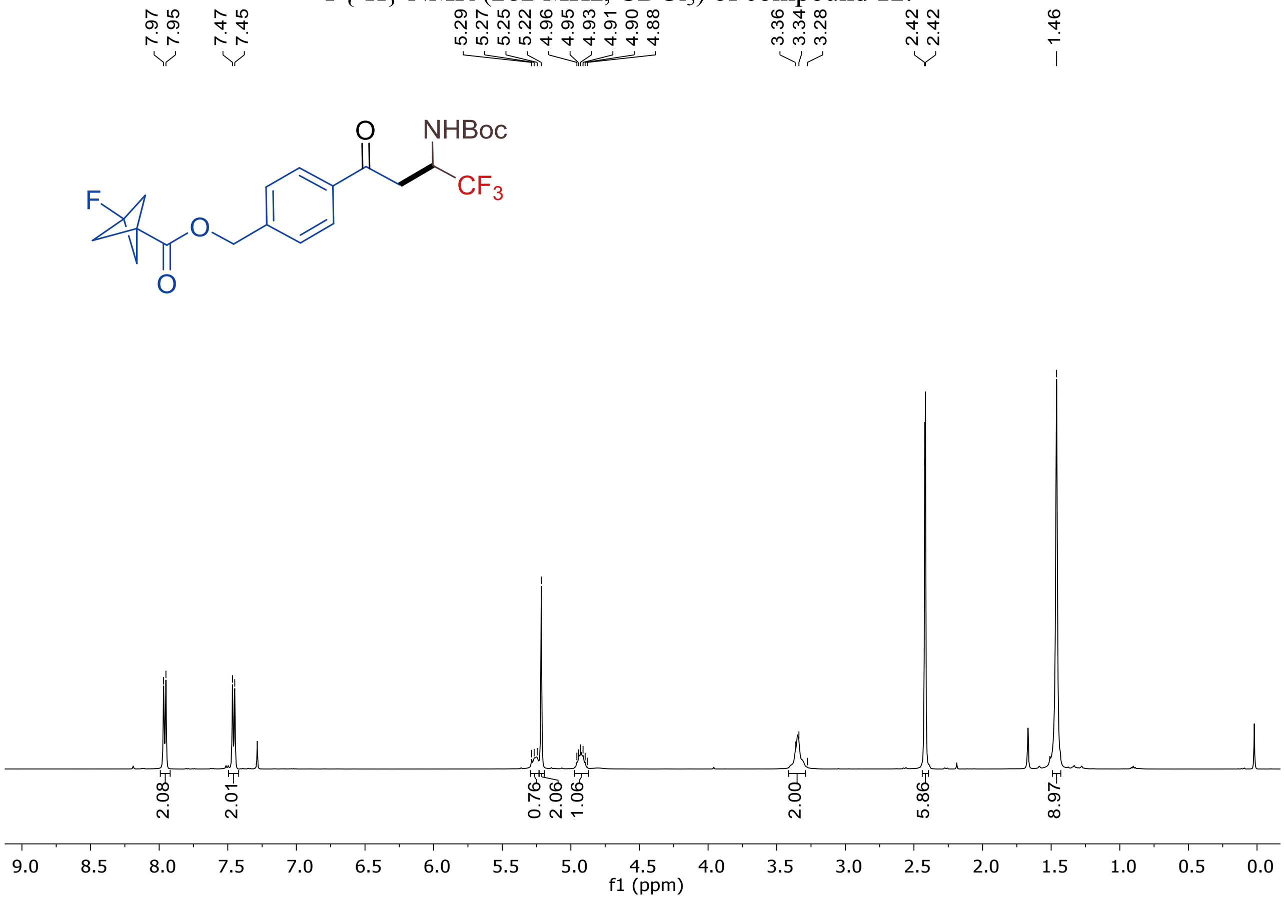

^{1}H NMR (500 MHz, $CDCl_3$) of compound **13**.

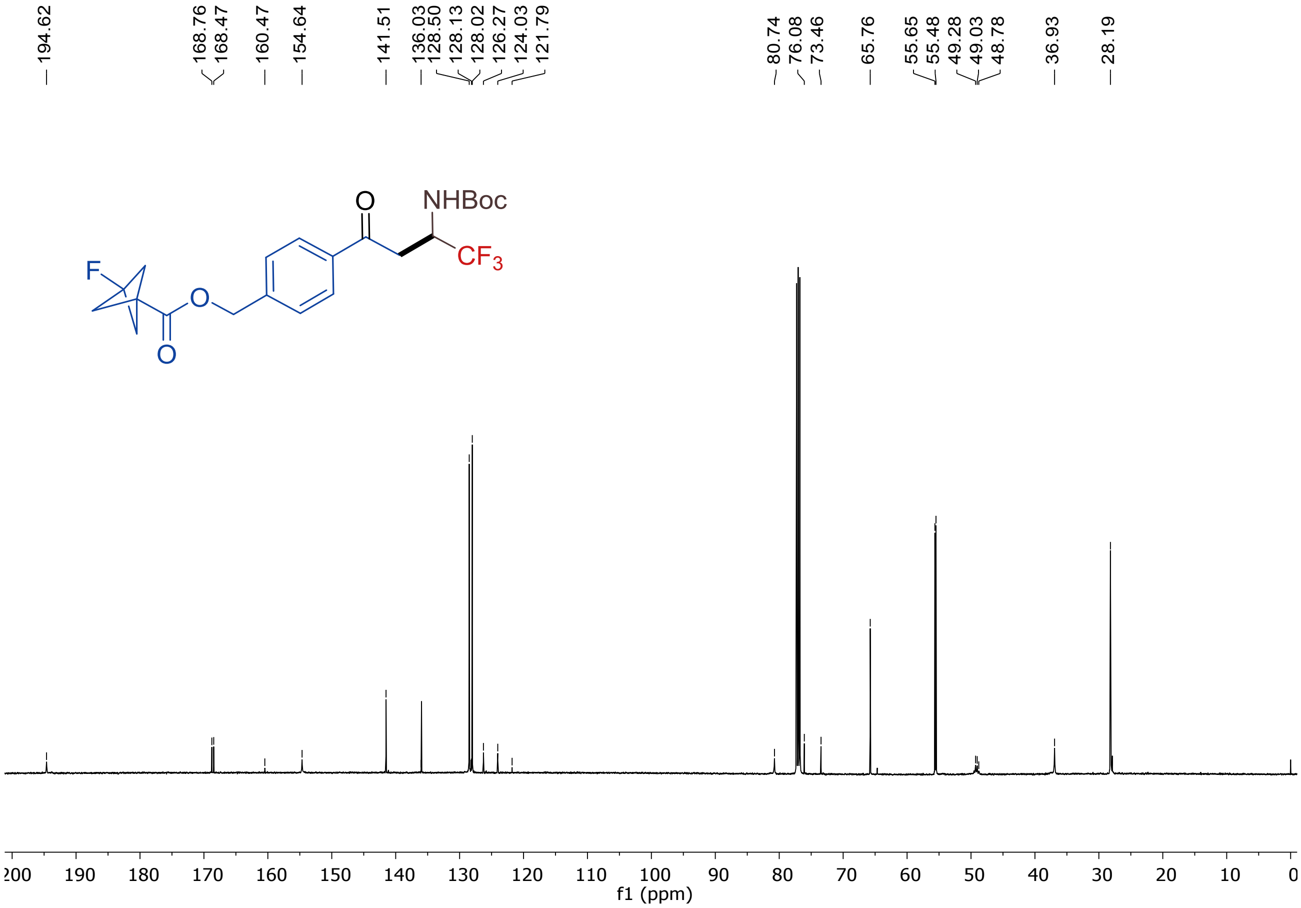

$^{13}C\{^{1}H\}$ NMR (126 MHz, $CDCl_3$) of compound **13**.

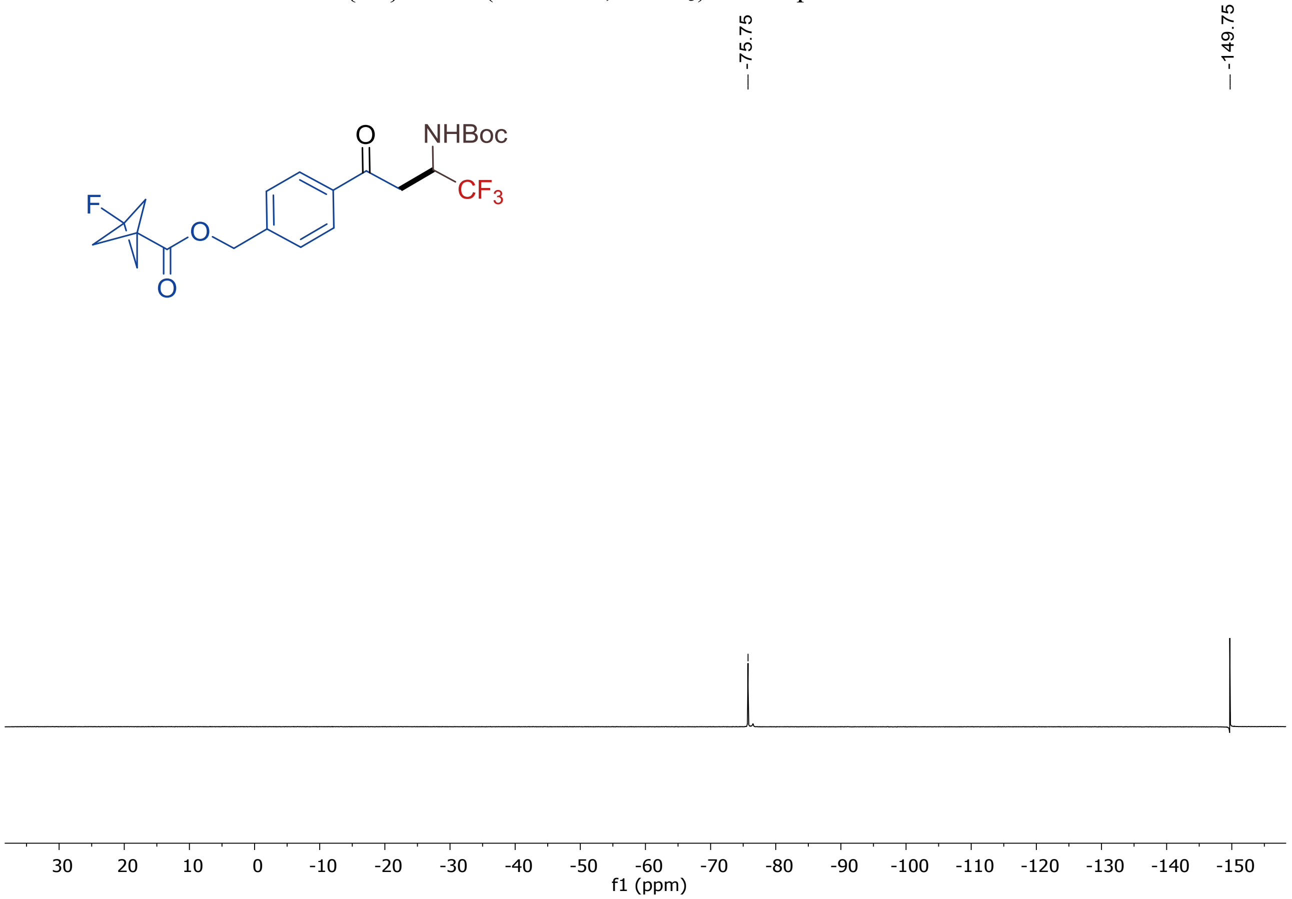

$^{19}F\{^{1}H\}$ NMR (282 MHz, $CDCl_3$) of compound **13**.

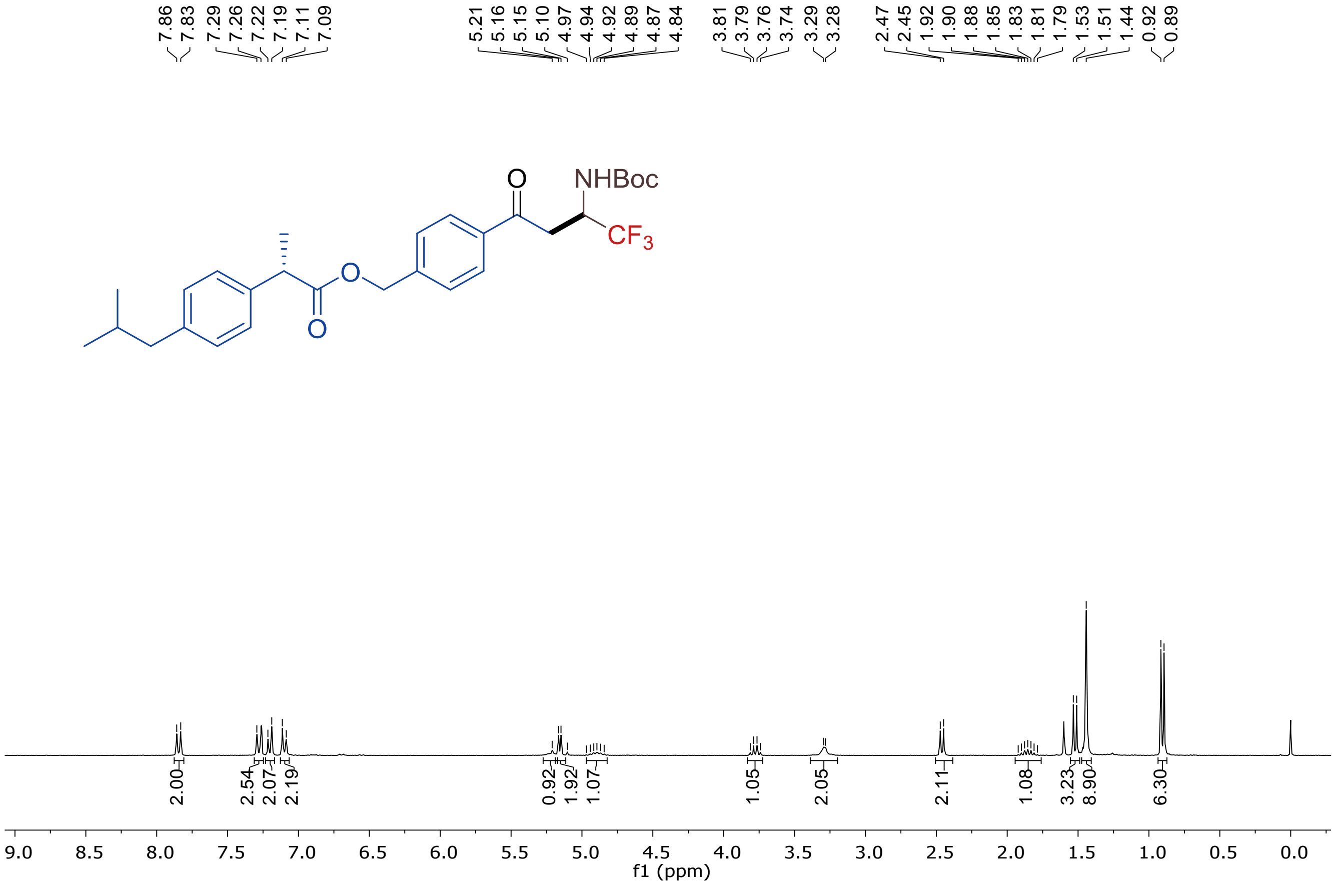

^{1}H NMR (300 MHz, $CDCl_3$) of compound **14**.

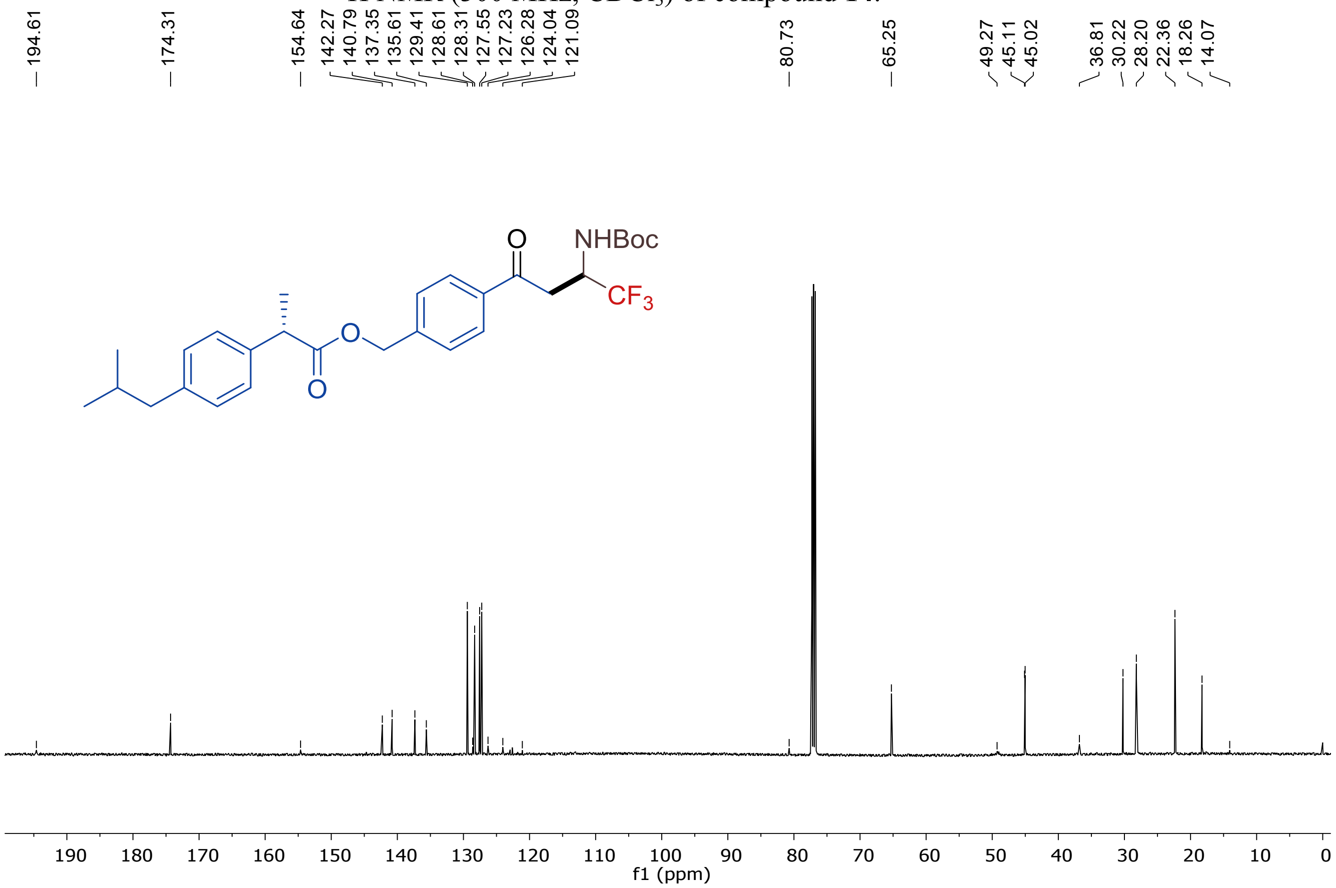

^{13}C{^{1}H} NMR (126 MHz, $CDCl_3$) of compound **14**.

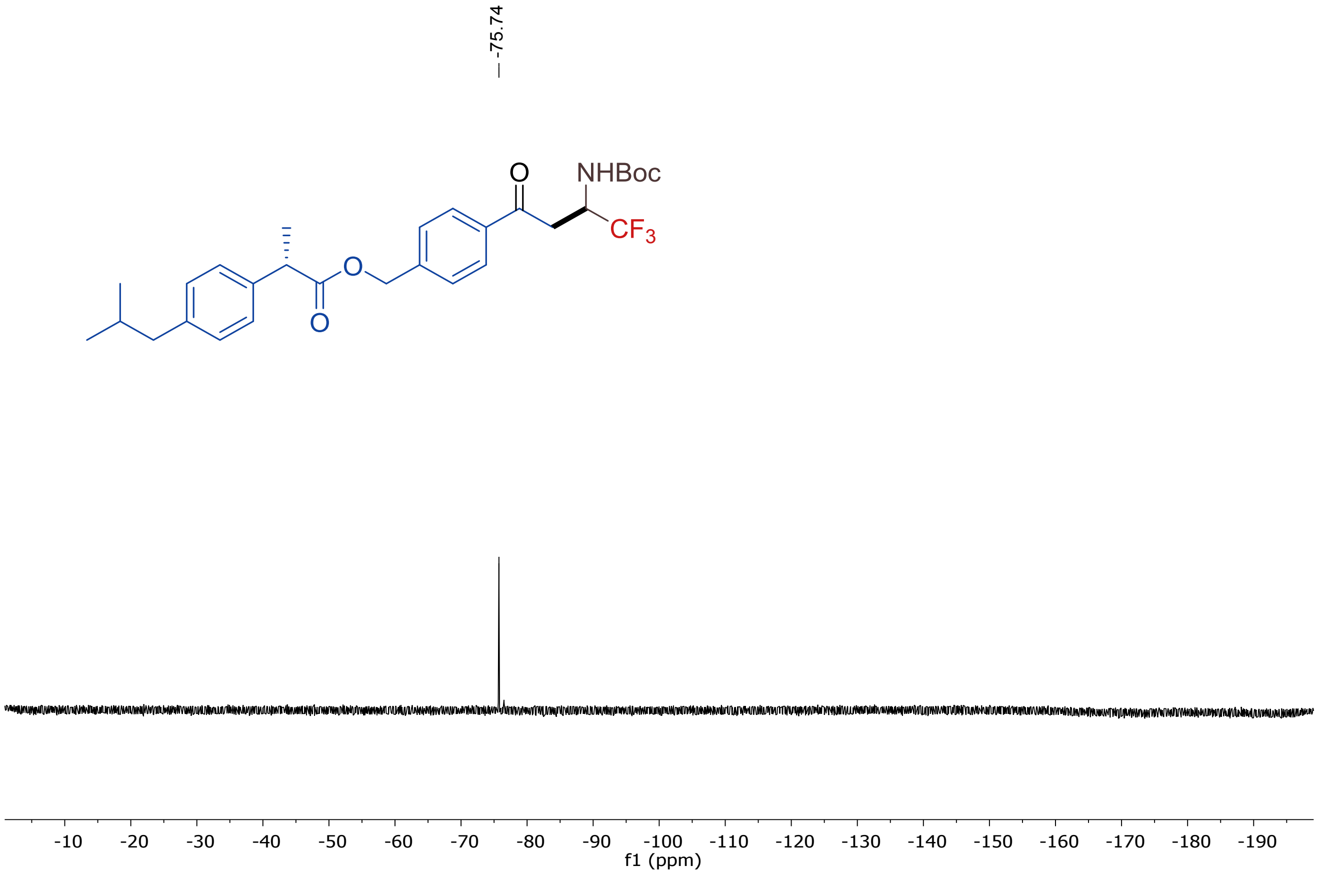

$^{19}F\{^{1}H\}$ NMR (282 MHz, $CDCl_3$) of compound **14**.

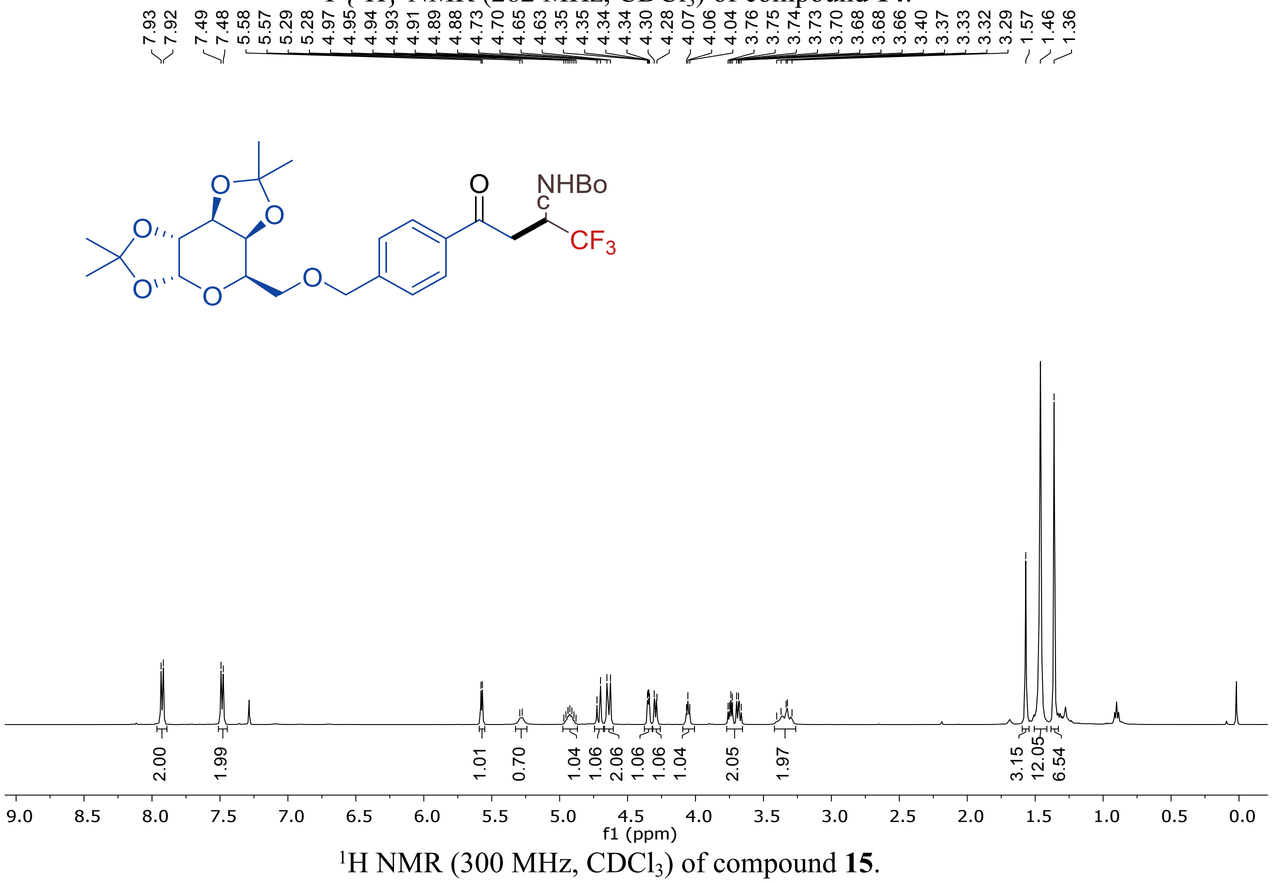

^{1}H NMR (300 MHz, $CDCl_3$) of compound **15**.

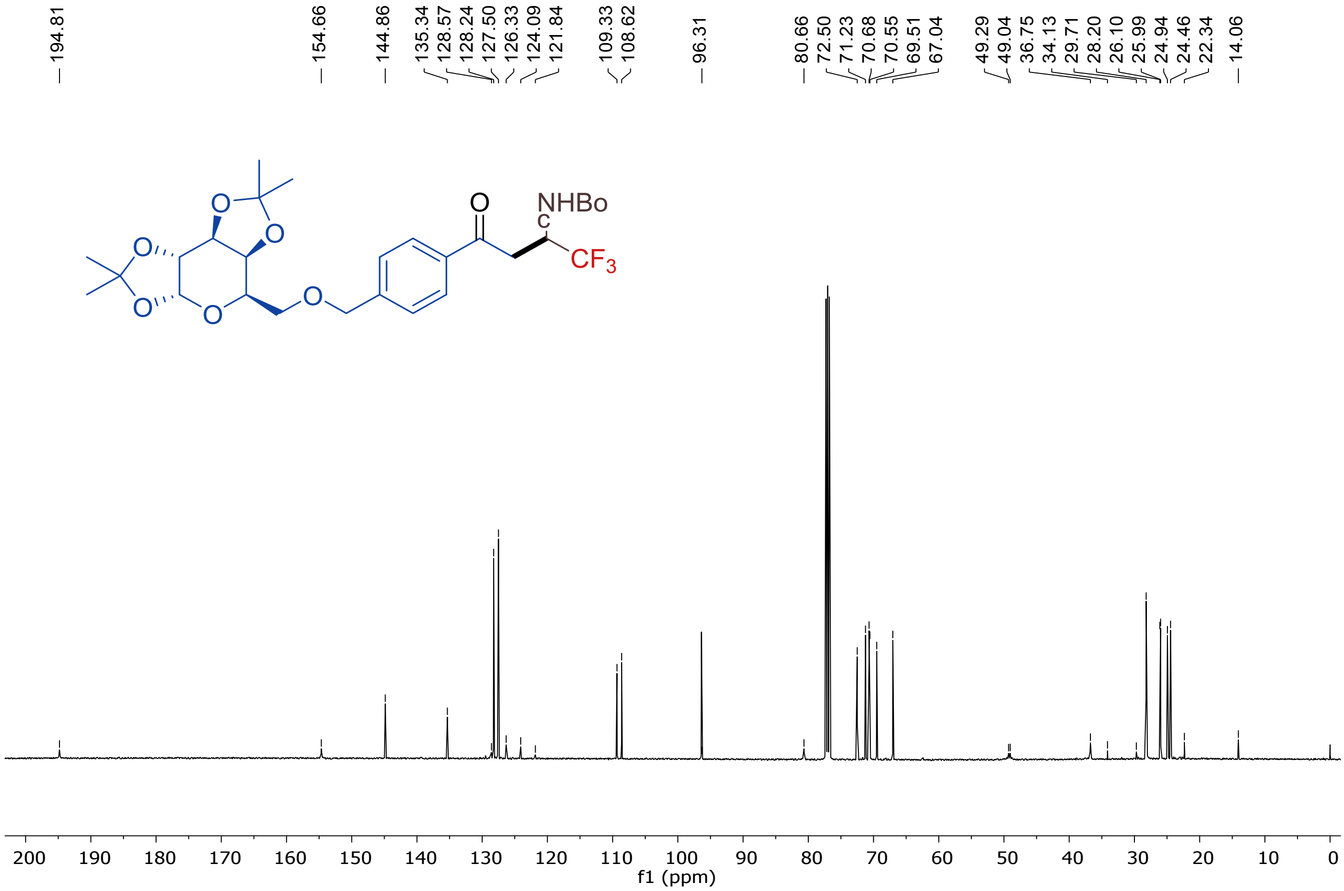

$^{13}C\{^{1}H\}$ NMR (126 MHz, $CDCl_3$) of compound **15**.

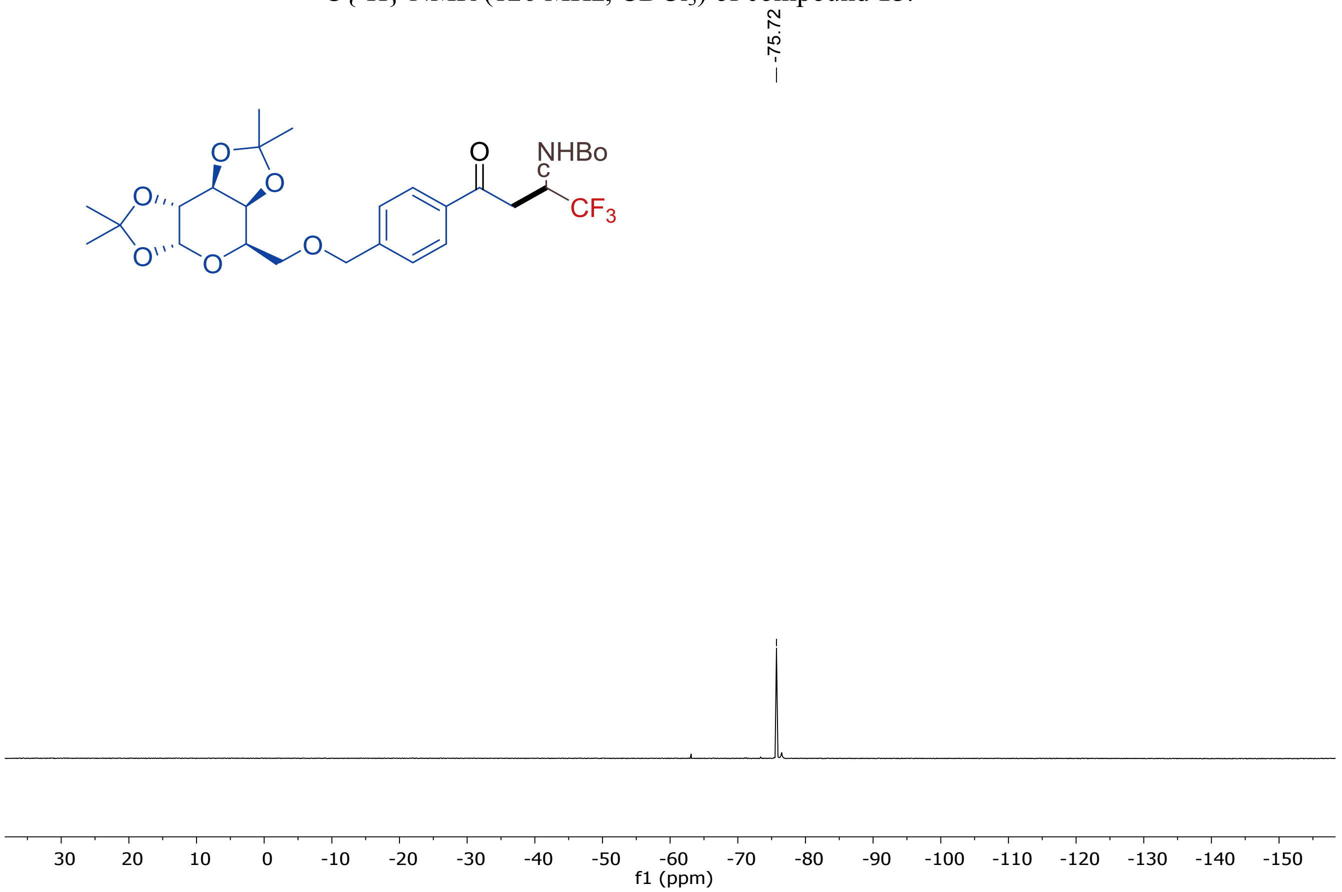

$^{19}F\{^{1}H\}$ NMR (282 MHz, $CDCl_3$) of compound **15**.

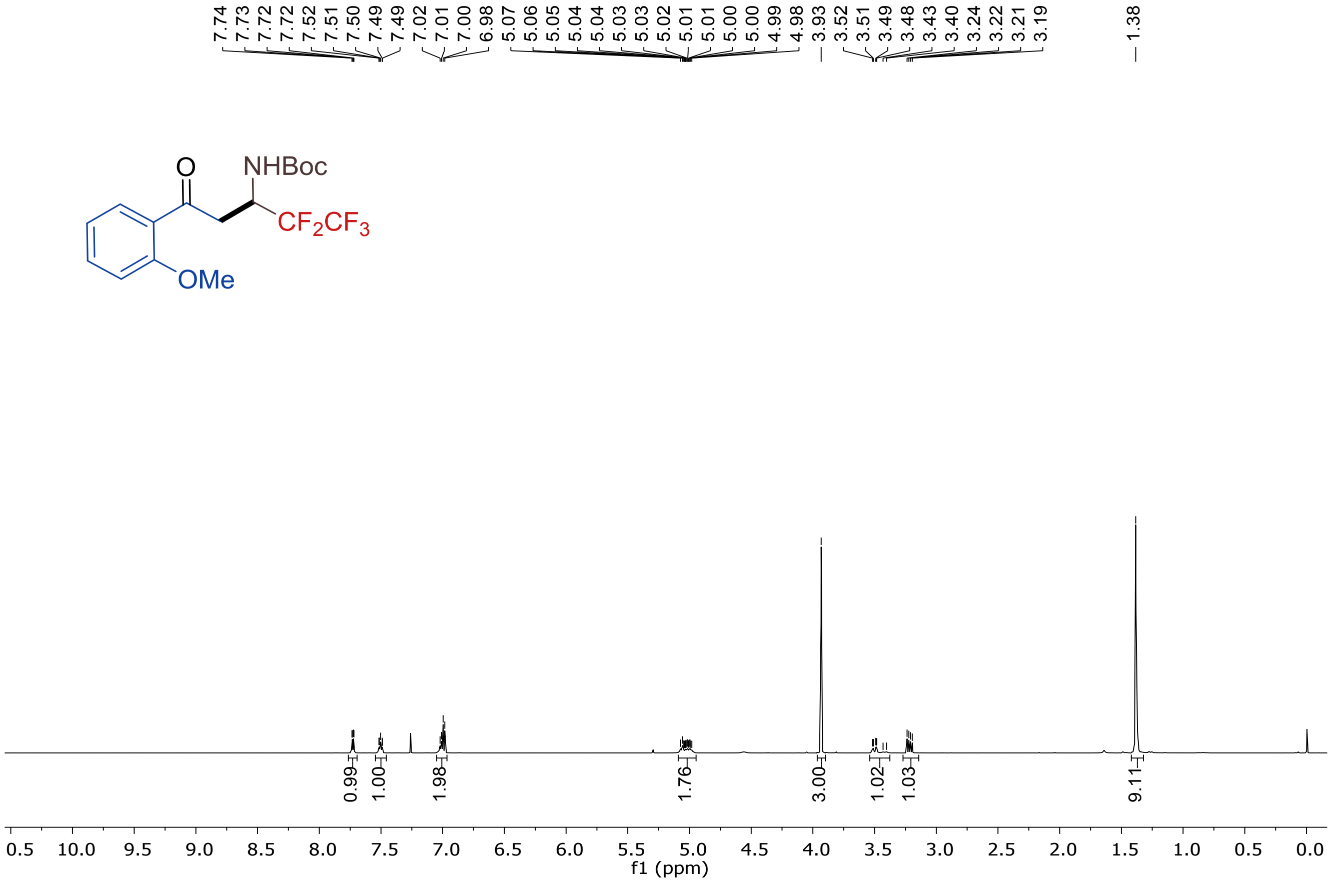

^{1}H NMR (600 MHz, $CDCl_3$) of compound **16**.

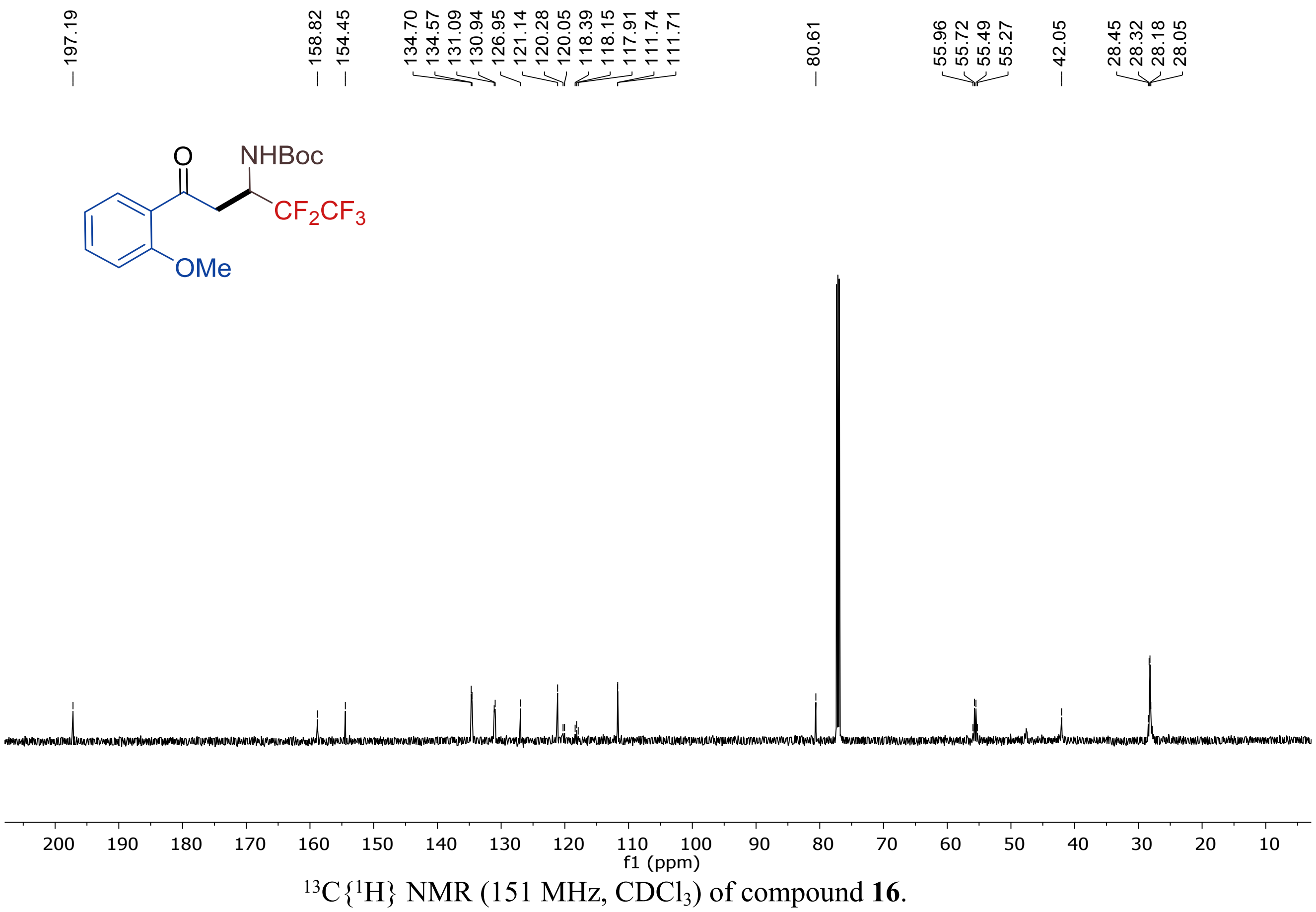

^{13}C{^{1}H} NMR (151 MHz, $CDCl_3$) of compound **16**.

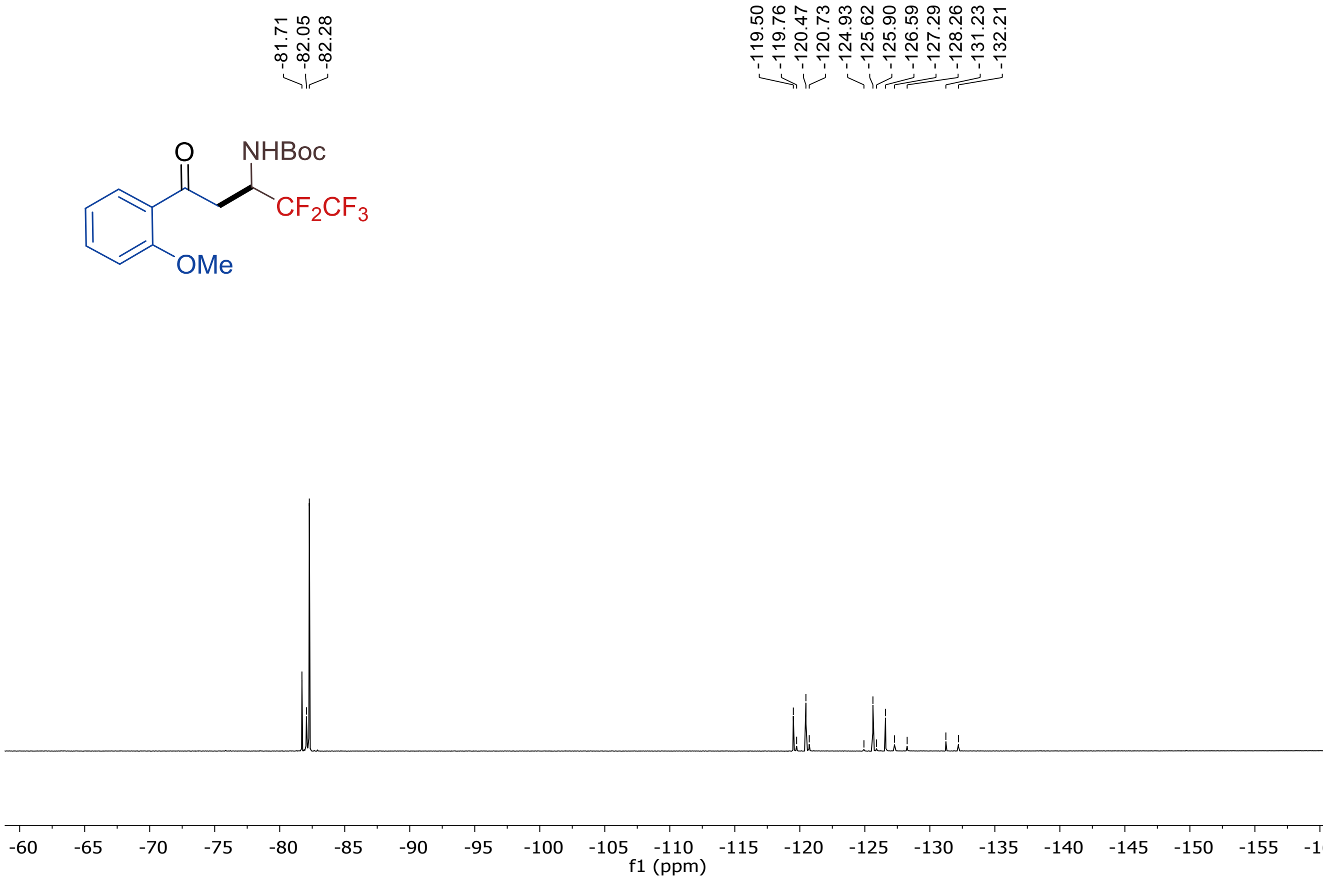

$^{19}F\{^{1}H\}$ NMR (282 MHz, $CDCl_3$) of compound **16**.

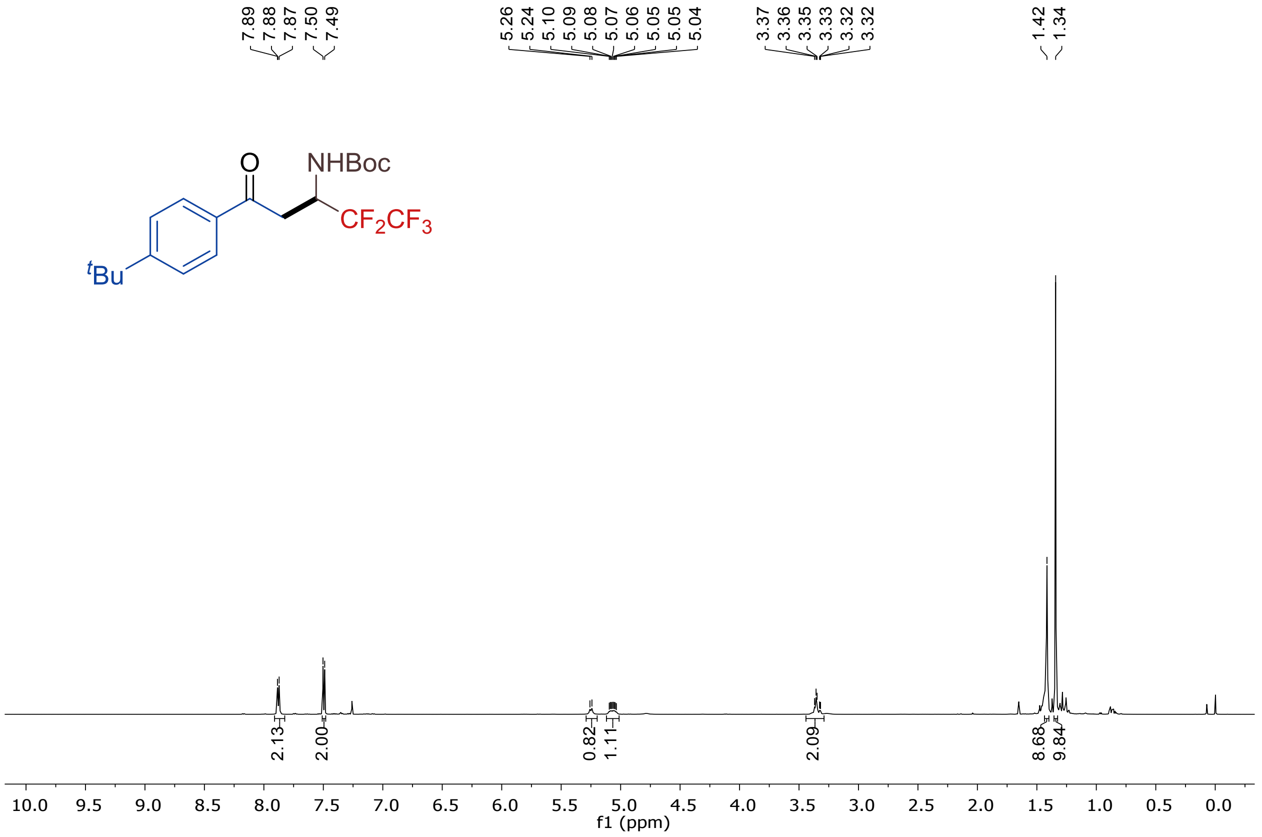

^{1}H NMR (600 MHz, $CDCl_3$) of compound **17**.

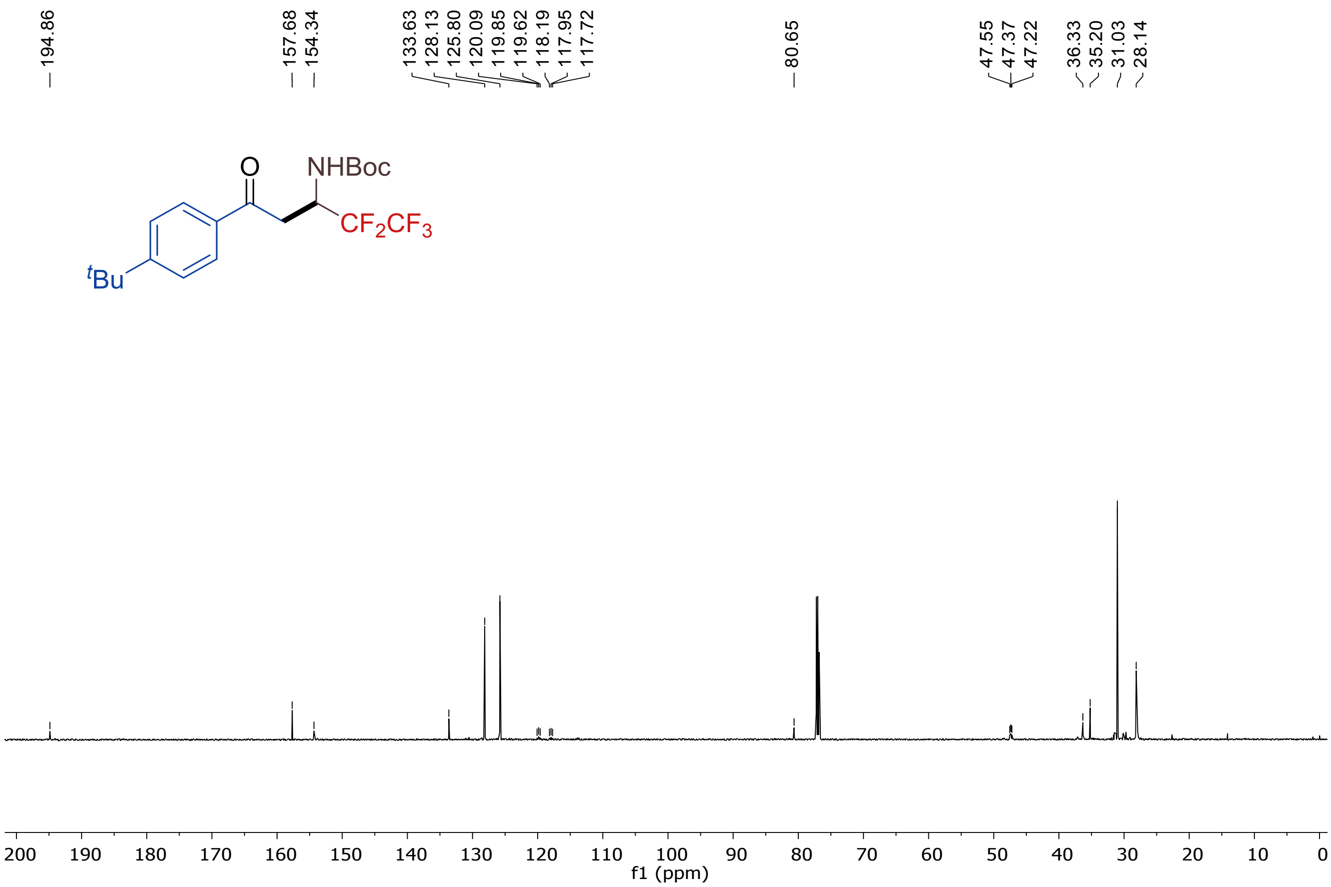

$^{13}C\{^1H\}$ NMR (151 MHz, $CDCl_3$) of compound **17**.

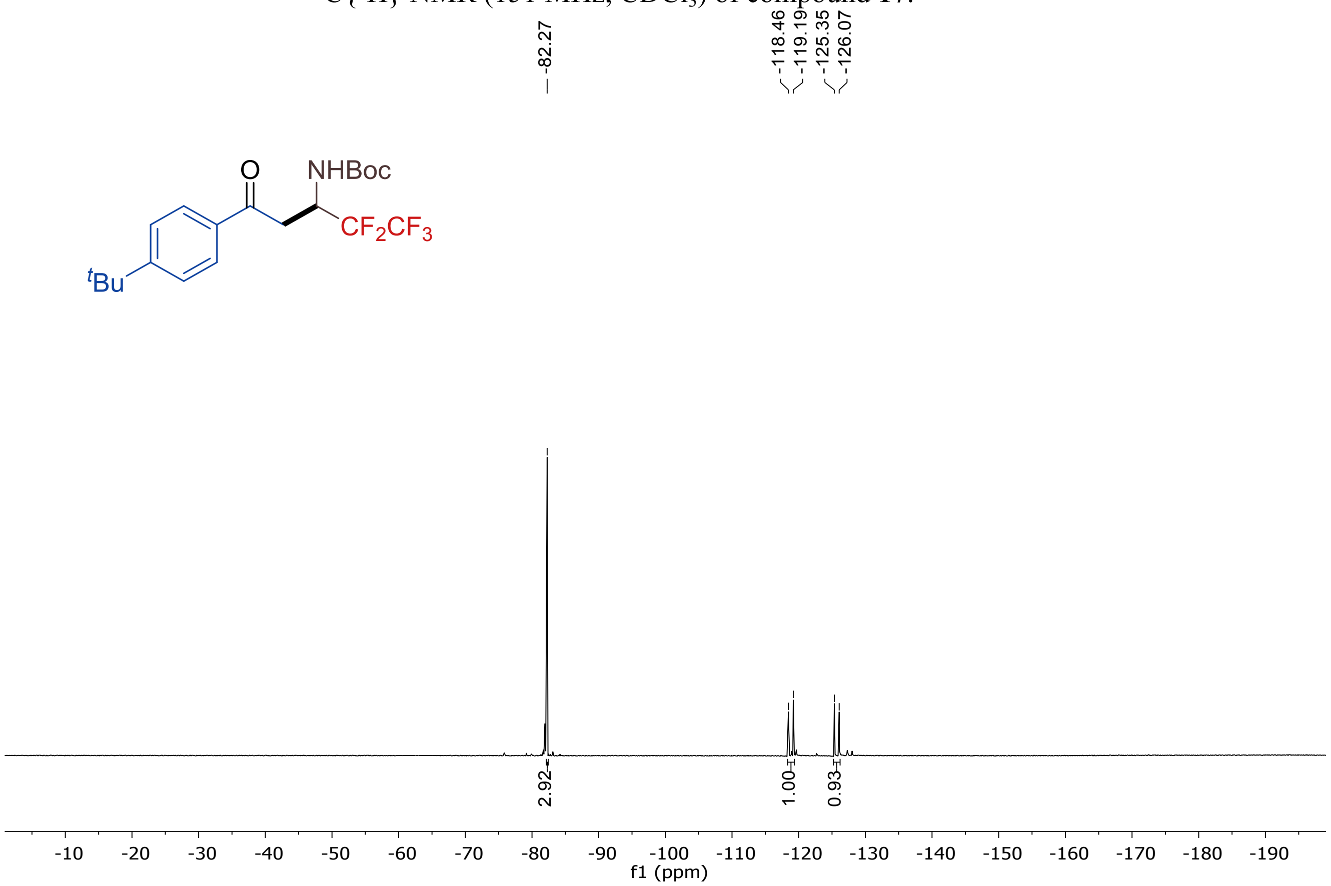

$^{19}F\{^1H\}$ NMR (377 MHz, $CDCl_3$) of compound **17**.

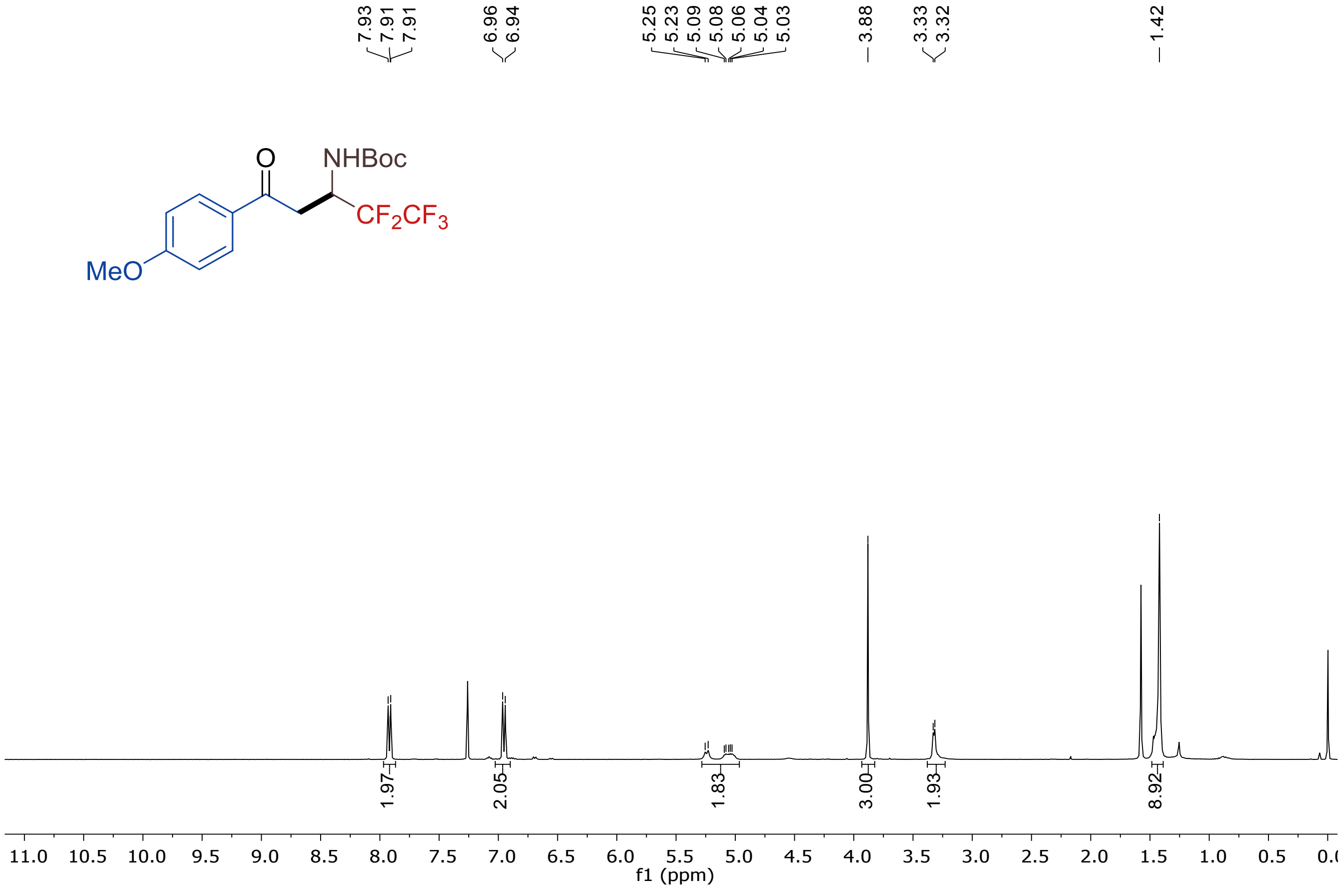

^{1}H NMR (400 MHz, $CDCl_3$) of compound **18**.

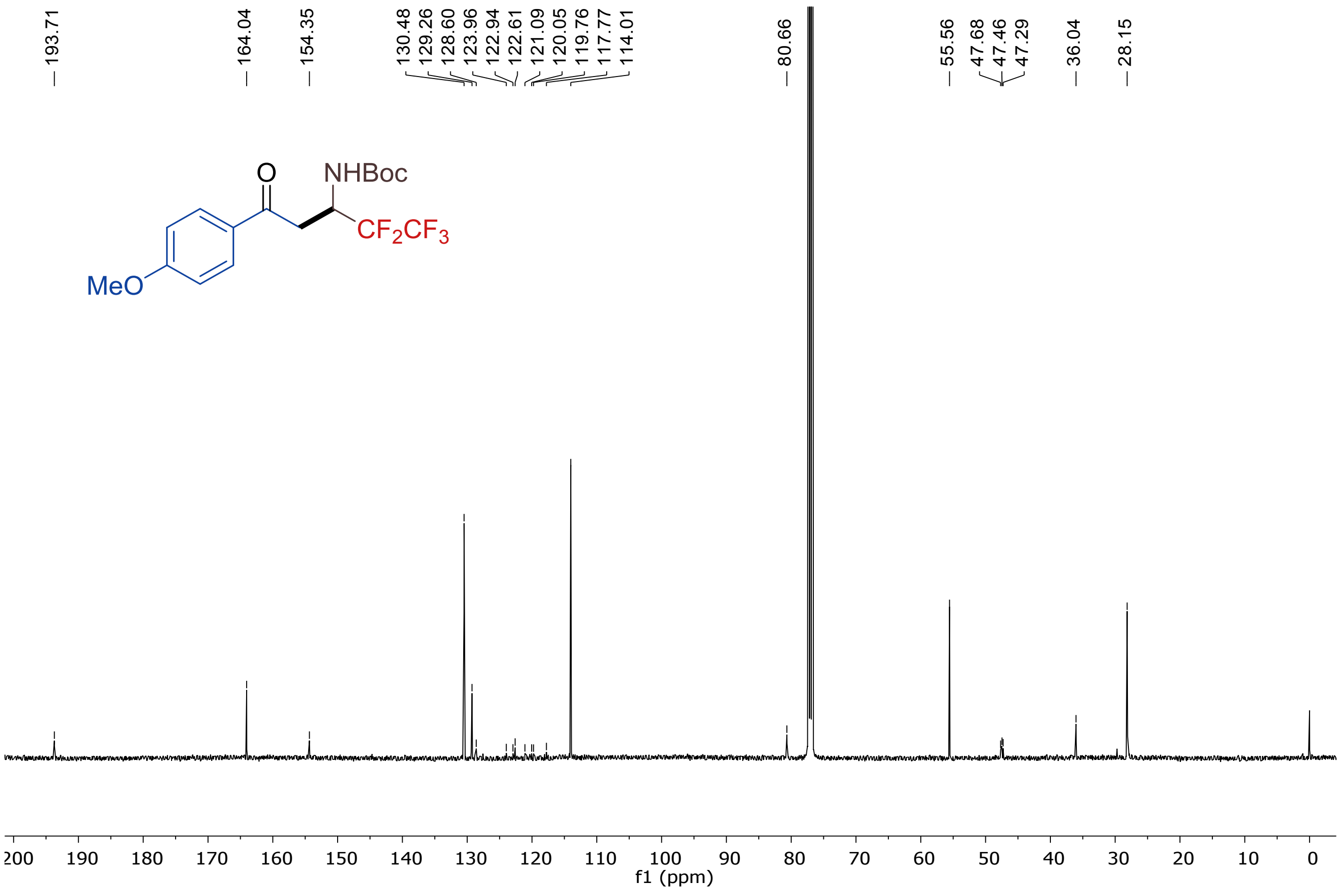

^{13}C{^{1}H} NMR (126 MHz, $CDCl_3$) of compound **18**.

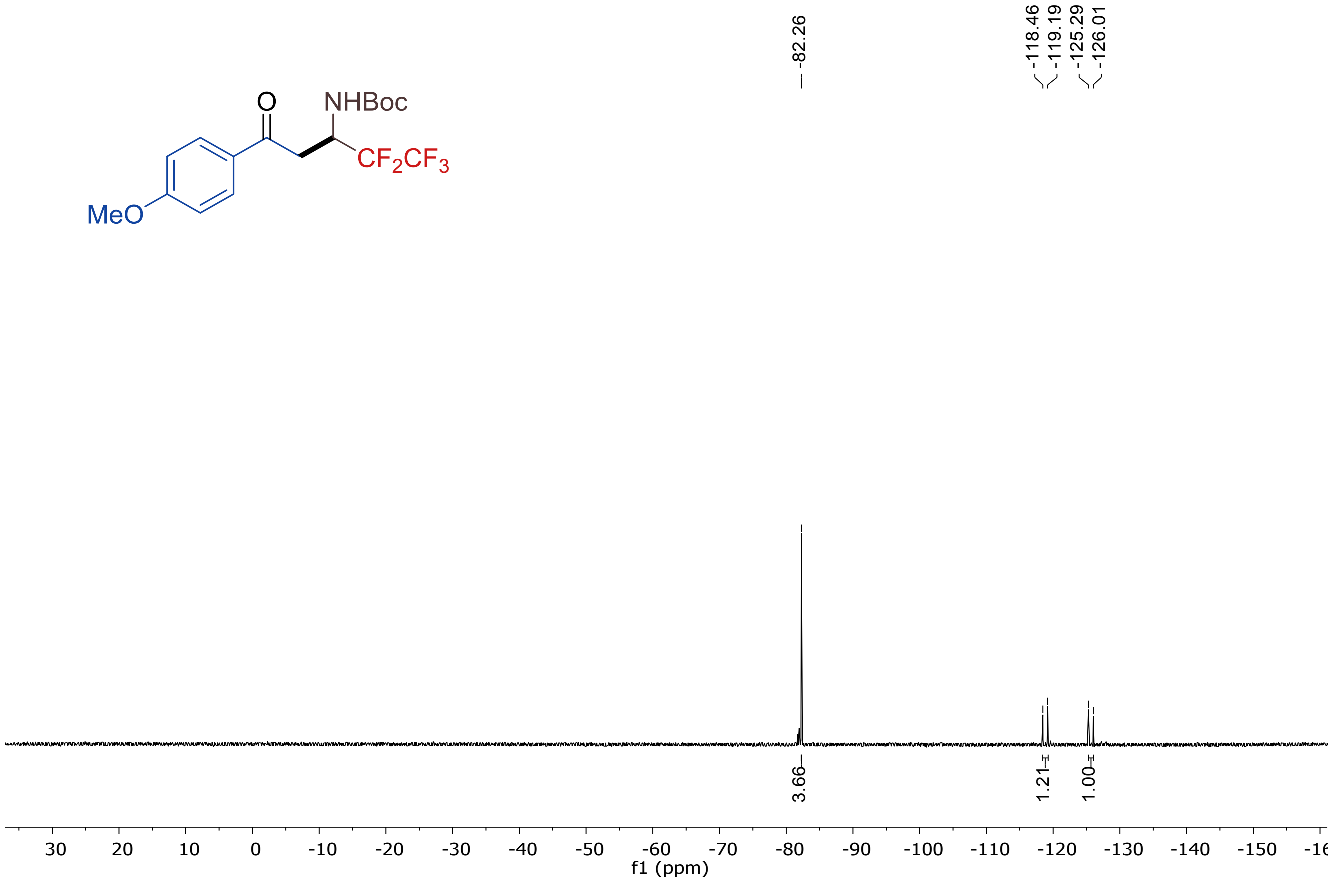

$^{19}F\{^{1}H\}$ NMR (377 MHz, $CDCl_3$) of compound **18**.

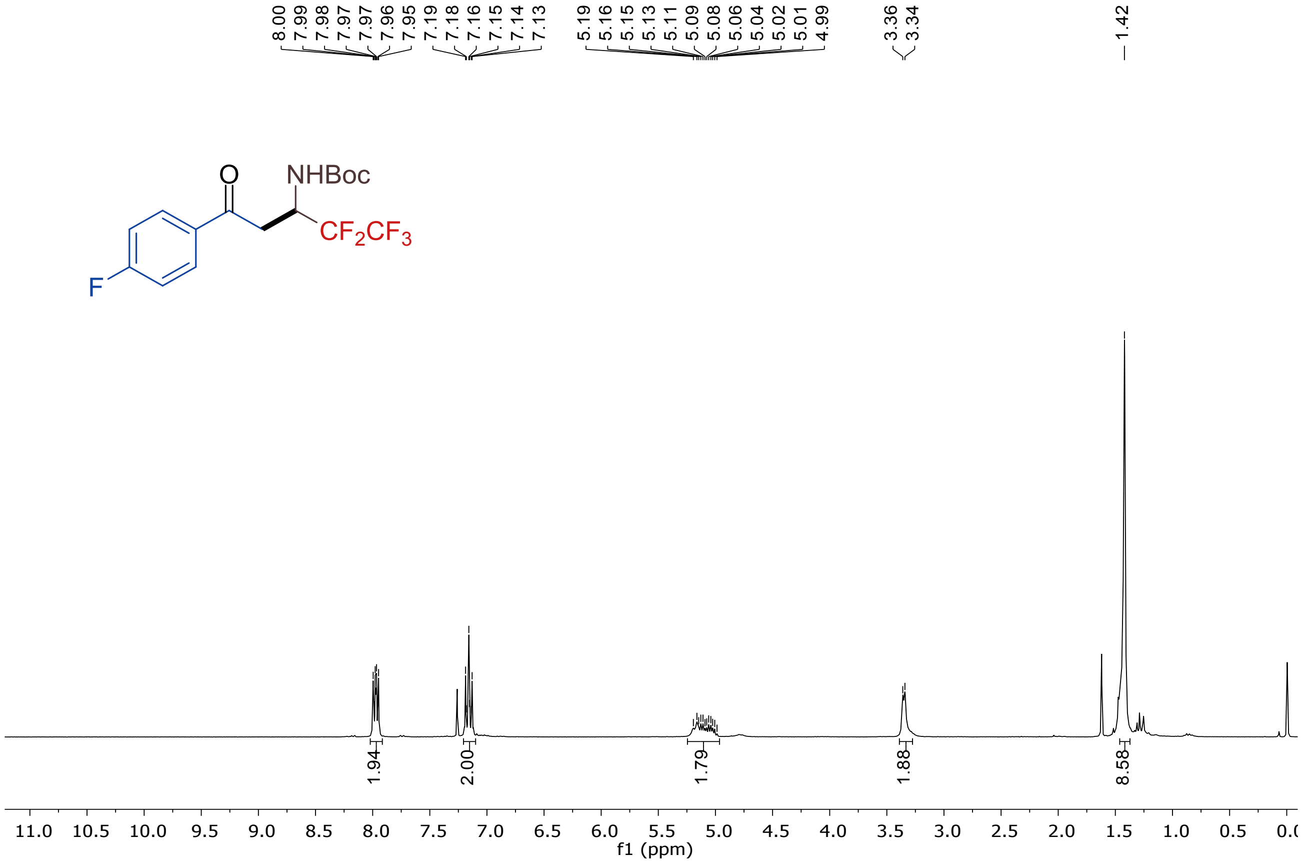

^{1}H NMR (600 MHz, $CDCl_3$) of compound **19**.

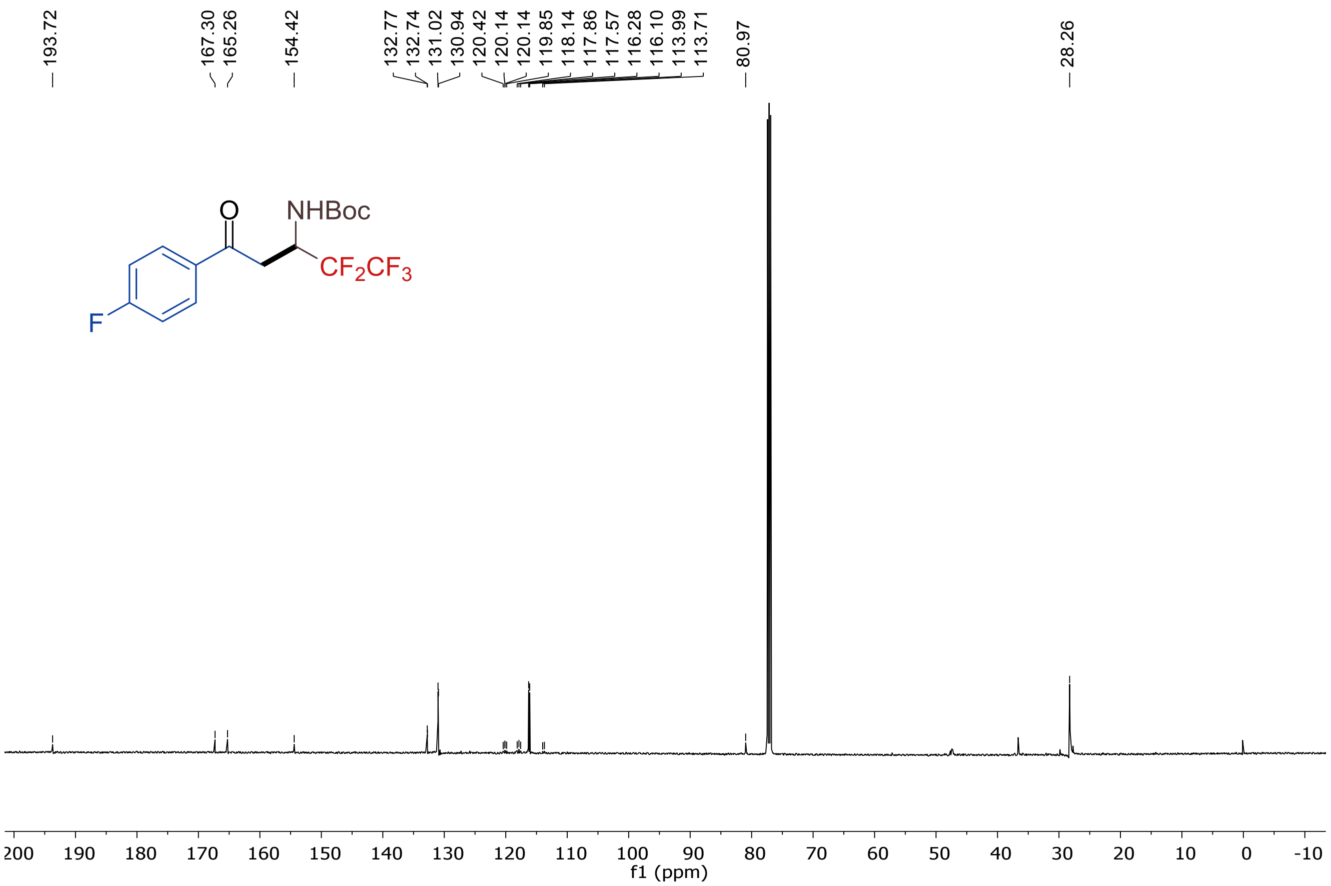

$^{13}C\{^1H\}$ NMR (126 MHz, $CDCl_3$) of compound **19**.

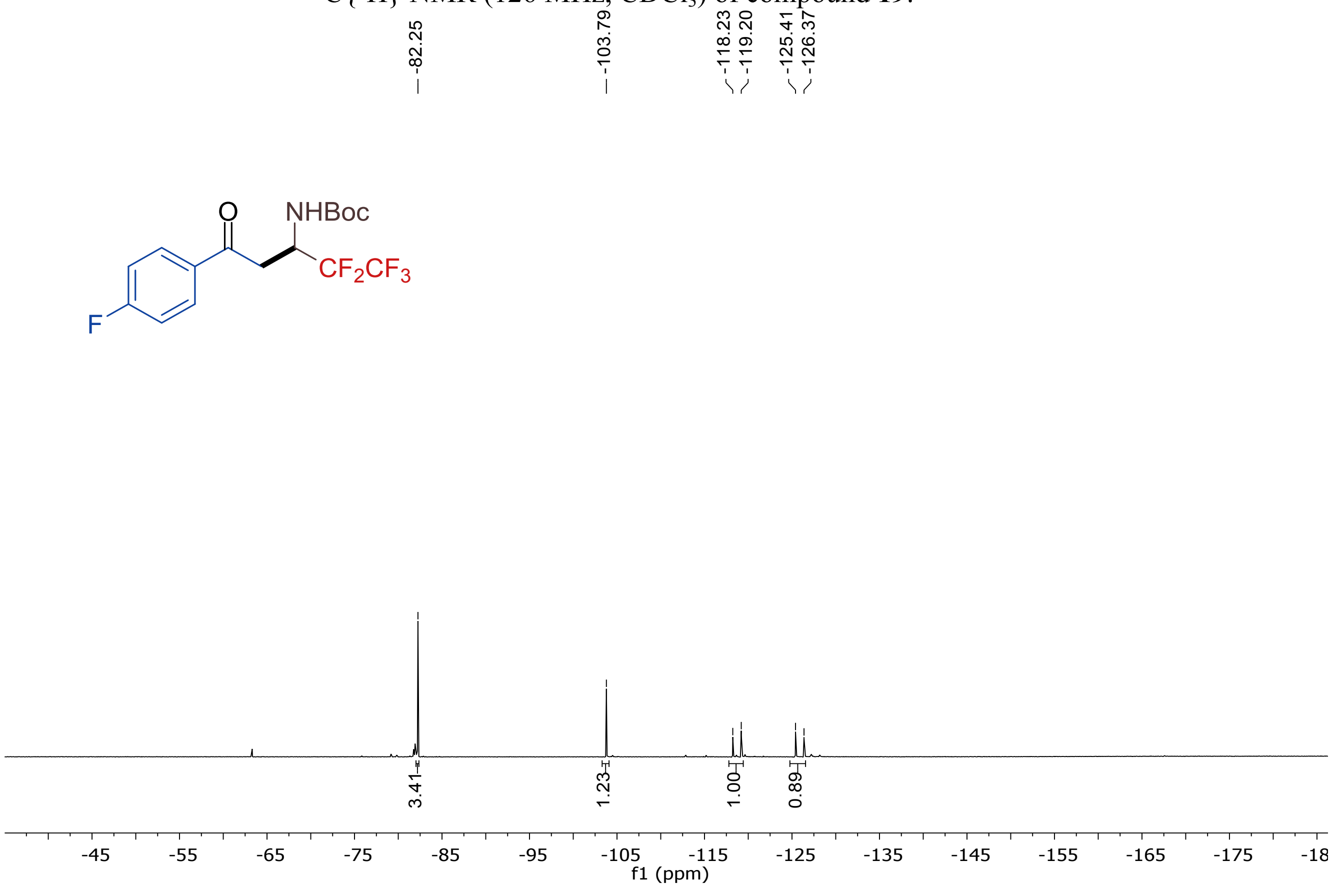

$^{19}F\{^1H\}$ NMR (377 MHz, $CDCl_3$) of compound **19**.

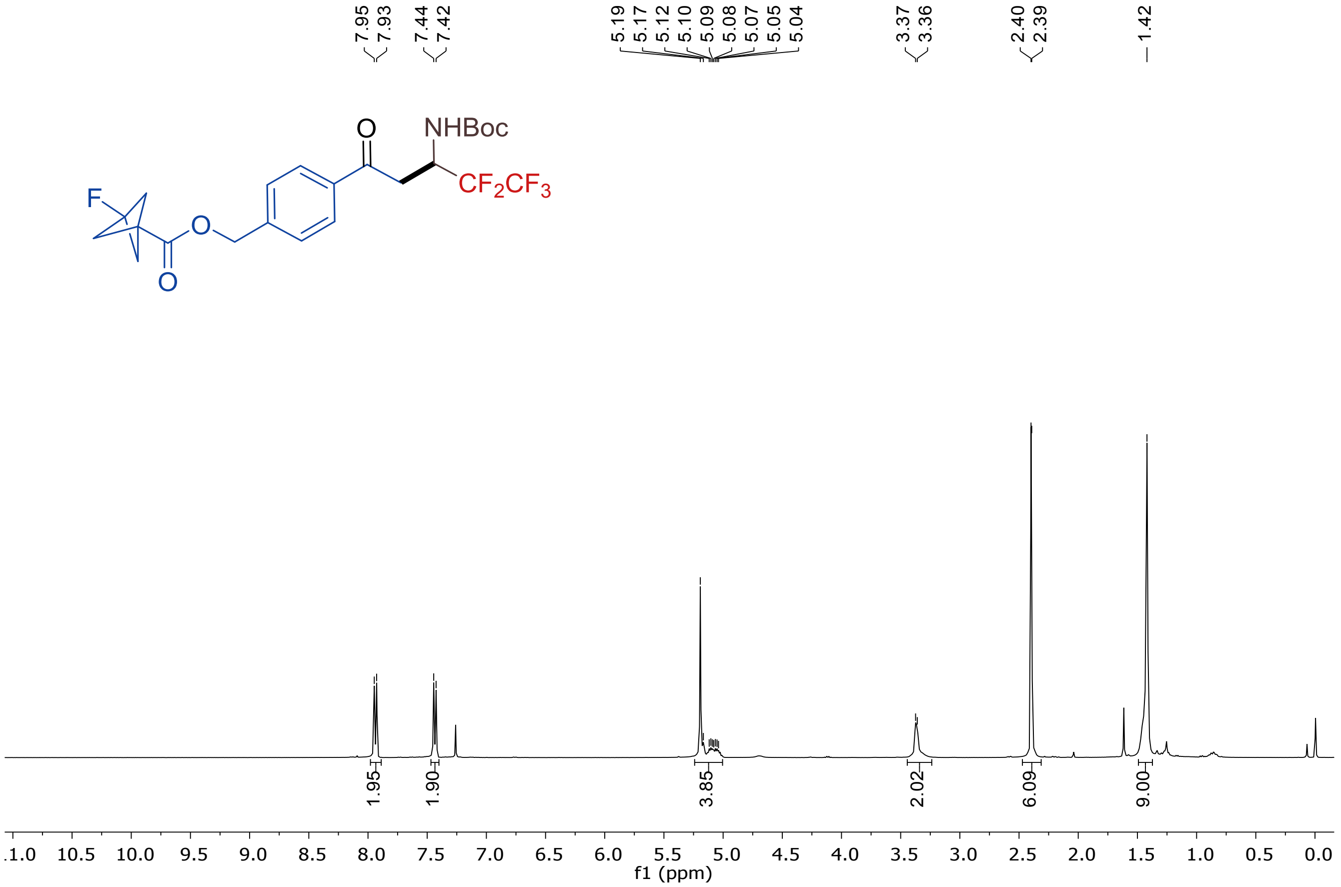

^{1}H NMR (400 MHz, $CDCl_3$) of compound **20**.

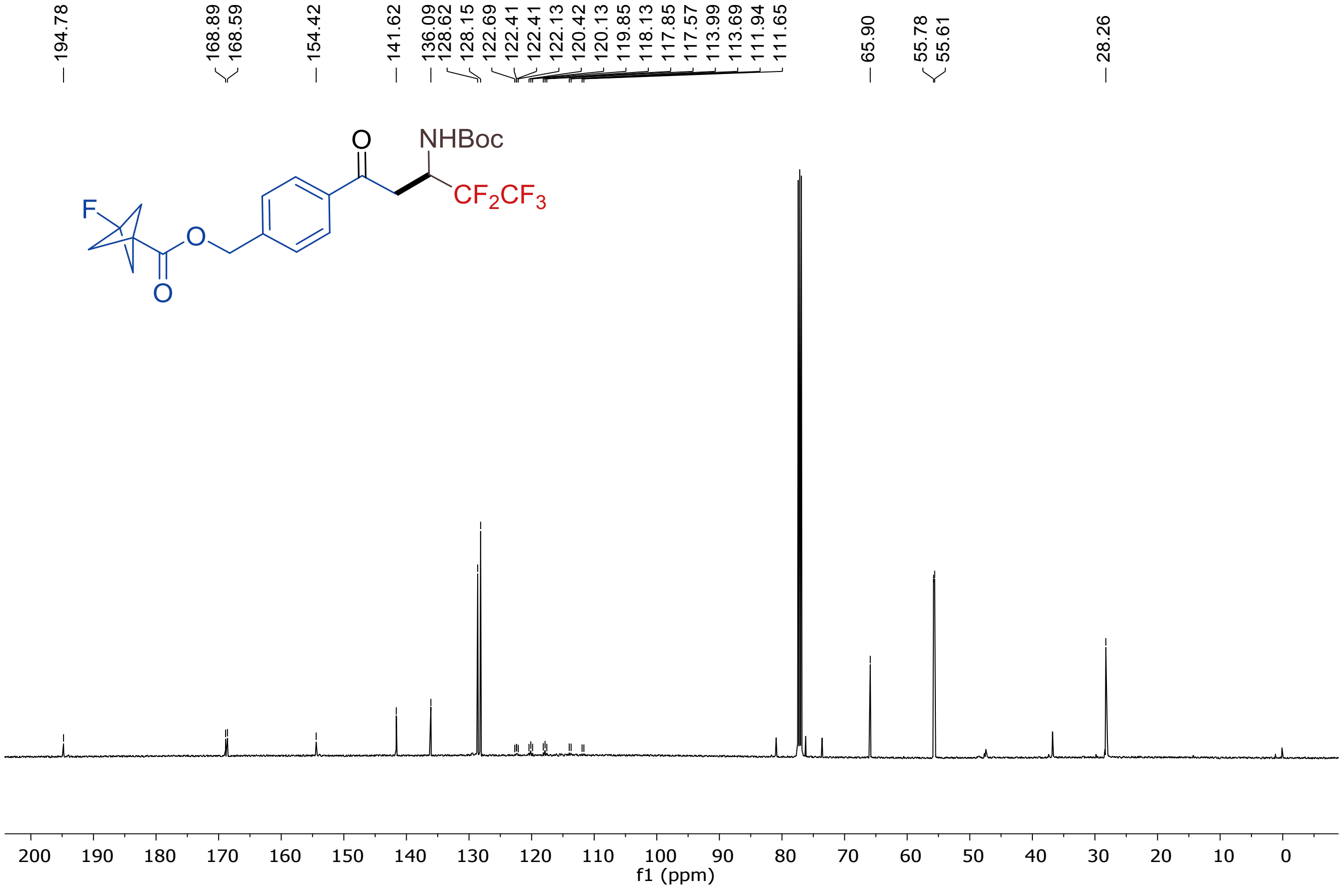

^{13}C{^{1}H} NMR (126 MHz, $CDCl_3$) of compound **20**.

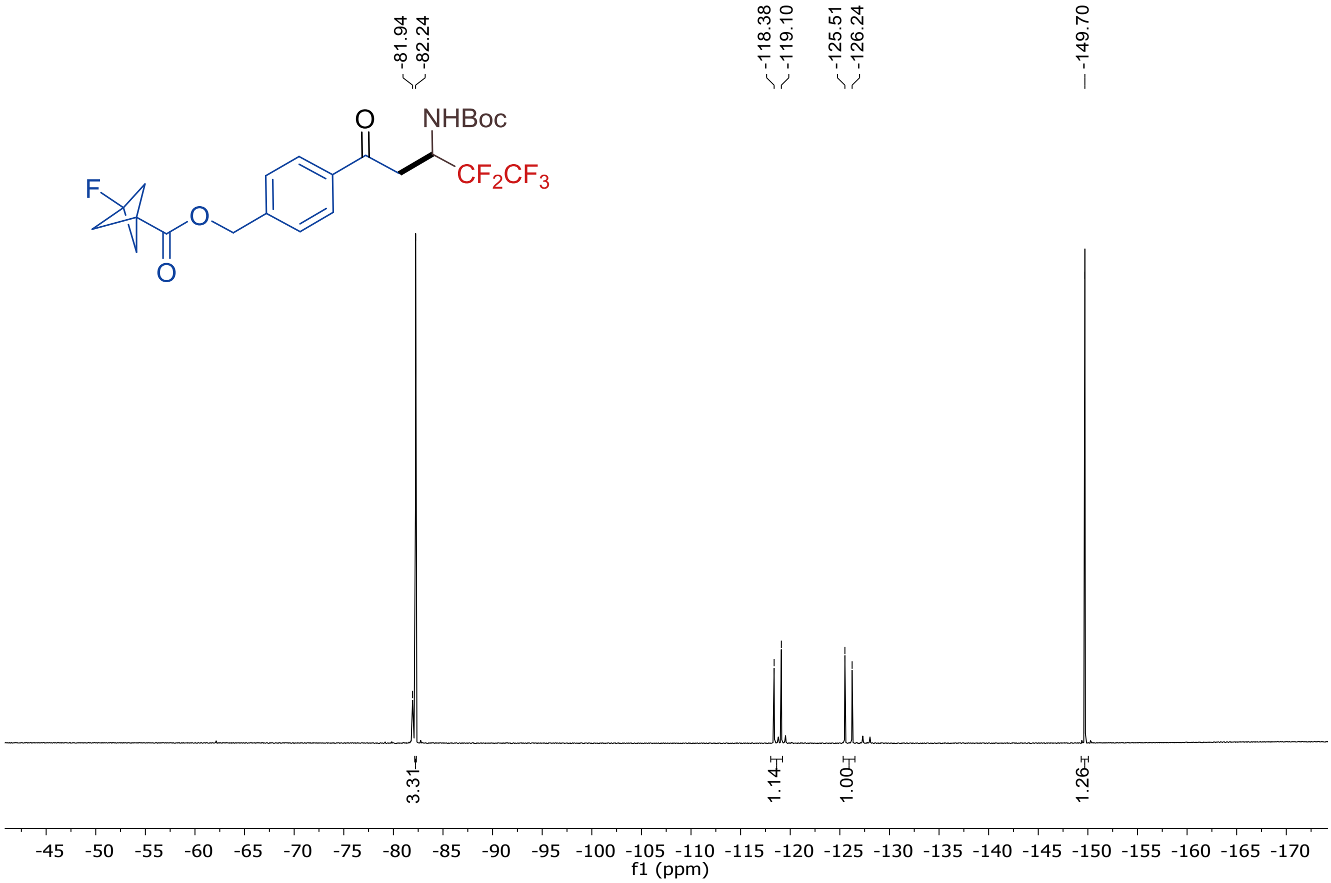

$^{19}F\{^{1}H\}$ NMR (377 MHz, $CDCl_3$) of compound **20**.

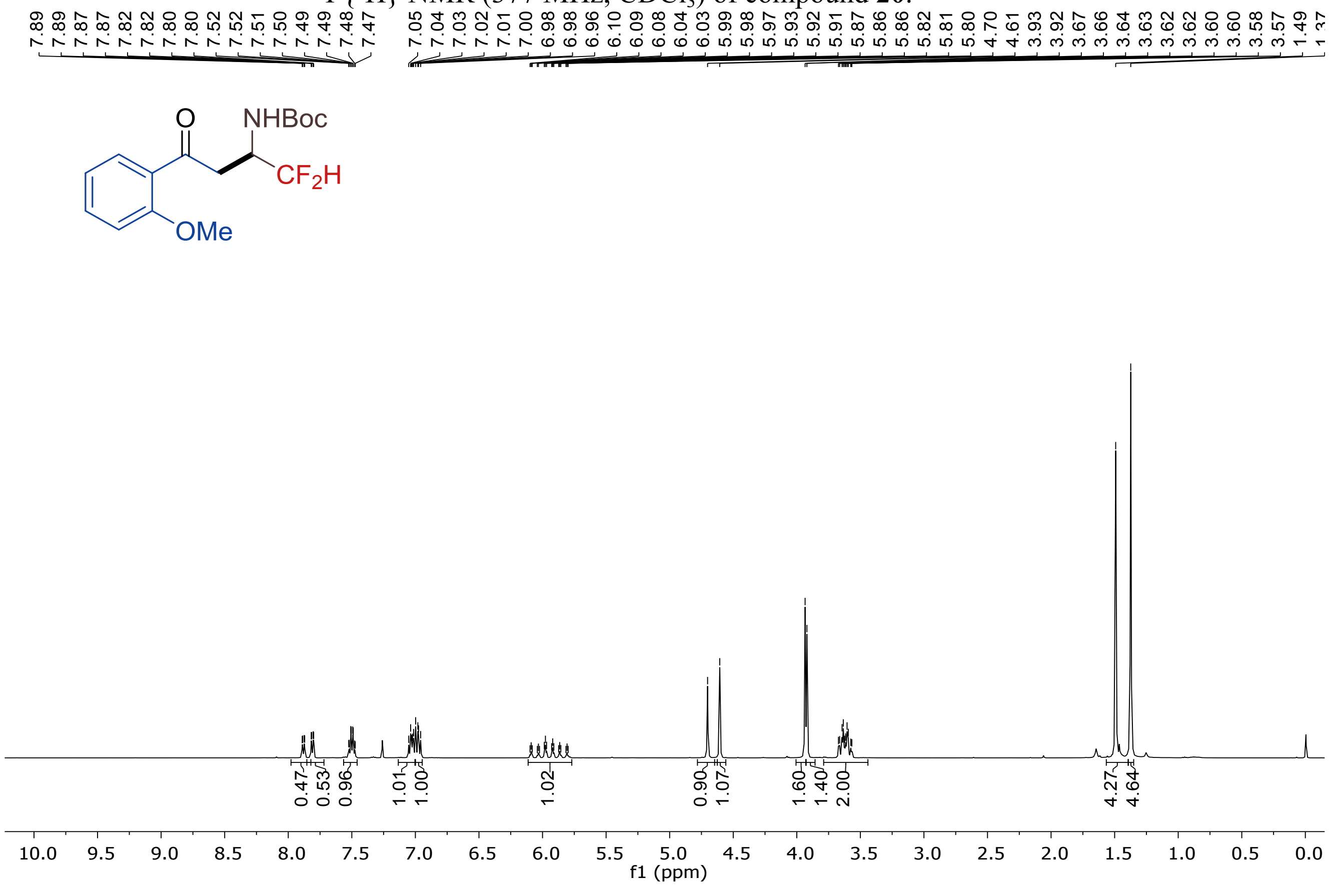

^{1}H NMR (500 MHz, $CDCl_3$) of compound **21**.

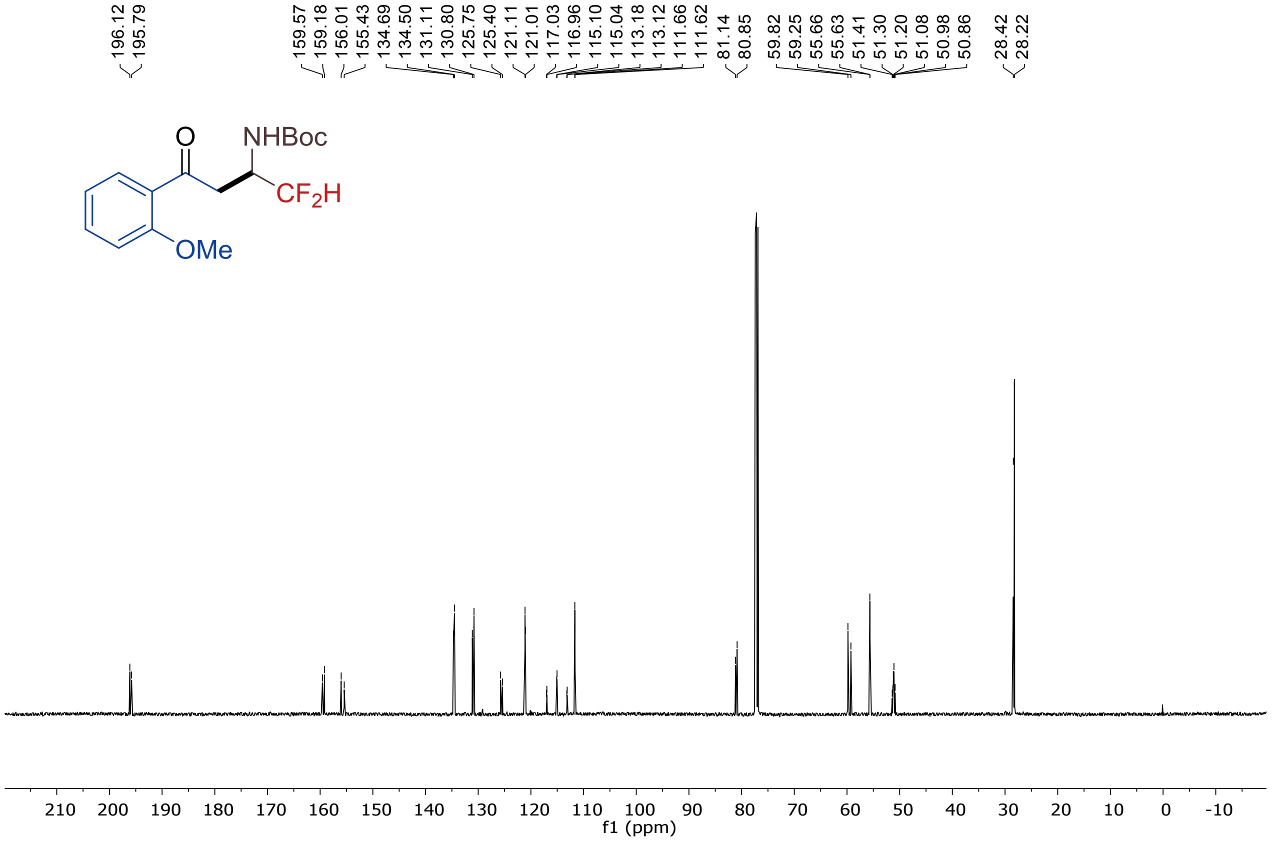

$^{13}C\{^1H\}$ NMR (126 MHz, $CDCl_3$) of compound **21**.

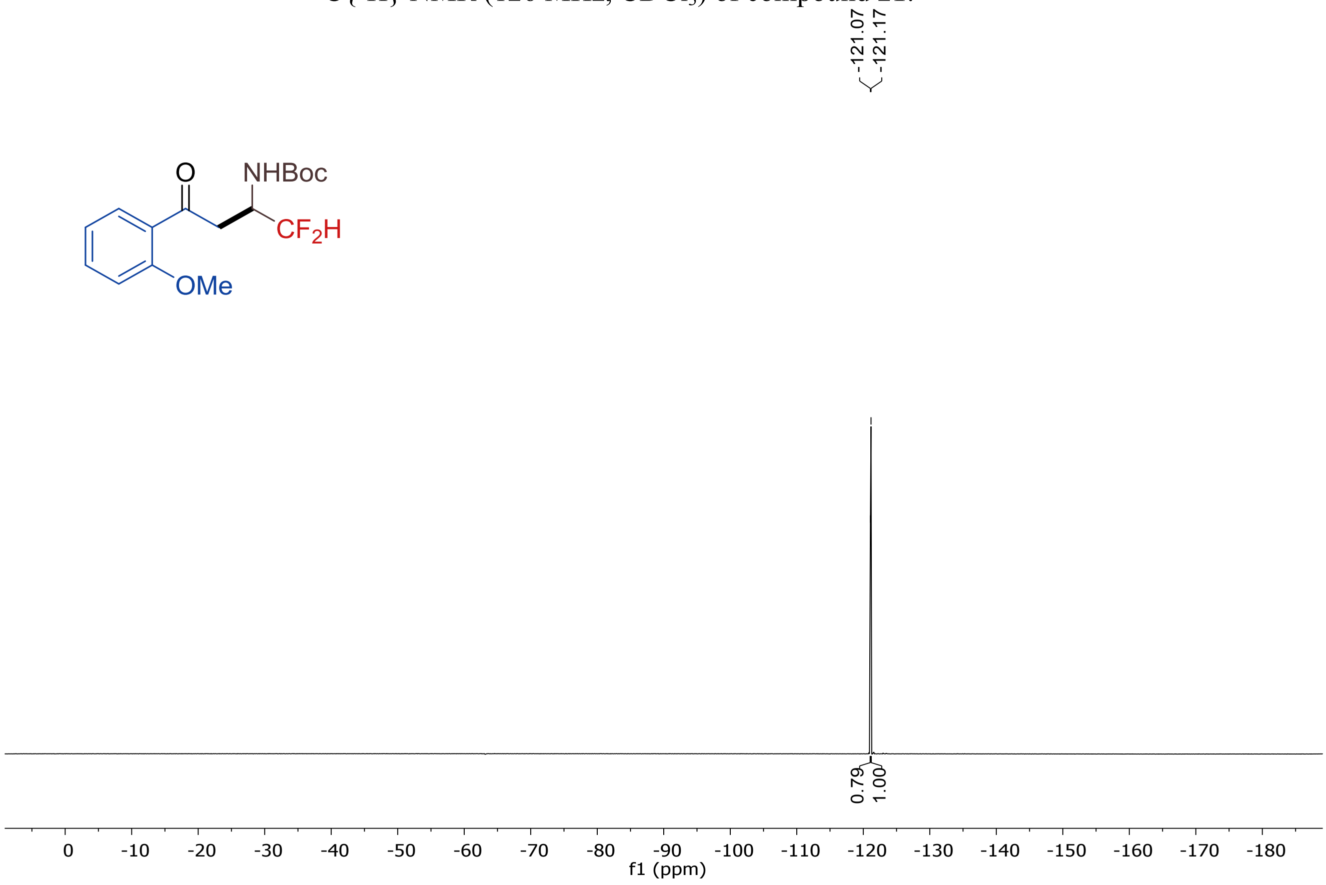

$^{19}F\{^1H\}$ NMR (377 MHz, $CDCl_3$) of compound **21**.

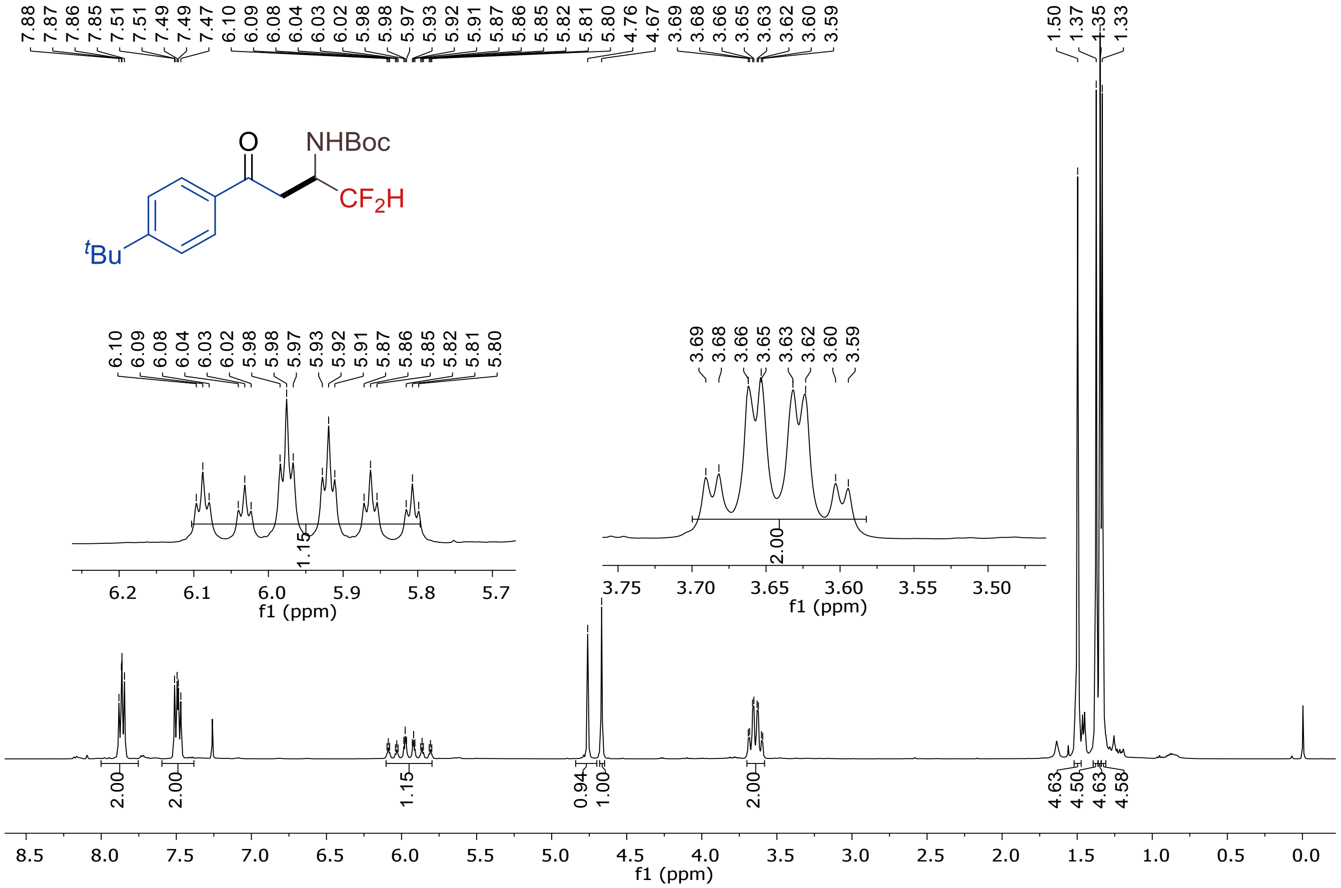

^{1}H NMR (500 MHz, $CDCl_3$) of compound **22**.

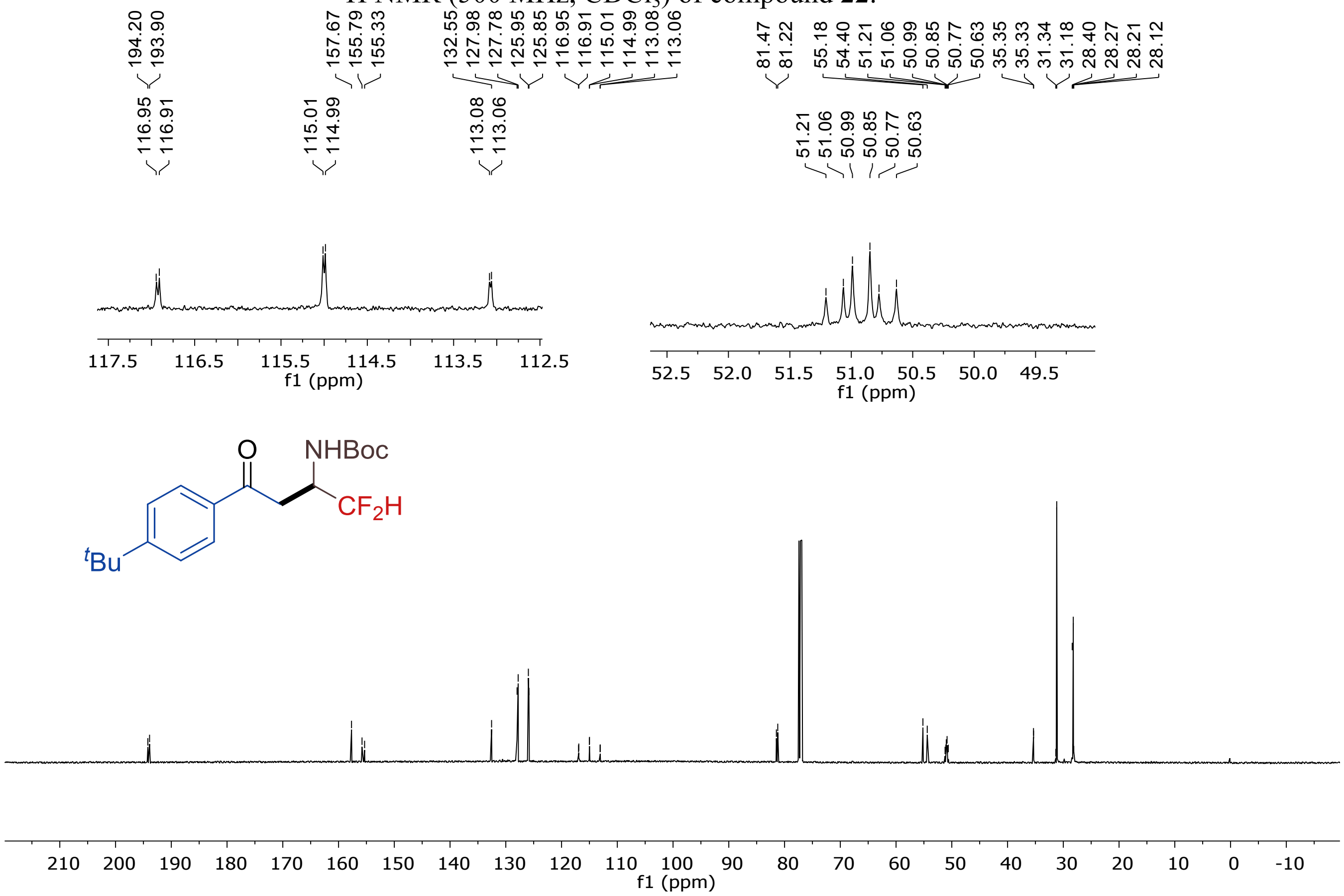

$^{13}C\{^{1}H\}$ NMR (151 MHz, $CDCl_3$) of compound **22**.

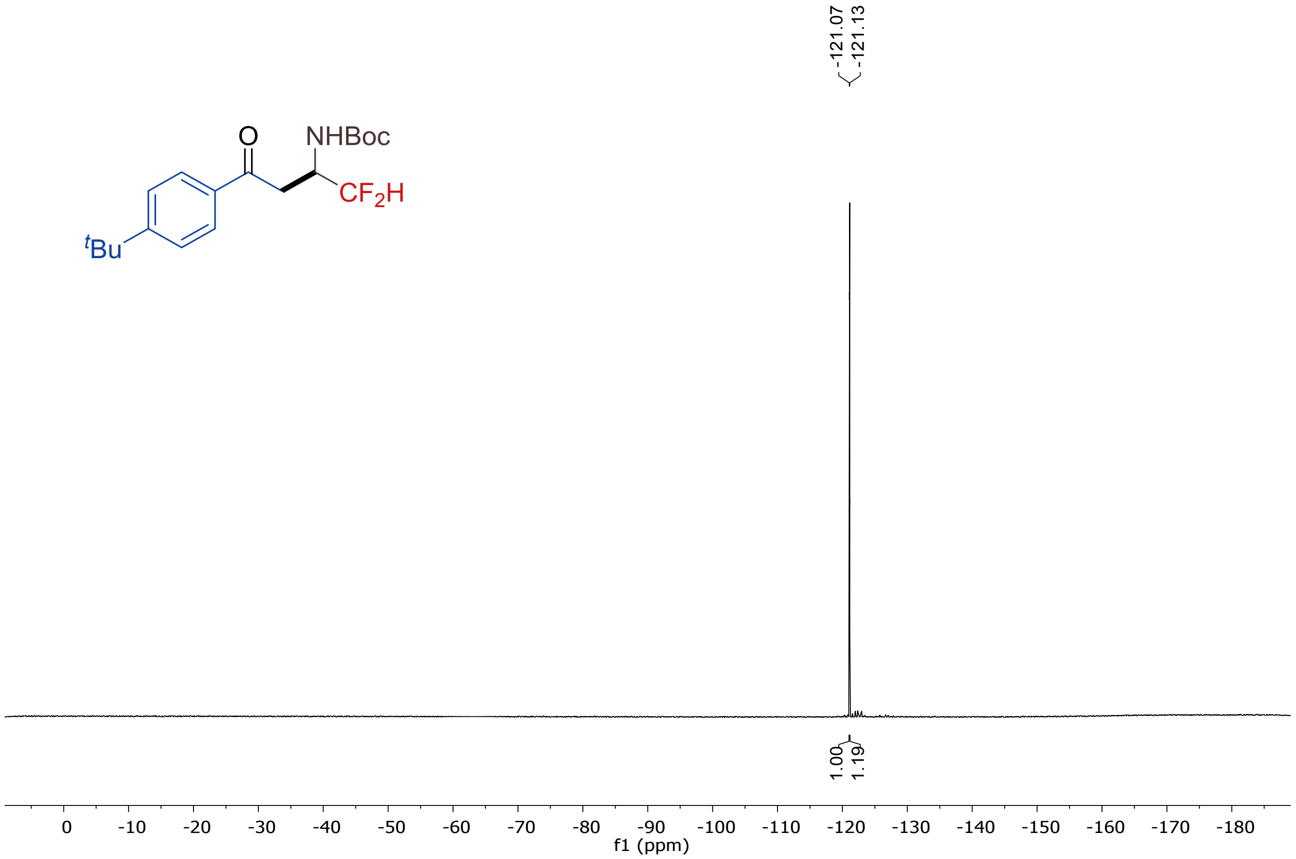

$^{19}F\{^{1}H\}$ NMR (377 MHz, $CDCl_3$) of compound **22**.

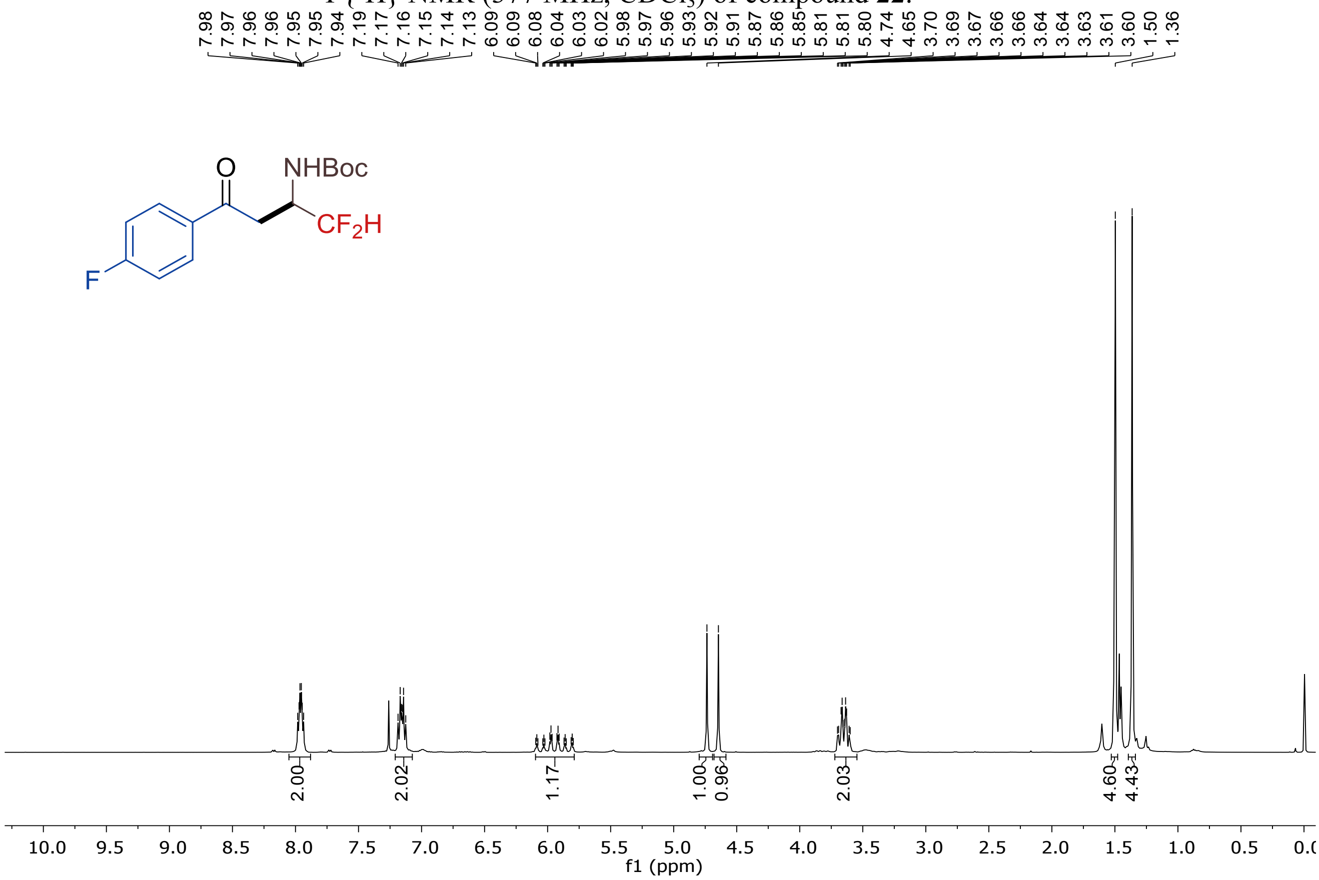

^{1}H NMR (500 MHz, $CDCl_3$) of compound **23**.

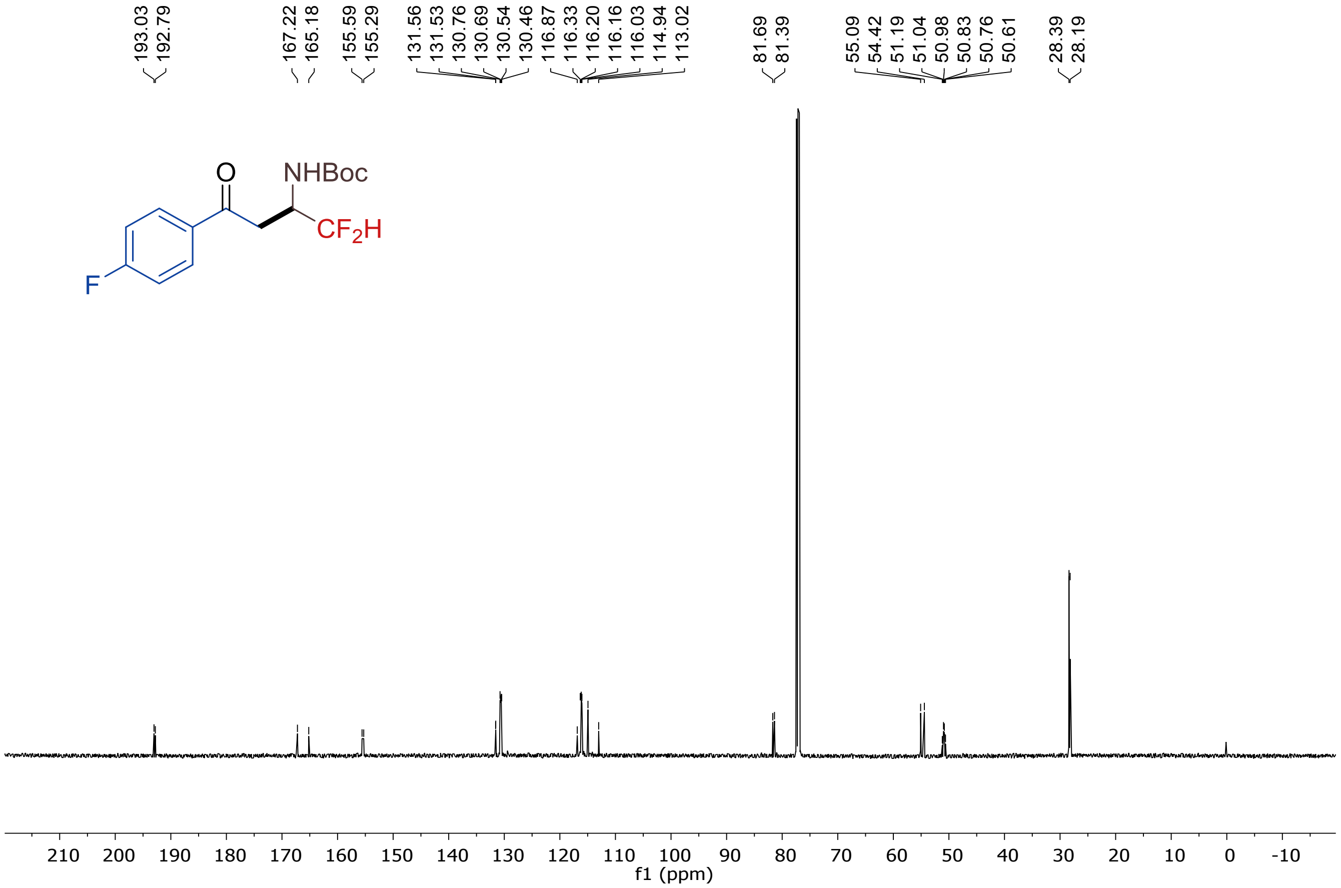

$^{13}C\{^{1}H\}$ NMR (126 MHz, $CDCl_3$) of compound **23**.

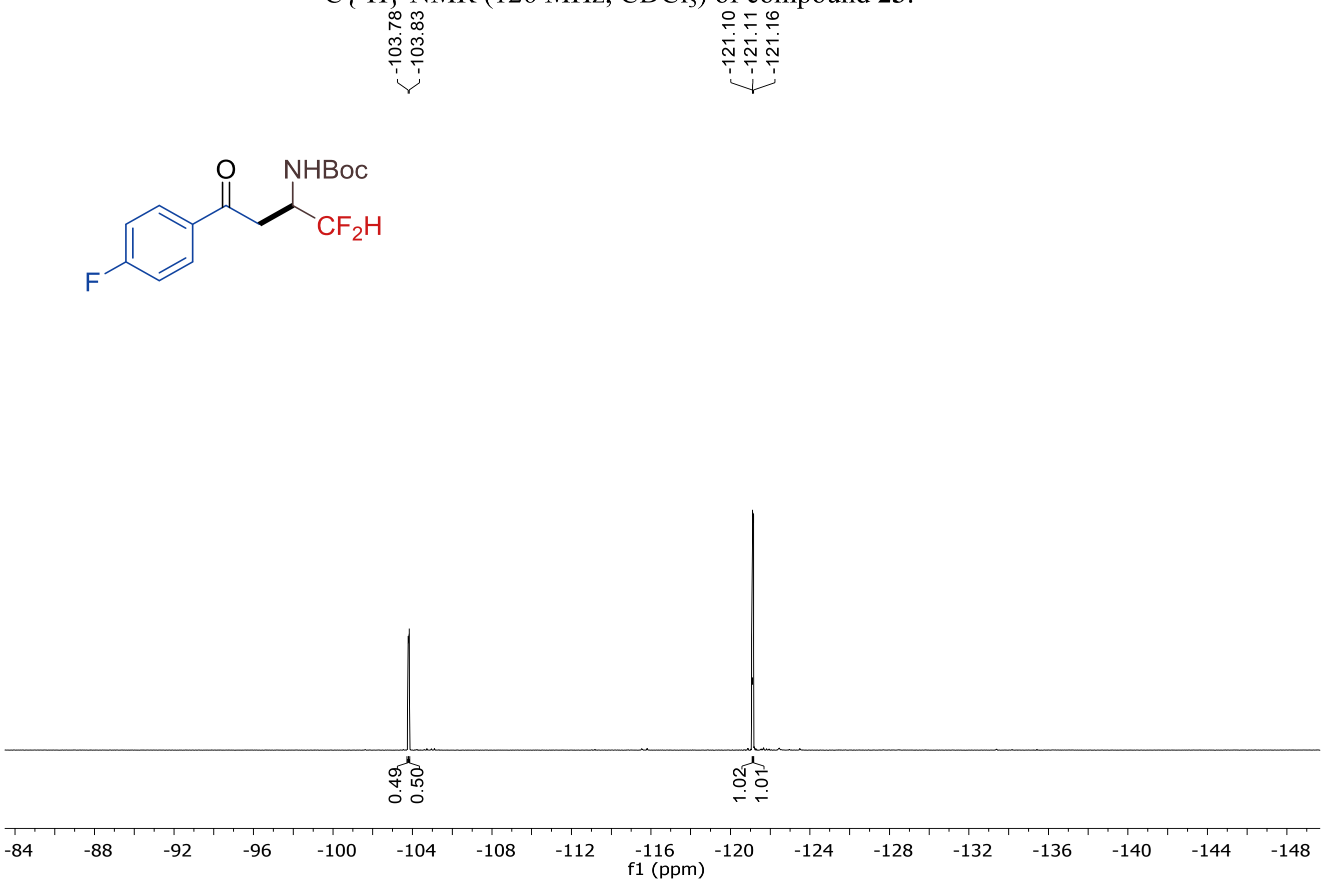

$^{19}F\{^{1}H\}$ NMR (377 MHz, $CDCl_3$) of compound **23**.

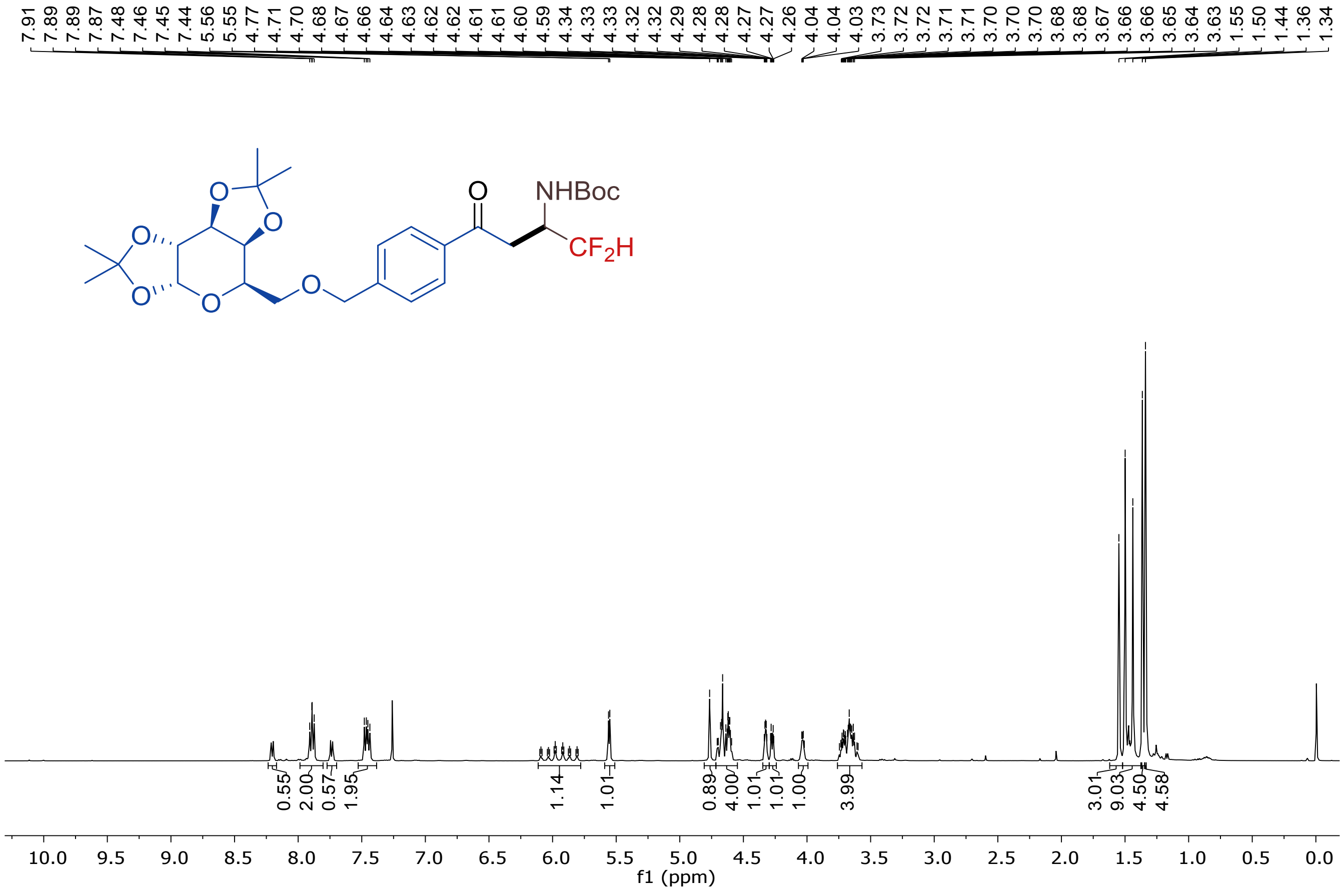

^{1}H NMR (600 MHz, $CDCl_3$) of compound **24**.

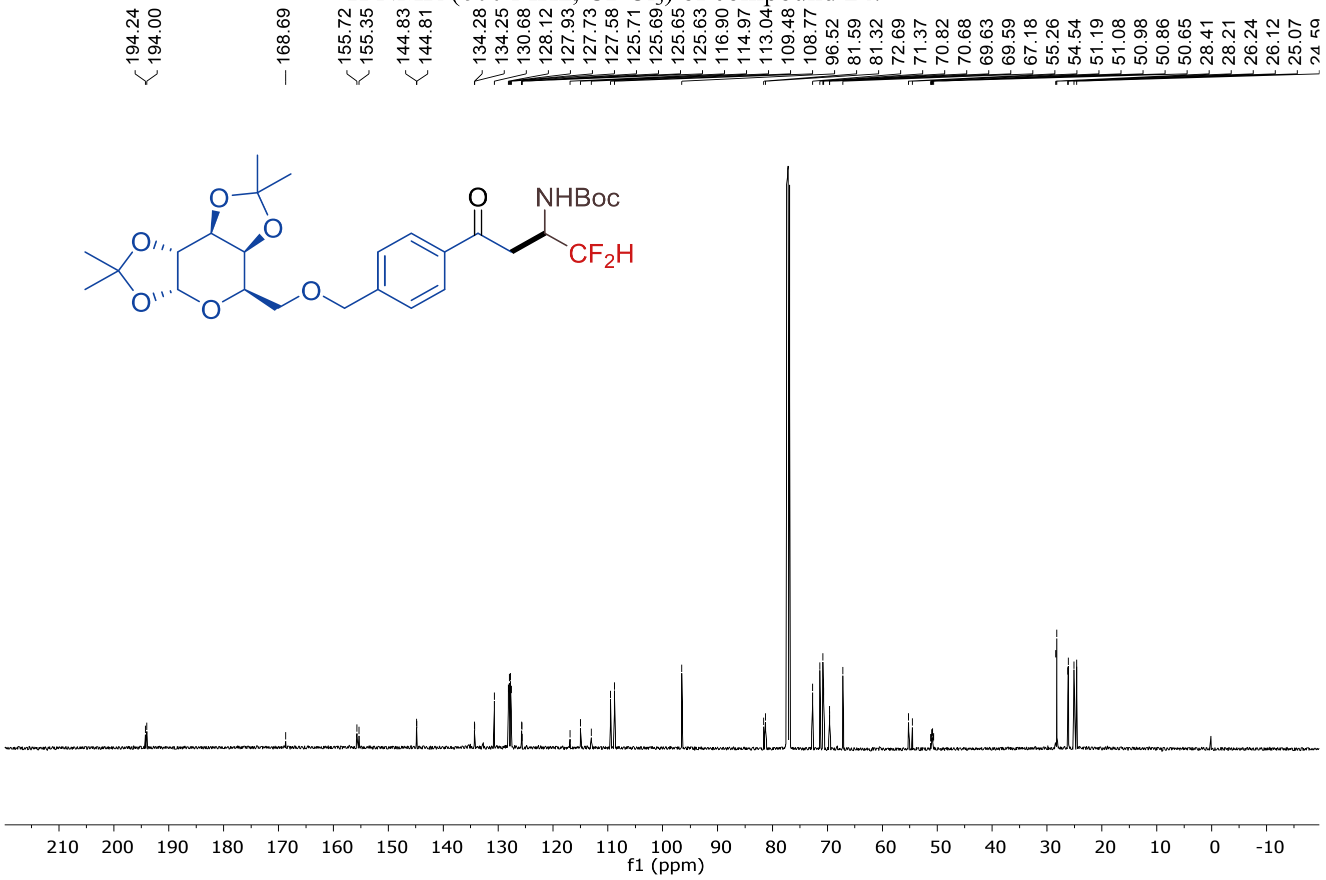

$^{13}C\{^{1}H\}$ NMR (126 MHz, $CDCl_3$) of compound **24**.

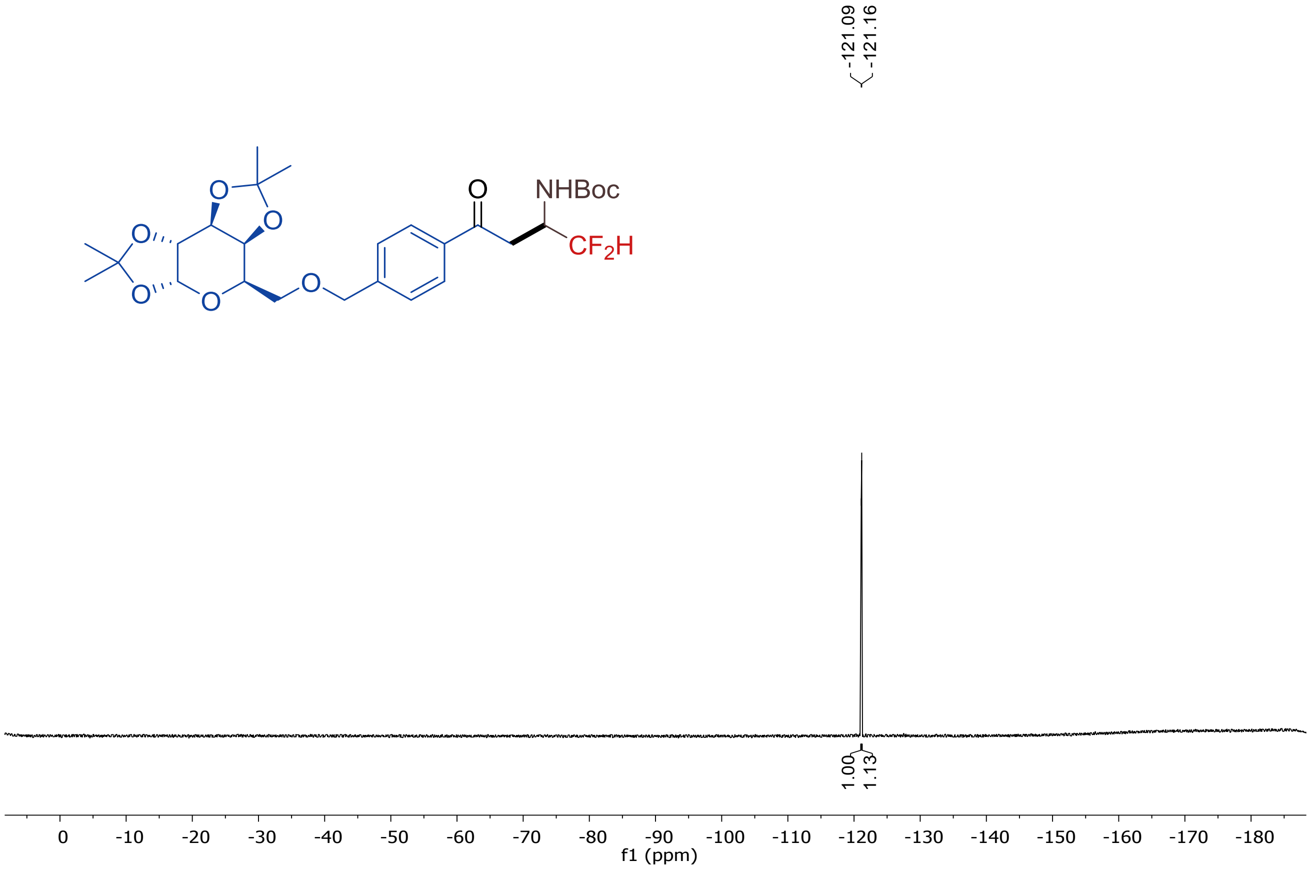

$^{19}F\{^1H\}$ NMR (282 MHz, $CDCl_3$) of compound **24**.

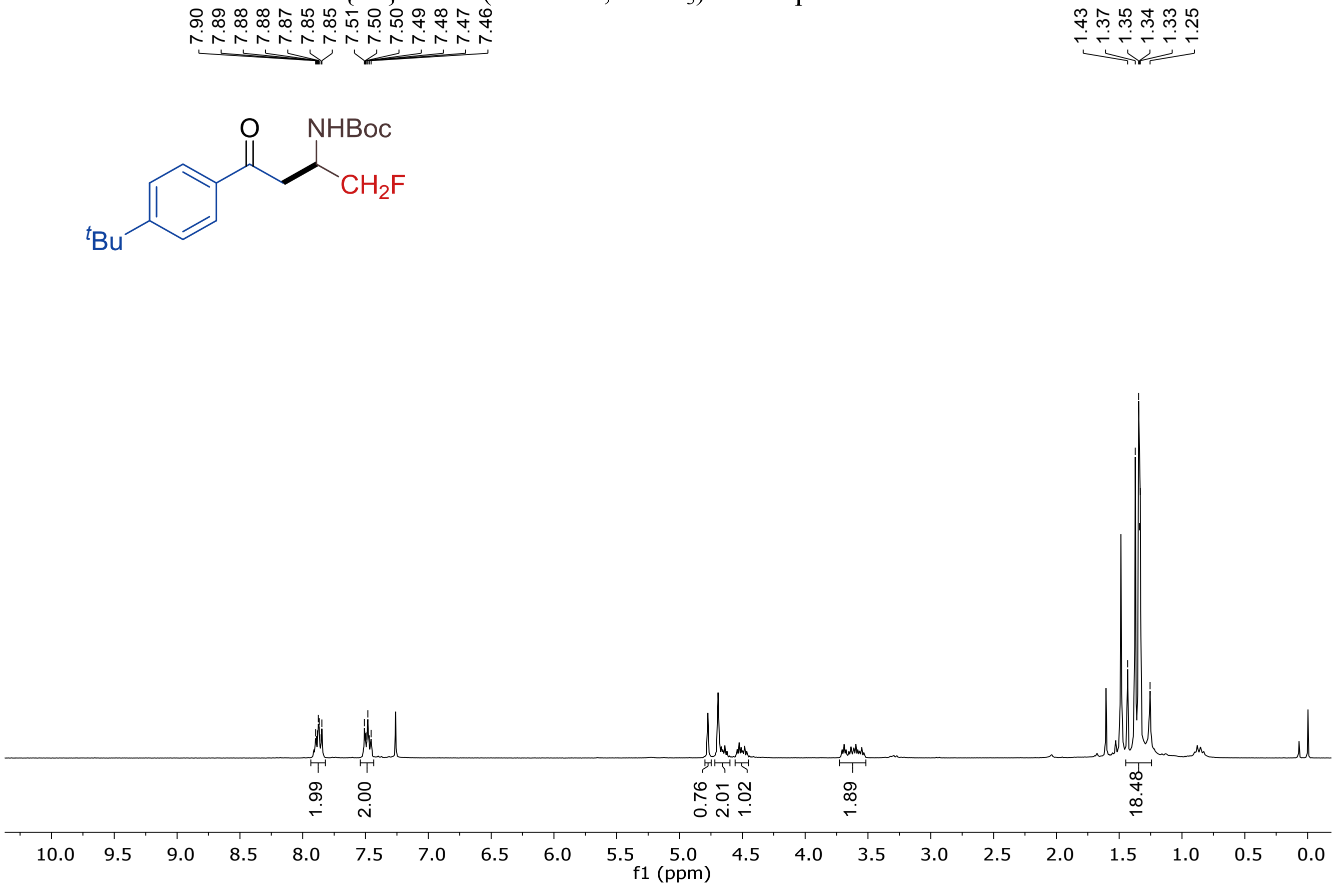

^1H NMR (300 MHz, $CDCl_3$) of compound **25**.

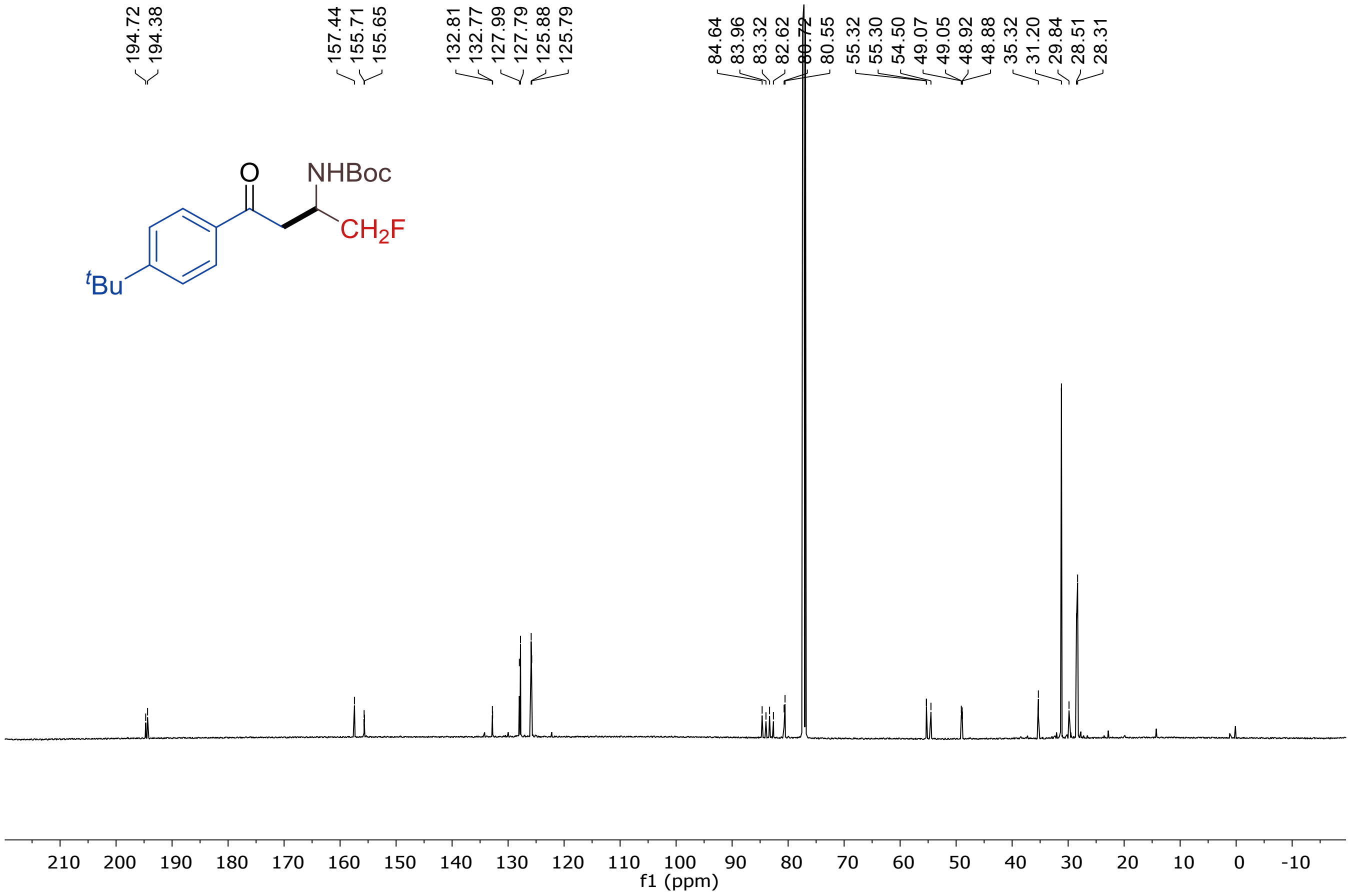

$^{13}C\{^{1}H\}$ NMR (126 MHz, $CDCl_3$) of compound **25**.

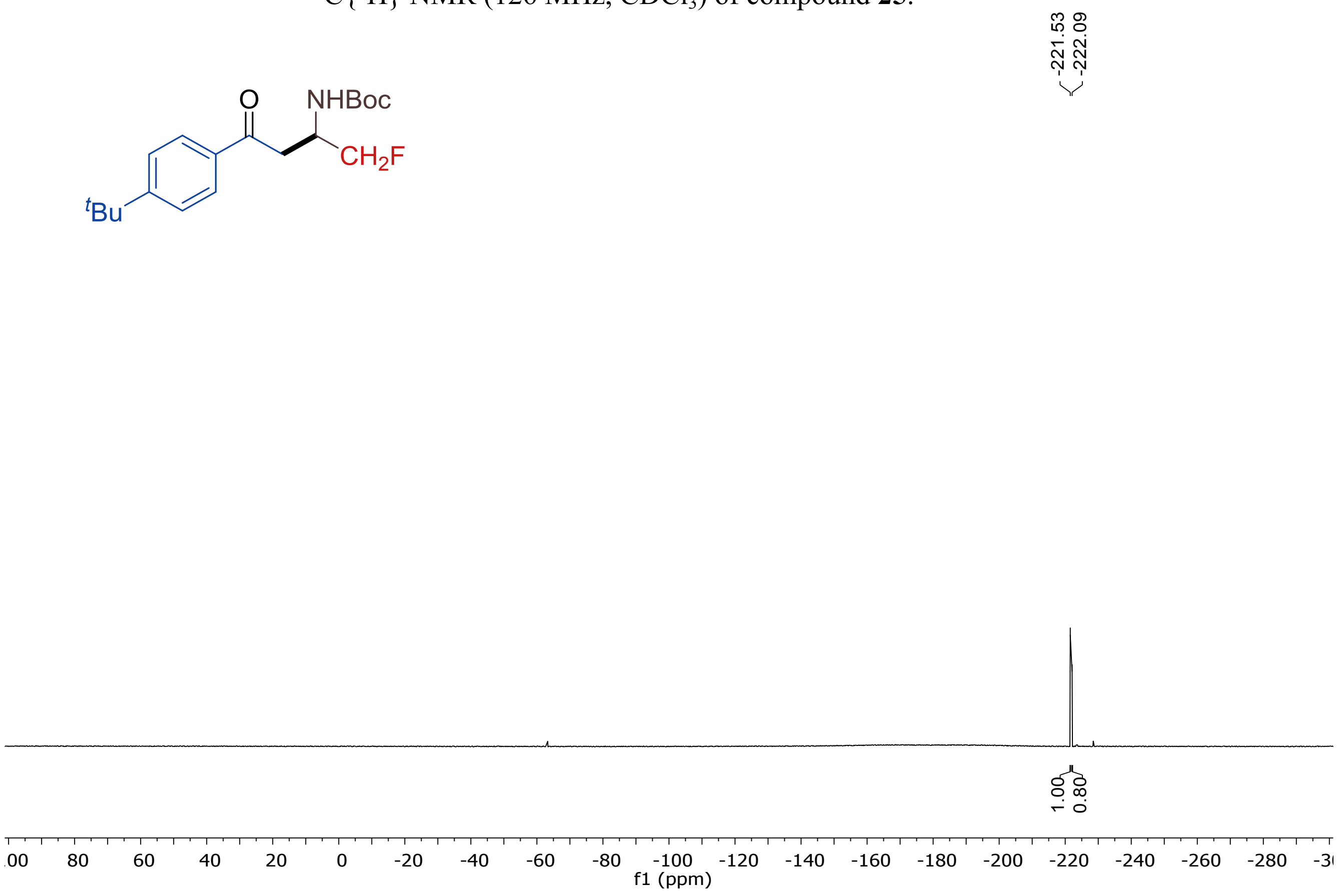

$^{19}F\{^{1}H\}$ NMR (282 MHz, $CDCl_3$) of compound **25**.

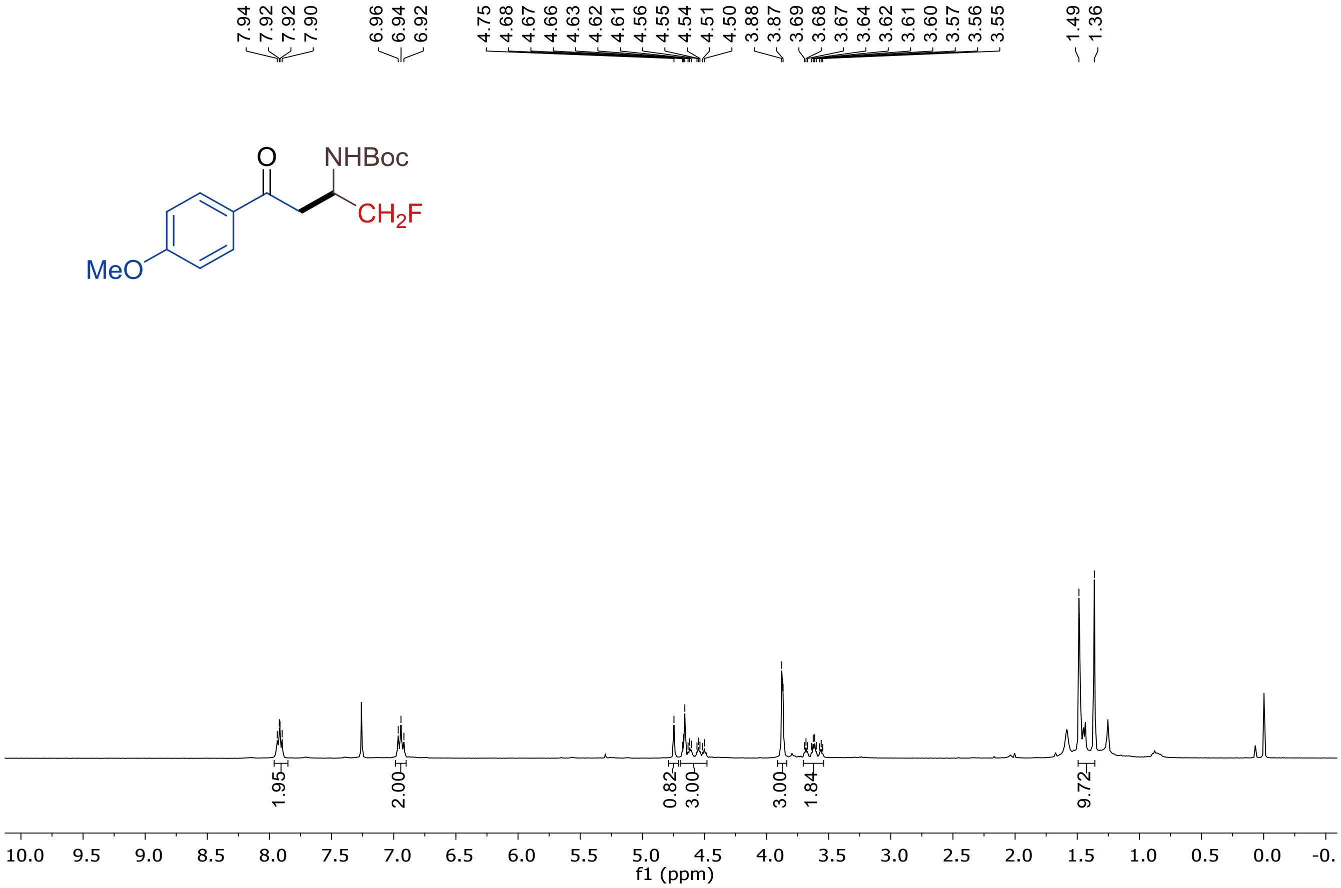

^{1}H NMR (400 MHz, $CDCl_3$) of compound **26**.

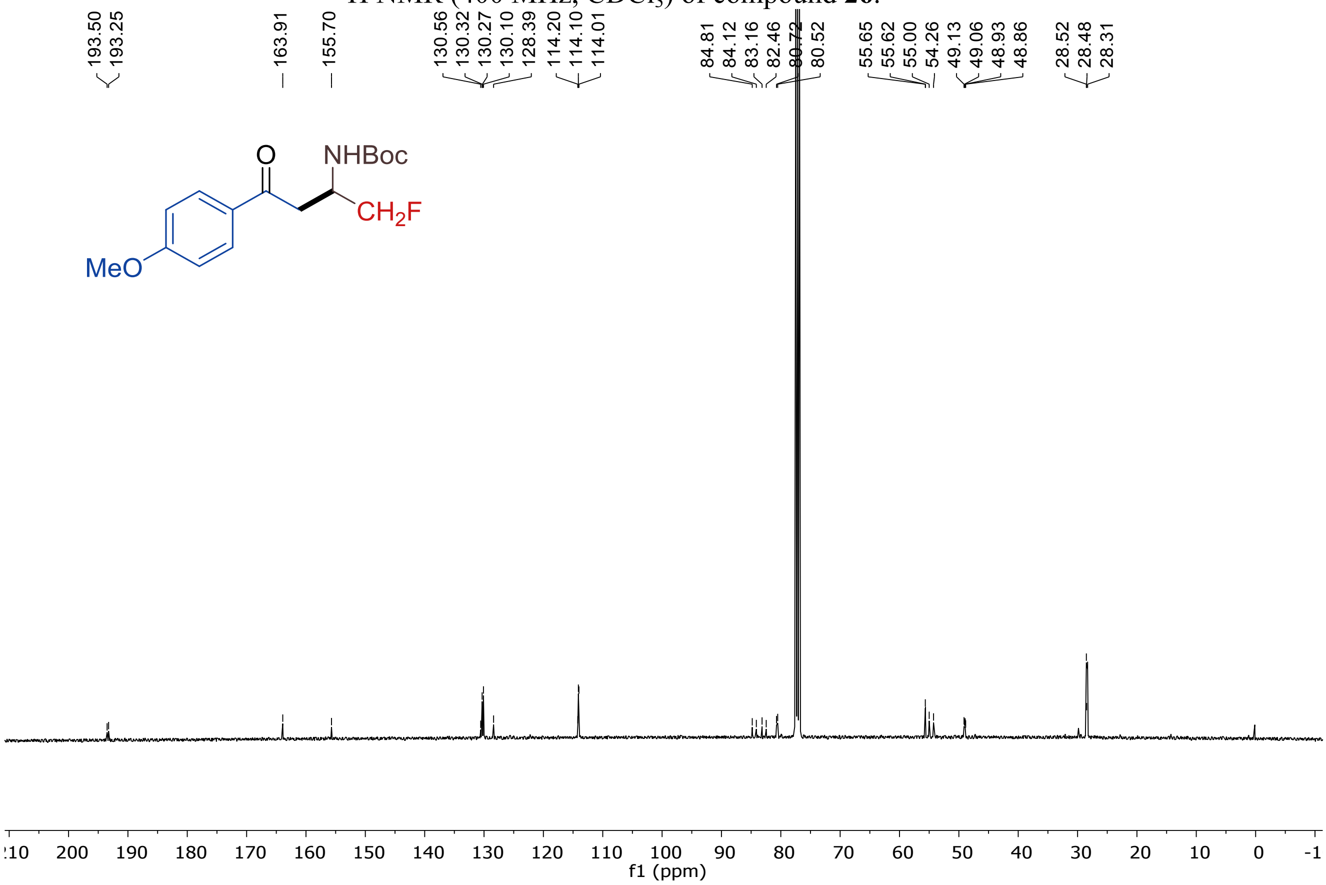

$^{13}C\{^{1}H\}$ NMR (101 MHz, $CDCl_3$) of compound **26**.

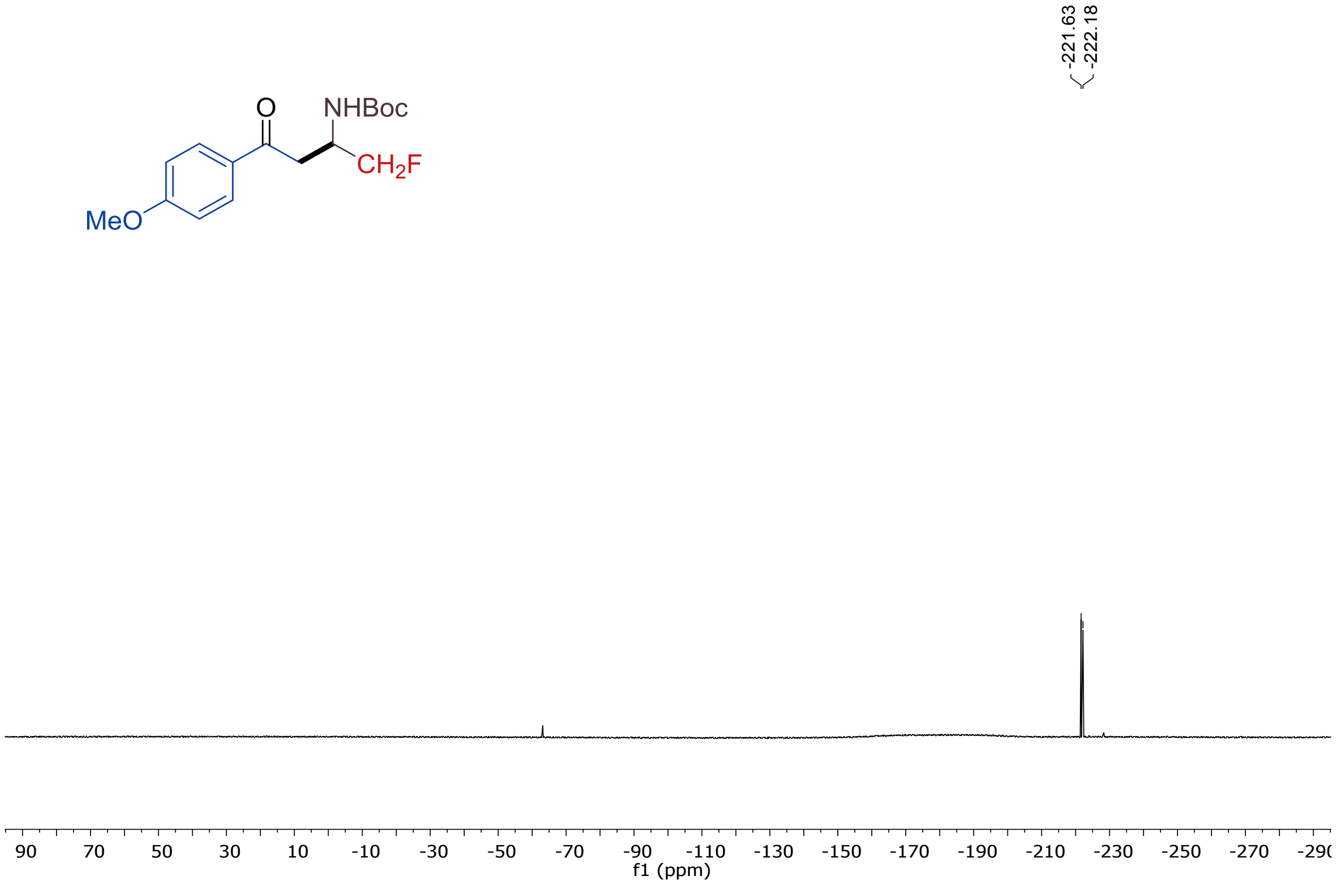

$^{19}F\{^1H\}$ NMR (377 MHz, $CDCl_3$) of compound **26**.

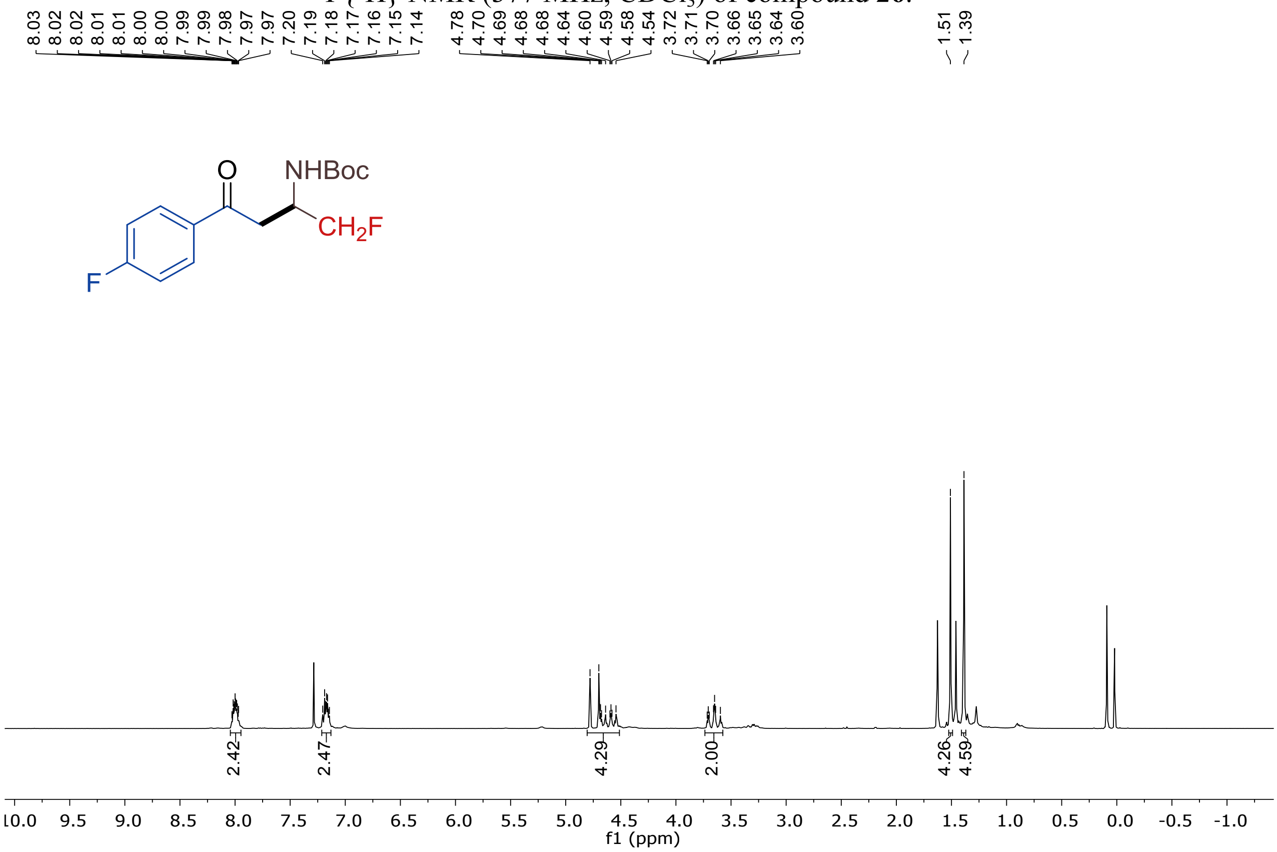

^1H NMR (500 MHz, $CDCl_3$) of compound **27**.

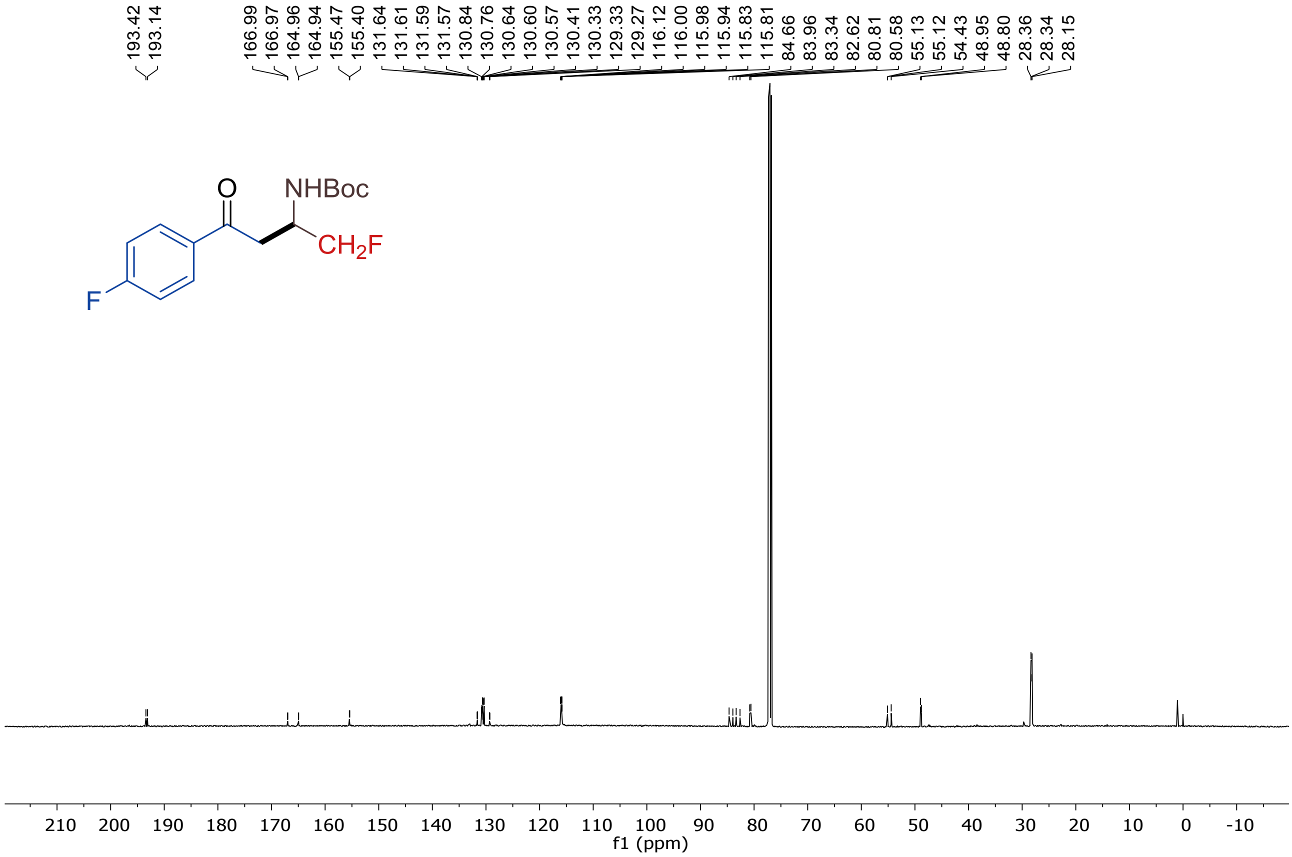

$^{13}C\{^{1}H\}$ NMR (126 MHz, $CDCl_3$) of compound **27**.

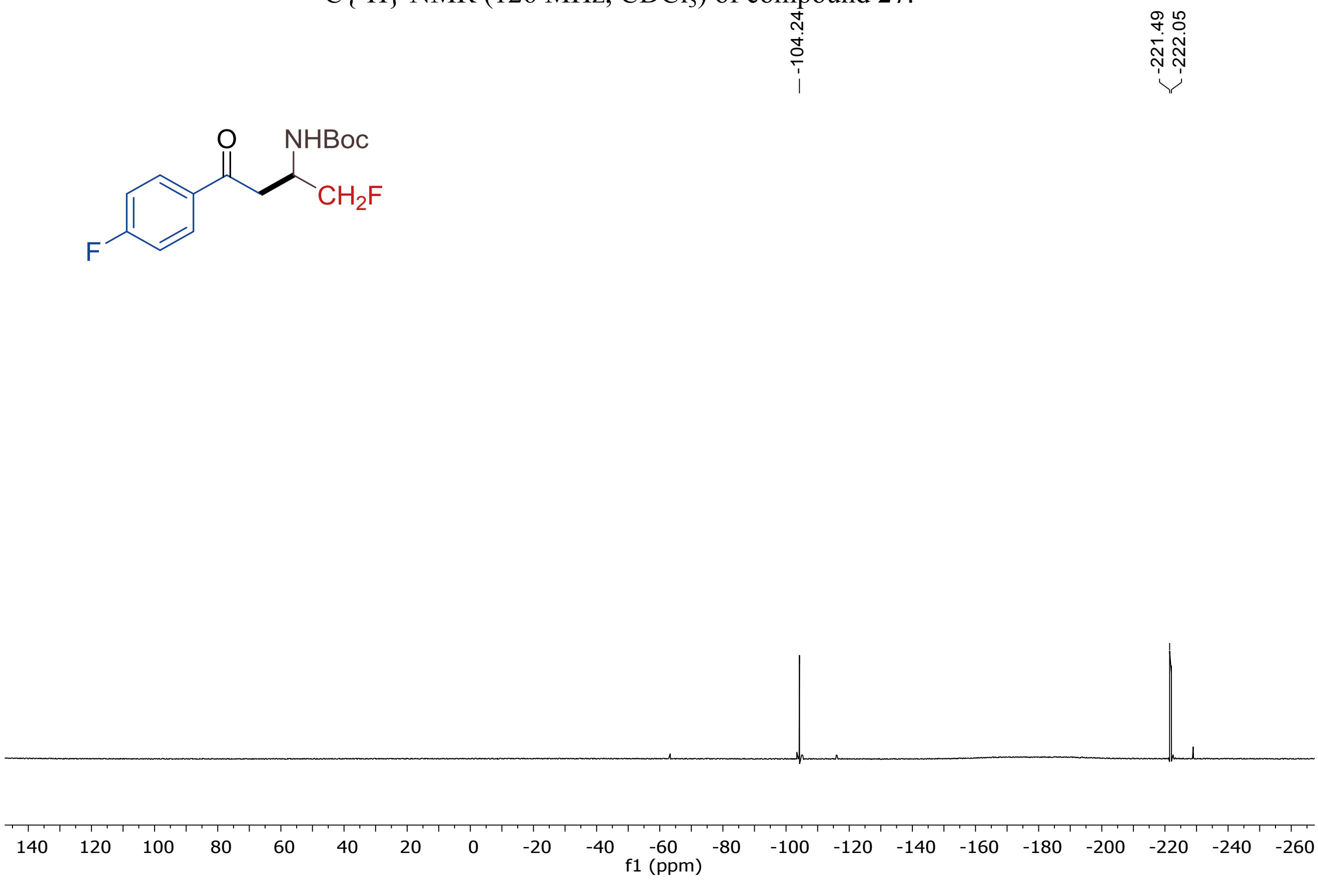

$^{19}F\{^{1}H\}$ NMR (377 MHz, $CDCl_3$) of compound **27**.

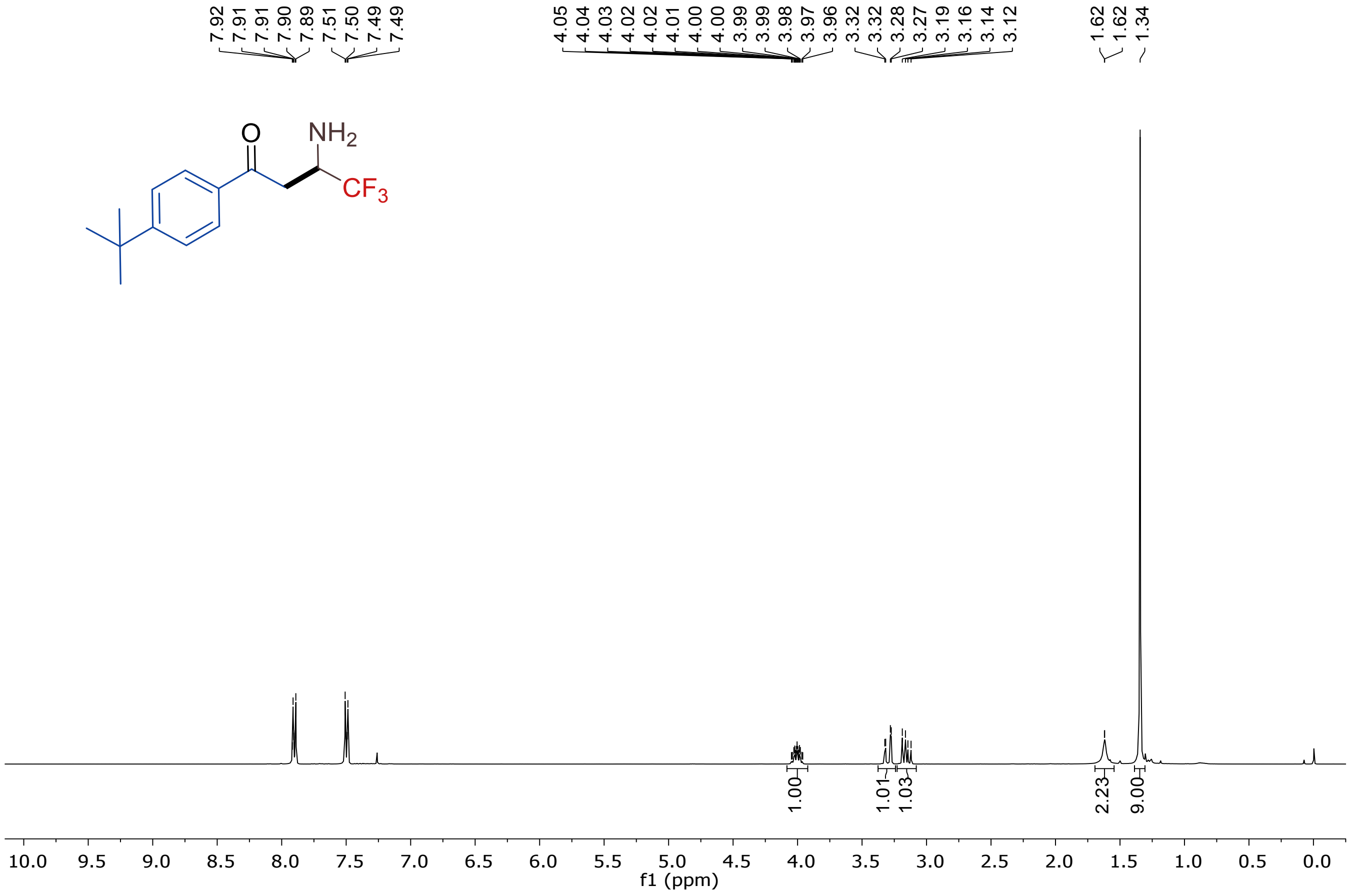

^{1}H NMR (400 MHz, $CDCl_3$) of compound **28**.

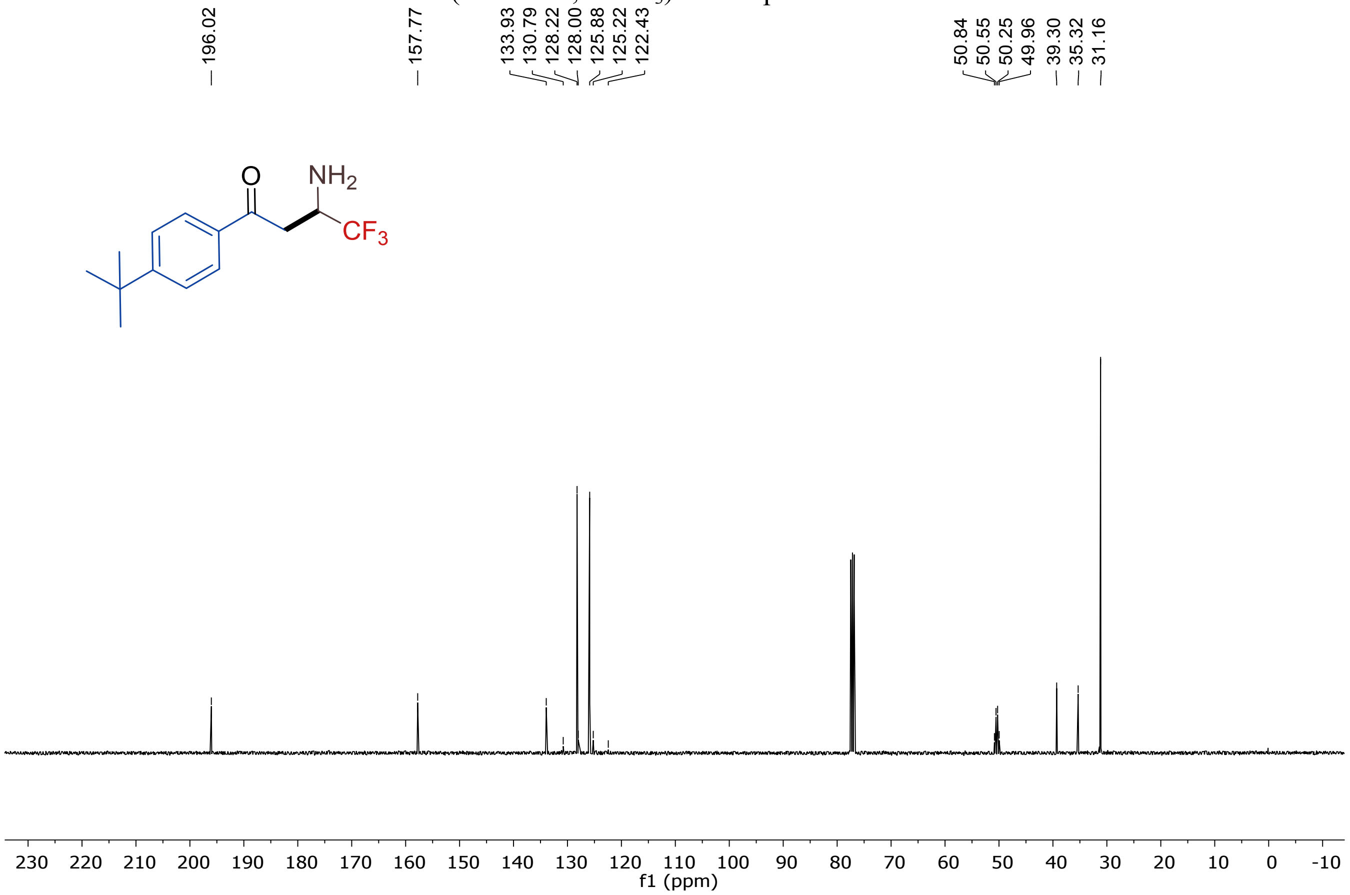

$^{13}C\{^{1}H\}$ NMR (101 MHz, $CDCl_3$) of compound **28**.

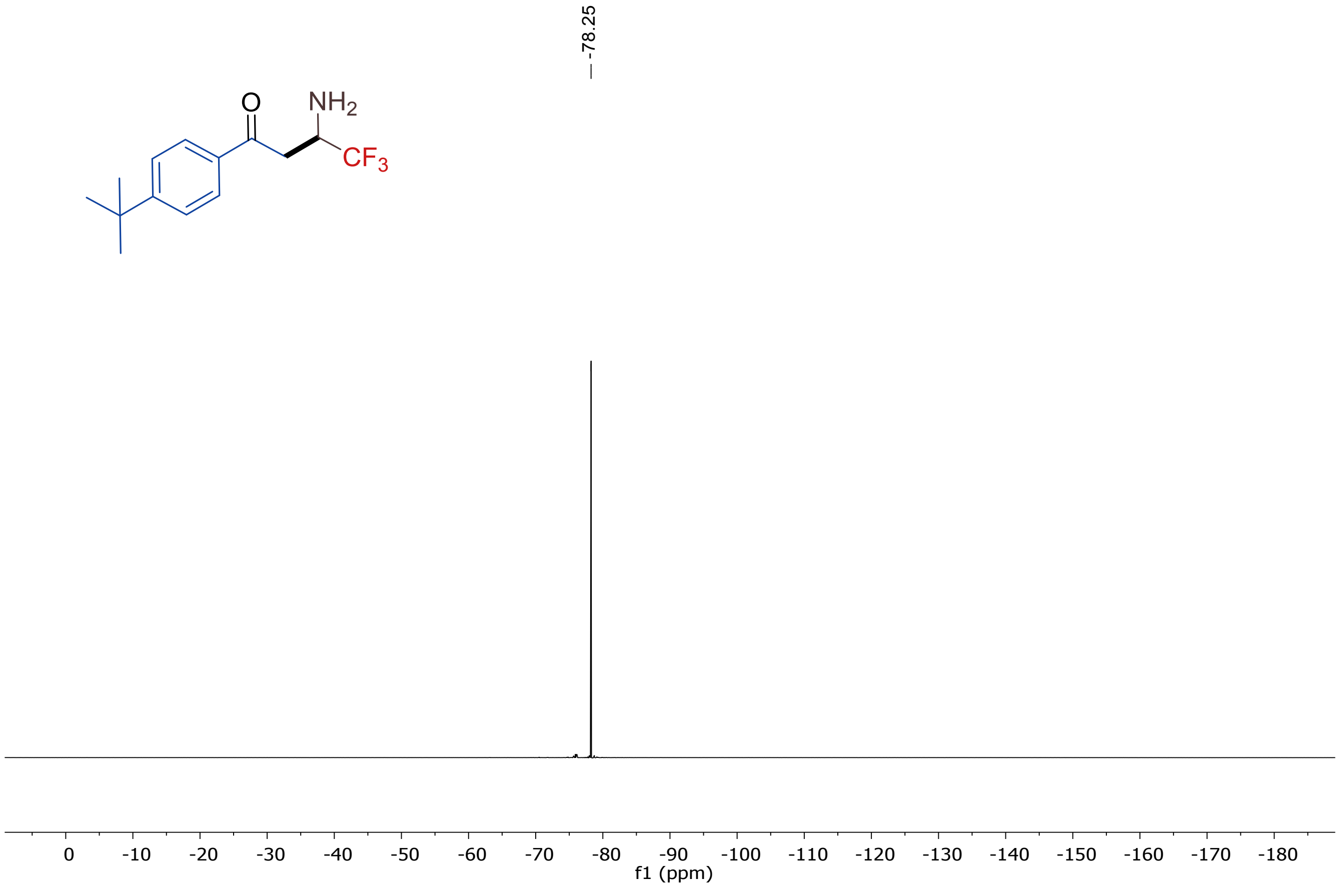

$^{19}F\{^{1}H\}$ NMR (377 MHz, $CDCl_3$) of compound **28**.

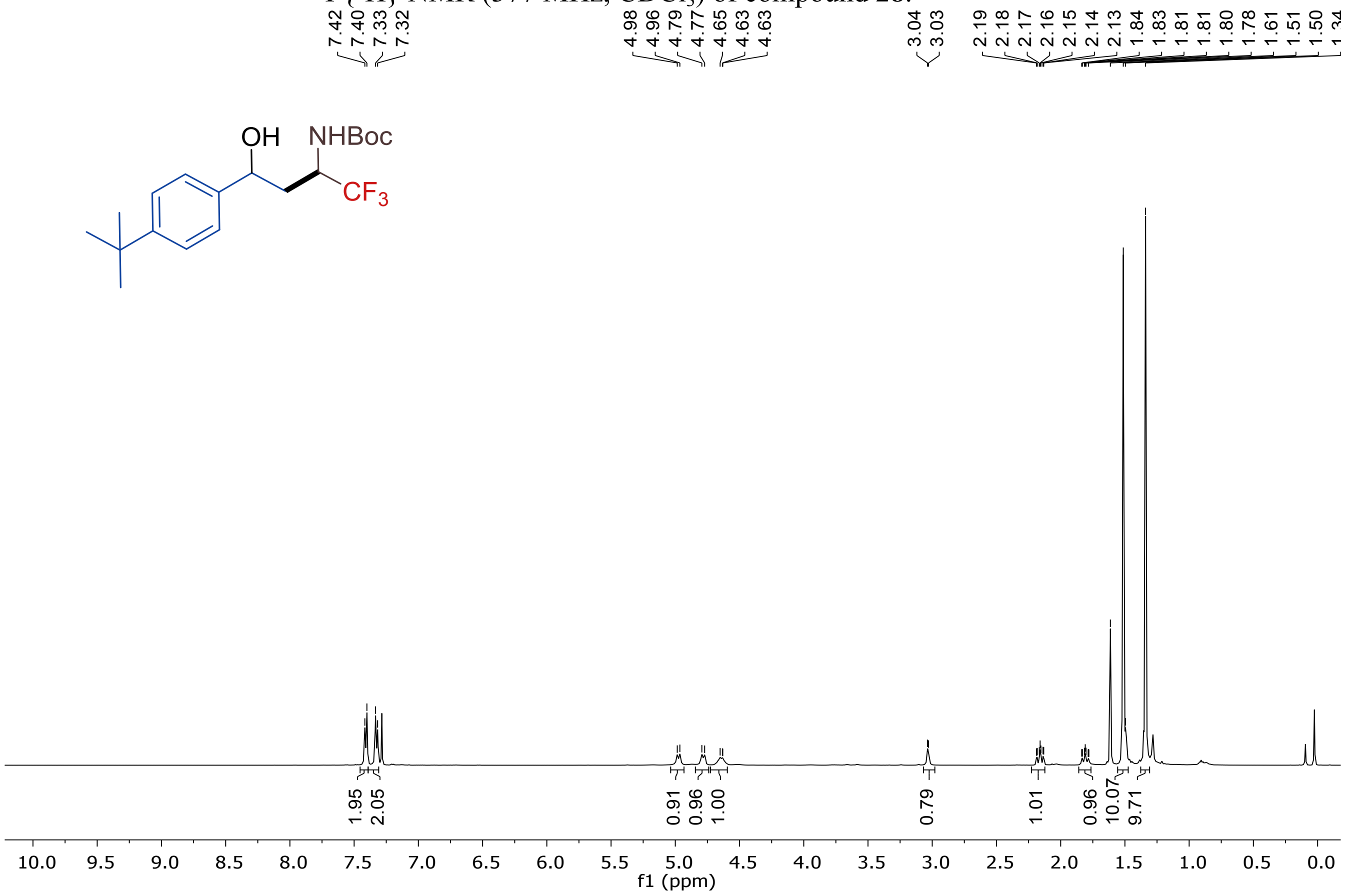

^{1}H NMR (500 MHz, $CDCl_3$) of compound **29-d1**.

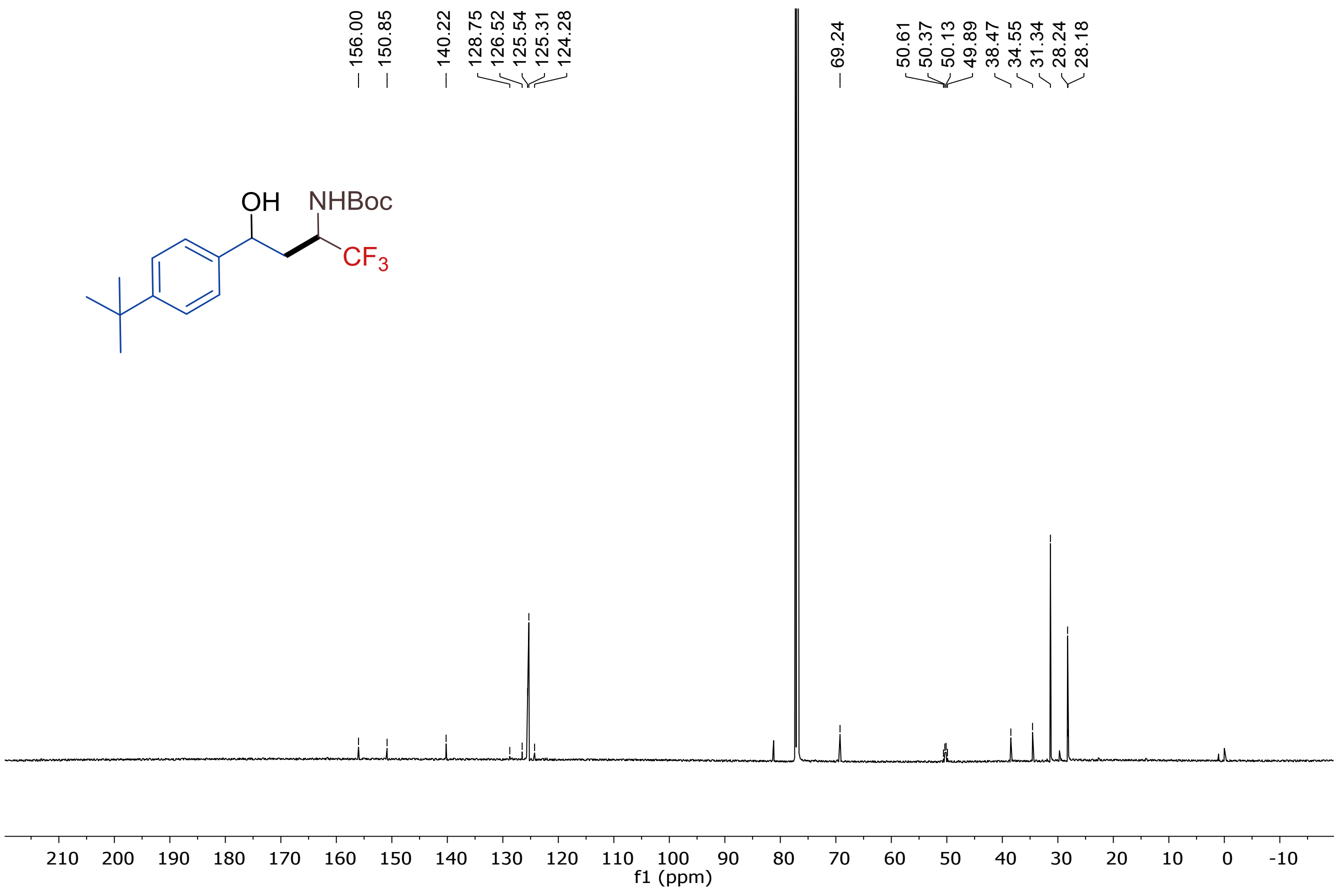

$^{13}C\{^{1}H\}$ NMR (126 MHz, $CDCl_3$) of compound **29-d1**.

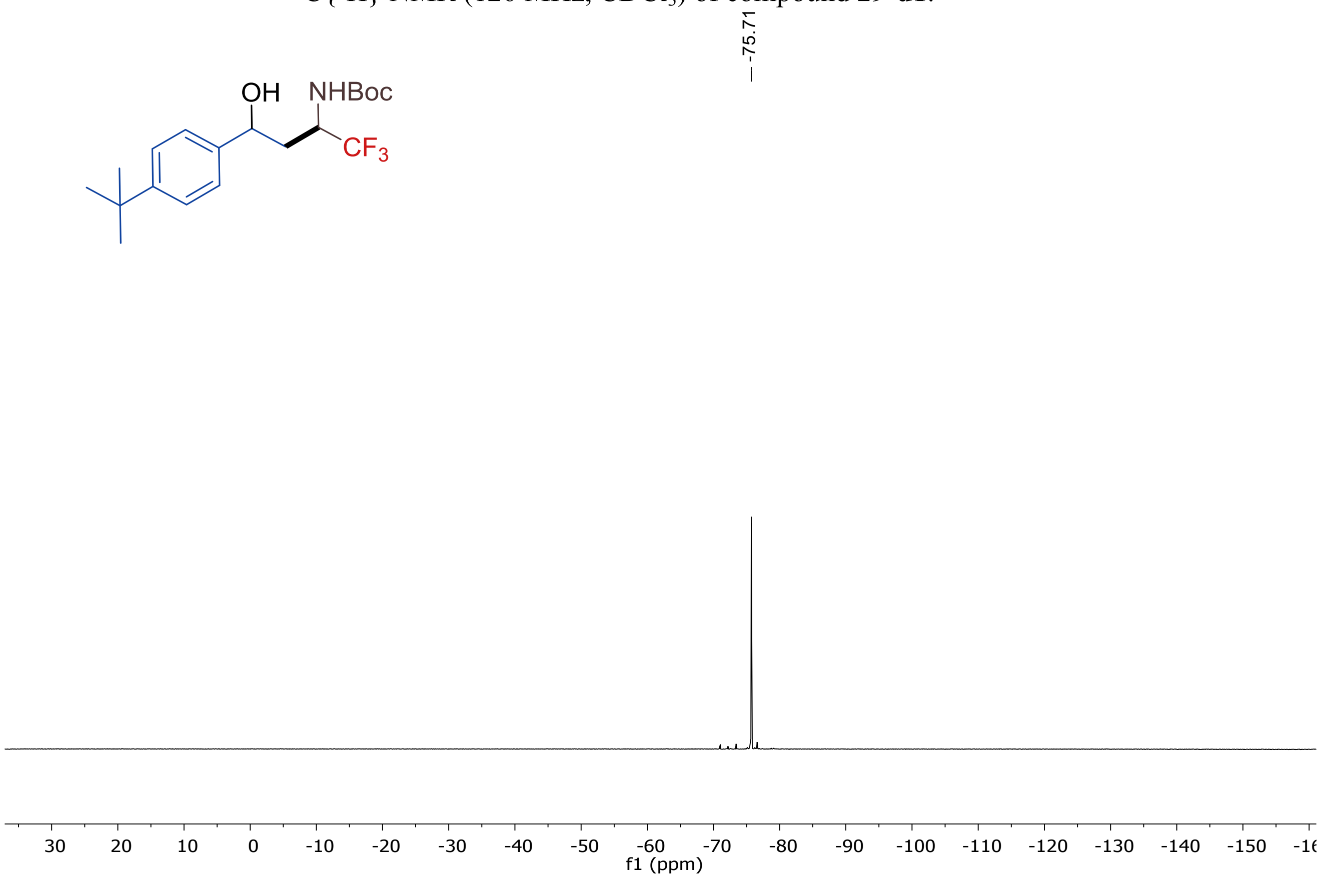

$^{19}F\{^{1}H\}$ NMR (377 MHz, $CDCl_3$) of compound **29-d1**.

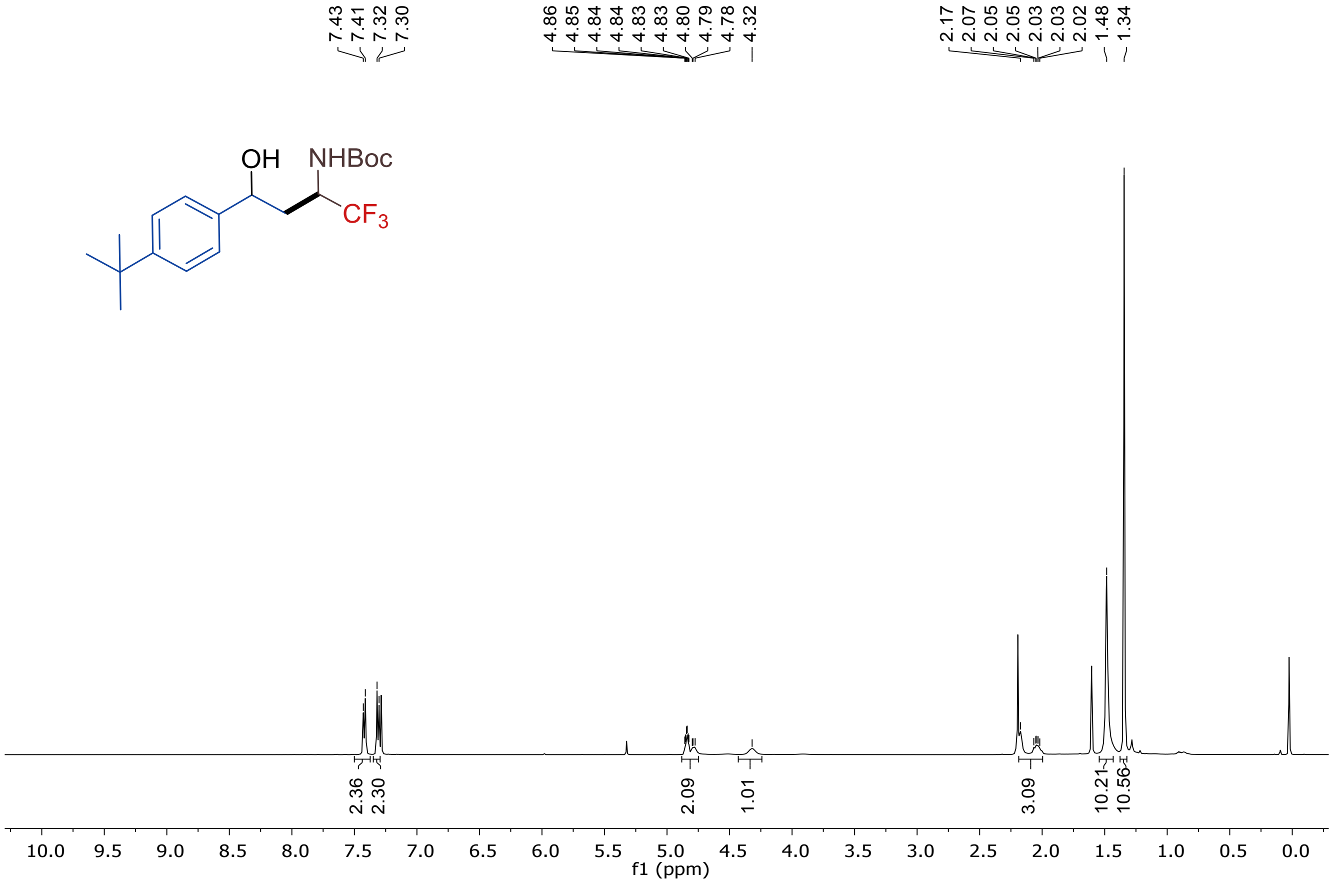

^{1}H NMR (500 MHz, $CDCl_3$) of compound **29-d2**.

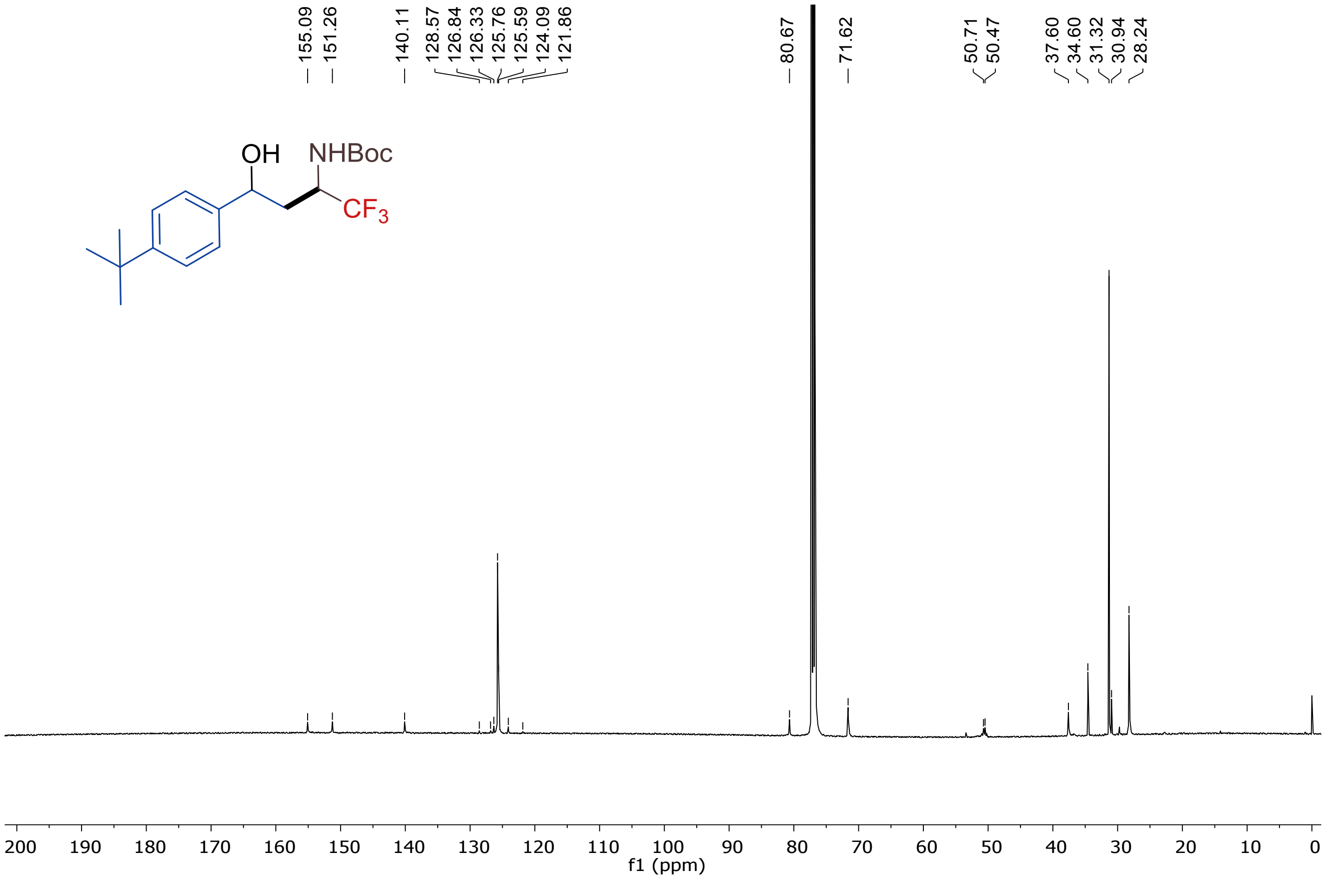

$^{13}C\{^{1}H\}$ NMR (126 MHz, $CDCl_3$) of compound **29-d2**.

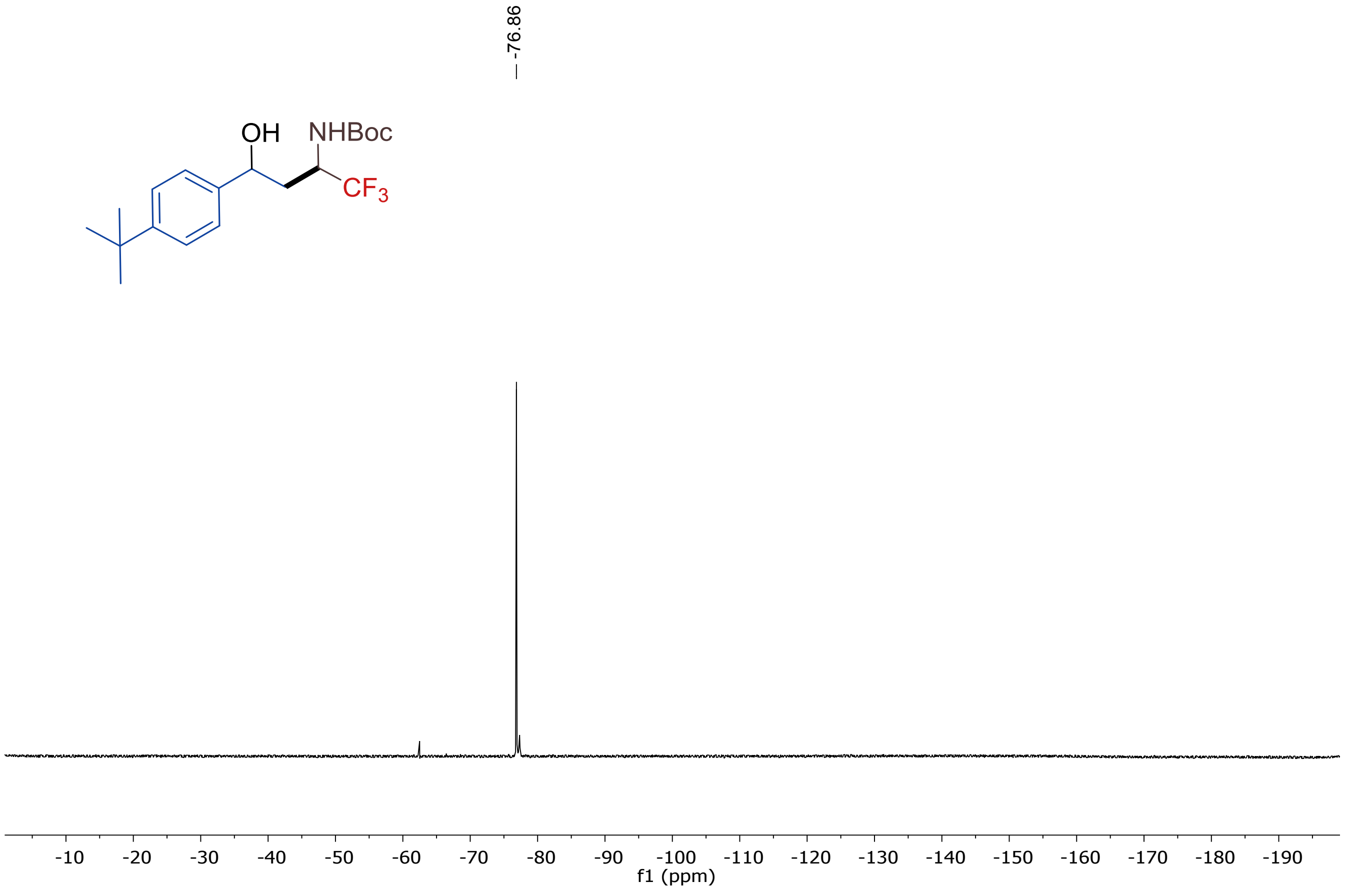

^{19}F{^{1}H} NMR (377 MHz, $CDCl_3$) of compound **29-d2**.

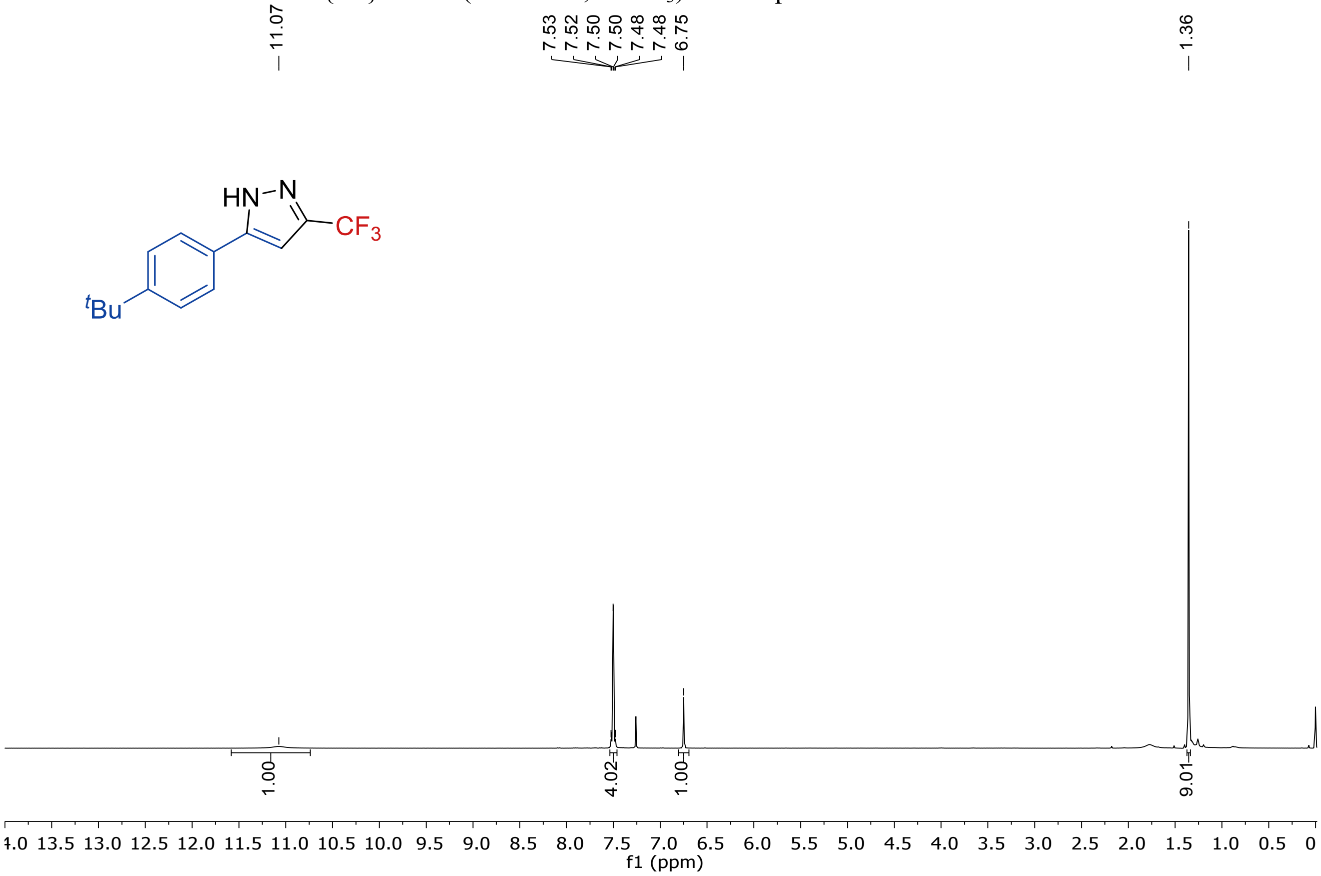

^{1}H NMR (400 MHz, $CDCl_3$) of compound **30**.

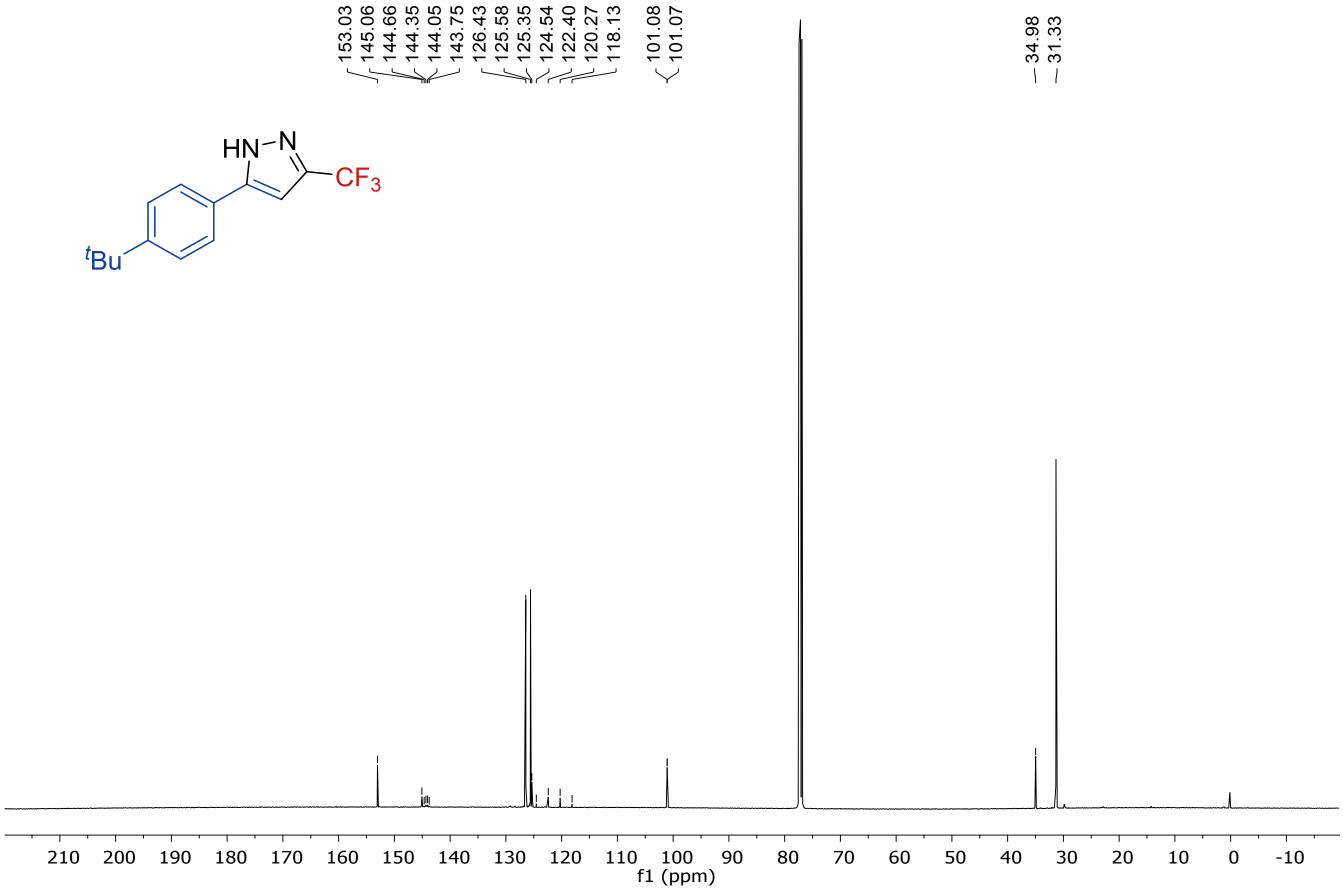

$^{13}C\{^1H\}$ NMR (126 MHz, $CDCl_3$) of compound **30**.

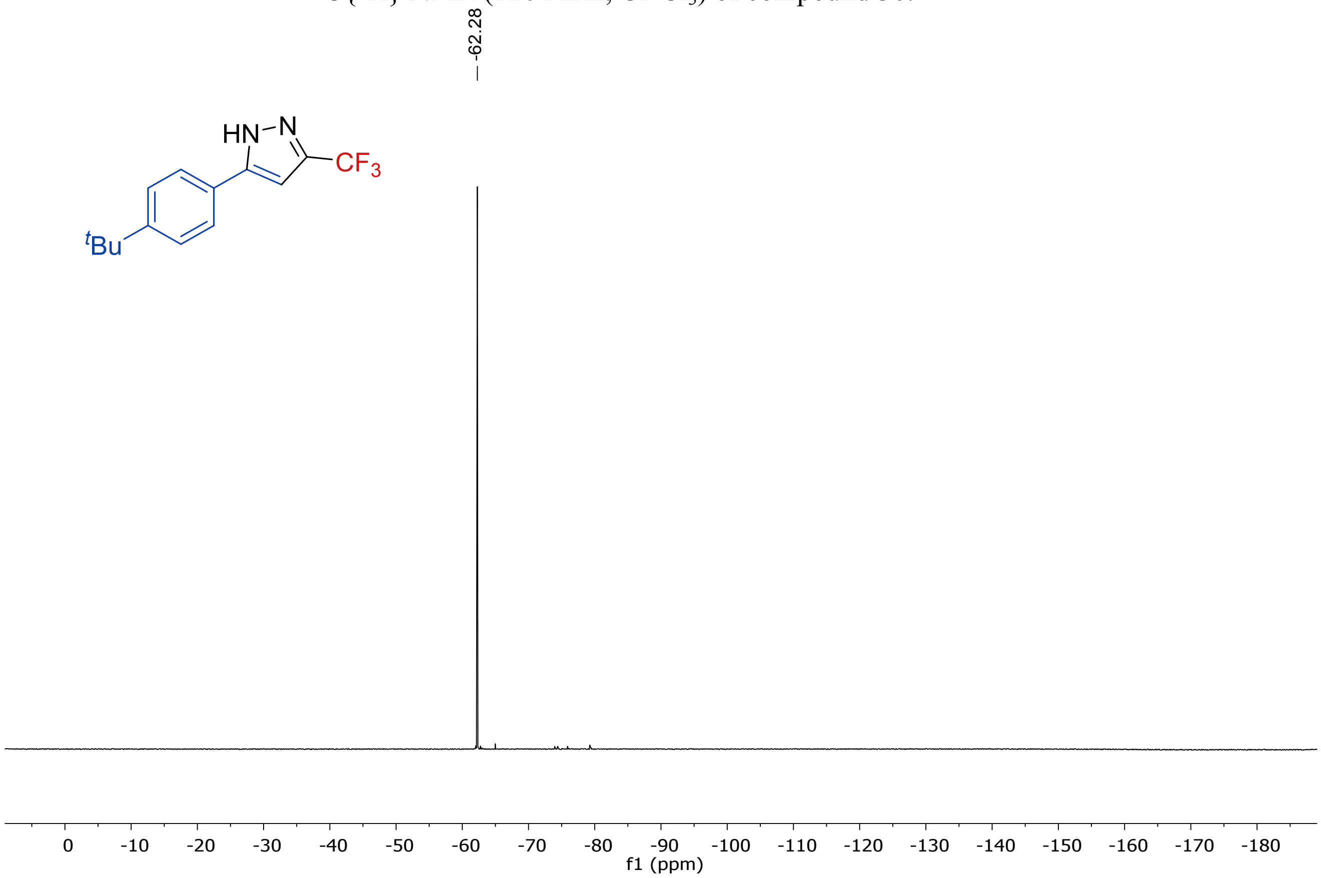

$^{19}F\{^1H\}$ NMR (377 MHz, $CDCl_3$) of compound **30**.

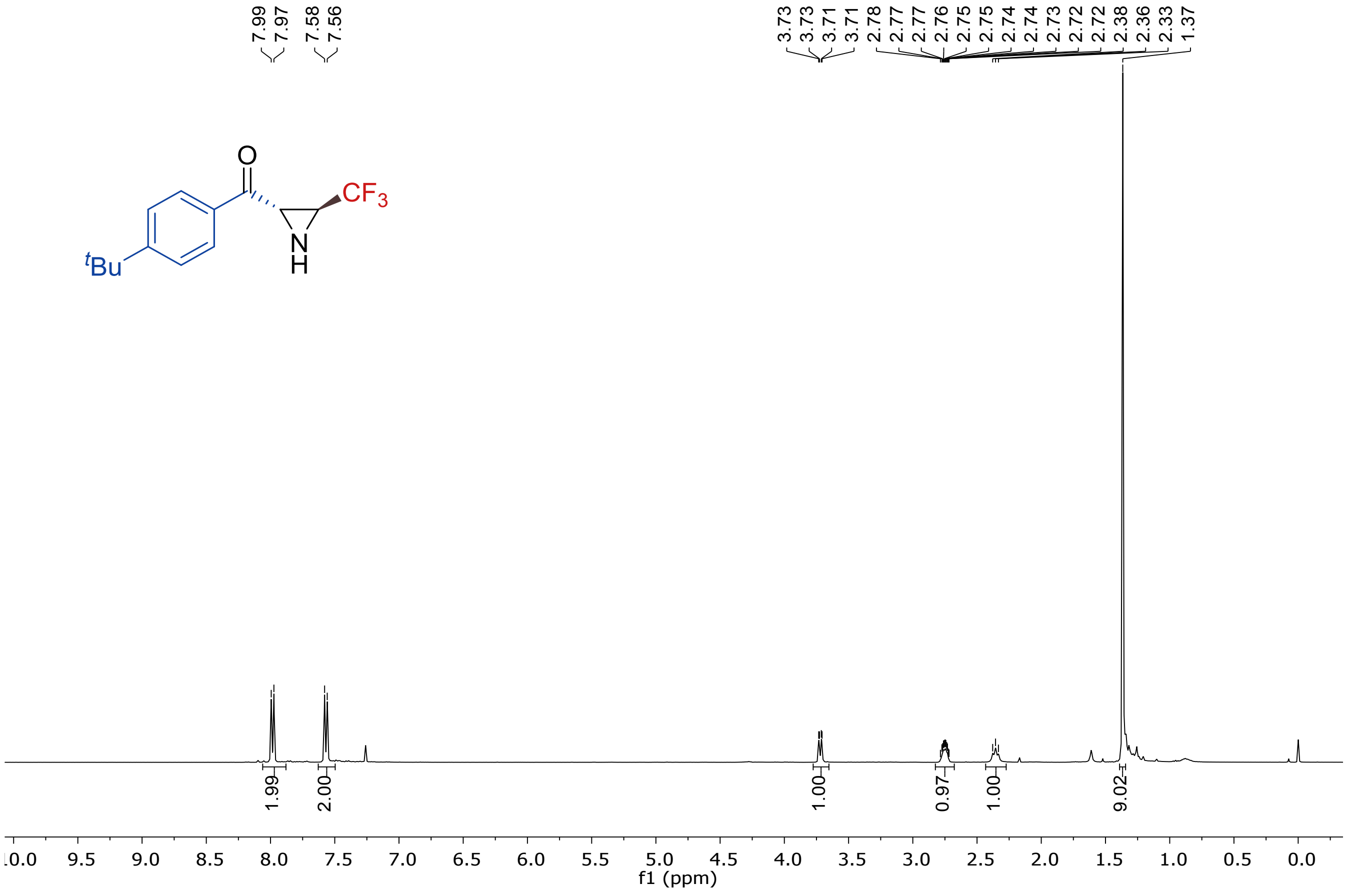

^{1}H NMR (400 MHz, $CDCl_3$) of compound **31**.

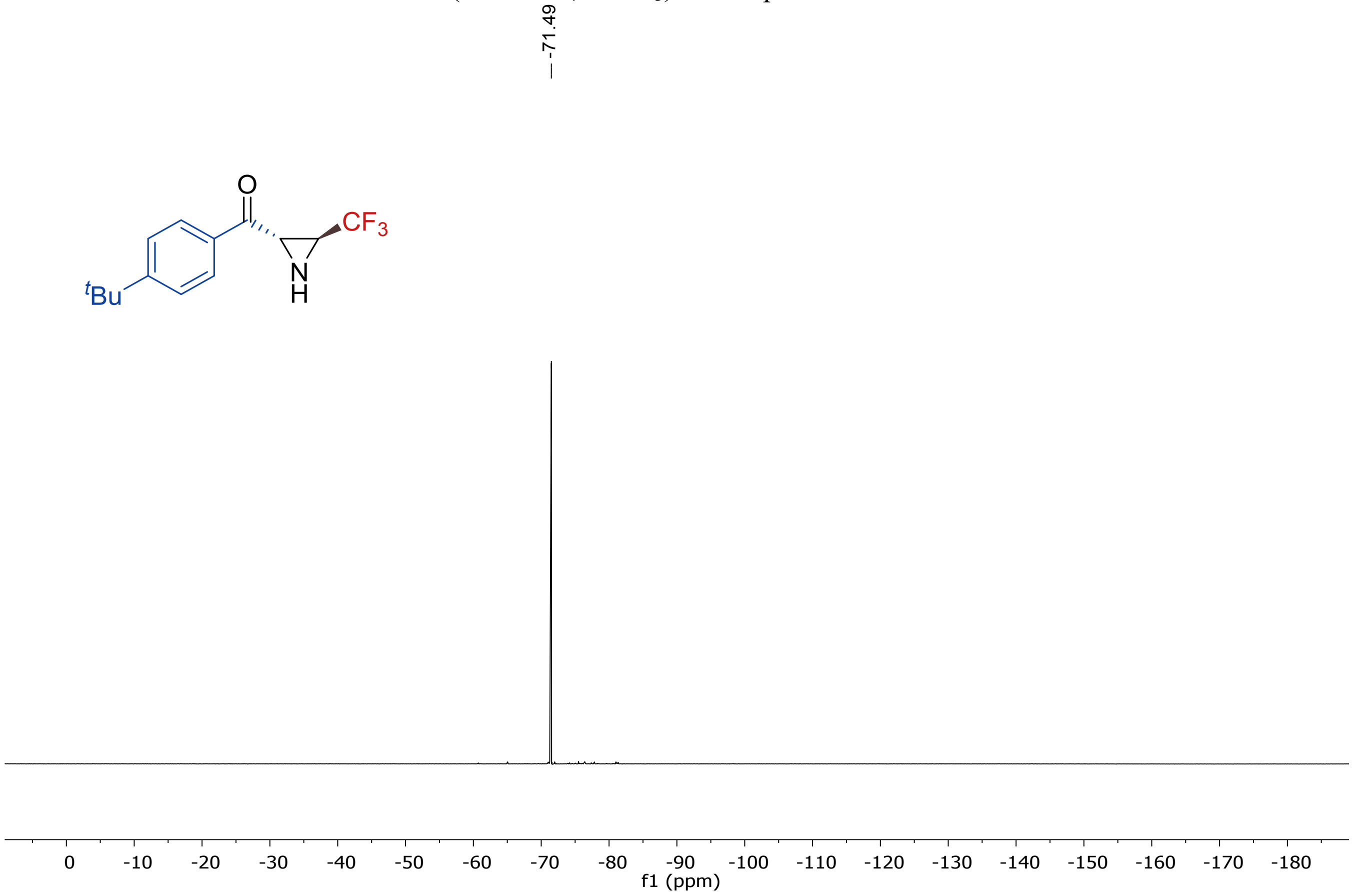

^{19}F{^{1}H} NMR (377 MHz, $CDCl_3$) of compound **31**.
